# Genetically instrumented LDL‐cholesterol lowering and multiple disease outcomes: A Mendelian randomization phenome‐wide association study in the UK Biobank

**DOI:** 10.1111/bcp.15793

**Published:** 2023-06-06

**Authors:** Kitty Pham, Anwar Mulugeta, Amanda Lumsden, Elina Hyppӧnen

**Affiliations:** ^1^ Australian Centre for Precision Health, Clinical & Health Sciences University of South Australia Adelaide South Australia Australia; ^2^ South Australian Health and Medical Research Institute Adelaide South Australia Australia; ^3^ Department of Pharmacology and Clinical Pharmacy, College of Health Sciences Addis Ababa University Addis Ababa Ethiopia

**Keywords:** cholesterol, lipid lowering, low‐density lipoprotein, Mendelian randomization, phenome‐wide, statins, UK Biobank

## Abstract

**Aims:**

Lipid‐lowering medications are widely used to control blood cholesterol levels and manage a range of cardiovascular and lipid disorders. We aimed to explore the possible associations between LDL lowering and multiple disease outcomes or biomarkers.

**Methods:**

We performed a Mendelian randomization phenome‐wide association study (MR‐PheWAS) in 337 475 UK Biobank participants to test for associations between four proposed LDL‐C‐lowering genetic risk scores (*PCSK9*, *HMGCR*, *NPC1L1* and *LDLR*) and 1135 disease outcomes, with follow‐up MR analyses in 52 serum, urine, imaging and clinical biomarkers. We used inverse‐variance weighted MR in the main analyses and complementary MR methods (weighted median, weighted mode, MR‐Egger and MR‐PRESSO) as sensitivity analyses. We accounted for multiple testing with false discovery rate correction (*P* < 2.0 × 10^−4^ for phecodes, *P* < 1.3 × 10^−2^ for biomarkers).

**Results:**

We found evidence for an association between genetically instrumented LDL lowering and 10 distinct disease outcomes, suggesting potential causality. All genetic instruments were associated with hyperlipidaemias and cardiovascular diseases in the expected directions. Biomarker analyses supported an effect of LDL‐C lowering through *PCSK9* on lung function (FEV [beta per 1 mg/dL lower LDL‐C −1.49, 95% CI −2.21, −0.78]; FVC [−1.42, 95% CI −2.29, −0.54]) and through *HMGCR* on hippocampal volume (beta per 1 mg/dL lower LDL‐C 6.09, 95% CI 1.74, 10.44).

**Conclusions:**

We found genetic evidence to support both positive and negative effects of LDL‐C lowering through all four LDL‐C‐lowering pathways. Future studies should further explore the effects of LDL‐C lowering on lung function and changes in brain volume.

**What is already known about this subject**

- Lipid‐lowering medications control blood cholesterol levels through a range of different pathways.
- Unfavourable blood lipid levels are known to be associated with cardiovascular disease, high blood pressure, diabetes and older age.
- Genetic analyses can help with drug safety profiling, by uncovering associations with disease outcomes and biomarkers.

**What this study adds**

- Our study is the first to compare LDL‐C lowering to a range of clinical and heart and brain MRI imaging biomarkers.
- There was no evidence for adverse disease associations, except diarrhoea. However, the PCSK9‐inhibitor proxy was associated with lower lung capacity, requiring further investigation.
- The statin proxy was associated with higher hippocampal volumes, potentially suggesting benefits for brain health.

## 1 INTRODUCTION

Lipid‐lowering medications are a widely prescribed category of medication used to control blood cholesterol levels and manage a range of vascular diseases.^1^ Unfavourable blood lipid levels (high total cholesterol [TC], high LDL‐cholesterol [LDL‐C], low HDL‐cholesterol [HDL‐C] and high triglycerides [TG]) are known to be associated with cardiovascular disease, high blood pressure, diabetes and older age.^2^ The most common type of lipid‐lowering medication is statins, which act on liver enzymes to downregulate the production of LDL‐C.^3^ Statins act by binding to 3‐hydroxy‐3‐methylglutaryl CoA (HMG‐CoA) reductase and inhibiting its function. There are 7–8 million adults currently taking statins within the United Kingdom.^4^ Other common LDL‐C‐lowering medications include cholesterol absorption inhibitors (ezetimibe), bile acid sequestrants (cholestyramine, colestipol) and proprotein convertase subtilisin/kexin type 9 (PCSK9) inhibitors (alirocumab, evolocumab).^1^

Lowering LDL‐C is known to be beneficial for cardiovascular health. A recent meta‐analysis on LDL‐C‐lowering therapies confirmed that the reductions in LDL‐C caused by lipid‐lowering medications were also associated with decreased rates of cardiovascular events.^5^ Recently, researchers have shifted their focus to *PCSK9*, a newer drug target which lowers LDL‐C by inactivating the PCSK9 protein in the liver and promoting the destruction of LDL‐C.^6^ Lowering LDL‐C through the PCSK9 inhibition pathway has been reported to have both risk‐increasing and risk‐decreasing disease associations, including decreased risk of myocardial infarction^7^ and decreased cancer risk,^8^ but also increased risk of type 2 diabetes.^9^

Our study uses an MR‐PheWAS approach which combines phenome‐wide association (PheWAS) and Mendelian randomization (MR) analyses. The PheWAS allows us to screen a population for any associations between a single variant or combined genetic risk score (GRS) and a wide range of phenotypes.^10^ MR analyses use genetic variants, associated with the exposure variable, to investigate the effects of an environmental exposure on disease risk.^11^ Since genetic variants are determined at conception, the analytical method is largely unaffected by confounding factors and reverse causality, allowing us to make causal inferences.

In our data‐driven hypothesis‐free study, we examine the effects of LDL‐C lowering through four distinct pathways representing current or potential drug targets. We investigated the PCSK9 inhibition pathway using variants near the *PCSK9* gene, which provides a genetic proxy for PCKS9 inhibitor drugs such as alirocumab and evolocumab.^6^ *HMGCR* variants were used to proxy the effect of statin drugs, which function through the inhibition of the HMGCR (HMG‐CoA reductase) enzyme.^3^ Ezetimibe lowers LDL‐C through the cholesterol absorption pathway via NPC1L1 (Niemann–Pick C1–like 1) protein inhibition, which can be mimicked using *NPC1L1* variants.^12^ Finally, variants in *LDLR* encode a newer drug target for LDL‐C lowering.^13^ *LDLR* encodes the LDL‐receptor protein that contributes to LDL transport into the cells which decreases the level of circulating LDL‐C.^14^ Our analyses are conducted in up to 337 475 participants within the UK Biobank, and we screen for associations with 1135 diseases outcomes, and a broad range of clinical measures, blood, urine and imaging biomarkers.

## 2 METHODS

### 2.1 Study population: UK Biobank

The UK Biobank is a prospective, population‐based cohort, with deep genotypic and phenotypic data on 502 536 participants aged 37–73 years.^15^ The resource compiles lifestyle, physical, genetic and imaging data collected from questionnaires, physical measurements and blood and tissue samples. Participants were recruited in 22 assessment centres across Scotland, England and Wales between 2006 and 2010. Further details on participant recruitment and data collection have been extensively reported elsewhere.^15^ We restricted the analyses to unrelated individuals of white British ancestry (Figure S1). Our final analysis sample contained 337 475 participants from the UK Biobank cohort.

As a secondary analysis of UK Biobank data, this study relies on the consent of subjects at their participation with the UK Biobank data collection studies.^16^ Ethical approval for the UK Biobank was granted by the National Information Governance Board for Health and Social Care and North West Multicentre Research Ethics Committee (11/NW/0382). Participants in the study have provided electronic consent for use of their anonymised data and access to their medical records for health‐related research. All participants have the right to withdraw at any point, without explanation or penalty. The researchers of this study have gained approval for use of the database under UK Biobank application number 10171.

### 2.2 Genetic instruments for LDL lowering

The Global Lipids Genetics Consortium (GLGC) identifies 157 loci associated with serum lipid levels, including 57 loci associated with LDL‐C.^17^ We selected SNPs within 100 KB either side of four gene regions (PCSK9, HMGCR, NP1L1 and LDLR).^18^ Each SNP was independently associated with LDL‐C at a genome‐wide significance level (*P* < 5.0 × 10^−8^) within the GLGC and had a linkage disequilibrium of *r* ^2^ < .2. For *NPC1L1*, rs2073547 was excluded due to evidence of deviation from the Hardy–Weinberg equilibrium (P_HWE = 7.5 × 10^−13^) (Table S1). Each SNP was coded based on the number of LDL‐C decreasing alleles (0, 1 or 2). Four GRS were constructed for *PCSK9*, *HMGCR*, *NPC1L1* and *LDLR*, to proxy the effect of different LDL‐C‐lowering medications (Table S2). GRS were determined by summing the risk alleles, which were weighted by the beta coefficient taken from variant‐LDL‐C association within the GLGC.

### 2.3 Phenome construction

Disease outcome information was collected from hospital admission electronic health records (EHR) and national death registers, including records up to 31 March 2017. All outcomes were coded according to the International Classification of Disease (ICD) versions 9 and 10 in the UK Biobank and mapped to a phenotype code (phecode). Full description of the phecode mapping process has been previously reported elsewhere.^19^ In our analyses, any phecodes with <200 cases in the analysis sample were excluded to maintain reasonable statistical power,^20^ leaving 1135 phecodes for analysis.

### 2.4 Biomarker data

Biomarker data from the UK Biobank baseline assessment and imaging sub‐phase were used, including serum markers, urine markers, clinical measurements and heart and brain MRI imaging. Serum and urine biomarkers (including cardiovascular, bone and joint, cancer, diabetes, renal and liver indicators) were collected from blood and urine samples at baseline.^21^ Body mass index (BMI) was calculated from height and weight measurements ((kg)/height (m)^2^), while body fat percentage was estimated from impedance measurements, both during baseline assessment.^15^, ^22^ Blood pressure was averaged from two automated readings at baseline. We accounted for the effect of blood pressure‐lowering medications by adding a correction constant of 15 mmHg to the systolic blood pressure values and 10 mmHg to the diastolic blood pressure values.^23^ Breath spirometry tests were performed at baseline to obtain the respiratory function measures.^15^ Brain and cardiac markers were taken from brain and heart MRI imaging data.^24^ Brain volume data were normalized for head size, and outlier values (±3SDs) were excluded for both brain and cardiac biomarkers.

### 2.5 Statistical analyses

Our main analyses were conducted in stages: (1) PheWAS of the disease outcomes; (2) two‐sample MR analysis of disease outcomes detected from the PheWAS; and (3) two‐sample MR analysis of related disease biomarkers. Firstly, a PheWAS approach was used to screen for any GRS–disease associations using GRS of four LDL‐C‐lowering targets. From over 1600 phecodes available within the UK Biobank, 1135 phecodes, within 18 disease categories, were investigated in our PheWAS (Tables S3 and S4). Using each GRS, we fitted a logistic regression with each disease outcome in a model adjusted for age, sex, assessment centre (as a dummy variable), SNP array (UK BiLEVE array or UK Biobank Axiom array) and 40 genetic principal components. We checked for any associations between each LDL‐C‐lowering GRS and known confounders (age, sex, smoking, alcohol consumption, physical activity, level of education and Townsend deprivation index). False discovery rate (FDR) correction was applied to account for multiple testing.^25^ This method determines the threshold by considering the ratio of false positive results to total positive test results, where false positives are determined as the 5% with the highest *P* values from the group of association with *P* < .05. We tested 1135 disease outcomes and four GRSs, leading to an FDR‐corrected *P* value threshold of 2.0 × 10^−4^.

We conducted two‐sample MR analyses on any GRS–disease associations that passed the FDR threshold in the first stage. Five MR methods were used: inverse‐variance weighted (IVW) MR, MR‐Egger, weighted median MR, weighted mode MR and MR‐PRESSO. Each method considers different levels of tolerance to horizontal pleiotropy, allowing us to assess whether associations are potentially causal or through other pathways. We checked for any distortion in the IVWMR estimates from outliers using leave‐one‐out analysis, and MR‐PRESSO outlier test, with additional evidence on horizontal pleiotropy from MR‐Egger intercept. For all analyses, the variant‐exposure estimates were taken from the GLGC, and variant‐outcome estimates were from the UK Biobank. Next, we repeated the two‐sample MR method using biomarker data to explore any underlying biological mechanisms that may explain observed associations with the disease outcomes. For sex‐dependant hormone biomarkers, we also performed sex stratified analyses. We calculated free testosterone and free oestradiol values using the Vermeulen equation and the Anderson equation, respectively.^26^, ^27^ An FDR‐corrected *P* value threshold of 1.3 × 10^−2^ was applied (calculated based on 52 biomarker outcomes and four GRSs).

We performed independent replication of identified GRS–disease and GRS–biomarker associations on MR‐Base using variant‐outcome association estimates available within the OpenGWAS repository. All replication analyses for disease outcomes were conducted in the FinnGen consortium (data release 4, 2020), comprised of >170 000 Finnish participants, which did not include overlap with the UK Biobank.^28^ We were able to conduct replication for 9 of 13 significant disease outcomes (including overlapping disease codes), which include hypercholesterolaemia, hyperlipidaemia, angina pectoris, aortic aneurysm, coronary atherosclerosis, hypertensive heart, ischaemic heart diseases, myocardial infarction and unstable angina pectoris.

Power estimations were calculated based on the method developed by Burgess.^29^ In our study, the *LDLR* GRS was adequately powered to detect a 20% increase in risk per 1 mg/dL decrease in LDL‐C for 10 phecodes, while all other GRS were unable to detect any phecodes (Table S5). For a 50% increase in risk, the *PCSK9* score was able to detect 17 phecodes, *HMGCR* score was able to detect 24 phecodes and the *LDLR* score was able to detect 186 phecodes. The power to detect 100% and 150% increases for each GRS are listed for all phecodes in Table S5. For all power calculations, we used a significance threshold *α* = 5% and power of 80%. We calculated the percentage variation in LDL‐C within our study population (UK Biobank) for each GRS: *r* ^2^_*PCSK9*_ = .12%, *r* ^2^_*HMGCR*_ = .15%, *r* ^2^_*NPC1L1*_ = .026% and *r* ^2^_*LDLR*_ = .64%.

Data management processes were conducted in STATA SE version 15, while all remaining analyses were performed in R version 3.6.1 software.^30^, ^31^ We utilized the PheWAS, MR‐PRESSO and two‐sample MR R packages.

### 2.6 Nomenclature of targets and ligands

Key protein targets and ligands in this article are hyperlinked to corresponding entries in http://www.guidetopharmacology.org and are permanently archived in the Concise Guide to PHARMACOLOGY 2019/20.^32^, ^33^

## 3 RESULTS

From the full UK Biobank cohort, we included 337 475 participants within our study sample. The sample was comprised of 53.7% women and 75.2% had above average self‐reported health (Table 1). According to the NHS guidelines for healthy cholesterol levels, nearly 70% of participants had above normal values for both TC and LDL‐C.^34^ Despite this, only 16.4% self‐reported the use of statin medication. As expected, the proportion of participants with above normal cholesterol and LDL‐C was significantly lower among statin users, compared to non‐users.

**TABLE 1:** Distribution of total cholesterol and LDL‐cholesterol across population characteristics, within our sub‐sample of the UK Biobank.

	*n* (%)	Total cholesterol				LDL‐direct cholesterol			
		Normal (%)	Above normal (%)	Missing (%)	*P* value	Normal (%)	Above normal (%)	Missing (%)	*P* value
Total population	337 475	25.8	69.5	4.7		25.4	69.8	4.8	
Sex					<1 × 10^−300^				3.6 × 10^−266^
Women	181 231 (53.7)	20.5	74.8	4.7		28.2	67.0	4.8	
Men	156 244 (46.3)	32.0	63.4	4.6		23.0	72.1	4.9	
Age (in years)					<1 × 10^−300^				<1 × 10^−300^
39–49 years	73 848 (21.9)	30.5	65.0	4.5		29.1	66.2	4.7	
50–59 years	128 289 (38.0)	21.4	74.0	4.6		21.3	73.9	4.8	
60–73 years	135 338 (40.1)	27.6	67.7	4.8		27.3	67.7	5.0	
BMI (in kg/m^2^)					1.1 × 10^−234^				7.4 × 10^−216^
Underweight, <18.5 kg/m^2^	1673 (0.5)	29.2	65.5	5.3		37.6	57.0	5.4	
Normal, (18.5, 25) kg/m^2^	109 862 (32.6)	24.6	70.8	4.6		27.3	67.9	4.8	
Overweight, (25, 30) kg/m^2^	143 745 (42.6)	24.3	71.1	4.6		22.8	72.4	4.8	
Obese, ≥30 kg/m^2^	81 101 (24.0)	30.1	65.1	4.8		27.0	68.0	5.0	
Missing	1094 (0.3)	33.7	59.7	6.6		33.0	60.0	7.0	
History of statin use					<1 × 10^−300^				<1 × 10^−300^
No	282 026 (83.6)	18.3	77.1	4.6		17.6	77.6	4.8	
Yes	55 449 (16.4)	64.5	30.8	4.7		65.0	30.1	4.9	
Self‐reported general health					<1 × 10^−300^				<1 × 10^−300^
Excellent	56 531 (16.8)	21.5	74.1	4.4		22.2	73.2	4.6	
Good	197 162 (58.4)	24.0	71.4	4.6		23.7	71.5	4.8	
Fair	68 619 (20.3)	31.9	63.3	4.8		30.5	64.5	5.0	
Poor	13 983 (4.1)	39.2	55.7	5.1		37.5	57.2	5.3	
Missing	1180 (0.4)	32.5	62.7	4.8		30.5	64.2	5.3	

*Note*: Normal ranges of cholesterol are defined using the NHS guidelines for cholesterol levels (https://www.nhs.uk/conditions/high‐cholesterol/cholesterol‐levels/). Recommended healthy levels are suggested to be below 90 mg/dL (5 mmol/L) for total cholesterol and below 54 mg/dL (3 mmol/L) for LDL‐cholesterol.

### 3.1 PheWAS analyses

The proposed GRSs were all significantly associated with LDL‐C (*P* < 1.8 × 10^−20^) (Table S2). The strongest association was between *LDLR* GRS LDL‐C, which explained 0.64% of variation in LDL‐C. The *PCSK9*, *HMGCR*, *NPC1L1* and *LDLR* scores were associated with lower LDL‐C, TC and apoB (Table S6). We found no associations between any GRSs and known confounders (Table S7).

**FIGURE 1:**
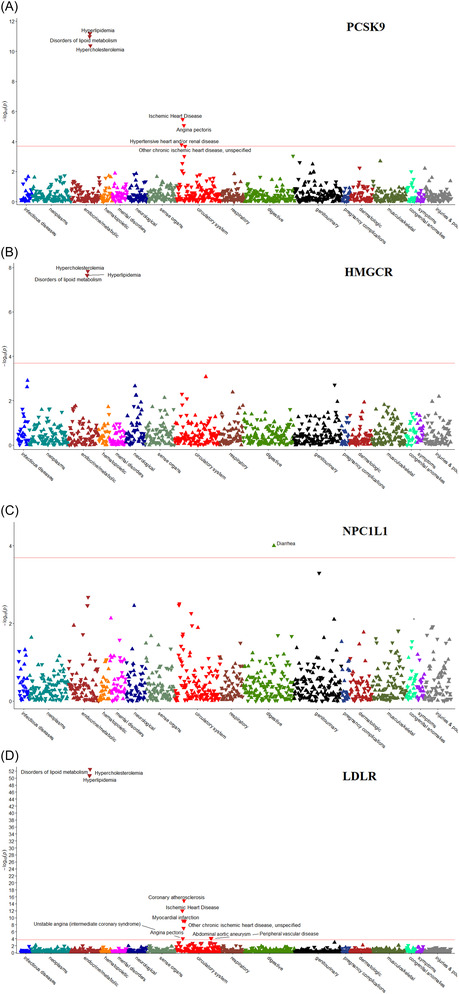
Manhattan plots for the phenome‐wide association analyses using LDL‐cholesterol‐lowering genetic risk scores: (A) PCSK9, (B) HMGCR, (C) NPC1L1 and (D) LDLR. For each LDL‐C‐GRS‐outcome association, a logistic model was used adjusting for age, sex, 40 principal components and SNP array. A higher LDL‐C‐lowering genetic risk score indicates lower serum LDL‐C levels. Red line: FDR threshold *P* = 2.0 × 10^−4^; downward triangles: OR < 1; upward triangles: OR ≥ 1.

Results from the PheWAS are shown using Manhattan plots (Figure 1). Across the four GRSs, we found significant signals for 13 disease outcomes which passed the 5% FDR threshold (*P* = 2.0 × 10^−4^). *PCSK9*, *HMGCR* and *LDLR* scores had strong associations with hyperlipidaemia, disorders of lipoid metabolism and hypercholesterolaemia (Figure 1A,B,D). *PCSK9* and *LDLR* also had significant associations with a range of cardiovascular outcomes. *NPC1L1* was only associated with diarrhoea (Figure 1C).

### 3.2 MR analyses of disease outcomes

**FIGURE 2:**
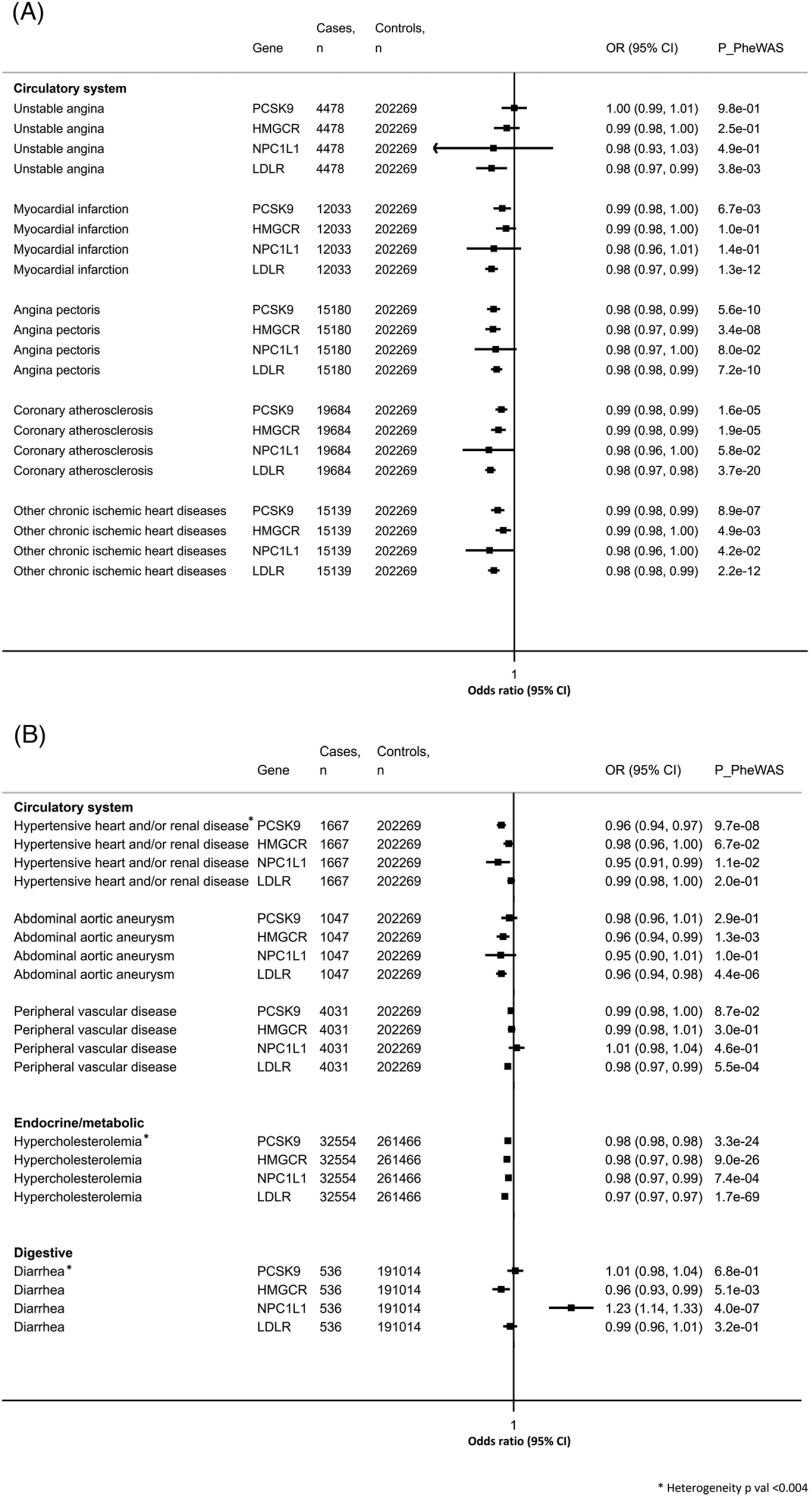
Forest plots for the Mendelian randomization analyses on the 10 distinct significant LDL‐C lowering–disease associations identified in the PheWAS using the LDL‐C‐lowering genetic instruments. Analyses using inverse‐variance weighted Mendelian randomization (IVWMR) are shown. Estimates are odds ratios (OR 95% CI) per 1 mg/dL lower LDL‐C. FDR threshold: *P* = 2.0 × 10^−4^.

After removing overlapping phecodes, we identified genetic evidence that suggests a causal association between at least one of four genetic instruments and 10 distinct diseases. All instruments were associated with hypercholesterolaemia in IVWMR analyses (Figure 2). We saw evidence for lower risks of at least one cardiovascular disease with all genetic instruments, with the most consistent associations seen between the LDLR instrument and coronary atherosclerosis (OR per 1 mg/dL decrease in LDL‐C 0.98, 95% CI 0.97, 0.98).

For all analyses, MR estimates were broadly similar across the weighted mode, weighted median and MR‐PRESSO methods, but not significant when using MR‐Egger regression (Table S8). We did not detect any unbalanced horizontal pleiotropy for any of the included SNPs, across all LDL‐C‐lowering targets (*P*_pleiotropy_ ≥ .27 for all, Table S8). We also found no evidence to suggest the presence of influential outliers using the leave‐one‐out and MR‐PRESSO tests (Figures S2–S5).

OpenWAS replication in the FinnGen cohort confirmed the associations between LDL lowering and hypercholesterolaemia, using the *HMGCR* genetic instrument and between LDL lowering and hypercholesterolaemia, hyperlipidaemia and unstable angina pectoris, using the LDLR genetic instrument (Table S9).

### 3.3 MR analyses of disease biomarkers

**FIGURE 3:**
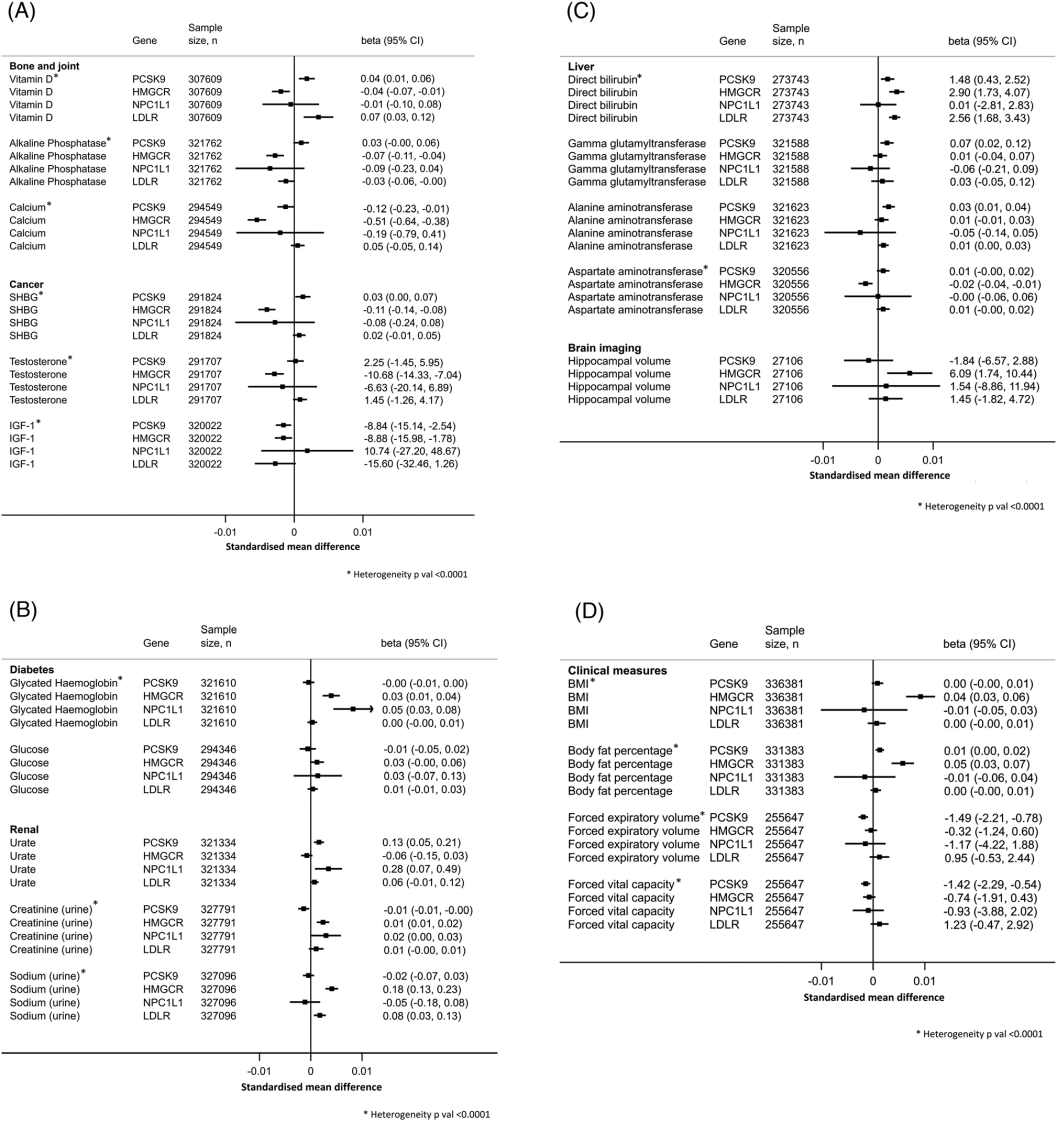
Forest plots for the Mendelian randomization analyses on the 20 significant LDL‐C lowering–biomarker associations, which pass the FDR threshold, using the LDL‐C‐lowering genetic instruments. Analyses using inverse‐variance weighted Mendelian randomization (IVWMR) are shown. Graphs are presented as standardized mean difference in biomarker levels for each GRS (to compare the estimates with the same scale) and estimates shown on the right are absolute beta values in their respective units (beta 95% CI) per 1 mg/dL lower LDL‐C. FDR threshold: *P* = 1.3 × 10^−2^. (A) Absolute beta values are presented in nmol/L (25(OH)D); IU/L (alkaline phosphatase); μmol (calcium); nmol/L (SHBG); pmol/L (testosterone) or pmol/L (IGF‐1) per 1 mg/dL lower LDL‐C. (B) Absolute beta values are presented in mmol/mol (HbA1c); mg/dL (glucose); μmol/L (urate); mmol/L (urine creatinine) or mmol/L (urine sodium) per 1 mg/dL lower LDL‐C. (C) Absolute beta values are presented in nmol/L (direct bilirubin); U/L (gamma glutamyltransferase); U/L (alanine aminotransferase); U/L (aspartate aminotransferase) or mm^3^ (hippocampal volume) per 1 mg/dL lower LDL‐C. (D) Absolute beta values are presented in kg/m^2^ (BMI); % (body fat percentage); mL (FEV) or mL (FVC) per 1 mg/dL lower LDL‐C.

In the final stage of our analyses, we explored associations with a range of serum, urine, body composition, blood pressure, spirometry, cardiac imaging and brain imaging biomarkers (Table S10). There was variation in the effects of lower LDL‐C on bone and joint health. *PCSK9* and *LDLR* were associated with higher 25‐hydroxyvitamin D (25(OH)D), while *HMGCR* was associated with lower 25(OH)D, lower alkaline phosphatase and slightly lower calcium (Figure 3A). The *HMGCR* genetic instrument identified cancer‐related biomarker associations between lower LDL‐C and lower sex hormone‐binding globulin (SHBG) (beta in nmol/L per 1 mg/dL decrease in LDL‐C −0.11, 95% CI −0.14, −0.08) and slightly lower testosterone (beta in pmol/L per 1 mg/dL decrease in LDL‐C −10.68, 95% CI −14.33, −7.04), while the *PCSK9* instrument was associated with lower IGF‐1. In sex stratified analyses, we found that *HMGCR* was associated with lower levels of total, and free, testosterone only in men, while the association between *HMGCR* and lower SHBG was only significant in women (Figure S6). LDL‐C lowering was associated with higher HbA1c, an indicator for increased risk of diabetes, for both *HMGCR* (beta in mmol/mol per 1 mg/dL decrease in LDL‐C 0.03, 95% CI 0.01, 0.04) and *NPC1L1* (beta 0.05, 95% CI 0.03, 0.08) (Figure 3B). For the renal biomarkers, *PCSK9* was associated with higher urate, *HMGCR* with higher urine creatinine and urine sodium and *LDLR* with higher urine sodium only.

There was genetic evidence to suggest a causal association between LDL‐C lowering and slightly lower direct bilirubin, which was consistent across three of four genetic instruments (Figure 3C). *PCSK9* was associated with higher gamma glutamyltransferase and higher alanine aminotransferase, and *HMGCR* was associated with lower aspartate aminotransferase. *HMGCR* and higher hippocampal volume was the only imaging biomarker association to pass the FDR correction (beta in mm^3^ per 1 mg/dL decrease in LDL‐C 6.09, 95% CI 1.74, 10.44). In our analyses of the clinical biomarkers, LDL‐C lowering proxied by *HMGCR* was associated with higher body fat measures (Figure 3D). There was evidence to support an association between *PCSK9* and spirometry test indicators for lower lung capacity (FEV1 beta in mL per 1 mg/dL decrease in LDL‐C −1.49, 95% CI −−2.21, −0.78; FVC beta −1.42, 95% CI −2.29, −0.54).

Results are shown for all biomarkers and for all MR methods in Tables S11–S14.

## 4 DISCUSSION

Our analyses confirmed the known associations between the LDL‐C‐lowering effect of statins and a range of metabolic and cardiovascular diseases. There was no evidence for adverse effects of lipid lowering, aside from confirming diarrhoea as a side effect of ezetimibe (targets *NPC1L1*)^35^ and suggestion for novel associations between the *PCSK9* genetic instrument and lower lung capacity (measured by FEV1 and FVC). Interestingly, LDL‐C lowering by *HMGCR* was associated with a higher hippocampal volume, which may support proposed benefits with respect to reduced dementia and depression risk.^36^, ^37^

Sinnott‐Armstrong et al.^38^ have conducted an MR analysis of the blood and urine biomarkers in the UK Biobank. They identified 51 causal relationships, including 32 disease associations. The study assessed genetic associations through GWAS, PheWAS and MR analyses. Consistent with our findings, *PCSK9* and *LDLR* were correlated with cardiovascular biomarkers, and *NPC1L1* with cardiovascular and hormone biomarkers in the biomarker phenotype distribution analyses; however, these genes were not reported on in subsequent analysis phases as alternate genes had stronger associations with the biomarkers. In our study, we additionally explored a wide range of diseases using phecode data and a wider range of biomarkers including clinical, cardiac imaging and brain imaging markers.

Recent PheWAS studies have focused on *PCSK9*, and all confirm the known association between PCSK9‐inhibitor lipid‐lowering medication and decreased risk of hypercholesterolaemia, hyperlipidaemia and cardiovascular disease.^9^, ^39^, ^40^ Our study identified strong associations between lower LDL‐C and lipid‐related metabolic diseases, which was consistent across most GRSs. This is as expected since disorders of lipoid metabolism, hyperlipidaemia and hypercholesterolaemia are known to be caused by unfavourable lipid profiles.^41^ Similarly, the relationship between LDL‐C and cardiovascular diseases is well known and supported by a recent meta‐analysis.^5^ Decreasing LDL‐C reduces the risk of cardiovascular disease by decreasing the atherosclerotic plaque build‐up on the artery walls.^42^ By scaling our estimates to represent a clinically relevant decrease in LDL‐C of 20 mg/dL, which is comparable to a low dose of statin medications,^43^ our results suggest that LDL‐C lowering through these targets may reduce risk of myocardial infarction by up to 33% (OR per 20 mg/dL 0.67) and peripheral vascular disease by up to 34% (OR per 20 mg/dL 0.66).

The only negative side effect identified in our phecode analysis was the relationship between *NPC1L1* and increased risk of diarrhoea, which can be explained by the NPC1L1 protein's effect on LDL‐C by inhibiting cholesterol absorption in the gastrointestinal tract.^12^ There are some previously reported associations between LDL‐C lowering and risk of disease which were not flagged within our study. Carter et al. found an association between genetic variants in the *HMGCR* gene region, a proxy for statins, and reduced overall cancer risk, but no associations with other statin‐related gene targets such as *PCSK9*, *LDLR* and *NPC1L1*.^8^ Similarly, an earlier study found evidence to support an association between the *HMGCR* gene and decreased risk of prostate, breast and ovarian cancers.^44^, ^45^ Although our MR‐PheWAS of the phecodes did not identify a significant association with any cancers, the biomarker analyses found an association between *HMGCR* (statin proxy) and lower levels of testosterone and SHBG. The relationship between these sex hormones and cancer risk is not consistent with all cancers; a recent study linked low serum testosterone in men with lower risk of prostate cancer.^46^ The exact mechanism is still unknown; however, studies suggest that statins may interrupt feedback from the pituitary glands to the testicles, signalling a decrease in production of testosterone.^47^ Our study also identified an association between PCSK9 inhibitors and lower IGF‐1, which may indicate a decrease in cancer risk, since IGF‐1 is implicated in the growth and proliferation of cancer cells.^48^ Our study may not be adequately powered in the phecode analyses to observe the cancer disease associations.

The relationship between LDL‐C and bone health biomarkers is still unclear. It is commonly suggested that 25(OH)D deficiency is linked with increased risk of hyperlipidaemia and cardiovascular disease^49^, ^50^; however, a recent study in the National Health and Nutrition Examination Survey database found that statin users had significantly higher 25(OH)D levels compared to non‐users.^51^ We found an association between the statin proxy and lower levels of vitamin D, alkaline phosphatase and calcium, while *PCSK9* and *LDLR* were associated with higher 25(OH)D. Kane et al. suggested that the relationship between LDL‐C and 25(OH)D is through the same pathway as statin medications, whereby vitamin D metabolites inhibit HMG‐CoA reductase to decrease cholesterol synthesis, inhibit CYP51A1 and interrupt cholesterol biotransformation.^52^ It is also possible that *HMGCR* inhibition inhibits 7‐dehydrocholesterol synthesis, which acts as a precursor to both cholesterol and vitamin D.^53^

In line with earlier studies by Ference et al.^54^ and Lotta et al.,^55^ the *HMGCR* and *NPC1L1* genetic instruments were associated with HbA1c (glycated haemoglobin), an indicator of increased diabetes risk.^56^ The association between *HMGCR* and lower SHBG is consistent with findings for HbA1c. Previous studies report that low SHBG is associated with obesity, insulin resistance and increased risk of metabolic diseases, such as diabetes.^57^, ^58^, ^59^ Our analyses of the clinical biomarkers identified an association between the statin proxy and higher BMI and body fat percentage. Although weight gain is debated as a direct side effect of taking statin medications, a cross‐sectional study of over 27 000 statin users in the US found that statin users compared to non‐users had increased caloric and fat intake and faster increase in BMI.^60^

We also found evidence to support an association between the *PCSK9* genetic instrument and lower FEV1 and FVC, which can indicate poor lung function and obstructive pulmonary diseases.^61^ In a mice study, PCSK9 expression was shown to be involved in the metastasis process of melanoma cells into lung epithelial cells, while study of the human lung cells found that PCSK9 had an anti‐apoptotic effect on cancer cells.^62^, ^63^ Similarly, clinical study of 803 elderly men found that statin use had a protective effect and attenuated yearly decline in FEV1 and FVC.^64^ LDL‐C is known to play a role in supplying cholesterol to lung cells and inhibiting local cholesterol biosynthesis so a causal effect appears biologically plausible, and it is possible that reduced availability of LDL‐C for lung cells upregulates local cholesterol biosynthesis, disrupting normal lung function.^65^ However, we did not find any association with respiratory diseases in the phecode analyses, so further investigation is needed to fully understand the FEV1 and FVC associations.

We found an association between the *HMGCR* genetic instrument and slightly higher hippocampal volume, which was the only imaging biomarker to pass FDR correction. To our knowledge, the association is yet to be reported in human subjects; however, a study performed in mice found that long‐term use of simvastatin impaired synaptic plasticity within the hippocampus.^66^ Hippocampal volumes have been shown to be clinically significant markers for risk of dementia, highlighting the need for future studies to confirm and further explain this association.^37^ In our biomarker MR analyses, *PCSK9* was associated with higher WMH volumes; however, the association was not significant after FDR correction. WMHs are an indicator of brain lesions and are known to be strong indicators of cognitive impairment, depression, dementia and stroke.^67^ Previous genetic studies of LDL‐C lowering via *PCSK9* reported detrimental effects on risk of Alzheimer's disease and depression.^68^, ^69^ Given that in the UK Biobank the neuroimaging biomarkers have been collected from a significantly smaller sample size (*n* ≤ 27 117) than the serum, urine and clinical markers, we may see an association between *PCSK9* and biomarkers of dementia or cognitive impairment as the number of participants in the imaging sub‐study increases.

One of the main strengths of our study is the large sample size and the availability of linked EHRs and mortality data. The PheWAS allows us to screen for a wide range of disease associations. Meanwhile, the application of MR analyses allows us to establish evidence for causality in a more feasible and cost‐effective manner than in randomized controlled trials.^70^ We use a range of MR methods and sensitivity analyses to detect pleiotropic effects and any potential biases. To our knowledge, our study is the first to compare LDL‐C lowering to not only the UK Biobank blood and urine markers, but also to a range of clinical and heart and brain MRI imaging biomarkers. Our study also allows for the comparison between different LDL‐lowering medication pathways and to observe their effects on disease outcomes and biomarkers.

It is also important to acknowledge the weaknesses of our study. Our study sample is comprised of only older participants with a white British ethnic background; hence, caution should be exercised when generalizing the results to the other populations. Healthy volunteer bias is known to be present in the UK Biobank.^71^ Power analyses showed that we were only powered to detect relatively large effects in disease outcomes, meaning that any mild or rare effects may be missed. Although the population available for analyses on disease outcomes was large (*N* ~ 337 000, up to 32 554 cases), the sample sizes available for the imaging outcomes were notably smaller, likely limiting the ability to detect associations (*n* < 27 106). We mentioned methods to detect pleiotropic effects; however, we cannot completely exclude bias due to pleiotropy, nor account for the effect of residual genetic confounding. MR analyses are designed to detect linear increases in LDL‐C‐lowering effects.^11^ It cannot accurately capture non‐linear associations and tends to underestimate the higher range of LDL‐C‐lowering effects. We used univariable MR to investigate the association of LDL‐C lowering on disease outcomes, and it is possible that some of the associations are mediated by factors such as BMI and blood pressure. Genetic instruments can only approximate average effects of LDL‐C lowering in an individual's lifetime but does not accurately reflect the complex changes in LDL‐C that can occur throughout life. Additionally, the genetic instruments were selected based on current and potential drug targets for LDL‐C‐lowering medications and were only weakly associated with LDL‐C. As sex‐specific genetic instruments were not available, we conducted sex stratified analyses using the overall GRS(s), assuming similar genetic association in men and women. We were unable to conduct OpenGWAS replication for all outcomes, and for outcomes that were available for replication analyses, sample sizes were considerably smaller than in our study.

In conclusion, we confirmed many of the known associations between LDL‐C‐lowering effects of statin medication and a range of metabolic and cardiovascular diseases. Our biomarker analyses suggested novel associations between the PCSK9‐inhibitor proxy and lower lung function (lower FEV1 and lower FVC) and between the statin proxy and higher hippocampal volumes. Future studies should aim to further investigate the effects of lipid lowering on lung function and brain volume, particularly in clinical settings.

## AUTHOR CONTRIBUTIONS

Kitty Pham analysed the data and prepared the first draft and conceptualized the study with Elina Hyppӧnen conceptualized the study. Anwar Mulugeta and Elina Hyppӧnen advised on data analyses. Kitty Pham, Anwar Mulugeta, Amanda Lumsden and Elina Hyppӧnen interpreted results, revised the paper and approved the manuscript for submission.

## CONFLICT OF INTEREST STATEMENT

The authors do not have any conflicts of interest to declare.

## Supporting information


**Table S1.** Association with LDL‐cholesterol for the 21 LDL‐C lowering variants (one variant excluded from GRS) used to construct the genetic risk score in the UK Biobank and the GLGC consortium (for *PCKS9*, *HMGCR*, *NPC1L1* and *LDLR*).
**Table S2.** Construction of LDL‐C lowering genetic risk scores.
**Table S3.** Disease categories and the number of cases with a phecode.
**Table S4.** List of 1135 phecodes tested in the PheWAS analyses.
**Table S5.** Power to detect associations with disease outcomes based on available numbers of cases, alpha 5%, instrument strength determined from the UK Biobank (PCSK9 r^2^ = 0.12%; HMGCR r^2^ = 0.15%; NPC1L1 r^2^ = 0.026%; LDLR r^2^ = 0.64%) and four different assumed effect sizes per 1 mg/dL decrease in LDL‐C (beta = 0.2; beta = 0.5; beta = 1.0; beta = 1.5). Phenotypes are listed by phecode number, as specified in Table S4.
**Table S6.** Associations of each genetic instrument (*PCSK9*, *HMGCR*, *NPC1L1* and *LDLR*) with cardiovascular related biomarkers (from the IVWMR analyses).
**Table S7.** Associations between each LDL‐C lowering genetic risk score and known confounders.
**Table S8.** MR analyses using MR IVW, weighted medium, weighted mode, MR‐Egger and MR‐PRESSO approaches, for 13 disease outcomes identified in the PheWAS of LDL‐C lowering variants under FDR corrected thresholds.
**Table S9.** Replication of MR analyses for disease phenotypes available in the MR‐Base repository.
**Table S10.** List of 52 serum, cardiac imaging, brain imaging and clinical biomarkers.
**Table S11.** MR analyses using MR IVW, weighted medium, weighted mode, MR‐Egger and MR‐PRESSO approaches, for all 52 serum, urine, cardiac imaging, brain imaging and clinical biomarkers, using the *PCSK9* genetic instrument.
**Table S12.** MR analyses using MR IVW, weighted medium, weighted mode, MR‐Egger and MR‐PRESSO approaches, for all 52 serum, urine, cardiac imaging, brain imaging and clinical biomarkers, using the *HMGCR* genetic instrument.
**Table S13.** MR analyses using MR IVW, weighted medium, weighted mode, MR‐Egger and MR‐PRESSO approaches, for all 52 serum, urine, cardiac imaging, brain imaging and clinical biomarkers, using the *NPC1L1* genetic instrument.
**Table S14.** MR analyses using MR IVW, weighted medium, weighted mode, MR‐Egger and MR‐PRESSO approaches, for all 52 serum, urine, cardiac imaging, brain imaging and clinical biomarkers, using the *LDLR* genetic instrument.
**Figure S1.** Participant flowchart showing the sample restriction from the full UK Biobank cohort to the final analysis sample for the MR‐PheWAS analysis.
**Figure S2.** Plots for the 10 distinct LDL‐C‐disease associations significant under FDR correction, for the *PCSK9* genetic risk score.
**Figure S3.** Plots for the 10 distinct LDL‐C‐disease associations significant under FDR correction, for the *HMGCR* genetic risk score.
**Figure S4.** Plots for the 10 distinct LDL‐C‐disease associations significant under FDR correction, for the *NPC1L1* genetic risk score.
**Figure S5.** Plots for the 10 distinct LDL‐C‐disease associations significant under FDR correction, for the *LDLR* genetic risk score.
**Figure S6.** Forest plots for the Mendelian randomization analyses on four LDL‐C lowering hormone biomarker associations, using the LDL‐C lowering genetic instruments and stratified by sex. Analyses using inverse‐variance weighted Mendelian randomization (IVWMR) are shown. Estimates are odds ratios (OR 95% CI) per 1 mg/dL lower LDL‐cholesterol.

## Data Availability

All data supporting this study will be available to approved users of the UK Biobank upon application.
