# Supplementary material for: Genetically instrumented LDL‐cholesterol lowering and multiple disease outcomes: A Mendelian randomization phenome‐wide association study in the UK Biobank
Source: Br J Clin Pharmacol. 2023 Jun 6;89(10):2992–3004. doi: 10.1111/bcp.15793 (PMC10952153; doi:10.1111/bcp.15793)

Online data supplement

Contents (Tables):

**Supplementary table 1.** Association with LDL-cholesterol for the 21 LDL-C lowering variants (1 variant excluded from GRS) used to construct the genetic risk score in the UK Biobank and the GLGC consortium (for *PCKS9*, *HMGCR*, *NPC1L1*, and *LDLR*)	**3**
**Supplementary table 2.** Construction of LDL-C lowering genetic risk scores	**4**
**Supplementary table 3.** Disease categories and the number of cases with a phecode	**5**
**Supplementary table 4.** List of 1135 phecodes tested in the PheWAS analyses	**6-13**
**Supplementary table 5.** Power to detect associations with disease outcomes based on available numbers of cases, alpha 5%, instrument strength determined from the UK Biobank (PCSK9 r2=0.12%; HMGCR r2=0.15%; NPC1L1 r2=0.026%; LDLR r2=0.64%) and four different assumed effect sizes per 1mg/dL decrease in LDL-C (beta=0.2; beta=0.5; beta=1.0; beta=1.5). Phenotypes are listed by phecode number, as specified above in Supplementary Table 4.	**14-29**
**Supplementary table 6.** Associations of each genetic instrument (*PCSK9*, *HMGCR*, *NPC1L1*, and *LDLR*) with cardiovascular related biomarkers (from the IVWMR analyses).	**30**
**Supplementary table 7.** Associations between each LDL-C lowering genetic risk score and known confounders.	**31**
**Supplementary table 8.** MR analyses using MR IVW, weighted medium, weighted mode, MR-Egger and MR-PRESSO approaches, for 13 disease outcomes identified in the PheWAS of LDL-C lowering variants under FDR corrected thresholds.	**32**
**Supplementary table 9.** Replication of MR analyses for disease phenotypes available in the MR-Base repository.	**33**
**Supplementary table 10.** List of 52 serum, cardiac imaging, brain imaging and clinical biomarkers.	**34**
**Supplementary table 11.** MR analyses using MR IVW, weighted medium, weighted mode, MR-Egger and MR-PRESSO approaches, for all 52 serum, urine, cardiac imaging, brain imaging and clinical biomarkers, using the *PCSK9* genetic instrument	**35**
**Supplementary table 12.** MR analyses using MR IVW, weighted medium, weighted mode, MR-Egger and MR-PRESSO approaches, for all 52 serum, urine, cardiac imaging, brain imaging and clinical biomarkers, using the *HMGCR* genetic instrument	**36**
**Supplementary table 13.** MR analyses using MR IVW, weighted medium, weighted mode, MR-Egger and MR-PRESSO approaches, for all 52 serum, urine, cardiac imaging, brain imaging and clinical biomarkers, using the *NPC1L1* genetic instrument	**37**
**Supplementary table 14.** MR analyses using MR IVW, weighted medium, weighted mode, MR-Egger and MR-PRESSO approaches, for all 52 serum, urine, cardiac imaging, brain imaging and clinical biomarkers, using the *LDLR* genetic instrument	**38**

Contents (Figures):

**Supplementary figure 1.** Participant flowchart showing the sample restriction from the full UK Biobank cohort to the final analysis sample for the MR-PheWAS analysis.	**39**
**Supplementary figure 2.** Plots for the 10 distinct LDL-C-disease associations significant under FDR correction, for the *PCSK9* genetic risk score.	**40-44**
**Supplementary figure 3.** Plots for the 10 distinct LDL-C-disease associations significant under FDR correction, for the *HMGCR* genetic risk score.	**45-49**
**Supplementary figure 4.** Plots for the 10 distinct LDL-C-disease associations significant under FDR correction, for the *NPC1L1* genetic risk score.	**50-54**
**Supplementary figure 5.** Plots for the 10 distinct LDL-C-disease associations significant under FDR correction, for the *LDLR* genetic risk score.	**55-59**
**Supplementary figure 6.** Forest plots for the mendelian randomisation analyses on 4 LDL-C lowering hormone biomarker associations, using the LDL-C lowering genetic instruments and stratified by sex. Analyses using inverse-variance weighted mendelian randomisation (IVWMR) are shown. Estimates are odds ratios (OR 95% CI) per 1mg/dL lower LDL-cholesterol.	**60**

Supplementary Table 1: Association with LDL-cholesterol for the 21 LDL-C lowering variants (1 variant excluded from GRS) used to construct the genetic risk score in the UK Biobank and the GLGC consortium.

							Change in LDL-C per risk allele (UKB)^				Change in LDL-C per one risk allele (GLGC)^			
**SNP**	**Gene**	**Chr**	**Effect allele**	**Other allele**	**Missing (UKB)**	**P_HWE (UKB)**	**EAF**	**Beta (mg/dL)**	**SE**	**P**	**EAF**	**Beta (mg/dL)**	**SE**	**P**
rs11206510	PCSK9	1	C	T	0.00	0.05	0.19	-1.538	0.108	<1E-300	0.15	-2.659	0.005	2.4E-53
rs2479409	PCSK9	1	A	G	0.00	0.37	0.65	-1.121	0.089	9.5E-37	0.67	-2.054	0.004	2.5E-50
rs2149041	PCSK9	1	C	G	0.82	0.30	0.81	-1.420	0.109	7.0E-39	0.84	-2.035	0.005	1.4E-35
rs2479394	PCSK9	1	A	G	0.00	0.76	0.72	-0.837	0.094	6.7E-19	0.72	-1.235	0.004	1.6E-19
rs10888897	PCSK9	1	T	C	0.00	0.94	0.39	-0.983	0.086	5.7E-30	0.39	-1.622	0.004	8.4E-31
rs7552841	PCSK9	1	C	T	12.49	0.01	0.63	-0.777	0.094	1.3E-16	0.63	-1.178	0.004	5.4E-15
rs562556	PCSK9	1	G	A	0.00	0.75	0.18	-0.842	0.110	1.8E-14	0.19	-2.048	0.007	6.2E-21
rs12916	HMGCR	5	T	C	0.00	0.76	0.60	-2.118	0.086	<1E-300	0.57	-2.346	0.122	7.8E-78
rs17238484	HMGCR	5	G	T	0.04	0.02	0.78	-1.761	0.101	<1E-300	0.75	-2.006	0.198	1.4E-21
rs5909	HMGCR	5	G	A	0.00	0.59	0.91	-1.582	0.145	1.1E-27	0.90	-1.974	0.282	4.9E-13
rs2303152	HMGCR	5	G	A	0.13	0.72	0.90	-1.087	0.141	1.2E-14	0.88	-1.354	0.205	1.0E-09
rs10066707	HMGCR	5	G	A	1.68	0.03	0.63	-1.379	0.088	<1E-300	0.58	-1.590	0.173	3.0E-19
rs2006760	HMGCR	5	C	G	2.95	0.70	0.80	-1.182	0.107	4.0E-28	0.81	-1.706	0.243	1.7E-13
rs217386	NPC1L1	7	A	G	0.00	0.13	0.42	-0.794	0.085	1.4E-20	0.41	-1.125	0.118	1.2E-19
rs2073547*	NPC1L1	7	A	G	0.00	7.48E-13	0.82	-1.242	0.108	2.1E-30	0.81	-1.504	0.152	1.9E-21
rs7791240	NPC1L1	7	T	C	0.01	0.63	0.91	-0.861	0.146	4.1E-09	0.91	-1.318	0.202	1.8E-10
rs10234070	NPC1L1	7	C	T	0.48	0.76	0.88	-0.540	0.131	3.8E-05	0.90	-0.915	0.183	1.5E-06
rs2300414	NPC1L1	7	G	A	0.30	0.84	0.93	-0.487	0.169	4.0E-03	0.93	-1.094	0.248	5.5E-06
rs6511720	LDLR	19	T	G	0.00	0.51	0.12	-5.945	0.130	<1E-300	0.11	-6.766	0.136	<1E-300
rs1122608	LDLR	19	T	G	0.00	0.74	0.25	-1.819	0.097	<1E-300	0.23	-2.118	0.108	2.0E-86
rs688	LDLR	19	C	T	0.11	0.12	0.55	-1.100	0.085	2.7E-38	0.56	-1.728	0.118	3.0E-48

UKB: UK Biobank; **GLGC:** Global Lipids Genetics Consortium; **EAF:** Effect allele frequency; and **P_HWE:** P-value for Hardy-Weinberg Equilibrium test

^ Estimates for the UKB were taken from models adjusted for age, sex, assessment centre, type of genotyping array, and 40 principal components; GLGC estimates were adjusted for age, sex and principal components.
* rs2073547 excluded from the NPC1L1 genetic risk score because it deviated from the Hardy-Weinberg equilibrium (P_HWE < 0.05).

Supplementary Table 2: Construction of LDL-C lowering genetic risk scores.

	No of SNPs	SNPs	% variation in LDL-C in UKB	Association of each GRS with LDL-C		F-statistic
				**Beta (95% CI)**	**P**	
**PCSK9 genetic risk score**	7	rs11206510<br>rs2479409<br>rs2149041<br>rs2479394<br>rs10888897<br>rs7552841<br>rs562556	0.12%	-0.01 (-0.01, -0.01)	8.2×10^{-89}	400.03
**HMGCR genetic risk score**	6	rs12916<br>rs17238484<br>rs5909<br>rs2303152<br>rs10066707<br>rs2006760	0.15%	-0.01 (-0.01, -0.01)	1.2×10^{-105}	481.80
**NPC1L1 genetic risk score**	4	rs217386<br>rs7791240<br>rs10234070<br>rs2300414	0.026%	-0.01 (-0.01, -0.01)	1.8×10^{-20}	84.42
**LDLR genetic risk score**	3	rs6511720<br>rs1122608<br>rs688	0.64%	-0.06 (-0.06, -0.06)	$< 1 \times 10^{-300}$	2077.99

Supplementary Table 3: Disease categories and the number of cases with a phecode.

		Number of cases with phecodes *			
**Disease categories**	**No of phecodes**	**Minimum**	**Median**	**Mean**	**Maximum**
Circulatory system	116	209	1509	5075	75408
Congenital anomalies	26	204	428	836	3002
Dermatologic	58	205	1192	2075	7950
Digestive	132	208	1826	4980	45454
Endocrine/metabolic	77	200	631	3436	35143
Genitourinary	123	200	1393	2913	17536
Hematopoietic	28	200	716	1888	12234
Infectious diseases	28	207	1069	2157	11864
Injuries & poisonings	70	211	1403	2832	18479
Mental disorders	41	248	837	4115	39468
Musculoskeletal	87	204	1419	4806	50335
Neoplasms	100	201	1404	3344	30162
Neurological	50	210	514	1352	11404
Pregnancy complications	28	217	1036	1317	4525
Respiratory	59	203	1762	2945	24896
Sense organs	74	209	760	1694	22347
Symptoms	22	212	2140	5270	37426
Miscellaneous	16	200	1293	4502	16572

**Total phecodes**	**1135**

* Number of phecode cases was the same across all genetic risk scores.

Supplementary Table 4: List of 1135 phecodes tested in the PheWAS analyses.

Phecode	Description	Group	Cases	Controls
008	Intestinal infection	infectious diseases	10361	304892
008.5	Bacterial enteritis	infectious diseases	2546	304892
008.52	Intestinal infection due to C. difficile	infectious diseases	673	304892
008.6	Viral Enteritis	infectious diseases	903	304892
038	Septicemia	infectious diseases	4885	304892
038.1	Gram negative septicemia	infectious diseases	962	304892
038.2	Gram positive septicemia	infectious diseases	506	304892
041	Bacterial infection NOS	infectious diseases	11864	304892
041.1	Staphylococcus infections	infectious diseases	3057	304892
041.11	Methicillin sensitive Staphylococcus aureus	infectious diseases	2191	304892
041.2	Streptococcus infection	infectious diseases	1659	304892
041.4	E. coli	infectious diseases	2977	304892
053	Herpes zoster	infectious diseases	426	304892
054	Herpes simplex	infectious diseases	207	304892
070	Viral hepatitis	infectious diseases	580	304892
070.3	Viral hepatitis C	infectious diseases	237	304892
070.4	Chronic hepatitis	infectious diseases	261	304892
070.9	Hepatitis NOS	infectious diseases	476	304892
078	Viral warts & HPV	infectious diseases	1176	304892
079	Viral infection	infectious diseases	2704	304892
079.9	Viremia, NOS	infectious diseases	1577	304892
080	Postoperative infection	infectious diseases	4201	304892
081	Infection/inflammation of internal prosthetic device; implant; & graft	infectious diseases	2438	304892
1000	Burns	miscellaneous	511	336964
1001	Foreign body injury	miscellaneous	1110	336365
1002	Symptoms concerning nutrition, metabolism, & development	miscellaneous	5846	331629
1004	Other signs & symptoms involving emotional state	miscellaneous	444	337031
1005	Other symptoms	miscellaneous	1476	335999
1006	Crushing injury	miscellaneous	200	337275
1007	Injury to blood vessels	miscellaneous	281	337194
1008	Crushing or internal injury to organs	miscellaneous	1051	336424
1009	Injury, NOS	miscellaneous	7326	330149
1010	Other tests	miscellaneous	16572	320903
1011	Complications of surgical & medical procedures	miscellaneous	10083	327392
1013	Asphyxia & hypoxemia	miscellaneous	349	337126
1014	Effects of heat, cold & air pressure	miscellaneous	254	337221
1015	Effects of other external causes	miscellaneous	10460	327015
1019	Other ill-defined & unknown causes of morbidity & mortality	miscellaneous	13786	323689
110	Dermatophytosis / Dermatomycosis	infectious diseases	294	304892
110.1	Dermatophytosis	infectious diseases	288	304892
1100	Family history of colonic polyps	miscellaneous	2277	335198
112	Candidiasis	infectious diseases	2400	304892
117	Mycoses	infectious diseases	307	304892
136	Other infectious & parasitic diseases	infectious diseases	237	304892
145	Cancer of mouth	neoplasms	627	234242
145.2	Cancer of tongue	neoplasms	303	234242
149	Cancer of larynx, pharynx, nasal cavities	neoplasms	630	234242
149.1	Cancer of oropharynx	neoplasms	257	234242
149.4	Cancer of larynx	neoplasms	252	234242
150	Cancer of esophagus	neoplasms	817	234242
151	Cancer of stomach	neoplasms	603	234242
153	Colorectal cancer	neoplasms	4541	234242
153.2	Colon cancer	neoplasms	3053	234242
153.3	Malignant neoplasm of rectum, rectosigmoid junction, & anus	neoplasms	2169	234242
155	Cancer of liver & intrahepatic bile duct	neoplasms	449	234242
157	Pancreatic cancer	neoplasms	756	234242
158	Neoplasm of unspecified nature of digestive system	neoplasms	1091	234242
159	Malignant neoplasm of other & ill-defined sites within the digestive organs & peritoneum	neoplasms	5580	234242
159.2	Malignant neoplasm of small intestine, including duodenum	neoplasms	204	234242
159.3	Malignant neoplasm of gallbladder & extrahepatic bile ducts	neoplasms	233	234242
159.4	Malignant neoplasm of retroperitoneum & peritoneum	neoplasms	205	234242
165	Cancer within the respiratory system	neoplasms	3012	234242
165.1	Cancer of bronchus; lung	neoplasms	2617	234242
170	Cancer of bone & connective tissue	neoplasms	443	234242
170.2	Cancer of connective tissue	neoplasms	320	234242
172	Skin cancer	neoplasms	14207	234242
172.1	Melanomas of skin, dx or hx	neoplasms	2739	234242
172.11	Melanomas of skin	neoplasms	2739	234242
172.2	Other non-epithelial cancer of skin	neoplasms	11594	234242
172.3	Carcinoma in situ of skin	neoplasms	741	234242
174	Breast cancer	neoplasms	12023	232710
174.1	Breast cancer [female]	neoplasms	11848	120733
174.11	Malignant neoplasm of female breast	neoplasms	11108	120733
175	Acquired absence of breast	neoplasms	3261	120184
180	Cervical cancer & dysplasia	neoplasms	3299	122212

Phecode	Description	Group	Cases	Controls
507	Pleurisy; pleural effusion	respiratory	6979	269389
508	Pulmonary collapse; interstitial & compensatory emphysema	respiratory	2447	269389
509	Respiratory failure, insufficiency, arrest	respiratory	3054	269389
509.1	Respiratory failure	respiratory	2479	269389
509.2	Respiratory insufficiency	respiratory	2205	269389
509.8	Dependence on respirator [Ventilator] or supplemental oxygen	respiratory	444	269389
510	Other diseases of lung	respiratory	1171	269389
512	Other symptoms of respiratory system	respiratory	9349	269389
512.1	Wheezing	respiratory	268	269389
512.2	Painful respiration	respiratory	203	269389
512.7	Shortness of breath	respiratory	6109	269389
512.8	Cough	respiratory	2986	269389
512.9	Other dyspnea	respiratory	884	269389
513	Respiratory abnormalities	respiratory	603	269389
513.4	Hyperventilation	respiratory	251	269389
514	Abnormal findings examination of lungs	respiratory	2607	269389
516	Abnormal sputum	respiratory	2036	269389
516.1	Hemoptysis	respiratory	1948	269389
519	Other diseases of respiratory system, not elsewhere classified	respiratory	9890	269389
519.2	Respiratory complications	respiratory	323	269389
519.8	Other diseases of respiratory system, NEC	respiratory	9307	269389
519.9	Symptoms involving respiratory system & other chest symptoms	respiratory	272	269389
520	Disorders of tooth development	digestive	2122	191014
520.2	Disturbances in tooth eruption	digestive	2041	191014
521	Diseases of hard tissues of teeth	digestive	3012	191014
521.1	Dental caries	digestive	2952	191014
522	Diseases of pulp & periapical tissues	digestive	1661	191014
522.5	Periapical abscess	digestive	1117	191014
523	Gingival & periodontal diseases	digestive	1638	191014
523.3	Periodontitis (acute or chronic)	digestive	1133	191014
523.32	Chronic periodontitis	digestive	1000	191014
524	Dentofacial anomalies, including malocclusion	digestive	309	191014
524.3	Anomalies of tooth position/malocclusion	digestive	276	191014
525	Other diseases of the teeth & supporting structures	digestive	2546	191014
526	Diseases of the jaws	digestive	909	191014
526.1	Cysts of the jaws	digestive	312	191014
526.4	Temporomandibular joint disorders	digestive	229	191014
526.41	Temporomandibular joint disorder, unspecified	digestive	222	191014
526.9	Jaw disease NOS	digestive	220	191014
527	Diseases of the salivary glands	digestive	663	191014
527.2	Sialoadenitis	digestive	340	191014
528	Diseases of the oral soft tissues, excluding lesions specific for gingiva & tongue	digestive	3788	191014
528.1	Stomatitis & mucositis	digestive	475	191014
528.11	Stomatitis & mucositis (ulcerative)	digestive	406	191014
528.5	Diseases of lips	digestive	644	191014
528.6	Leukoplakia of oral mucosa	digestive	298	191014
528.7	Sialolithiasis	digestive	289	191014
529	Diseases & other conditions of the tongue	digestive	1210	191014
529.1	Glossitis	digestive	290	191014
530	Diseases of esophagus	digestive	34889	191014
530.1	Esophagitis, GERD, & related diseases	digestive	32569	191014
530.11	GERD	digestive	15750	191014
530.12	Ulcer of esophagus	digestive	4731	191014
530.13	Barrett's esophagus	digestive	3105	191014
530.14	Reflux esophagitis	digestive	10069	191014
530.2	Esophageal bleeding (varices/hemorrhage)	digestive	1770	191014
530.3	Stricture & stenosis of esophagus	digestive	1940	191014
530.5	Disorders of esophageal motility	digestive	637	191014
530.7	Gastroesophageal laceration-hemorrhage syndrome	digestive	303	191014
530.9	Heartburn	digestive	2010	191014
531	Peptic ulcer (excl. esophageal)	digestive	7143	191014
531.1	Hemorrhage from gastrointestinal ulcer	digestive	638	191014
531.2	Gastric ulcer	digestive	4026	191014
531.3	Duodenal ulcer	digestive	2794	191014
531.4	Peptic ulcer, site unspecified	digestive	404	191014
532	Dysphagia	digestive	6669	191014
535	Gastritis & duodenitis	digestive	28094	191014
535.1	Acute gastritis	digestive	1145	191014
535.2	Atrophic gastritis	digestive	372	191014
535.6	Duodenitis	digestive	7254	191014
535.8	Other specified gastritis	digestive	8264	191014
536	Disorders of function of stomach	digestive	11052	191014
536.8	Dyspepsia & other specified disorders of function of stomach	digestive	11046	191014
537	Other disorders of stomach & duodenum	digestive	3459	191014
540	Appendiceal conditions	digestive	3462	191014

Phecode	Description	Group	Cases	Controls
180.1	Cervical cancer	neoplasms	1622	122212
180.3	Cervical intraepithelial neoplasia [CIN] [Cervical dysplasia]	neoplasms	1757	122212
182	Malignant neoplasm of uterus	neoplasms	1240	122251
184	Cancer of other female genital organs	neoplasms	2388	122251
184.1	Malignant neoplasm of ovary & other uterine adnexa	neoplasms	2091	122251
184.11	Malignant neoplasm of ovary	neoplasms	2072	122251
184.2	Cancer of other female genital organs (excluding uterus & ovary)	neoplasms	281	122251
185	Cancer of prostate	neoplasms	7072	105218
187	Cancer of other male genital organs	neoplasms	3330	111991
187.1	Malignant neoplasm of unspecified male genital organ	neoplasms	3085	111991
187.2	Malignant neoplasm of testis	neoplasms	3194	111991
189	Cancer of urinary organs (incl. kidney & bladder)	neoplasms	4104	234242
189.1	Cancer of kidney & renal pelvis	neoplasms	1098	234242
189.11	Malignant neoplasm of kidney, except pelvis	neoplasms	1055	234242
189.2	Cancer of bladder	neoplasms	2339	234242
189.21	Malignant neoplasm of bladder	neoplasms	2083	234242
191	Manlignant & unknown neoplasms of brain & nervous system	neoplasms	702	234242
191.1	Cancer of brain & nervous system	neoplasms	579	234242
191.11	Cancer of brain	neoplasms	539	234242
193	Thyroid cancer	neoplasms	345	234242
195	Cancer, suspected or other	neoplasms	17637	234242
195.1	Malignant neoplasm, other	neoplasms	16966	234242
196	Radiotherapy	neoplasms	12225	234242
197	Chemotherapy	neoplasms	30162	234242
198	Secondary malignant neoplasm	neoplasms	10103	234242
198.1	Secondary malignancy of lymph nodes	neoplasms	5504	234242
198.2	Secondary malignancy of respiratory organs	neoplasms	2544	234242
198.3	Secondary malignant neoplasm of digestive systems	neoplasms	1757	234242
198.4	Secondary malignant neoplasm of liver	neoplasms	2970	234242
198.5	Secondary malignancy of brain/spine	neoplasms	927	234242
198.6	Secondary malignancy of bone	neoplasms	2454	234242
198.7	Secondary malignant neoplasm of skin	neoplasms	201	234242
199	Neoplasm of uncertain behavior	neoplasms	1504	234242
200	Myeloproliferative disease	neoplasms	1054	234242
200.1	Polycythemia vera	neoplasms	408	232957
201	Hodgkin's disease	neoplasms	252	234242
202	Cancer of other lymphoid, histiocytic tissue	neoplasms	2358	234242
202.2	Non-Hodgkins lymphoma	neoplasms	1853	234242
202.21	Nodular lymphoma	neoplasms	358	234242
202.24	Large cell lymphoma	neoplasms	623	234242
204	Leukemia	neoplasms	1774	234242
204.1	Lymphoid leukemia	neoplasms	612	234242
204.12	Lymphoid leukemia, chronic	neoplasms	540	234242
204.2	Myeloid leukemia	neoplasms	507	234242
204.21	Myeloid leukemia, acute	neoplasms	353	234242
204.4	Multiple myeloma	neoplasms	593	234242
208	Benign neoplasm of colon	neoplasms	20688	234242
210	Benign neoplasm of lip, oral cavity, & pharynx	neoplasms	911	234242
211	Benign neoplasm of other parts of digestive system	neoplasms	5805	234242
212	Benign neoplasm of respiratory & intrathoracic organs	neoplasms	341	234242
213	Benign neoplasm of bone & articular cartilage	neoplasms	284	234242
214	Lipoma	neoplasms	5942	234242
214.1	Lipoma of skin & subcutaneous tissue	neoplasms	4303	234242
215	Other benign neoplasm of connective & other soft tissue	neoplasms	1015	234242
216	Benign neoplasm of skin	neoplasms	8174	234242
217	Vascular hamartomas & non-neoplastic nevi	neoplasms	592	234242
217.1	Nevus, non-neoplastic	neoplasms	548	234242
218	Benign neoplasm of uterus	neoplasms	9233	122251
218.1	Uterine leiomyoma	neoplasms	9007	122251
218.2	Other benign neoplasm of uterus	neoplasms	304	122251
220	Benign neoplasm of ovary	neoplasms	1320	122251
225	Benign neoplasm of brain & other parts of nervous system	neoplasms	818	234242
225.1	Benign neoplasm of brain, cranial nerves, meninges	neoplasms	764	234242
226	Benign neoplasm of thyroid glands	neoplasms	260	234242
227	Benign neoplasm of other endocrine glands & related structures	neoplasms	895	234242
227.2	Benign neoplasm of parathyroid gland	neoplasms	377	234242
227.3	Benign neoplasm of pituitary gland & craniopharyngeal duct (pouch)	neoplasms	319	234242
228	Hemangioma & lymphangioma, any site	neoplasms	1487	234242
229	Benign neoplasm of unspecified sites	neoplasms	2113	234242
240	Simple & unspecified goiter	endocrine/metabolic	542	261466
241	Nontoxic nodular goiter	endocrine/metabolic	1539	261466
241.1	Nontoxic uninodular goiter	endocrine/metabolic	478	261466
241.2	Nontoxic multinodular goiter	endocrine/metabolic	631	261466
242	Thyrotoxicosis with or without goiter	endocrine/metabolic	1795	261466
242.1	Graves' disease	endocrine/metabolic	439	261466
242.3	Exophthalmos	endocrine/metabolic	210	261466

Phecode	Description	Group	Cases	Controls
540.1	Appendicitis	digestive	3299	191014
540.11	Acute appendicitis	digestive	2801	191014
550	Abdominal hernia	digestive	45454	191014
550.1	Inguinal hernia	digestive	15079	191014
550.2	Diaphragmatic hernia	digestive	26753	191014
550.3	Femoral hernia	digestive	634	191014
550.4	Umbilical hernia	digestive	3634	191014
550.5	Ventral hernia	digestive	3384	191014
550.6	Incisional hernia	digestive	246	191014
555	Inflammatory bowel disease & other gastroenteritis & colitis	digestive	4149	191014
555.1	Regional enteritis	digestive	1621	191014
555.2	Ulcerative colitis	digestive	2920	191014
555.21	Ulcerative colitis (chronic)	digestive	541	191014
556	Ulceration of the lower GI tract	digestive	1084	191014
556.1	Ulceration of intestine	digestive	711	191014
557	Intestinal malabsorption (non-celiac)	digestive	287	191014
557.1	Celiac disease	digestive	1764	191014
558	Noninfectious gastroenteritis	digestive	13696	191014
559	Ileostomy status	digestive	1579	191014
560	Intestinal obstruction without mention of hernia	digestive	4175	191014
560.1	Paralytic ileus	digestive	602	191014
560.2	Impaction of intestine	digestive	282	191014
560.3	Peritoneal or intestinal adhesions	digestive	874	191014
560.4	Other intestinal obstruction	digestive	3467	191014
561	Symptoms involving digestive system	digestive	16127	191014
561.1	Diarrhea	digestive	536	191014
561.2	Flatulence	digestive	1767	191014
562	Diverticulosis & diverticulitis	digestive	28363	191014
562.1	Diverticulosis	digestive	28326	191014
563			10878	191014
564	Functional digestive disorders	digestive	22341	191014
564.1	Irritable Bowel Syndrome	digestive	5580	191014
564.8			1743	191014
564.9	Personal history of diseases of digestive system	digestive	15641	191014
565	Anal & rectal conditions	digestive	14352	191014
565.1	Anal & rectal polyp	digestive	7409	191014
567	Peritonitis & retroperitoneal infections	digestive	949	191014
568	Other disorders of peritoneum	digestive	3567	191014
568.1	Peritoneal adhesions (postoperative) (postinfection)	digestive	3346	191014
569	Other disorders of intestine	digestive	4313	191014
569.1	Toxic gastroenteritis & colitis	digestive	208	191014
569.2	Gastrointestinal complications	digestive	515	191014
571	Chronic liver disease & cirrhosis	digestive	4206	191014
571.5	Other chronic nonalcoholic liver disease	digestive	3329	191014
571.51	Cirrhosis of liver without mention of alcohol	digestive	749	191014
571.6	Primary biliary cirrhosis	digestive	258	191014
571.8	Liver abscess & sequelae of chronic liver disease	digestive	1140	191014
571.81	Portal hypertension	digestive	589	191014
572	Ascites (nonmalignant)	digestive	1656	191014
573	Other disorders of liver	digestive	1712	191014
573.3	Hepatomegaly	digestive	295	191014
573.5	Jaundice (not of newborn)	digestive	914	191014
573.7	Abnormal results of function study of liver	digestive	3714	191014
573.9	Abnormal serum enzyme levels	digestive	214	191014
574	Cholelithiasis & cholecystitis	digestive	15179	191014
574.1	Cholelithiasis	digestive	12924	191014
574.11	Cholelithiasis with acute cholecystitis	digestive	1537	191014
574.12	Cholelithiasis with other cholecystitis	digestive	5200	191014
574.2	Calculus of bile duct	digestive	2550	191014
574.3	Cholecystitis without cholelithiasis	digestive	2574	191014
575	Other biliary tract disease	digestive	3989	191014
575.1	Cholangitis	digestive	599	191014
575.2	Obstruction of bile duct	digestive	850	191014
575.6	Cholesterolosis of gallbladder	digestive	437	191014
575.7	Other disorders of gallbladder	digestive	1372	191014
575.8	Other disorders of biliary tract	digestive	1096	191014
575.9	Nonspecific abnormal findings on radiological & other examination of biliary tract	digestive	360	191014
577	Diseases of pancreas	digestive	2747	191014
577.1	Acute pancreatitis	digestive	1925	191014
577.2	Chronic pancreatitis	digestive	473	191014
577.3	Cyst & pseudocyst of pancreas	digestive	388	191014
578	Gastrointestinal hemorrhage	digestive	19880	191014
578.1	Hematemesis	digestive	1882	191014
578.2	Blood in stool	digestive	2514	191014
578.8	Hemorrhage of rectum & anus	digestive	11473	191014
578.9	Hemorrhage of gastrointestinal tract	digestive	6068	191014

Phecode	Description	Group	Cases	Controls
244	Hypothyroidism	endocrine/metabolic	14603	261466
244.1	Secondary hypothyroidism	endocrine/metabolic	1144	261466
244.4	Hypothyroidism NOS	endocrine/metabolic	13923	261466
245	Thyroiditis	endocrine/metabolic	282	261466
245.2	Chronic thyroiditis	endocrine/metabolic	215	261466
245.21	Chronic lymphocytic thyroiditis	endocrine/metabolic	202	261466
246	Other disorders of thyroid	endocrine/metabolic	386	261466
250	Diabetes mellitus	endocrine/metabolic	19441	261466
250.1	Type 1 diabetes	endocrine/metabolic	2582	261466
250.11	Type 1 diabetes with ketoacidosis	endocrine/metabolic	212	261466
250.13	Type 1 diabetes with ophthalmic manifestations	endocrine/metabolic	451	261466
250.2	Type 2 diabetes	endocrine/metabolic	18758	261466
250.22	Type 2 diabetes with renal manifestations	endocrine/metabolic	213	261466
250.23	Type 2 diabetes with ophthalmic manifestations	endocrine/metabolic	1355	261466
250.24	Type 2 diabetes with neurological manifestations	endocrine/metabolic	598	261466
250.4	Abnormal glucose	endocrine/metabolic	678	261466
250.41	Impaired fasting glucose	endocrine/metabolic	288	261466
250.42	Other abnormal glucose	endocrine/metabolic	391	261466
250.6	Polyneuropathy in diabetes	endocrine/metabolic	408	261466
250.7	Diabetic retinopathy	endocrine/metabolic	1398	254478
251.1	Hypoglycemia	endocrine/metabolic	1024	261466
252	Disorders of parathyroid gland	endocrine/metabolic	940	261466
252.1	Hyperparathyroidism	endocrine/metabolic	830	261466
253	Disorders of the pituitary gland & its hypothalamic control	endocrine/metabolic	697	261466
253.2	Pituitary hypofunction	endocrine/metabolic	261	261466
255	Disorders of adrenal glands	endocrine/metabolic	636	261466
255.2	Adrenal hypofunction	endocrine/metabolic	363	261466
255.21	Glucocorticoid deficiency	endocrine/metabolic	363	261466
256	Ovarian dysfunction	endocrine/metabolic	241	143689
256.4	Polycystic ovaries	endocrine/metabolic	200	143689
260	Protein-calorie malnutrition	endocrine/metabolic	275	261466
260.6	Anorexia	endocrine/metabolic	885	261466
261	Vitamin deficiency	endocrine/metabolic	1684	261466
261.2	Vitamin B-complex deficiencies	endocrine/metabolic	911	261466
261.4	Vitamin D deficiency	endocrine/metabolic	788	261466
262	Mineral deficiency NEC	endocrine/metabolic	298	261466
269	Proteinuria	endocrine/metabolic	324	261466
270	Disorders of protein plasma/amino-acid transport & metabolism	endocrine/metabolic	365	261466
270.3	Disorders of plasma protein metabolism	endocrine/metabolic	321	261466
270.32	Paraproteinemia	endocrine/metabolic	246	261466
272	Disorders of lipoid metabolism	endocrine/metabolic	35143	261466
272.1	Hyperlipidemia	endocrine/metabolic	35033	261466
272.11	Hypercholesterolemia	endocrine/metabolic	32554	261466
272.9	Unspecified disorder of lipoid metabolism	endocrine/metabolic	251	261466
274	Gout & other crystal arthropathies	endocrine/metabolic	4155	261466
274.1	Gout	endocrine/metabolic	3587	261466
274.2	Crystal arthropathies	endocrine/metabolic	628	261466
274.21	Chondrocalcinosis	endocrine/metabolic	557	261466
275	Disorders of mineral metabolism	endocrine/metabolic	2470	261466
275.1	Disorders of iron metabolism	hematopoietic	661	309446
275.3	Disorders of magnesium metabolism	endocrine/metabolic	543	261466
275.5	Disorders of calcium/phosphorus metabolism	endocrine/metabolic	1454	261466
275.53	Disorders of phosphorus metabolism	endocrine/metabolic	273	261466
276	Disorders of fluid, electrolyte, & acid-base balance	endocrine/metabolic	7916	261466
276.1	Electrolyte imbalance	endocrine/metabolic	5081	261466
276.11	Hyperosmolality &/or hypernatremia	endocrine/metabolic	323	261466
276.12	Hypoosmolality &/or hyponatremia	endocrine/metabolic	2295	261466
276.13	Hyperpotassemia	endocrine/metabolic	1176	261466
276.14	Hypopotassemia	endocrine/metabolic	1781	261466
276.4	Acid-base balance disorder	endocrine/metabolic	1421	261466
276.41	Acidosis	endocrine/metabolic	1278	261466
276.5	Hypovolemia	endocrine/metabolic	3349	261466
276.6	Fluid overload	endocrine/metabolic	615	261466
277	Other disorders of metabolism	endocrine/metabolic	1034	261466
277.4	Disorders of bilirubin excretion	endocrine/metabolic	383	261466
277.5	Other disorders of lipoid metabolism	endocrine/metabolic	256	261466
278	Overweight, obesity & other hyperalimentation	endocrine/metabolic	12786	261466
278.1	Obesity	endocrine/metabolic	12646	261466
279	Disorders involving the immune mechanism	endocrine/metabolic	271	261466
279.1	Immunity deficiency	endocrine/metabolic	223	261466
279.7	Other immunological findings	endocrine/metabolic	239	261466
280	Iron deficiency anemias	hematopoietic	8403	310388
280.1	Iron deficiency anemias, unspecified or not due to blood loss	hematopoietic	8022	310388
280.2	Iron deficiency anemia secondary to blood loss (chronic)	hematopoietic	436	310388
281	Other deficiency anemia	hematopoietic	1150	310388
281.1	Megaloblastic anemia	hematopoietic	1093	310388
281.11	Pernicious anemia	hematopoietic	769	310388
281.13	Folate-deficiency anemia	hematopoietic	200	310388
284	Aplastic anemia	hematopoietic	480	310388
579	Other symptoms involving abdomen & pelvis	digestive	1543	191014
579.2	Splenomegaly	digestive	447	191014
579.8	Nonspecific abnormal findings in stool contents	digestive	1887	191014
580	Nephritis; nephrosis; renal sclerosis	genitourinary	1984	234338
580.1	Glomerulonephritis	genitourinary	906	234338
580.14	Chronic glomerulonephritis, NOS	genitourinary	723	234338
580.2	Nephrotic syndrome without mention of glomerulonephritis	genitourinary	1042	234338
580.3	Nephritis & nephropathy without mention of glomerulonephritis	genitourinary	301	234338
580.32	Nephritis & nephropathy with pathological lesion	genitourinary	286	234338
585	Renal failure	genitourinary	10995	234338
585.1	Acute renal failure	genitourinary	6203	234338
585.2	Renal failure NOS	genitourinary	1430	234338
585.3	Chronic renal failure [CKD]	genitourinary	5710	234338
585.31	Renal dialysis	genitourinary	507	234338
585.32	End stage renal disease	genitourinary	426	234338
585.33	Chronic Kidney Disease, Stage III	genitourinary	3054	234338
585.34	Chronic Kidney Disease, Stage IV	genitourinary	455	234338
585.4	Chronic kidney disease, Stage I or II	genitourinary	343	234338
586	Other disorders of the kidney & ureters	genitourinary	3536	234338
586.1	Anatomical abnormatilies of kidney & ureters	genitourinary	232	234338
586.11	Small kidney	genitourinary	200	234338
586.2	Cyst of kidney, acquired	genitourinary	1393	234338
586.4	Stricture/obstruction of ureter	genitourinary	936	234338
587	Kidney replaced by transpant	genitourinary	334	234338
590	Pyelonephritis	genitourinary	1361	234338
591	Urinary tract infection	genitourinary	12543	234338
592	Cystitis & urethritis	genitourinary	2885	234338
592.1	Cystitis	genitourinary	2766	234338
592.12	Chronic cystitis	genitourinary	833	234338
592.13	Chronic interstitial cystitis	genitourinary	233	234338
593	Hematuria	genitourinary	14542	234338
594	Urinary calculus	genitourinary	6165	234338
594.1	Calculus of kidney	genitourinary	3198	234338
594.2	Calculus of lower urinary tract	genitourinary	767	234338
594.3	Calculus of ureter	genitourinary	2243	234338
594.8	Renal colic	genitourinary	1754	234338
595	Hydronephrosis	genitourinary	2075	234338
596	Other disorders of bladder	genitourinary	9275	234338
596.1	Bladder neck obstruction	genitourinary	1831	234338
596.5	Functional disorders of bladder	genitourinary	1431	234338
597	Other disorders of urethra & urinary tract	genitourinary	3888	234338
597.1	Urethral stricture (not specified as infectious)	genitourinary	3159	234338
598	Abnormal findings on examination of urine	genitourinary	3286	234338
598.9	Other nonspecific findings on examination of urine	genitourinary	299	234338
599	Other symptoms/disorders or the urinary system	genitourinary	17536	234338
599.2	Retention of urine	genitourinary	7478	234338
599.3	Dysuria	genitourinary	1104	234338
599.4	Urinary incontinence	genitourinary	8031	234338
599.5	Frequency of urination & polyuria	genitourinary	3626	234338
599.9	Other abnormality of urination	genitourinary	1683	234338
600	Hyperplasia of prostate	genitourinary	11447	114193
601	Inflammatory diseases of prostate	genitourinary	2119	114193
601.1	Prostatitis	genitourinary	1527	114193
601.11	Acute prostatitis	genitourinary	231	114193
601.12	Chronic prostatitis	genitourinary	908	114193
601.4	Balanoposthitis	genitourinary	283	114193
601.8	Other inflammatory disorders of male genital organs	genitourinary	291	114193
602	Other disorders of prostate	genitourinary	1292	114193
603	Other disorders of testis	genitourinary	2075	117407
603.1	Hydrocele	genitourinary	1274	117407
604	Disorders of penis	genitourinary	3185	117407
604.1	Redundant prepuce & phimosis/BXO	genitourinary	2635	117407
604.3	Peyronie's disease	genitourinary	752	117407
605	Erectile dysfunction [ED]	genitourinary	482	117407
608	Other disorders of male genital organs	genitourinary	2620	117407
610	Benign mammary dysplasias	genitourinary	2479	116931
610.1	Cystic mastopathy	genitourinary	805	116931
610.2	Fibro adenosis of breast	genitourinary	275	116931
610.3	Fibrosclerosis of breast	genitourinary	266	116931
610.4	Benign neoplasm of breast	genitourinary	1353	116931
610.8	Other specified benign mammary dysplasias	genitourinary	644	116931
611	Abnormal findings on mammogram or breast exam	genitourinary	1363	234338
611.3	Lump or mass in breast	genitourinary	1305	234338
612	Breast conditions, congenital or relating to hormones	genitourinary	788	234338
612.2	Hypertrophy of breast (Gynecomastia)	genitourinary	715	234338
613	Other nonmalignant breast conditions	genitourinary	1712	234338
613.1	Inflammatory disease of breast	genitourinary	615	234338
613.7	Other signs & symptoms in breast	genitourinary	614	234338
613.8	Other specified disorders of breast	genitourinary	289	234338

Phecode	Description	Group	Cases	Controls
285	Other anemias	hematopoietic	12234	310388
285.1	Acute post hemorrhagic anemia	hematopoietic	237	310388
285.2	Anemia of chronic disease	hematopoietic	736	310388
285.22	Anemia in neoplastic disease	hematopoietic	433	310388
286	Coagulation defects	hematopoietic	1023	310388
286.1	Congenital coagulation defects	hematopoietic	442	310388
286.12	Congenital deficiency of other clotting factors (including factor VII)	hematopoietic	278	310388
286.7	Other & unspecified coagulation defects	hematopoietic	411	310388
287	Purpura & other hemorrhagic conditions	hematopoietic	1804	310388
287.3	Thrombocytopenia	hematopoietic	1667	310388
287.31	Primary thrombocytopenia	hematopoietic	415	310388
288	Diseases of white blood cells	hematopoietic	697	310388
288.1	Decreased white blood cell count	hematopoietic	3267	310388
288.11	Neutropenia	hematopoietic	3267	310388
289	Other diseases of blood & blood-forming organs	hematopoietic	954	310388
289.3	Personal history of diseases of blood & blood-forming organs	hematopoietic	318	310388
289.4	Lymphadenitis	hematopoietic	2636	310388
289.5	Diseases of spleen	hematopoietic	514	310388
289.8	Polycythemia, secondary	hematopoietic	315	310063
290	Delirium dementia & amnestic & other cognitive disorders	mental disorders	2856	262912
290.1	Dementias	mental disorders	1583	262912
290.11	Alzheimer's disease	mental disorders	666	262912
290.13	Senile dementia	mental disorders	847	262912
290.16	Vascular dementia	mental disorders	361	262912
290.2	Delirium due to conditions classified elsewhere	mental disorders	1157	262912
291	Other specified nonpsychotic and/or transient mental disorders	mental disorders	693	262912
291.8	Alteration of consciousness	mental disorders	484	262912
292	Neurological disorders	mental disorders	5218	262912
292.1	Aphasia/speech disturbance	mental disorders	1626	262912
292.2	Mild cognitive impairment	mental disorders	248	262912
292.3	Memory loss	mental disorders	730	262912
292.4	Altered mental status	mental disorders	2527	262912
292.6	Hallucinations	mental disorders	350	262912
293	Symptoms involving head & neck	mental disorders	1929	262912
293.1	Swelling, mass, or lump in head & neck [Space-occupying lesion, intracranial NOS]	mental disorders	832	262912
295	Schizophrenia & other psychotic disorders	mental disorders	793	262912
295.1	Schizophrenia	mental disorders	513	262912
296	Mood disorders	mental disorders	13263	262912
296.1	Bipolar	mental disorders	1015	262912
296.2	Depression	mental disorders	12701	262912
296.22	Major depressive disorder	mental disorders	12629	262912
297	Suicidal ideation or attempt	mental disorders	1529	262912
297.2	Suicide or self-inflicted injury	mental disorders	1457	262912
300	Anxiety disorders	mental disorders	8295	262912
300.1	Anxiety disorder	mental disorders	7701	262912
300.12	Agoraphobia, social phobia, & panic disorder	mental disorders	820	262912
300.13	Phobia	mental disorders	649	262912
301	Personality disorders	mental disorders	338	262912
302	Sexual & gender identity disorders	mental disorders	279	262912
303	Psychogenic & somatoform disorders	mental disorders	457	262912
303.3	Psychogenic disorder	mental disorders	339	262912
304	Adjustment reaction	mental disorders	258	262912
306	Other mental disorder	mental disorders	39468	262912
306.9	Tension headache	mental disorders	280	262912
315	Developmental delays & disorders	mental disorders	563	262912
316	Substance addiction & disorders	mental disorders	412	262912
317	Alcohol-related disorders	mental disorders	12721	262912
317.1	Alcoholism	mental disorders	10886	262912
317.11	Alcoholic liver damage	mental disorders	837	262912
318	Tobacco use disorder	mental disorders	18403	262912
320	Meningitis	neurological	391	295570
323	Encephalitis	neurological	349	295570
324	Other CNS infection & poliomyelitis	neurological	305	295570
327	Sleep disorders	neurological	1210	295570
327.3	Sleep apnea	neurological	4435	295570

Phecode	Description	Group	Cases	Controls
614	Inflammatory diseases of female pelvic organs	genitourinary	5602	116931
614.1	Pelvic peritoneal adhesions, female (postoperative) (post infection)	genitourinary	2293	116931
614.3	Pelvic inflammatory disease (PID)	genitourinary	802	116931
614.32	Chronic inflammatory pelvic disease	genitourinary	352	116931
614.33	Pelvic inflammatory disease, NOS	genitourinary	308	116931
614.4	Inflammatory diseases of uterus, except cervix	genitourinary	245	116931
614.5	Inflammatory disease of cervix, vagina, & vulva	genitourinary	2642	116931
614.51	Cervicitis & endocervicitis	genitourinary	1116	116931
614.52	Vaginitis & vulvovaginitis	genitourinary	591	116931
614.53	Cyst or abscess of Bartholin's gland	genitourinary	658	116931
614.54	Abscess or ulceration of vulva	genitourinary	228	116931
615	Endometriosis	genitourinary	3495	116931
617	Disorders secondary to childbirth, surgery, trauma	genitourinary	1252	234338
618	Genital prolapse	genitourinary	10694	116931
618.1	Prolapse of vaginal walls	genitourinary	6724	116931
618.2	Uterine/Uterovaginal prolapse	genitourinary	4836	116931
618.5	Prolapse of vaginal vault after hysterectomy	genitourinary	459	116931
618.6	Vaginal enterocele, congenital or acquired	genitourinary	629	116931
619	Noninflammatory female genital disorders	genitourinary	8298	116931
619.1	Noninflammatory disorders of ovary, fallopian tube, & broad ligament	genitourinary	683	116931
619.2	Disorders of uterus, NEC	genitourinary	3020	116931
619.3	Noninflammatory disorders of cervix	genitourinary	2536	116931
619.4	Noninflammatory disorders of vagina	genitourinary	1535	116931
619.5	Noninflammatory disorders of vulva & perineum	genitourinary	1209	116931
621	Endometrial hyperplasia	genitourinary	970	116931
622	Polyp of female genital organs	genitourinary	9429	116931
622.1	Polyp of corpus uteri	genitourinary	6913	116931
622.2	Mucous polyp of cervix	genitourinary	2951	116931
623	Hypertrophy of female genital organs	genitourinary	1089	116931
624	Symptoms involving female genital tract	genitourinary	805	116931
624.1	Dystrophy of female genital tract	genitourinary	254	116931
624.2	Atrophy of female genital tract	genitourinary	440	116931
624.9	stress incontinence, female	genitourinary	5074	116931
625	Pain & other symptoms associated with female genital organs	genitourinary	2526	116931
625.1	Dyspareunia	genitourinary	1007	116931
626	Disorders of menstruation & other abnormal bleeding from female genital tract	genitourinary	15577	116931
626.1	Irregular menstrual cycle/bleeding	genitourinary	13278	116931
626.12	Excessive or frequent menstruation	genitourinary	8228	116931
626.13	Irregular menstrual cycle	genitourinary	1646	116931
626.14	Irregular menstrual bleeding	genitourinary	3244	116931
626.2	Dysmenorrhea	genitourinary	1487	116931
626.8	Infertility, female	genitourinary	1139	116931
627	Menopausal & postmenopausal disorders	genitourinary	9346	116931
627.1	Postmenopausal bleeding	genitourinary	7915	116931
627.3	Postmenopausal atrophic vaginitis	genitourinary	1078	116931
627.4	Premenopausal menorrhagia	genitourinary	252	116931
628	Ovarian cyst	genitourinary	4147	116931
634	Miscarriage; stillbirth	pregnancy complications	4525	169668
634.1	Missed abortion/Hydatidiform mole	pregnancy complications	1038	169668
634.3	Ectopic pregnancy	pregnancy complications	272	169668
635	Hemorrhage during pregnancy; childbirth & postpartum	pregnancy complications	1680	169668
635.2	Antepartum hemorrhage, abruptio placentae, & placenta previa	pregnancy complications	739	169668
635.3	Placenta previa & abruptio placenta	pregnancy complications	1054	169668
636	Early or threatened labor; hemorrhage in early pregnancy	pregnancy complications	2079	169668
636.2	Early onset of delivery	pregnancy complications	508	169668
636.3	Hemorrhage in early pregnancy	pregnancy complications	872	169668
642	Hypertension complicating pregnancy, childbirth, & the puerperium	pregnancy complications	946	169668
642.1	Preeclampsia & eclampsia	pregnancy complications	259	169668
644	Anemia during pregnancy	pregnancy complications	677	169668
645	Late pregnancy & failed induction	pregnancy complications	1054	325859
646	Other complications of pregnancy NEC	pregnancy complications	1868	169668
647.1	Infections of genitourinary tract during pregnancy	pregnancy complications	226	169668
650	Normal delivery	pregnancy complications	1568	325859
651	Multiple gestation	pregnancy complications	299	325859
652	Malposition & malpresentation of fetus or obstruction	pregnancy complications	1428	325859

Phecode	Description	Group	Cases	Controls	Phecode	Description	Group	Cases	Controls
331	Other cerebral degenerations	neurological	1287	295570	653	Problems associated with amniotic cavity & membranes	pregnancy complications	1257	169668
331.1	Hydrocephalus	neurological	515	295570	654.1	Abnormality of organs & soft tissues of pelvis complicating pregnancy, childbirth, or the puerperium	pregnancy complications	1034	169668
331.9	Cerebral degeneration, unspecified	neurological	514	295570	654.2	Rhesus isoimmunization in pregnancy	pregnancy complications	258	169668
332	Parkinson's disease	neurological	1345	295570	655	Known or suspected fetal abnormality affecting management of mother	pregnancy complications	3585	169668
333	Extrapyramidal disease & abnormal movement disorders	neurological	1094	295570	656	Other perinatal conditions of fetus or newborn	pregnancy complications	499	325859
333.1	Essential tremor	neurological	271	295570	661	Fetal distress & abnormal forces of labor		1819	325859
333.4	Torsion dystonia	neurological	265	295570	663	Umbilical cord complications during labor & delivery		511	325859
334	Degenerative disease of the spinal cord	neurological	1896	295570	665	Obstetrical/birth trauma	pregnancy complications	4394	325859
334.2	Anterior horn cell disease	neurological	277	295570	669	Complications of labor & delivery NEC	pregnancy complications	2212	169668
335	Multiple sclerosis	neurological	1222	295570	674	Other complications of the puerperium NEC	pregnancy complications	217	169668
337	Disorders of the autonomic nervous system	neurological	355	295570	681	Superficial cellulitis & abscess	dermatologic	7381	289596
338	Pain	neurological	742	295570	681.1	Cellulitis & abscess of fingers/toes	dermatologic	550	289596
338.1	Acute pain	neurological	363	295570	681.2	Cellulitis & abscess of face/neck	dermatologic	509	289596
338.2	Chronic pain	neurological	387	295570	681.3	Cellulitis & abscess of arm/hand	dermatologic	5521	289596
339	Other headache syndromes	neurological	7243	295570	681.5	Cellulitis & abscess of leg, except foot	dermatologic	5524	289596
340	Migraine	neurological	2962	295570	681.6	Cellulitis & abscess of foot, toe	dermatologic	5489	289596
340.1	Migrain with aura	neurological	217	295570	681.7	Cellulitis & abscess of trunk	dermatologic	623	289596
341	Other demyelinating diseases of central nervous system	neurological	221	295570	686	Other local infections of skin & subcutaneous tissue	dermatologic	1364	289596
342	Hemiplegia	neurological	1600	295570	686.1	Carbuncle & furuncle	dermatologic	2170	289596
344	Other paralytic syndromes	neurological	658	295570	686.3	Pilonidal cyst	dermatologic	510	289596
345	Epilepsy, recurrent seizures, convulsions	neurological	4890	295570	686.4	Pyogenic granuloma	dermatologic	337	289596
345.1	Epilepsy	neurological	877	295570	687	Symptoms affecting skin	dermatologic	1302	289596
345.11	Generalized convulsive epilepsy	neurological	423	295570	687.1	Rash & other nonspecific skin eruption	dermatologic	2100	289596
345.12	Partial epilepsy	neurological	273	295570	687.2	Localized superficial swelling, mass, or lump	dermatologic	947	289596
345.3	Convulsions	neurological	2204	295570	687.4	Disturbance of skin sensation	dermatologic	2787	289596
348	Other conditions of brain	neurological	854	295570	689	Disorder of skin & subcutaneous tissue NOS	dermatologic	5767	289596
348.2	Cerebral edema & compression of brain	neurological	280	295570	690	Erythematosquamous dermatosis	dermatologic	3042	289596
348.7	Coma	neurological	338	295570	690.1	Seborrheic dermatitis	dermatologic	3024	289596
348.8	Encephalopathy, not elsewhere classified	neurological	267	295570	694	Dyschromia & Vitiligo	dermatologic	952	289596
348.9	Other conditions of brain, NOS	neurological	440	295570	694.2	Other dyschromia	dermatologic	762	289596
349	Other & unspecified disorders of the nervous system	neurological	403	295570	695	Erythematous conditions	dermatologic	2344	289596
350	Abnormal movement	neurological	3488	295570	695.3	Rosacea	dermatologic	312	289596
350.1	Abnormal involuntary movements	neurological	968	295570	695.4	Lupus (localized & systemic)	dermatologic	391	288757
350.2	Abnormality of gait	neurological	2371	295570	695.42	Systemic lupus erythematosus	dermatologic	324	288757
350.3	Lack of coordination	neurological	368	295570	695.7	Prurigo & Lichen	dermatologic	724	289596
351	Other peripheral nerve disorders	neurological	11404	295570	695.8	Other specified erythematous conditions	dermatologic	231	289596
352	Disorders of other cranial nerves	neurological	1378	295570	695.9	Unspecified erythematous condition	dermatologic	486	289596
352.1	Trigeminal nerve disorders [CN5]	neurological	485	295570	696	Psoriasis & related disorders	dermatologic	2369	286399
352.2	Facial nerve disorders [CN7]	neurological	842	295570	696.4	Psoriasis	dermatologic	2308	286399
353	Nerve root & plexus disorders	neurological	2069	295570	696.41	Psoriasis vulgaris	dermatologic	1756	286399
355	Complex regional/central pain syndrome	neurological	329	295570	696.42	Psoriatic arthropathy	dermatologic	711	286399
357	Inflammatory & toxic neuropathy	neurological	1558	295570	697	Sarcoidosis	dermatologic	522	289596
358	Myoneural disorders	neurological	210	295570	698	Pruritus & related conditions	dermatologic	767	289596
359	Muscular dystrophies & other myopathies	neurological	439	295570	700	Corns & callosities	dermatologic	280	289596
359.2	Myopathy	neurological	337	295570	701	Other hypertrophic & atrophic conditions of skin	dermatologic	4868	289596
360	Disorders of the globe	sense organs	315	287331	701.2	Scar conditions & fibrosis of skin	dermatologic	2159	289596
360.2	Progressive myopia	sense organs	227	287331	701.3	Circumscribed scleroderma	dermatologic	478	289596
361	Retinal detachments & defects	sense organs	3118	287331	701.4	Keloid scar	dermatologic	205	289596
361.1	Retinal detachment with retinal defect	sense organs	1401	287331	701.5	Abnormal granulation tissue	dermatologic	447	289596
362	Other retinal disorders	sense organs	5021	286896	702	Degenerative skin conditions & other dermatoses	dermatologic	5494	289596
362.2	Degeneration of macula & posterior pole of retina	sense organs	2640	286896	702.1	Actinic keratosis	dermatologic	2726	289596
362.29	Macular degeneration (senile) of retina NOS	sense organs	2637	286896	702.2	Seborrheic keratosis	dermatologic	2973	289596
362.3	Other nondiabetic retinopathy	sense organs	1059	286896	703	Diseases of nail, NOS	dermatologic	345	289596
362.31	Separation of retinal layers	sense organs	1003	286896	703.1	Ingrown nail	dermatologic	837	289596
362.4	Retinal vascular changes & abnomalities	sense organs	872	286896	704	Diseases of hair & hair follicles	dermatologic	4724	289596
364	Corneal opacity & other disorders of cornea	sense organs	751	287331	705.8	Hyperhidrosis	dermatologic	569	289596
364.4	Corneal degenerations	sense organs	240	287331	706	Diseases of sebaceous glands	dermatologic	7950	289596
364.5	Corneal dystrophy	sense organs	236	287331	706.2	Sebaceous cyst	dermatologic	7878	289596
365	Glaucoma	sense organs	4986	287331	707	Chronic ulcer of skin	dermatologic	2310	289596
365.1	Open-angle glaucoma	sense organs	1216	287331	707.1	Decubitus ulcer	dermatologic	1184	289596
365.11	Primary open angle glaucoma	sense organs	1212	287331	707.2	Chronic ulcer of leg or foot	dermatologic	1199	289596
365.2	Primary angle-closure glaucoma	sense organs	742	287331	709	Diffuse diseases of connective tissue	dermatologic	3306	289596
366	Cataract	sense organs	22347	287331	709.2	Sicca syndrome	dermatologic	512	289596
366.2	Senile cataract	sense organs	9863	287331	709.7	Unspecified diffuse connective tissue disease	dermatologic	2592	289596
367	Disorders of refraction & accommodation; blindness & low vision	sense organs	2886	287331	710	Osteomyelitis, periostitis, & other infections involving bone	musculoskeletal	620	295120
367.1	Myopia	sense organs	1422	287331	710.1	Osteomyelitis	musculoskeletal	602	295120
367.2	Astigmatism	sense organs	225	287331	710.19	Unspecified osteomyelitis	musculoskeletal	444	295120
367.8	Hypermetropia	sense organs	278	287331	711	Arthropathy associated with infections	musculoskeletal	433	295120
367.9	Blindness & low vision	sense organs	950	287331	711.1	Pyogenic arthritis	musculoskeletal	333	295120
368	Visual disturbances	sense organs	3085	287331	714	Rheumatoid arthritis & other inflammatory polyarthropathies	musculoskeletal	4896	294409
368.1	Amblyopia	sense organs	607	287331	714.1	Rheumatoid arthritis	musculoskeletal	4369	294409
368.2	Diplopia & disorders of binocular vision	sense organs	723	287331	715	Other inflammatory spondylopathies	musculoskeletal	1641	294409
368.4	Visual field defects	sense organs	370	287331	715.2	Ankylosing spondylitis	musculoskeletal	399	294409
368.9	Subjective visual disturbances	sense organs	635	287331	716	Other arthropathies	musculoskeletal	38203	295120
368.91	Psychophysical visual disturbances	sense organs	222	287331	716.2	Unspecified monoarthritis	musculoskeletal	16046	295120
369	Infection of the eye	sense organs	574	287331	716.9	Arthropathy NOS	musculoskeletal	38053	295120

Phecode	Description	Group	Cases	Controls
369.5	Conjunctivitis, infectious	sense organs	241	287331
370	Keratitis	sense organs	283	287331
371	Inflammation of the eye	sense organs	2760	287331
371.1	Uveitis, noninfectious or NOS	sense organs	485	287331
371.3	Inflammation of eyelids	sense organs	2161	287331
372	Disorders of conjunctiva	sense organs	731	287331
374	Other disorders of eyelids	sense organs	5327	287331
374.1	Ectropion or entropion	sense organs	1101	287331
374.3	Ptosis of eyelid	sense organs	1725	287331
375	Disorders of lacrimal system	sense organs	2085	287331
375.2	Epiphora	sense organs	840	287331
377	Disorders of optic nerve & visual pathways	sense organs	438	287331
377.3	Optic neuritis/neuropathy	sense organs	234	287331
378	Strabismus & other disorders of binocular eye movements	sense organs	1329	287331
378.1	Strabismus (not specified as paralytic)	sense organs	836	287331
378.2	Nystagmus & other irregular eye movements	sense organs	209	287331
378.5	Paralytic strabismus	sense organs	264	287331
379	Other disorders of eye	sense organs	4107	287331
379.2	Disorders of vitreous body	sense organs	1443	287331
379.3	Aphakia & other disorders of lens	sense organs	2166	287331
379.5	Disorders of iris & ciliary body	sense organs	371	287331
380	Disorders of external ear	sense organs	614	287331
380.1	Otitis externa	sense organs	478	287331
380.4	Impacted cerumen	sense organs	323	287331
381	Otitis media & Eustachian tube disorders	sense organs	2003	287331
381.1	Otitis media	sense organs	1613	287331
381.11	Suppurative & unspecified otitis media	sense organs	739	287331
381.9	Otorrhea	sense organs	242	287331
382	Otalgia	sense organs	288	287331
383	Otosclerosis	sense organs	275	287331
384	Other disorders of tympanic membrane	sense organs	1161	287331
384.4	Perforation of tympanic membrane	sense organs	894	287331
385	Other disorders of middle ear & mastoid	sense organs	768	287331
385.3	Cholesteatoma	sense organs	521	287331
386	Vertiginous syndromes & other disorders of vestibular system	sense organs	1729	287331
386.1	Meniere's disease	sense organs	559	287331
386.2	Peripheral or central vertigo	sense organs	464	287331
386.3	Labyrinthitis	sense organs	710	287331
386.9	Dizziness & giddiness (Light-headedness & vertigo)	sense organs	4685	287331
389	Hearing loss	sense organs	4810	287331
389.1	Sensorineural hearing loss	sense organs	499	287331
389.2	Conductive hearing loss	sense organs	412	287331
389.4	Tinnitus	sense organs	539	287331
394	Rheumatic disease of the heart valves	circulatory system	5564	202269
394.1	Mitral valve stenosis & aortic valve stenosis	circulatory system	680	202269
394.2	Mitral valve disease	circulatory system	3323	202269
394.3	Aortic valve disease	circulatory system	1453	202269
394.7	Disease of tricuspid valve	circulatory system	1335	202269
395	Heart valve disorders	circulatory system	6317	202269
395.1	Nonrheumatic mitral valve disorders	circulatory system	3224	202269
395.2	Nonrheumatic aortic valve disorders	circulatory system	2915	202269
395.6	Heart valve replaced	circulatory system	1554	202269
396	Abnormal heart sounds	circulatory system	1382	202269
401	Hypertension	circulatory system	75408	202269
401.1	Essential hypertension	circulatory system	75160	202269
401.2	Hypertensive heart and/or renal disease	circulatory system	1667	202269
401.21	Hypertensive heart disease	circulatory system	395	202269
401.22	Hypertensive chronic kidney disease	circulatory system	1285	202269
402	Elevated blood pressure reading without diagnosis of hypertension	circulatory system	1464	202269
411	Ischemic Heart Disease	circulatory system	30489	202269
411.1	Unstable angina (intermediate coronary syndrome)	circulatory system	4478	202269
411.2	Myocardial infarction	circulatory system	12033	202269
411.3	Angina pectoris	circulatory system	15180	202269
411.4	Coronary atherosclerosis	circulatory system	19684	202269
411.41	Aneurysm & dissection of heart	circulatory system	735	202269
411.8	Other chronic ischemic heart disease, unspecified	circulatory system	15139	202269
411.9	Other acute & subacute forms of ischemic heart disease	circulatory system	1423	202269
414	Other forms of chronic heart disease	circulatory system	2245	202269
415	Pulmonary heart disease	circulatory system	4937	202269
415.2	Chronic pulmonary heart disease	circulatory system	976	202269
415.21	Primary pulmonary hypertension	circulatory system	414	202269
416	Cardiomegaly	circulatory system	3070	202269
418	Nonspecific chest pain	circulatory system	26417	202269
418.1	Precordial pain	circulatory system	3619	202269
420	Carditis	circulatory system	2276	202269
420.2	Pericarditis	circulatory system	1431	202269
420.21	Acute pericarditis	circulatory system	209	202269
420.3	Endocarditis	circulatory system	764	202269
425	Cardiomyopathy	circulatory system	1363	202269
425.1	Primary/intrinsic cardiomyopathies	circulatory system	1307	202269
426	Cardiac conduction disorders	circulatory system	7651	202269

Phecode	Description	Group	Cases	Controls
717	Polymyalgia Rheumatica	musculoskeletal	1354	336121
720	Spinal stenosis	musculoskeletal	4105	322347
721	Spondylosis & allied disorders	musculoskeletal	8311	322347
721.1	Spondylosis without myelopathy	musculoskeletal	7917	322347
721.2	Spondylosis with myelopathy	musculoskeletal	204	322347
721.8	Other allied disorders of spine	musculoskeletal	384	322347
722	Intervertebral disc disorders	musculoskeletal	6764	322347
722.1	Displacement of intervertebral disc	musculoskeletal	495	322347
722.6	Degeneration of intervertebral disc	musculoskeletal	2865	322347
722.7	Intervertebral disc disorder with myelopathy	musculoskeletal	398	322347
722.9	Other & unspecified disc disorder	musculoskeletal	4097	322347
723	Other disorders of cervical region	musculoskeletal	537	322347
724	Other & unspecified disorders of back	musculoskeletal	1156	322347
724.1	Disorders of sacrum	musculoskeletal	358	322347
724.2	Disorders of coccyx	musculoskeletal	361	322347
724.9	Other unspecified back disorders	musculoskeletal	723	322347
726	Peripheral enthesopathies & allied syndromes	musculoskeletal	14369	306383
726.1	Enthesopathy	musculoskeletal	9643	306383
726.2	Synoviopathy	musculoskeletal	527	306383
726.3	Bursitis	musculoskeletal	822	306383
726.4	Calcaneal spur; Exostosis NOS	musculoskeletal	3462	306383
727	Other disorders of synovium, tendon, & bursa	musculoskeletal	12755	306383
727.1	Synovitis & tenosynovitis	musculoskeletal	5004	306383
727.4	Ganglion & cyst of synovium, tendon, & bursa	musculoskeletal	2903	306383
727.5	Rupture of synovium	musculoskeletal	587	306383
727.6	Rupture of tendon, nontraumatic	musculoskeletal	3877	306383
728	Disorders of muscle, ligament, & fascia	musculoskeletal	832	306383
728.2	Laxity of ligament or hypermobility syndrome	musculoskeletal	402	306383
728.7	Fasciitis	musculoskeletal	3729	306383
728.71	Contracture of palmar fascia [Dupuytren's disease]	musculoskeletal	3406	306383
729	Other disorders of soft tissues	musculoskeletal	6003	306383
729.1	Rheumatism, unspecified & fibrositis	musculoskeletal	835	306383
731	Osteitis deformans & osteopathies associated with other disorders classified elsewhere	musculoskeletal	290	320016
731.1	Osteitis deformans [Paget's disease of bone]	musculoskeletal	239	320016
732	Osteochondropathies	musculoskeletal	461	320016
732.1	Juvenile osteochondrosis	musculoskeletal	205	320016
733	Other disorders of bone & cartilage	musculoskeletal	2100	320016
733.2	Cyst of bone	musculoskeletal	295	320016
733.4	Aseptic necrosis of bone	musculoskeletal	518	320016
733.8	Malunion & nonunion of fracture	musculoskeletal	1175	320016
735	Acquired foot deformities	musculoskeletal	8929	324005
735.1	Flat foot	musculoskeletal	215	324005
735.2	Acquired toe deformities	musculoskeletal	4749	324005
735.21	Hammer toe (acquired)	musculoskeletal	1813	324005
735.23	Hallux rigidus	musculoskeletal	1423	324005
735.3	Hallux valgus (Bunion)	musculoskeletal	6000	324005
736	Other acquired deformities of limbs	musculoskeletal	1764	324005
736.2	Acquired deformities of finger	musculoskeletal	234	324005
737	Curvature of spine	musculoskeletal	1419	324005
737.1	Kyphosis (acquired)	musculoskeletal	213	324005
737.3	Kyphoscoliosis & scoliosis	musculoskeletal	1174	324005
738	Other acquired musculoskeletal deformity	musculoskeletal	1991	324005
738.4	Acquired spondylolisthesis	musculoskeletal	1644	324005
739	Contracture of joint	musculoskeletal	496	324005
740	Osteoarthrosis	musculoskeletal	50335	287140
740.1	Osteoarthritis; localized	musculoskeletal	31034	287140
740.11	Osteoarthrosis, localized, primary	musculoskeletal	8866	287140
740.12	Osteoarthrosis, localized, secondary	musculoskeletal	634	287140
740.2	Osteoarthrosis, generalized	musculoskeletal	4353	287140
740.9	Osteoarthrosis NOS	musculoskeletal	28265	287140
741	Symptoms & disorders of the joints	musculoskeletal	3744	331152
741.2	Stiffness of joint	musculoskeletal	590	331152
741.4	Joint effusions	musculoskeletal	1400	331152
742	Derangement of joint, non-traumatic	musculoskeletal	2856	331152
742.1	Loose body in joint	musculoskeletal	297	331152
742.2	Pathological, developmental or recurrent dislocation	musculoskeletal	383	331152
742.8	Articular cartilage disorder	musculoskeletal	970	331152
742.9	Other derangement of joint	musculoskeletal	2384	331152
743	Osteoporosis, osteopenia & pathological fracture	musculoskeletal	7552	329923
743.1	Osteoporosis	musculoskeletal	6241	329923
743.11	Osteoporosis NOS	musculoskeletal	6220	329923
743.2	Pathologic fracture	musculoskeletal	571	329923
743.21	Pathologic fracture of vertebrae	musculoskeletal	325	329923
743.9	Osteopenia or other disorder of bone & cartilage	musculoskeletal	836	329923
745	Pain in joint	musculoskeletal	7733	329742
747	Cardiac & circulatory congenital anomalies	congenital anomalies	3002	329064
747.1	Cardiac congenital anomalies	congenital anomalies	2826	329064
747.11	Cardiac shunt/ heart septal defect	congenital anomalies	576	329064
747.12	Valvular heart disease/ heart chambers	congenital anomalies	270	329064
747.13	Congenital anomalies of great vessels	congenital anomalies	2015	329064
749	Congenital anomalies of face & neck	congenital anomalies	295	329064

Phecode	Description	Group	Cases	Controls	Phecode	Description	Group	Cases	Controls
426.2	Atrioventricular [AV] block	circulatory system	2459	202269	750	Digestive congenital anomalies	congenital anomalies	706	329064
426.21	First degree AV block	circulatory system	1220	202269	750.1	Upper gastrointestinal congenital anomalies	congenital anomalies	351	329064
426.23	Second degree AV block	circulatory system	508	202269	750.13	Congenital anomalies of mouth/tongue	congenital anomalies	204	329064
426.24	Atrioventricular block, complete	circulatory system	659	202269	750.2	Lower gastrointestinal congenital anomalies	congenital anomalies	348	329064
426.3	Bundle branch block	circulatory system	3818	202269	750.21	Congenital anomalies of intestine	congenital anomalies	222	329064
426.31	Right bundle branch block	circulatory system	1758	202269	751	Genitourinary congenital anomalies	congenital anomalies	1465	329064
426.32	Left bundle branch block	circulatory system	2030	202269	751.1	Congenital anomalies of genital organs	congenital anomalies	633	329064
426.4	Anomalous atrioventricular excitation	circulatory system	210	202269	751.11	Congenital anomalies of female genital organs	congenital anomalies	392	176821
426.9	Cardiac pacemaker/device in situ	circulatory system	2715	202269	751.12	Congenital anomalies of male genital organs	congenital anomalies	205	152243
426.91	Cardiac pacemaker in situ	circulatory system	2511	202269	751.2	Congenital anomalies of urinary system	congenital anomalies	801	329064
427	Cardiac dysrhythmias	circulatory system	25228	202269	751.21	Cystic kidney disease	congenital anomalies	394	329064
427.1	Paroxysmal tachycardia, unspecified	circulatory system	3202	202269	751.22	Other specified congenital anomalies of kidney	congenital anomalies	333	329064
427.11	Paroxysmal supraventricular tachycardia	circulatory system	2326	202269	752	Nervous system congenital anomalies	congenital anomalies	1689	329064
427.12	Paroxysmal ventricular tachycardia	circulatory system	956	202269	752.1	Neural tube defects	congenital anomalies	1613	329064
427.2	Atrial fibrillation & flutter	circulatory system	15491	202269	754	Congenital musculoskeletal deformities of spine	congenital anomalies	1491	329064
427.21	Atrial fibrillation	circulatory system	983	202269	755	Congenital anomalies of limbs	congenital anomalies	432	329064
427.3	Other specified cardiac dysrhythmias	circulatory system	3400	202269	755.1	Congenital deformities of feet	congenital anomalies	222	329064
427.4	Cardiac arrest & ventricular fibrillation	circulatory system	1324	202269	756	Other congenital musculoskeletal anomalies	congenital anomalies	590	329064
427.41	Ventricular fibrillation & flutter	circulatory system	365	202269	756.5	Congenital osteodystrophy	congenital anomalies	425	329064
427.42	Cardiac arrest	circulatory system	1121	202269	759	Other & unspecified congenital anomalies	congenital anomalies	223	329064
427.5	Arrhythmia (cardiac) NOS	circulatory system	998	202269	760	Back pain	symptoms	10889	257697
427.6	Premature beats	circulatory system	837	202269	761	Cervicalgia	symptoms	1764	257697
427.7	Tachycardia NOS	circulatory system	2398	202269	764	Sciatica	symptoms	2339	257697
427.8	Sinoatrial node dysfunction (Bradycardia)	circulatory system	484	202269	765	Cervical radiculitis	symptoms	505	257697
427.9	Palpitations	circulatory system	3694	202269	766	Neuralgia, neuritis, & radiculitis NOS	symptoms	1265	257697
428	Congestive heart failure; nonhypertensive	circulatory system	6185	202269	770	Myalgia & myositis unspecified	symptoms	1789	257697
428.1	Congestive heart failure (CHF) NOS	circulatory system	3650	202269	771	Musculoskeletal symptoms referable to limbs	symptoms	776	257697
428.2	Heart failure NOS	circulatory system	4914	202269	771.1	Swelling of limb	symptoms	4680	257697
429	Ill-defined descriptions & complications of heart disease	circulatory system	756	202269	772	Symptoms of the muscles	symptoms	676	257697
429.2	Abnormal function study of cardiovascular system	circulatory system	553	202269	773	Pain in limb	symptoms	5984	257697
430	Intracranial hemorrhage	circulatory system	1984	202269	780	Hypothermia/Chills	symptoms	212	257697
430.1	Subarachnoid hemorrhage	circulatory system	816	202269	781	Symptoms involving nervous & musculoskeletal systems	symptoms	9086	257697
430.2	Intracerebral hemorrhage	circulatory system	855	202269	782.3	Edema	symptoms	1941	257697
430.3	Subdural hemorrhage	circulatory system	308	202269	783	Fever of unknown origin	symptoms	3998	257697
433	Cerebrovascular disease	circulatory system	9077	202269	785	Abdominal pain	symptoms	37426	257697
433.1	Occlusion & stenosis of precerebral arteries	circulatory system	1354	202269	788	Syncope & collapse	symptoms	9030	257697
433.12	Cerebral atherosclerosis	circulatory system	270	202269	789	Nausea & vomiting	symptoms	11420	257697
433.2	Occlusion of cerebral arteries	circulatory system	4355	202269	790.6	Other abnormal blood chemistry	symptoms	6857	257697
433.21	Cerebral artery occlusion, with cerebral infarction	circulatory system	4193	202269	791	Gangrene	symptoms	645	257697
433.3	Cerebral ischemia	circulatory system	3089	202269	793.2	Nonspecific abnormal findings on radiological & other examination of other intrathoracic organs	symptoms	608	257697
433.31	Transient cerebral ischemia	circulatory system	2115	202269	798	Malaise & fatigue	symptoms	3438	257697
433.5	Cerebral aneurysm	circulatory system	364	202269	798.1	Chronic fatigue syndrome	symptoms	607	257697
433.8	Late effects of cerebrovascular disease	circulatory system	1333	202269	800	Fracture of lower limb	injuries & poisonings	6862	243043
440	Atherosclerosis	circulatory system	1453	202269	800.1	Fracture of neck of femur	injuries & poisonings	1926	243043
440.2	Atherosclerosis of the extremities	circulatory system	848	202269	800.2	Fracture of unspecified part of femur	injuries & poisonings	888	243043
440.9	Atherosclerosis of aorta	circulatory system	331	202269	800.3	Fracture of tibia & fibula	injuries & poisonings	1976	243043
441	Vascular insufficiency of intestine	circulatory system	701	202269	800.4	Fracture of patella	injuries & poisonings	689	243043
441.1	Acute vascular insufficiency of intestine	circulatory system	321	202269	801	Fracture of ankle & foot	injuries & poisonings	1992	243043
442	Other aneurysm	circulatory system	2079	202269	801.1	Fracture of foot	injuries & poisonings	1220	243043
442.1	Aortic aneurysm	circulatory system	1632	202269	802	Fracture of pelvis	injuries & poisonings	759	243043
442.11	Abdominal aortic aneurysm	circulatory system	1047	202269	803	Fracture of upper limb	injuries & poisonings	8356	243043
442.8	Aneurysm of other specified artery	circulatory system	218	202269	803.1	Fracture of humerus	injuries & poisonings	1610	243043
443	Peripheral vascular disease	circulatory system	4031	202269	803.2	Fracture of radius & ulna	injuries & poisonings	5165	243043
443.1	Raynaud's syndrome	circulatory system	1162	202269	803.3	Fracture of clavicle or scapula	injuries & poisonings	1581	243043
443.7	Peripheral angiopathy in diseases classified elsewhere	circulatory system	397	202269	804	Fracture of hand or wrist	injuries & poisonings	3156	243043
443.9	Peripheral vascular disease, unspecified	circulatory system	2679	202269	805	Fracture of vertebral column without mention of spinal cord injury	injuries & poisonings	1685	243043
444	Arterial embolism & thrombosis	circulatory system	903	202269	807	Fracture of ribs	injuries & poisonings	1555	243043
444.1	Arterial embolism & thrombosis of lower extremity artery	circulatory system	560	202269	809	Fracture of unspecified bones	injuries & poisonings	10448	243043
446	Polyarteritis nodosa & allied conditions	circulatory system	857	202269	817	Concussion	injuries & poisonings	219	243043
446.5	Giant cell arteritis	circulatory system	417	202269	818	Intracranial hemorrhage (injury)	injuries & poisonings	667	243043
446.9	Arteritis NOS	circulatory system	258	202269	819	Skull & face fracture & other intercranial injury	injuries & poisonings	2986	243043
447	Other disorders of arteries & arterioles	circulatory system	1320	202269	830	Dislocation	injuries & poisonings	1954	243043
447.1	Stricture of artery	circulatory system	858	202269	835	Internal derangement of knee	injuries & poisonings	13967	243043
450	Noninfectious disorders of lymphatic channels	circulatory system	805	202269	836	Traumatic arthropathy	injuries & poisonings	498	243043
451	Phlebitis & thrombophlebitis	circulatory system	3916	202269	840	Sprains & strains	injuries & poisonings	866	243043
451.2	Phlebitis & thrombophlebitis of lower extremities	circulatory system	3612	202269	840.3	Joint/ligament sprain	injuries & poisonings	457	243043
452	Other venous embolism & thrombosis	circulatory system	598	202269	850	Hemorrhage or hematoma complicating a procedure	injuries & poisonings	5032	243043
454	Varicose veins	circulatory system	10793	202269	851	Complications of transplants & reattached limbs	injuries & poisonings	17270	243043
454.1	Varicose veins of lower extremity	circulatory system	10333	202269	853	Complication of colostomy or enterostomy	injuries & poisonings	494	243043
454.11	Varicose veins of lower extremity, symptomtic	circulatory system	716	202269	854	Complications of cardiac/vascular device, implant, & graft	injuries & poisonings	2535	243043
455	Hemorrhoids	circulatory system	23176	202269	857	Mechanical complication of unspecified genitourinary device, implant, & graft	injuries & poisonings	1436	243043
456	Chronic venous insufficiency [CVI]	circulatory system	224	202269	858	Complication of internal orthopedic device	injuries & poisonings	3502	243043
458	Hypotension	circulatory system	6659	202269	859	Complication due to other implant & internal device	injuries & poisonings	3509	243043

Phecode	Description	Group	Cases	Controls	Phecode	Description	Group	Cases	Controls
458.1	Orthostatic hypotension	circulatory system	1622	202269	870	Open wounds of head; neck; & trunk	injuries & poisonings	3784	243043
458.2	Iatrogenic hypotension	circulatory system	250	202269	870.1	Open wound or laceration of eye or eyelid	injuries & poisonings	522	243043
458.9	Hypotension NOS	circulatory system	4073	202269	870.3	Other open wound of head & face	injuries & poisonings	2938	243043
459	Other disorders of circulatory system	circulatory system	17142	202269	870.4	Open wound of nose & sinus	injuries & poisonings	247	243043
459.9	Circulatory disease NEC	circulatory system	16931	202269	870.5	Open wound of lip & mouth	injuries & poisonings	374	243043
465	Acute upper respiratory infections of multiple or unspecified sites	respiratory	2223	269389	871	Open wounds of extremities	injuries & poisonings	4405	243043
465.2	Acute pharyngitis	respiratory	841	269389	872	Traumatic amputation	injuries & poisonings	435	243043
470	Septal Deviations/Turbinate Hypertrophy	respiratory	4226	269389	873	Broken tooth	injuries & poisonings	250	243043
471	Nasal polyps	respiratory	2933	269389	907	Injuries to the nervous system	injuries & poisonings	1285	243043
472	Chronic pharyngitis & nasopharyngitis	respiratory	906	269389	915	Superficial injury without mention of infection	injuries & poisonings	4691	243043
473	Diseases of the larynx & vocal cords	respiratory	2414	269389	916	Contusion	injuries & poisonings	1533	243043
473.3	Paralysis/spasm of vocal cords or larynx	respiratory	263	269389	938	Dermatitis due to solar radiation	dermatologic	368	288850
473.4	Voice disturbance	respiratory	972	269389	938.2	Chronic dermatitis due to solar radiation	dermatologic	256	288850
474	Acute & chronic tonsillitis	respiratory	1762	269389	939	Atopic/contact dermatitis due to other or unspecified	dermatologic	2227	288850
474.1	Acute tonsillitis	respiratory	552	269389	941	Adverse reaction to serum or vaccine	injuries & poisonings	259	241796
474.2	Chronic tonsillitis & adenoiditis	respiratory	971	269389	946	Anaphylactic shock NOS	injuries & poisonings	527	241796
475	Chronic sinusitis	respiratory	2395	269389	947	Urticaria	dermatologic	541	288850
476	Allergic rhinitis	respiratory	1225	269389	949	Allergies, other	injuries & poisonings	397	241796
477	Epistaxis or throat hemorrhage	respiratory	2412	269389	958	Certain early complications of trauma or procedure	injuries & poisonings	531	243043
478	Throat pain	respiratory	338	269389	960	Poisoning by antibiotics	injuries & poisonings	18479	243043
479	Other upper respiratory disease	respiratory	3786	269389	960.2	Allergy/adverse effect of penicillin	injuries & poisonings	16028	243043
480	Pneumonia	respiratory	11693	269389	961	Poisoning by other anti-infectives	injuries & poisonings	411	243043
480.1	Bacterial pneumonia	respiratory	7555	269389	961.1	Poisoning/allergy of sulfonamides	injuries & poisonings	842	243043
480.11	Pneumococcal pneumonia	respiratory	6741	269389	962	Poisoning by hormones & synthetic substitutes	injuries & poisonings	482	243043
480.5	Bronchopneumonia & lung abscess	respiratory	894	269389	962.3	Hormones & synthetic substitutes causing adverse effects in therapeutic use	injuries & poisonings	357	243043
481	Influenza	respiratory	418	269389	963	Poisoning by primarily systemic agents	injuries & poisonings	2626	243043
483	Acute bronchitis & bronchiolitis	respiratory	227	269389	964	Poisoning by agents primarily affecting blood constituents	injuries & poisonings	391	243043
495	Asthma	respiratory	24896	269389	964.1	Anticoagulants causing adverse effects	injuries & poisonings	298	243043
495.2	Asthma with exacerbation	respiratory	229	269389	965	Poisoning by analgesics, antipyretics, & antirheumatics	injuries & poisonings	6181	243043
496	Chronic airway obstruction	respiratory	11361	269389	965.1	Opiates & related narcotics causing adverse effects in therapeutic use	injuries & poisonings	1370	243043
496.1	Emphysema	respiratory	1937	269389	965.3	Salicylates causing adverse effects in therapeutic use	injuries & poisonings	378	243043
496.2	Chronic bronchitis	respiratory	3258	269389	966	Poisoning by anticonvulsants & anti-Parkinsonism drugs	injuries & poisonings	507	243043
496.21	Obstructive chronic bronchitis	respiratory	2997	269389	967	Adverse effects of sedatives or other central nervous system depressants & anesthetics	injuries & poisonings	515	243043
496.3	Bronchiectasis	respiratory	2143	269389	969	Poisoning by psychotropic agents	injuries & poisonings	1748	243043
497	Bronchitis	respiratory	647	269389	971	Poisoning by drugs primarily affecting the autonomic nervous system	injuries & poisonings	511	243043
500	Lung disease due to external agents	respiratory	459	269389	972	Poisoning by agents primarily affecting the cardiovascular system	injuries & poisonings	828	243043
500.2	Pneumoconiosis	respiratory	273	269389	973	Poisoning by agents primarily affecting the gastrointestinal system	injuries & poisonings	211	243043
501	Pneumonitis due to inhalation of food or vomitus	respiratory	852	269389	977	Personal history of allergy to medicinal agents	injuries & poisonings	5864	243043
502	Postinflammatory pulmonary fibrosis	respiratory	1036	269389	979	Adverse drug events & drug allergies	injuries & poisonings	692	243043
503	Pulmonary congestion & hypostasis	respiratory	534	269389	981	Toxic effect of (non-ethyl) alcohol & petroleum & other solvents	injuries & poisonings	262	243043
504	Other alveolar & parietoalveolar pneumonopathy	respiratory	389	269389	990	Effects radiation NOS	injuries & poisonings	3850	243043
506	Empyema & pneumothorax	respiratory	1207	269389	994	Sepsis & SIRS	injuries & poisonings	3970	243043
					994.2	Sepsis	injuries & poisonings	3970	243043

Supplementary Table 5: Power to detect associations with disease outcomes based on available numbers of cases, alpha 5%, instrument strength determined from the UK Biobank (PCSK9 r2=0.12%; HMGCR r2=0.15%; NPC1L1 r2=0.026%; LDLR r2=0.64%) and four different assumed effect sizes per 1mg/dL decrease in LDL-C (beta=0.2; beta=0.5; beta=1.0; beta=1.5). Phenotypes are listed by phecode number, as specified above in Supplementary Table 4.

			PCSK9				HMGCR				NPC1L1				LDLR			
Phenotype	Cases, n	Controls, n	Power at beta=0.2	Power at beta=0.5	Power at beta=1.0	Power at beta=1.5	Power at beta=0.2	Power at beta=0.5	Power at beta=1.0	Power at beta=1.5	Power at beta=0.2	Power at beta=0.5	Power at beta=1.0	Power at beta=1.5	Power at beta=0.2	Power at beta=0.5	Power at beta=1.0	Power at beta=1.5
008	10361	304892	0.10	0.42	0.94	1.00	0.12	0.48	0.97	1.00	0.05	0.13	0.37	0.69	0.36	0.98	1.00	1.00
008.5	2546	304892	0.05	0.14	0.42	0.75	0.06	0.16	0.49	0.82	0.04	0.06	0.13	0.23	0.12	0.52	0.98	1.00
008.52	673	304892	0.04	0.07	0.15	0.28	0.04	0.07	0.17	0.32	0.03	0.04	0.06	0.09	0.06	0.18	0.54	0.87
008.6	903	304892	0.04	0.08	0.18	0.35	0.04	0.08	0.21	0.41	0.03	0.04	0.07	0.11	0.07	0.22	0.67	0.95
038	4885	304892	0.07	0.23	0.68	0.95	0.08	0.26	0.76	0.98	0.04	0.08	0.20	0.39	0.20	0.79	1.00	1.00
038.1	962	304892	0.04	0.08	0.19	0.37	0.04	0.09	0.22	0.43	0.03	0.04	0.07	0.11	0.07	0.23	0.70	0.96
038.2	506	304892	0.04	0.06	0.12	0.22	0.04	0.06	0.14	0.25	0.03	0.04	0.06	0.08	0.05	0.14	0.43	0.77
041	11864	304892	0.11	0.47	0.96	1.00	0.13	0.53	0.98	1.00	0.05	0.14	0.41	0.74	0.40	0.99	1.00	1.00
041.1	3057	304892	0.06	0.16	0.49	0.82	0.06	0.18	0.56	0.88	0.04	0.07	0.14	0.27	0.14	0.59	0.99	1.00
041.11	2191	304892	0.05	0.13	0.37	0.69	0.05	0.14	0.43	0.76	0.04	0.06	0.11	0.21	0.11	0.46	0.96	1.00
041.2	1659	304892	0.05	0.11	0.30	0.57	0.05	0.12	0.34	0.65	0.03	0.05	0.10	0.17	0.09	0.37	0.90	1.00
041.4	2977	304892	0.06	0.16	0.48	0.81	0.06	0.18	0.55	0.88	0.04	0.06	0.14	0.26	0.14	0.58	0.99	1.00
053	426	304892	0.03	0.05	0.11	0.19	0.04	0.06	0.12	0.22	0.03	0.04	0.05	0.07	0.05	0.13	0.38	0.69
054	207	304892	0.03	0.04	0.07	0.11	0.03	0.05	0.08	0.13	0.03	0.03	0.04	0.05	0.04	0.08	0.21	0.41
070	580	304892	0.04	0.06	0.13	0.24	0.04	0.07	0.15	0.28	0.03	0.04	0.06	0.08	0.06	0.16	0.48	0.82
070.3	237	304892	0.03	0.05	0.08	0.12	0.03	0.05	0.09	0.14	0.03	0.03	0.04	0.06	0.04	0.09	0.23	0.45
070.4	261	304892	0.03	0.05	0.08	0.13	0.03	0.05	0.09	0.15	0.03	0.03	0.04	0.06	0.04	0.09	0.25	0.49
070.9	476	304892	0.04	0.06	0.12	0.21	0.04	0.06	0.13	0.24	0.03	0.04	0.05	0.08	0.05	0.14	0.41	0.74
078	1176	304892	0.04	0.09	0.22	0.44	0.04	0.10	0.26	0.50	0.03	0.05	0.08	0.13	0.08	0.28	0.78	0.98
079	2704	304892	0.06	0.15	0.44	0.78	0.06	0.17	0.51	0.84	0.04	0.06	0.13	0.24	0.13	0.54	0.99	1.00
079.9	1577	304892	0.05	0.10	0.28	0.55	0.05	0.11	0.33	0.62	0.03	0.05	0.09	0.16	0.09	0.35	0.89	1.00
080	4201	304892	0.07	0.20	0.62	0.92	0.07	0.23	0.69	0.96	0.04	0.08	0.18	0.35	0.18	0.73	1.00	1.00
081	2438	304892	0.05	0.14	0.41	0.73	0.06	0.15	0.47	0.81	0.04	0.06	0.12	0.22	0.12	0.50	0.98	1.00
1000	511	336964	0.04	0.06	0.12	0.22	0.04	0.06	0.14	0.25	0.03	0.04	0.06	0.08	0.05	0.14	0.44	0.77
1001	1110	336365	0.04	0.08	0.21	0.42	0.04	0.09	0.25	0.48	0.03	0.05	0.08	0.13	0.08	0.26	0.76	0.98
1002	5846	331629	0.08	0.26	0.76	0.98	0.08	0.31	0.83	0.99	0.04	0.09	0.23	0.46	0.23	0.86	1.00	1.00
1004	444	337031	0.03	0.06	0.11	0.20	0.04	0.06	0.12	0.23	0.03	0.04	0.05	0.07	0.05	0.13	0.39	0.71
1005	1476	335999	0.05	0.10	0.27	0.52	0.05	0.11	0.31	0.60	0.03	0.05	0.09	0.15	0.09	0.33	0.86	1.00
1006	200	337275	0.03	0.04	0.07	0.11	0.03	0.05	0.08	0.13	0.03	0.03	0.04	0.05	0.04	0.08	0.20	0.39
1007	281	337194	0.03	0.05	0.08	0.14	0.03	0.05	0.09	0.16	0.03	0.03	0.05	0.06	0.05	0.10	0.27	0.52
1008	1051	336424	0.04	0.08	0.20	0.40	0.04	0.09	0.24	0.46	0.03	0.04	0.08	0.12	0.07	0.25	0.73	0.97
1009	7326	330149	0.09	0.32	0.84	0.99	0.09	0.37	0.90	1.00	0.05	0.10	0.28	0.54	0.27	0.92	1.00	1.00
1010	16572	320903	0.14	0.59	0.99	1.00	0.16	0.67	1.00	1.00	0.06	0.17	0.53	0.87	0.52	1.00	1.00	1.00
1011	10083	327392	0.10	0.41	0.93	1.00	0.11	0.47	0.97	1.00	0.05	0.12	0.36	0.68	0.35	0.98	1.00	1.00
1013	349	337126	0.03	0.05	0.10	0.16	0.03	0.05	0.11	0.19	0.03	0.04	0.05	0.07	0.05	0.11	0.32	0.61
1014	254	337221	0.03	0.05	0.08	0.13	0.03	0.05	0.09	0.15	0.03	0.03	0.04	0.06	0.04	0.09	0.25	0.48
1015	10460	327015	0.10	0.42	0.94	1.00	0.12	0.49	0.97	1.00	0.05	0.13	0.37	0.69	0.36	0.98	1.00	1.00
1019	13786	323689	0.12	0.52	0.98	1.00	0.14	0.60	0.99	1.00	0.06	0.15	0.46	0.80	0.45	1.00	1.00	1.00
110	294	304892	0.03	0.05	0.09	0.14	0.03	0.05	0.10	0.16	0.03	0.03	0.05	0.06	0.05	0.10	0.28	0.54
110.1	288	304892	0.03	0.05	0.09	0.14	0.03	0.05	0.10	0.16	0.03	0.03	0.05	0.06	0.05	0.10	0.27	0.53
1100	2277	335198	0.05	0.13	0.38	0.71	0.06	0.15	0.44	0.78	0.04	0.06	0.12	0.21	0.11	0.48	0.97	1.00
112	2400	304892	0.05	0.13	0.40	0.73	0.06	0.15	0.46	0.80	0.04	0.06	0.12	0.22	0.12	0.50	0.97	1.00
117	307	304892	0.03	0.05	0.09	0.15	0.03	0.05	0.10	0.17	0.03	0.03	0.05	0.06	0.05	0.10	0.29	0.55
136	237	304892	0.03	0.05	0.08	0.12	0.03	0.05	0.09	0.14	0.03	0.03	0.04	0.06	0.04	0.09	0.23	0.45
145	627	234242	0.04	0.06	0.14	0.26	0.04	0.07	0.16	0.30	0.03	0.04	0.06	0.09	0.06	0.17	0.51	0.85
145.2	303	234242	0.03	0.05	0.09	0.15	0.03	0.05	0.10	0.17	0.03	0.03	0.05	0.06	0.05	0.10	0.28	0.55
149	630	234242	0.04	0.06	0.14	0.26	0.04	0.07	0.16	0.30	0.03	0.04	0.06	0.09	0.06	0.17	0.52	0.85
149.1	257	234242	0.03	0.05	0.08	0.13	0.03	0.05	0.09	0.15	0.03	0.03	0.04	0.06	0.04	0.09	0.25	0.48
149.4	252	234242	0.03	0.05	0.08	0.13	0.03	0.05	0.09	0.15	0.03	0.03	0.04	0.06	0.04	0.09	0.24	0.48
150	817	234242	0.04	0.07	0.17	0.32	0.04	0.08	0.19	0.37	0.03	0.04	0.07	0.10	0.07	0.21	0.62	0.93
151	603	234242	0.04	0.06	0.14	0.25	0.04	0.07	0.15	0.29	0.03	0.04	0.06	0.09	0.06	0.16	0.50	0.84
153	4541	234242	0.07	0.21	0.65	0.94	0.07	0.25	0.72	0.97	0.04	0.08	0.19	0.37	0.19	0.76	1.00	1.00
153.2	3053	234242	0.06	0.16	0.49	0.82	0.06	0.18	0.56	0.88	0.04	0.07	0.14	0.27	0.14	0.59	0.99	1.00
153.3	2169	234242	0.05	0.13	0.37	0.68	0.05	0.14	0.43	0.76	0.04	0.06	0.11	0.20	0.11	0.46	0.96	1.00
155	449	234242	0.04	0.06	0.11	0.20	0.04	0.06	0.13	0.23	0.03	0.04	0.05	0.07	0.05	0.13	0.39	0.72
157	756	234242	0.04	0.07	0.16	0.30	0.04	0.08	0.18	0.35	0.03	0.04	0.07	0.10	0.06	0.19	0.59	0.91
158	1091	234242	0.04	0.08	0.21	0.41	0.04	0.09	0.24	0.47	0.03	0.05	0.08	0.12	0.08	0.26	0.75	0.98
159	5580	234242	0.07	0.25	0.73	0.97	0.08	0.29	0.81	0.99	0.04	0.09	0.22	0.44	0.22	0.84	1.00	1.00
159.2	204	234242	0.03	0.04	0.07	0.11	0.03	0.05	0.08	0.13	0.03	0.03	0.04	0.05	0.04	0.08	0.21	0.40
159.3	233	234242	0.03	0.05	0.08	0.12	0.03	0.05	0.08	0.14	0.03	0.03	0.04	0.06	0.04	0.09	0.23	0.45
159.4	205	234242	0.03	0.04	0.07	0.11	0.03	0.05	0.08	0.13	0.03	0.03	0.04	0.05	0.04	0.08	0.21	0.40
165	3012	234242	0.06	0.16	0.48	0.82	0.06	0.18	0.55	0.88	0.04	0.06	0.14	0.26	0.14	0.59	0.99	1.00
165.1	2617	234242	0.05	0.14	0.43	0.76	0.06	0.16	0.50	0.83	0.04	0.06	0.13	0.24	0.13	0.53	0.98	1.00
170	443	234242	0.03	0.06	0.11	0.20	0.04	0.06	0.12	0.23	0.03	0.04	0.05	0.07	0.05	0.13	0.39	0.71
170.2	320	234242	0.03	0.05	0.09	0.15	0.03	0.05	0.10	0.18	0.03	0.03	0.05	0.06	0.05	0.11	0.30	0.57
172	14207	234242	0.13	0.53	0.98	1.00	0.14	0.60	0.99	1.00	0.06	0.15	0.47	0.81	0.46	1.00	1.00	1.00
172.1	2739	234242	0.06	0.15	0.45	0.78	0.06	0.17	0.51	0.85	0.04	0.06	0.13	0.25	0.13	0.55	0.99	1.00

172.11	2739	234242	0.06	0.15	0.45	0.78	0.06	0.17	0.51	0.85	0.04	0.06	0.13	0.25	0.13	0.55	0.99	1.00
172.2	11594	234242	0.11	0.45	0.96	1.00	0.12	0.52	0.98	1.00	0.05	0.13	0.40	0.73	0.39	0.99	1.00	1.00
172.3	741	234242	0.04	0.07	0.16	0.30	0.04	0.07	0.18	0.34	0.03	0.04	0.06	0.10	0.06	0.19	0.58	0.90
174	12023	232710	0.11	0.47	0.96	1.00	0.13	0.53	0.98	1.00	0.05	0.14	0.41	0.74	0.40	0.99	1.00	1.00
174.1	11848	120733	0.11	0.44	0.95	1.00	0.12	0.51	0.98	1.00	0.05	0.13	0.39	0.72	0.38	0.99	1.00	1.00
174.11	11108	120733	0.11	0.42	0.94	1.00	0.12	0.49	0.97	1.00	0.05	0.13	0.37	0.69	0.36	0.98	1.00	1.00
175	3261	120184	0.06	0.17	0.51	0.84	0.06	0.19	0.58	0.90	0.04	0.07	0.15	0.28	0.14	0.61	0.99	1.00
180	3299	122212	0.06	0.17	0.51	0.85	0.06	0.19	0.58	0.90	0.04	0.07	0.15	0.28	0.15	0.62	0.99	1.00
180.1	1622	122212	0.05	0.10	0.29	0.56	0.05	0.12	0.33	0.63	0.03	0.05	0.10	0.16	0.09	0.36	0.89	1.00
180.3	1757	122212	0.05	0.11	0.31	0.59	0.05	0.12	0.36	0.67	0.03	0.05	0.10	0.17	0.10	0.38	0.91	1.00
182	1240	122251	0.04	0.09	0.23	0.45	0.05	0.10	0.27	0.52	0.03	0.05	0.08	0.13	0.08	0.29	0.80	0.99
184	2388	122251	0.05	0.13	0.40	0.72	0.06	0.15	0.46	0.79	0.04	0.06	0.12	0.22	0.12	0.49	0.97	1.00
184.1	2091	122251	0.05	0.12	0.36	0.66	0.05	0.14	0.41	0.74	0.03	0.06	0.11	0.20	0.11	0.44	0.95	1.00
184.11	2072	122251	0.05	0.12	0.35	0.66	0.05	0.14	0.41	0.74	0.03	0.06	0.11	0.20	0.11	0.44	0.95	1.00
184.2	281	122251	0.03	0.05	0.08	0.14	0.03	0.05	0.09	0.16	0.03	0.03	0.05	0.06	0.05	0.10	0.27	0.52
185	7072	105218	0.08	0.30	0.81	0.99	0.09	0.34	0.88	1.00	0.05	0.10	0.26	0.51	0.25	0.90	1.00	1.00
187	3330	111991	0.06	0.17	0.51	0.85	0.06	0.19	0.59	0.90	0.04	0.07	0.15	0.28	0.15	0.62	1.00	1.00
187.1	3085	111991	0.06	0.16	0.48	0.82	0.06	0.18	0.55	0.88	0.04	0.06	0.14	0.27	0.14	0.59	0.99	1.00
187.2	3194	111991	0.06	0.16	0.50	0.83	0.06	0.19	0.57	0.89	0.04	0.07	0.15	0.27	0.14	0.60	0.99	1.00
189	4104	234242	0.06	0.20	0.60	0.92	0.07	0.23	0.68	0.95	0.04	0.07	0.18	0.34	0.17	0.72	1.00	1.00
189.1	1098	234242	0.04	0.08	0.21	0.41	0.04	0.09	0.24	0.48	0.03	0.05	0.08	0.12	0.08	0.26	0.75	0.98
189.11	1055	234242	0.04	0.08	0.20	0.40	0.04	0.09	0.24	0.46	0.03	0.04	0.08	0.12	0.07	0.25	0.73	0.97
189.2	2339	234242	0.05	0.13	0.39	0.72	0.06	0.15	0.45	0.79	0.04	0.06	0.12	0.22	0.12	0.48	0.97	1.00
189.21	2083	234242	0.05	0.12	0.36	0.67	0.05	0.14	0.41	0.74	0.03	0.06	0.11	0.20	0.11	0.44	0.95	1.00
191	702	234242	0.04	0.07	0.15	0.28	0.04	0.07	0.17	0.33	0.03	0.04	0.06	0.09	0.06	0.18	0.56	0.89
191.1	579	234242	0.04	0.06	0.13	0.24	0.04	0.07	0.15	0.28	0.03	0.04	0.06	0.08	0.06	0.16	0.48	0.82
191.11	539	234242	0.04	0.06	0.13	0.23	0.04	0.06	0.14	0.26	0.03	0.04	0.06	0.08	0.06	0.15	0.46	0.79
193	345	234242	0.03	0.05	0.10	0.16	0.03	0.05	0.11	0.19	0.03	0.04	0.05	0.07	0.05	0.11	0.32	0.60
195	17637	234242	0.14	0.61	0.99	1.00	0.16	0.69	1.00	1.00	0.06	0.18	0.55	0.88	0.53	1.00	1.00	1.00
195.1	16966	234242	0.14	0.60	0.99	1.00	0.16	0.67	1.00	1.00	0.06	0.17	0.53	0.87	0.52	1.00	1.00	1.00
196	12225	234242	0.11	0.47	0.97	1.00	0.13	0.54	0.98	1.00	0.05	0.14	0.42	0.75	0.41	0.99	1.00	1.00
197	30162	234242	0.21	0.82	1.00	1.00	0.24	0.88	1.00	1.00	0.08	0.26	0.76	0.98	0.74	1.00	1.00	1.00
198	10103	234242	0.10	0.41	0.93	1.00	0.11	0.47	0.96	1.00	0.05	0.12	0.36	0.67	0.35	0.98	1.00	1.00
198.1	5504	234242	0.07	0.25	0.73	0.97	0.08	0.29	0.80	0.99	0.04	0.09	0.22	0.43	0.22	0.83	1.00	1.00
198.2	2544	234242	0.05	0.14	0.42	0.75	0.06	0.16	0.48	0.82	0.04	0.06	0.13	0.23	0.12	0.52	0.98	1.00
198.3	1757	234242	0.05	0.11	0.31	0.59	0.05	0.12	0.36	0.67	0.03	0.05	0.10	0.17	0.10	0.38	0.92	1.00
198.4	2970	234242	0.06	0.16	0.48	0.81	0.06	0.18	0.55	0.88	0.04	0.06	0.14	0.26	0.14	0.58	0.99	1.00
198.5	927	234242	0.04	0.08	0.19	0.36	0.04	0.08	0.21	0.41	0.03	0.04	0.07	0.11	0.07	0.23	0.68	0.95
198.6	2454	234242	0.05	0.14	0.41	0.74	0.06	0.15	0.47	0.81	0.04	0.06	0.12	0.22	0.12	0.50	0.98	1.00
198.7	201	234242	0.03	0.04	0.07	0.11	0.03	0.05	0.08	0.13	0.03	0.03	0.04	0.05	0.04	0.08	0.20	0.40
199	1504	234242	0.05	0.10	0.27	0.53	0.05	0.11	0.32	0.60	0.03	0.05	0.09	0.15	0.09	0.34	0.87	1.00
200	1054	234242	0.04	0.08	0.20	0.40	0.04	0.09	0.24	0.46	0.03	0.04	0.08	0.12	0.07	0.25	0.73	0.97
200.1	408	232957	0.03	0.05	0.11	0.18	0.04	0.06	0.12	0.21	0.03	0.04	0.05	0.07	0.05	0.12	0.36	0.68
201	252	234242	0.03	0.05	0.08	0.13	0.03	0.05	0.09	0.15	0.03	0.03	0.04	0.06	0.04	0.09	0.24	0.48
202	2358	234242	0.05	0.13	0.39	0.72	0.06	0.15	0.46	0.79	0.04	0.06	0.12	0.22	0.12	0.49	0.97	1.00
202.2	1853	234242	0.05	0.11	0.32	0.62	0.05	0.13	0.38	0.69	0.03	0.05	0.10	0.18	0.10	0.40	0.93	1.00
202.21	358	234242	0.03	0.05	0.10	0.17	0.03	0.06	0.11	0.19	0.03	0.04	0.05	0.07	0.05	0.11	0.33	0.62
202.24	623	234242	0.04	0.06	0.14	0.26	0.04	0.07	0.16	0.30	0.03	0.04	0.06	0.09	0.06	0.17	0.51	0.85
204	1774	234242	0.05	0.11	0.31	0.60	0.05	0.12	0.36	0.67	0.03	0.05	0.10	0.17	0.10	0.39	0.92	1.00
204.1	612	234242	0.04	0.06	0.14	0.25	0.04	0.07	0.16	0.29	0.03	0.04	0.06	0.09	0.06	0.17	0.50	0.84
204.12	540	234242	0.04	0.06	0.13	0.23	0.04	0.06	0.14	0.27	0.03	0.04	0.06	0.08	0.06	0.15	0.46	0.79
204.2	507	234242	0.04	0.06	0.12	0.22	0.04	0.06	0.14	0.25	0.03	0.04	0.06	0.08	0.05	0.14	0.43	0.77
204.21	353	234242	0.03	0.05	0.10	0.17	0.03	0.05	0.11	0.19	0.03	0.04	0.05	0.07	0.05	0.11	0.32	0.61
204.4	593	234242	0.04	0.06	0.13	0.25	0.04	0.07	0.15	0.29	0.03	0.04	0.06	0.09	0.06	0.16	0.49	0.83
208	20688	234242	0.16	0.68	1.00	1.00	0.18	0.75	1.00	1.00	0.07	0.20	0.61	0.92	0.60	1.00	1.00	1.00
210	911	234242	0.04	0.08	0.18	0.35	0.04	0.08	0.21	0.41	0.03	0.04	0.07	0.11	0.07	0.22	0.67	0.95
211	5805	234242	0.08	0.26	0.75	0.98	0.08	0.30	0.82	0.99	0.04	0.09	0.23	0.45	0.22	0.85	1.00	1.00
212	341	234242	0.03	0.05	0.09	0.16	0.03	0.05	0.11	0.18	0.03	0.04	0.05	0.07	0.05	0.11	0.31	0.60
213	284	234242	0.03	0.05	0.09	0.14	0.03	0.05	0.09	0.16	0.03	0.03	0.05	0.06	0.05	0.10	0.27	0.52
214	5942	234242	0.08	0.27	0.76	0.98	0.08	0.31	0.83	0.99	0.04	0.09	0.24	0.46	0.23	0.86	1.00	1.00
214.1	4303	234242	0.07	0.21	0.62	0.93	0.07	0.24	0.70	0.96	0.04	0.08	0.18	0.35	0.18	0.74	1.00	1.00
215	1015	234242	0.04	0.08	0.20	0.39	0.04	0.09	0.23	0.45	0.03	0.04	0.07	0.12	0.07	0.24	0.72	0.97
216	8174	234242	0.09	0.34	0.88	1.00	0.10	0.40	0.93	1.00	0.05	0.11	0.30	0.58	0.29	0.94	1.00	1.00
217	592	234242	0.04	0.06	0.13	0.25	0.04	0.07	0.15	0.29	0.03	0.04	0.06	0.09	0.06	0.16	0.49	0.83
217.1	548	234242	0.04	0.06	0.13	0.23	0.04	0.07	0.14	0.27	0.03	0.04	0.06	0.08	0.06	0.15	0.46	0.80
218	9233	122251	0.10	0.37	0.90	1.00	0.11	0.43	0.94	1.00	0.05	0.11	0.33	0.62	0.32	0.96	1.00	1.00
218.1	9007	122251	0.09	0.36	0.89	1.00	0.10	0.42	0.94	1.00	0.05	0.11	0.32	0.61	0.31	0.96	1.00	1.00
218.2	304	122251	0.03	0.05	0.09	0.15	0.03	0.05	0.10	0.17	0.03	0.03	0.05	0.06	0.05	0.10	0.28	0.55
220	1320	122251	0.04	0.09	0.24	0.48	0.05	0.10	0.28	0.55	0.03	0.05	0.09	0.14	0.08	0.30	0.82	0.99
225	818	234242	0.04	0.07	0.17	0.32	0.04	0.08	0.19	0.37	0.03	0.04	0.07	0.10	0.07	0.21	0.63	0.93
225.1	764	234242	0.04	0.07	0.16	0.31	0.04	0.08	0.18	0.35	0.03	0.04	0.07	0.10	0.06	0.20	0.60	0.91
226	260	234242	0.03	0.05	0.08	0.13	0.03	0.05	0.09	0.15	0.03	0.03	0.04	0.06	0.04	0.09	0.25	0.49
227	895	234242	0.04	0.08	0.18	0.35	0.04	0.08	0.21	0.40	0.03	0.04	0.07	0.11	0.07	0.22	0.66	0.95
227.2	377	234242	0.03	0.05	0.10	0.17	0.04	0.06	0.11	0.20	0.03	0.04	0.05	0.07	0.05	0.12	0.34	0.64

227.3	319	234242	0.03	0.05	0.09	0.15	0.03	0.05	0.10	0.17	0.03	0.03	0.05	0.06	0.05	0.11	0.30	0.57
228	1487	234242	0.05	0.10	0.27	0.52	0.05	0.11	0.31	0.60	0.03	0.05	0.09	0.15	0.09	0.34	0.87	1.00
229	2113	234242	0.05	0.12	0.36	0.67	0.05	0.14	0.42	0.75	0.04	0.06	0.11	0.20	0.11	0.45	0.95	1.00
240	542	261466	0.04	0.06	0.13	0.23	0.04	0.06	0.14	0.27	0.03	0.04	0.06	0.08	0.06	0.15	0.46	0.80
241	1539	261466	0.05	0.10	0.28	0.54	0.05	0.11	0.32	0.61	0.03	0.05	0.09	0.16	0.09	0.35	0.88	1.00
241.1	478	261466	0.04	0.06	0.12	0.21	0.04	0.06	0.13	0.24	0.03	0.04	0.05	0.08	0.05	0.14	0.41	0.74
241.2	631	261466	0.04	0.06	0.14	0.26	0.04	0.07	0.16	0.30	0.03	0.04	0.06	0.09	0.06	0.17	0.52	0.85
242	1795	261466	0.05	0.11	0.32	0.60	0.05	0.12	0.37	0.68	0.03	0.05	0.10	0.18	0.10	0.39	0.92	1.00
242.1	439	261466	0.03	0.06	0.11	0.20	0.04	0.06	0.12	0.22	0.03	0.04	0.05	0.07	0.05	0.13	0.39	0.71
242.3	210	261466	0.03	0.04	0.07	0.12	0.03	0.05	0.08	0.13	0.03	0.03	0.04	0.05	0.04	0.08	0.21	0.41
244	14603	261466	0.13	0.54	0.98	1.00	0.14	0.61	0.99	1.00	0.06	0.16	0.48	0.82	0.47	1.00	1.00	1.00
244.1	1144	261466	0.04	0.09	0.22	0.43	0.04	0.09	0.25	0.49	0.03	0.05	0.08	0.13	0.08	0.27	0.77	0.98
244.4	13923	261466	0.12	0.52	0.98	1.00	0.14	0.60	0.99	1.00	0.06	0.15	0.46	0.80	0.45	1.00	1.00	1.00
245	282	261466	0.03	0.05	0.09	0.14	0.03	0.05	0.09	0.16	0.03	0.03	0.05	0.06	0.05	0.10	0.27	0.52
245.2	215	261466	0.03	0.04	0.07	0.12	0.03	0.05	0.08	0.13	0.03	0.03	0.04	0.05	0.04	0.08	0.21	0.42
245.21	202	261466	0.03	0.04	0.07	0.11	0.03	0.05	0.08	0.13	0.03	0.03	0.04	0.05	0.04	0.08	0.20	0.40
246	386	261466	0.03	0.05	0.10	0.18	0.04	0.06	0.11	0.20	0.03	0.04	0.05	0.07	0.05	0.12	0.35	0.65
250	19441	261466	0.15	0.65	1.00	1.00	0.18	0.73	1.00	1.00	0.06	0.19	0.59	0.91	0.57	1.00	1.00	1.00
250.1	2582	261466	0.05	0.14	0.43	0.76	0.06	0.16	0.49	0.83	0.04	0.06	0.13	0.23	0.12	0.52	0.98	1.00
250.11	212	261466	0.03	0.04	0.07	0.12	0.03	0.05	0.08	0.13	0.03	0.03	0.04	0.05	0.04	0.08	0.21	0.41
250.13	451	261466	0.04	0.06	0.11	0.20	0.04	0.06	0.13	0.23	0.03	0.04	0.05	0.07	0.05	0.13	0.40	0.72
250.2	18758	261466	0.15	0.64	1.00	1.00	0.17	0.72	1.00	1.00	0.06	0.19	0.58	0.90	0.56	1.00	1.00	1.00
250.22	213	261466	0.03	0.04	0.07	0.12	0.03	0.05	0.08	0.13	0.03	0.03	0.04	0.05	0.04	0.08	0.21	0.42
250.23	1355	261466	0.04	0.09	0.25	0.49	0.05	0.10	0.29	0.56	0.03	0.05	0.09	0.14	0.08	0.31	0.83	0.99
250.24	598	261466	0.04	0.06	0.13	0.25	0.04	0.07	0.15	0.29	0.03	0.04	0.06	0.09	0.06	0.16	0.50	0.83
250.4	678	261466	0.04	0.07	0.15	0.28	0.04	0.07	0.17	0.32	0.03	0.04	0.06	0.09	0.06	0.18	0.55	0.88
250.41	288	261466	0.03	0.05	0.09	0.14	0.03	0.05	0.10	0.16	0.03	0.03	0.05	0.06	0.05	0.10	0.27	0.53
250.42	391	261466	0.03	0.05	0.10	0.18	0.04	0.06	0.11	0.20	0.03	0.04	0.05	0.07	0.05	0.12	0.35	0.66
250.6	408	261466	0.03	0.05	0.11	0.18	0.04	0.06	0.12	0.21	0.03	0.04	0.05	0.07	0.05	0.12	0.36	0.68
250.7	1398	254478	0.04	0.10	0.26	0.50	0.05	0.11	0.30	0.57	0.03	0.05	0.09	0.15	0.09	0.32	0.85	0.99
251.1	1024	261466	0.04	0.08	0.20	0.39	0.04	0.09	0.23	0.45	0.03	0.04	0.07	0.12	0.07	0.25	0.72	0.97
252	940	261466	0.04	0.08	0.19	0.36	0.04	0.08	0.22	0.42	0.03	0.04	0.07	0.11	0.07	0.23	0.69	0.96
252.1	830	261466	0.04	0.07	0.17	0.33	0.04	0.08	0.20	0.38	0.03	0.04	0.07	0.10	0.07	0.21	0.63	0.93
253	697	261466	0.04	0.07	0.15	0.28	0.04	0.07	0.17	0.33	0.03	0.04	0.06	0.09	0.06	0.18	0.56	0.88
253.2	261	261466	0.03	0.05	0.08	0.13	0.03	0.05	0.09	0.15	0.03	0.03	0.04	0.06	0.04	0.09	0.25	0.49
255	636	261466	0.04	0.06	0.14	0.26	0.04	0.07	0.16	0.30	0.03	0.04	0.06	0.09	0.06	0.17	0.52	0.85
255.2	363	261466	0.03	0.05	0.10	0.17	0.03	0.06	0.11	0.19	0.03	0.04	0.05	0.07	0.05	0.12	0.33	0.63
255.21	363	261466	0.03	0.05	0.10	0.17	0.03	0.06	0.11	0.19	0.03	0.04	0.05	0.07	0.05	0.12	0.33	0.63
256	241	143689	0.03	0.05	0.08	0.13	0.03	0.05	0.09	0.14	0.03	0.03	0.04	0.06	0.04	0.09	0.24	0.46
256.4	200	143689	0.03	0.04	0.07	0.11	0.03	0.05	0.08	0.13	0.03	0.03	0.04	0.05	0.04	0.08	0.20	0.39
260	275	261466	0.03	0.05	0.08	0.14	0.03	0.05	0.09	0.16	0.03	0.03	0.05	0.06	0.05	0.10	0.26	0.51
260.6	885	261466	0.04	0.07	0.18	0.35	0.04	0.08	0.21	0.40	0.03	0.04	0.07	0.11	0.07	0.22	0.66	0.94
261	1684	261466	0.05	0.11	0.30	0.58	0.05	0.12	0.35	0.65	0.03	0.05	0.10	0.17	0.10	0.37	0.90	1.00
261.2	911	261466	0.04	0.08	0.18	0.35	0.04	0.08	0.21	0.41	0.03	0.04	0.07	0.11	0.07	0.22	0.67	0.95
261.4	788	261466	0.04	0.07	0.16	0.31	0.04	0.08	0.19	0.36	0.03	0.04	0.07	0.10	0.07	0.20	0.61	0.92
262	298	261466	0.03	0.05	0.09	0.15	0.03	0.05	0.10	0.17	0.03	0.03	0.05	0.06	0.05	0.10	0.28	0.54
269	324	261466	0.03	0.05	0.09	0.16	0.03	0.05	0.10	0.18	0.03	0.03	0.05	0.06	0.05	0.11	0.30	0.58
270	365	261466	0.03	0.05	0.10	0.17	0.03	0.06	0.11	0.19	0.03	0.04	0.05	0.07	0.05	0.12	0.33	0.63
270.3	321	261466	0.03	0.05	0.09	0.15	0.03	0.05	0.10	0.18	0.03	0.03	0.05	0.06	0.05	0.11	0.30	0.57
270.32	246	261466	0.03	0.05	0.08	0.13	0.03	0.05	0.09	0.14	0.03	0.03	0.04	0.06	0.04	0.09	0.24	0.47
272	35143	261466	0.23	0.87	1.00	1.00	0.27	0.92	1.00	1.00	0.08	0.30	0.82	0.99	0.80	1.00	1.00	1.00
272.1	35033	261466	0.23	0.87	1.00	1.00	0.27	0.92	1.00	1.00	0.08	0.30	0.82	0.99	0.80	1.00	1.00	1.00
272.11	32554	261466	0.22	0.85	1.00	1.00	0.26	0.90	1.00	1.00	0.08	0.28	0.79	0.99	0.78	1.00	1.00	1.00
272.9	251	261466	0.03	0.05	0.08	0.13	0.03	0.05	0.09	0.15	0.03	0.03	0.04	0.06	0.04	0.09	0.24	0.47
274	4155	261466	0.07	0.20	0.61	0.92	0.07	0.23	0.69	0.96	0.04	0.07	0.18	0.34	0.17	0.72	1.00	1.00
274.1	3587	261466	0.06	0.18	0.55	0.88	0.07	0.21	0.62	0.93	0.04	0.07	0.16	0.31	0.16	0.66	1.00	1.00
274.2	628	261466	0.04	0.06	0.14	0.26	0.04	0.07	0.16	0.30	0.03	0.04	0.06	0.09	0.06	0.17	0.52	0.85
274.21	557	261466	0.04	0.06	0.13	0.24	0.04	0.07	0.15	0.27	0.03	0.04	0.06	0.08	0.06	0.15	0.47	0.81
275	2470	261466	0.05	0.14	0.41	0.74	0.06	0.16	0.47	0.81	0.04	0.06	0.12	0.23	0.12	0.51	0.98	1.00
275.1	661	309446	0.04	0.07	0.14	0.27	0.04	0.07	0.16	0.31	0.03	0.04	0.06	0.09	0.06	0.17	0.54	0.87
275.3	543	261466	0.04	0.06	0.13	0.23	0.04	0.06	0.14	0.27	0.03	0.04	0.06	0.08	0.06	0.15	0.46	0.80
275.5	1454	261466	0.05	0.10	0.27	0.52	0.05	0.11	0.31	0.59	0.03	0.05	0.09	0.15	0.09	0.33	0.86	1.00
275.53	273	261466	0.03	0.05	0.08	0.14	0.03	0.05	0.09	0.16	0.03	0.03	0.05	0.06	0.04	0.10	0.26	0.51
276	7916	261466	0.09	0.34	0.87	1.00	0.10	0.39	0.92	1.00	0.05	0.11	0.30	0.57	0.29	0.94	1.00	1.00
276.1	5081	261466	0.07	0.23	0.70	0.96	0.08	0.27	0.77	0.98	0.04	0.08	0.21	0.41	0.20	0.80	1.00	1.00
276.11	323	261466	0.03	0.05	0.09	0.15	0.03	0.05	0.10	0.18	0.03	0.03	0.05	0.06	0.05	0.11	0.30	0.58
276.12	2295	261466	0.05	0.13	0.39	0.71	0.06	0.15	0.45	0.78	0.04	0.06	0.12	0.21	0.12	0.48	0.97	1.00
276.13	1176	261466	0.04	0.09	0.22	0.44	0.04	0.10	0.26	0.50	0.03	0.05	0.08	0.13	0.08	0.28	0.78	0.98
276.14	1781	261466	0.05	0.11	0.31	0.60	0.05	0.12	0.36	0.68	0.03	0.05	0.10	0.18	0.10	0.39	0.92	1.00
276.4	1421	261466	0.04	0.10	0.26	0.51	0.05	0.11	0.30	0.58	0.03	0.05	0.09	0.15	0.09	0.32	0.85	0.99
276.41	1278	261466	0.04	0.09	0.24	0.47	0.05	0.10	0.28	0.54	0.03	0.05	0.08	0.14	0.08	0.30	0.81	0.99
276.5	3349	261466	0.06	0.17	0.52	0.86	0.06	0.20	0.60	0.91	0.04	0.07	0.15	0.29	0.15	0.63	1.00	1.00
276.6	615	261466	0.04	0.06	0.14	0.26	0.04	0.07	0.16	0.30	0.03	0.04	0.06	0.09	0.06	0.17	0.51	0.84
277	1034	261466	0.04	0.08	0.20	0.39	0.04	0.09	0.23	0.45	0.03	0.04	0.08	0.12	0.07	0.25	0.73	0.97

277.4	383	261466	0.03	0.05	0.10	0.18	0.04	0.06	0.11	0.20	0.03	0.04	0.05	0.07	0.05	0.12	0.35	0.65
277.5	256	261466	0.03	0.05	0.08	0.13	0.03	0.05	0.09	0.15	0.03	0.03	0.04	0.06	0.04	0.09	0.25	0.48
278	12786	261466	0.12	0.49	0.97	1.00	0.13	0.56	0.99	1.00	0.05	0.14	0.44	0.77	0.42	0.99	1.00	1.00
278.1	12646	261466	0.12	0.49	0.97	1.00	0.13	0.56	0.99	1.00	0.05	0.14	0.43	0.76	0.42	0.99	1.00	1.00
279	271	261466	0.03	0.05	0.08	0.14	0.03	0.05	0.09	0.16	0.03	0.03	0.05	0.06	0.04	0.10	0.26	0.50
279.1	223	261466	0.03	0.04	0.08	0.12	0.03	0.05	0.08	0.14	0.03	0.03	0.04	0.06	0.04	0.09	0.22	0.43
279.7	239	261466	0.03	0.05	0.08	0.13	0.03	0.05	0.09	0.14	0.03	0.03	0.04	0.06	0.04	0.09	0.23	0.46
280	8403	310388	0.09	0.35	0.89	1.00	0.10	0.41	0.93	1.00	0.05	0.11	0.31	0.60	0.30	0.95	1.00	1.00
280.1	8022	310388	0.09	0.34	0.87	1.00	0.10	0.39	0.92	1.00	0.05	0.11	0.30	0.58	0.29	0.94	1.00	1.00
280.2	436	310388	0.03	0.06	0.11	0.19	0.04	0.06	0.12	0.22	0.03	0.04	0.05	0.07	0.05	0.13	0.38	0.70
281	1150	310388	0.04	0.09	0.22	0.43	0.04	0.09	0.25	0.49	0.03	0.05	0.08	0.13	0.08	0.27	0.77	0.98
281.1	1093	310388	0.04	0.08	0.21	0.41	0.04	0.09	0.24	0.47	0.03	0.05	0.08	0.12	0.08	0.26	0.75	0.98
281.11	769	310388	0.04	0.07	0.16	0.31	0.04	0.08	0.18	0.36	0.03	0.04	0.07	0.10	0.06	0.20	0.60	0.91
281.13	200	310388	0.03	0.04	0.07	0.11	0.03	0.05	0.08	0.13	0.03	0.03	0.04	0.05	0.04	0.08	0.20	0.39
284	480	310388	0.04	0.06	0.12	0.21	0.04	0.06	0.13	0.24	0.03	0.04	0.05	0.08	0.05	0.14	0.42	0.75
285	12234	310388	0.12	0.48	0.97	1.00	0.13	0.55	0.99	1.00	0.05	0.14	0.42	0.75	0.41	0.99	1.00	1.00
285.1	237	310388	0.03	0.05	0.08	0.12	0.03	0.05	0.09	0.14	0.03	0.03	0.04	0.06	0.04	0.09	0.23	0.45
285.2	736	310388	0.04	0.07	0.16	0.30	0.04	0.07	0.18	0.34	0.03	0.04	0.06	0.10	0.06	0.19	0.58	0.90
285.22	433	310388	0.03	0.06	0.11	0.19	0.04	0.06	0.12	0.22	0.03	0.04	0.05	0.07	0.05	0.13	0.38	0.70
286	1023	310388	0.04	0.08	0.20	0.39	0.04	0.09	0.23	0.45	0.03	0.04	0.07	0.12	0.07	0.25	0.72	0.97
286.1	442	310388	0.03	0.06	0.11	0.20	0.04	0.06	0.12	0.23	0.03	0.04	0.05	0.07	0.05	0.13	0.39	0.71
286.12	278	310388	0.03	0.05	0.08	0.14	0.03	0.05	0.09	0.16	0.03	0.03	0.05	0.06	0.05	0.10	0.26	0.51
286.7	411	310388	0.03	0.05	0.11	0.19	0.04	0.06	0.12	0.21	0.03	0.04	0.05	0.07	0.05	0.12	0.37	0.68
287	1804	310388	0.05	0.11	0.32	0.61	0.05	0.13	0.37	0.68	0.03	0.05	0.10	0.18	0.10	0.39	0.92	1.00
287.3	1667	310388	0.05	0.11	0.30	0.57	0.05	0.12	0.34	0.65	0.03	0.05	0.10	0.17	0.10	0.37	0.90	1.00
287.31	415	310388	0.03	0.05	0.11	0.19	0.04	0.06	0.12	0.21	0.03	0.04	0.05	0.07	0.05	0.13	0.37	0.68
288	697	310388	0.04	0.07	0.15	0.28	0.04	0.07	0.17	0.33	0.03	0.04	0.06	0.09	0.06	0.18	0.56	0.88
288.1	3267	310388	0.06	0.17	0.51	0.85	0.06	0.19	0.59	0.90	0.04	0.07	0.15	0.28	0.15	0.62	1.00	1.00
288.11	3267	310388	0.06	0.17	0.51	0.85	0.06	0.19	0.59	0.90	0.04	0.07	0.15	0.28	0.15	0.62	1.00	1.00
289	954	310388	0.04	0.08	0.19	0.37	0.04	0.09	0.22	0.43	0.03	0.04	0.07	0.11	0.07	0.23	0.69	0.96
289.3	318	310388	0.03	0.05	0.09	0.15	0.03	0.05	0.10	0.17	0.03	0.03	0.05	0.06	0.05	0.11	0.30	0.57
289.4	2636	310388	0.05	0.14	0.43	0.77	0.06	0.16	0.50	0.84	0.04	0.06	0.13	0.24	0.13	0.53	0.98	1.00
289.5	514	310388	0.04	0.06	0.12	0.22	0.04	0.06	0.14	0.25	0.03	0.04	0.06	0.08	0.05	0.15	0.44	0.77
289.8	315	310063	0.03	0.05	0.09	0.15	0.03	0.05	0.10	0.17	0.03	0.03	0.05	0.06	0.05	0.11	0.29	0.57
290	2856	262912	0.06	0.15	0.46	0.80	0.06	0.17	0.53	0.86	0.04	0.06	0.14	0.25	0.13	0.56	0.99	1.00
290.1	1583	262912	0.05	0.10	0.28	0.55	0.05	0.11	0.33	0.62	0.03	0.05	0.09	0.16	0.09	0.35	0.89	1.00
290.11	666	262912	0.04	0.07	0.15	0.27	0.04	0.07	0.17	0.32	0.03	0.04	0.06	0.09	0.06	0.18	0.54	0.87
290.13	847	262912	0.04	0.07	0.17	0.33	0.04	0.08	0.20	0.39	0.03	0.04	0.07	0.11	0.07	0.21	0.64	0.94
290.16	361	262912	0.03	0.05	0.10	0.17	0.03	0.06	0.11	0.19	0.03	0.04	0.05	0.07	0.05	0.11	0.33	0.62
290.2	1157	262912	0.04	0.09	0.22	0.43	0.04	0.10	0.25	0.50	0.03	0.05	0.08	0.13	0.08	0.27	0.77	0.98
291	693	262912	0.04	0.07	0.15	0.28	0.04	0.07	0.17	0.33	0.03	0.04	0.06	0.09	0.06	0.18	0.56	0.88
291.8	484	262912	0.04	0.06	0.12	0.21	0.04	0.06	0.13	0.24	0.03	0.04	0.05	0.08	0.05	0.14	0.42	0.75
292	5218	262912	0.07	0.24	0.71	0.96	0.08	0.28	0.78	0.98	0.04	0.08	0.21	0.42	0.21	0.81	1.00	1.00
292.1	1626	262912	0.05	0.10	0.29	0.56	0.05	0.12	0.34	0.64	0.03	0.05	0.10	0.16	0.09	0.36	0.89	1.00
292.2	248	262912	0.03	0.05	0.08	0.13	0.03	0.05	0.09	0.15	0.03	0.03	0.04	0.06	0.04	0.09	0.24	0.47
292.3	730	262912	0.04	0.07	0.16	0.29	0.04	0.07	0.18	0.34	0.03	0.04	0.06	0.10	0.06	0.19	0.58	0.90
292.4	2527	262912	0.05	0.14	0.42	0.75	0.06	0.16	0.48	0.82	0.04	0.06	0.13	0.23	0.12	0.51	0.98	1.00
292.6	350	262912	0.03	0.05	0.10	0.16	0.03	0.05	0.11	0.19	0.03	0.04	0.05	0.07	0.05	0.11	0.32	0.61
293	1929	262912	0.05	0.12	0.33	0.63	0.05	0.13	0.39	0.71	0.03	0.05	0.11	0.19	0.10	0.42	0.94	1.00
293.1	832	262912	0.04	0.07	0.17	0.33	0.04	0.08	0.20	0.38	0.03	0.04	0.07	0.10	0.07	0.21	0.63	0.93
295	793	262912	0.04	0.07	0.16	0.31	0.04	0.08	0.19	0.37	0.03	0.04	0.07	0.10	0.07	0.20	0.61	0.92
295.1	513	262912	0.04	0.06	0.12	0.22	0.04	0.06	0.14	0.25	0.03	0.04	0.06	0.08	0.05	0.15	0.44	0.77
296	13263	262912	0.12	0.50	0.98	1.00	0.14	0.58	0.99	1.00	0.06	0.15	0.45	0.78	0.43	0.99	1.00	1.00
296.1	1015	262912	0.04	0.08	0.20	0.39	0.04	0.09	0.23	0.45	0.03	0.04	0.07	0.12	0.07	0.24	0.72	0.97
296.2	12701	262912	0.12	0.49	0.97	1.00	0.13	0.56	0.99	1.00	0.05	0.14	0.43	0.77	0.42	0.99	1.00	1.00
296.22	12629	262912	0.12	0.49	0.97	1.00	0.13	0.56	0.99	1.00	0.05	0.14	0.43	0.76	0.42	0.99	1.00	1.00
297	1529	262912	0.05	0.10	0.28	0.54	0.05	0.11	0.32	0.61	0.03	0.05	0.09	0.16	0.09	0.34	0.88	1.00
297.2	1457	262912	0.05	0.10	0.27	0.52	0.05	0.11	0.31	0.59	0.03	0.05	0.09	0.15	0.09	0.33	0.86	1.00
300	8295	262912	0.09	0.35	0.88	1.00	0.10	0.40	0.93	1.00	0.05	0.11	0.31	0.59	0.30	0.95	1.00	1.00
300.1	7701	262912	0.09	0.33	0.86	1.00	0.10	0.38	0.91	1.00	0.05	0.10	0.29	0.56	0.28	0.93	1.00	1.00
300.12	820	262912	0.04	0.07	0.17	0.32	0.04	0.08	0.19	0.38	0.03	0.04	0.07	0.10	0.07	0.21	0.63	0.93
300.13	649	262912	0.04	0.07	0.14	0.27	0.04	0.07	0.16	0.31	0.03	0.04	0.06	0.09	0.06	0.17	0.53	0.86
301	338	262912	0.03	0.05	0.09	0.16	0.03	0.05	0.10	0.18	0.03	0.04	0.05	0.07	0.05	0.11	0.31	0.59
302	279	262912	0.03	0.05	0.08	0.14	0.03	0.05	0.09	0.16	0.03	0.03	0.05	0.06	0.05	0.10	0.27	0.52
303	457	262912	0.04	0.06	0.11	0.20	0.04	0.06	0.13	0.23	0.03	0.04	0.05	0.08	0.05	0.13	0.40	0.73
303.3	339	262912	0.03	0.05	0.09	0.16	0.03	0.05	0.10	0.18	0.03	0.04	0.05	0.07	0.05	0.11	0.31	0.60
304	258	262912	0.03	0.05	0.08	0.13	0.03	0.05	0.09	0.15	0.03	0.03	0.04	0.06	0.04	0.09	0.25	0.49
306	39468	262912	0.25	0.90	1.00	1.00	0.29	0.94	1.00	1.00	0.09	0.33	0.85	0.99	0.84	1.00	1.00	1.00
306.9	280	262912	0.03	0.05	0.08	0.14	0.03	0.05	0.09	0.16	0.03	0.03	0.05	0.06	0.05	0.10	0.27	0.52
315	563	262912	0.04	0.06	0.13	0.24	0.04	0.07	0.15	0.27	0.03	0.04	0.06	0.08	0.06	0.16	0.47	0.81
316	412	262912	0.03	0.05	0.11	0.19	0.04	0.06	0.12	0.21	0.03	0.04	0.05	0.07	0.05	0.12	0.37	0.68
317	12721	262912	0.12	0.49	0.97	1.00	0.13	0.56	0.99	1.00	0.05	0.14	0.43	0.77	0.42	0.99	1.00	1.00
317.1	10886	262912	0.11	0.43	0.95	1.00	0.12	0.50	0.97	1.00	0.05	0.13	0.38	0.70	0.37	0.98	1.00	1.00
317.11	837	262912	0.04	0.07	0.17	0.33	0.04	0.08	0.20	0.38	0.03	0.04	0.07	0.10	0.07	0.21	0.64	0.93

318	18403	262912	0.15	0.63	1.00	1.00	0.17	0.71	1.00	1.00	0.06	0.19	0.57	0.89	0.55	1.00	1.00	1.00
320	391	295570	0.03	0.05	0.10	0.18	0.04	0.06	0.11	0.20	0.03	0.04	0.05	0.07	0.05	0.12	0.35	0.66
323	349	295570	0.03	0.05	0.10	0.16	0.03	0.05	0.11	0.19	0.03	0.04	0.05	0.07	0.05	0.11	0.32	0.61
324	305	295570	0.03	0.05	0.09	0.15	0.03	0.05	0.10	0.17	0.03	0.03	0.05	0.06	0.05	0.10	0.29	0.55
327	1210	295570	0.04	0.09	0.23	0.45	0.05	0.10	0.26	0.51	0.03	0.05	0.08	0.13	0.08	0.28	0.79	0.99
327.3	4435	295570	0.07	0.21	0.64	0.94	0.07	0.24	0.72	0.97	0.04	0.08	0.19	0.36	0.18	0.75	1.00	1.00
331	1287	295570	0.04	0.09	0.24	0.47	0.05	0.10	0.28	0.54	0.03	0.05	0.08	0.14	0.08	0.30	0.82	0.99
331.1	515	295570	0.04	0.06	0.12	0.22	0.04	0.06	0.14	0.26	0.03	0.04	0.06	0.08	0.06	0.15	0.44	0.77
331.9	514	295570	0.04	0.06	0.12	0.22	0.04	0.06	0.14	0.25	0.03	0.04	0.06	0.08	0.05	0.15	0.44	0.77
332	1345	295570	0.04	0.09	0.25	0.49	0.05	0.10	0.29	0.56	0.03	0.05	0.09	0.14	0.08	0.31	0.83	0.99
333	1094	295570	0.04	0.08	0.21	0.41	0.04	0.09	0.24	0.47	0.03	0.05	0.08	0.12	0.08	0.26	0.75	0.98
333.1	271	295570	0.03	0.05	0.08	0.14	0.03	0.05	0.09	0.16	0.03	0.03	0.05	0.06	0.04	0.10	0.26	0.50
333.4	265	295570	0.03	0.05	0.08	0.13	0.03	0.05	0.09	0.15	0.03	0.03	0.05	0.06	0.04	0.10	0.25	0.50
334	1896	295570	0.05	0.12	0.33	0.63	0.05	0.13	0.38	0.70	0.03	0.05	0.10	0.18	0.10	0.41	0.93	1.00
334.2	277	295570	0.03	0.05	0.08	0.14	0.03	0.05	0.09	0.16	0.03	0.03	0.05	0.06	0.05	0.10	0.26	0.51
335	1222	295570	0.04	0.09	0.23	0.45	0.05	0.10	0.27	0.52	0.03	0.05	0.08	0.13	0.08	0.29	0.80	0.99
337	355	295570	0.03	0.05	0.10	0.17	0.03	0.05	0.11	0.19	0.03	0.04	0.05	0.07	0.05	0.11	0.32	0.62
338	742	295570	0.04	0.07	0.16	0.30	0.04	0.08	0.18	0.35	0.03	0.04	0.06	0.10	0.06	0.19	0.58	0.90
338.1	363	295570	0.03	0.05	0.10	0.17	0.03	0.06	0.11	0.19	0.03	0.04	0.05	0.07	0.05	0.12	0.33	0.63
338.2	387	295570	0.03	0.05	0.10	0.18	0.04	0.06	0.11	0.20	0.03	0.04	0.05	0.07	0.05	0.12	0.35	0.65
339	7243	295570	0.09	0.31	0.84	0.99	0.09	0.36	0.90	1.00	0.05	0.10	0.28	0.54	0.27	0.92	1.00	1.00
340	2962	295570	0.06	0.16	0.48	0.81	0.06	0.18	0.55	0.87	0.04	0.06	0.14	0.26	0.14	0.58	0.99	1.00
340.1	217	295570	0.03	0.04	0.07	0.12	0.03	0.05	0.08	0.13	0.03	0.03	0.04	0.05	0.04	0.09	0.22	0.42
341	221	295570	0.03	0.04	0.08	0.12	0.03	0.05	0.08	0.13	0.03	0.03	0.04	0.06	0.04	0.09	0.22	0.43
342	1600	295570	0.05	0.10	0.29	0.55	0.05	0.12	0.33	0.63	0.03	0.05	0.09	0.16	0.09	0.36	0.89	1.00
344	658	295570	0.04	0.07	0.14	0.27	0.04	0.07	0.16	0.31	0.03	0.04	0.06	0.09	0.06	0.17	0.53	0.87
345	4890	295570	0.07	0.23	0.68	0.95	0.08	0.26	0.76	0.98	0.04	0.08	0.20	0.39	0.20	0.79	1.00	1.00
345.1	877	295570	0.04	0.07	0.18	0.34	0.04	0.08	0.20	0.40	0.03	0.04	0.07	0.11	0.07	0.22	0.66	0.94
345.11	423	295570	0.03	0.05	0.11	0.19	0.04	0.06	0.12	0.22	0.03	0.04	0.05	0.07	0.05	0.13	0.37	0.69
345.12	273	295570	0.03	0.05	0.08	0.14	0.03	0.05	0.09	0.16	0.03	0.03	0.05	0.06	0.04	0.10	0.26	0.51
345.3	2204	295570	0.05	0.13	0.37	0.69	0.05	0.14	0.43	0.77	0.04	0.06	0.12	0.21	0.11	0.46	0.96	1.00
348	854	295570	0.04	0.07	0.17	0.34	0.04	0.08	0.20	0.39	0.03	0.04	0.07	0.11	0.07	0.21	0.64	0.94
348.2	280	295570	0.03	0.05	0.08	0.14	0.03	0.05	0.09	0.16	0.03	0.03	0.05	0.06	0.05	0.10	0.27	0.52
348.7	338	295570	0.03	0.05	0.09	0.16	0.03	0.05	0.10	0.18	0.03	0.04	0.05	0.07	0.05	0.11	0.31	0.59
348.8	267	295570	0.03	0.05	0.08	0.14	0.03	0.05	0.09	0.15	0.03	0.03	0.05	0.06	0.04	0.10	0.26	0.50
348.9	440	295570	0.03	0.06	0.11	0.20	0.04	0.06	0.12	0.22	0.03	0.04	0.05	0.07	0.05	0.13	0.39	0.71
349	403	295570	0.03	0.05	0.10	0.18	0.04	0.06	0.12	0.21	0.03	0.04	0.05	0.07	0.05	0.12	0.36	0.67
350	3488	295570	0.06	0.18	0.54	0.87	0.07	0.20	0.61	0.92	0.04	0.07	0.16	0.30	0.15	0.65	1.00	1.00
350.1	968	295570	0.04	0.08	0.19	0.37	0.04	0.09	0.22	0.43	0.03	0.04	0.07	0.11	0.07	0.24	0.70	0.96
350.2	2371	295570	0.05	0.13	0.40	0.72	0.06	0.15	0.46	0.80	0.04	0.06	0.12	0.22	0.12	0.49	0.97	1.00
350.3	368	295570	0.03	0.05	0.10	0.17	0.03	0.06	0.11	0.20	0.03	0.04	0.05	0.07	0.05	0.12	0.33	0.63
351	11404	295570	0.11	0.45	0.96	1.00	0.12	0.52	0.98	1.00	0.05	0.13	0.40	0.72	0.39	0.99	1.00	1.00
352	1378	295570	0.04	0.09	0.25	0.49	0.05	0.11	0.29	0.57	0.03	0.05	0.09	0.15	0.09	0.32	0.84	0.99
352.1	485	295570	0.04	0.06	0.12	0.21	0.04	0.06	0.13	0.24	0.03	0.04	0.05	0.08	0.05	0.14	0.42	0.75
352.2	842	295570	0.04	0.07	0.17	0.33	0.04	0.08	0.20	0.38	0.03	0.04	0.07	0.11	0.07	0.21	0.64	0.93
353	2069	295570	0.05	0.12	0.36	0.66	0.05	0.14	0.41	0.74	0.03	0.06	0.11	0.20	0.11	0.44	0.95	1.00
355	329	295570	0.03	0.05	0.09	0.16	0.03	0.05	0.10	0.18	0.03	0.03	0.05	0.06	0.05	0.11	0.30	0.58
357	1558	295570	0.05	0.10	0.28	0.54	0.05	0.11	0.33	0.62	0.03	0.05	0.09	0.16	0.09	0.35	0.88	1.00
358	210	295570	0.03	0.04	0.07	0.12	0.03	0.05	0.08	0.13	0.03	0.03	0.04	0.05	0.04	0.08	0.21	0.41
359	439	295570	0.03	0.06	0.11	0.20	0.04	0.06	0.12	0.22	0.03	0.04	0.05	0.07	0.05	0.13	0.39	0.71
359.2	337	295570	0.03	0.05	0.09	0.16	0.03	0.05	0.10	0.18	0.03	0.04	0.05	0.07	0.05	0.11	0.31	0.59
360	315	287331	0.03	0.05	0.09	0.15	0.03	0.05	0.10	0.17	0.03	0.03	0.05	0.06	0.05	0.11	0.29	0.57
360.2	227	287331	0.03	0.04	0.08	0.12	0.03	0.05	0.08	0.14	0.03	0.03	0.04	0.06	0.04	0.09	0.22	0.44
361	3118	287331	0.06	0.16	0.49	0.83	0.06	0.18	0.57	0.89	0.04	0.07	0.15	0.27	0.14	0.60	0.99	1.00
361.1	1401	287331	0.04	0.10	0.26	0.50	0.05	0.11	0.30	0.57	0.03	0.05	0.09	0.15	0.09	0.32	0.85	0.99
362	5021	286896	0.07	0.23	0.69	0.96	0.08	0.27	0.77	0.98	0.04	0.08	0.21	0.40	0.20	0.80	1.00	1.00
362.2	2640	286896	0.05	0.14	0.43	0.77	0.06	0.16	0.50	0.84	0.04	0.06	0.13	0.24	0.13	0.53	0.98	1.00
362.29	2637	286896	0.05	0.14	0.43	0.77	0.06	0.16	0.50	0.84	0.04	0.06	0.13	0.24	0.13	0.53	0.98	1.00
362.3	1059	286896	0.04	0.08	0.21	0.40	0.04	0.09	0.24	0.46	0.03	0.04	0.08	0.12	0.07	0.25	0.74	0.97
362.31	1003	286896	0.04	0.08	0.20	0.38	0.04	0.09	0.23	0.44	0.03	0.04	0.07	0.12	0.07	0.24	0.71	0.97
362.4	872	286896	0.04	0.07	0.18	0.34	0.04	0.08	0.20	0.39	0.03	0.04	0.07	0.11	0.07	0.22	0.65	0.94
364	751	287331	0.04	0.07	0.16	0.30	0.04	0.08	0.18	0.35	0.03	0.04	0.06	0.10	0.06	0.19	0.59	0.91
364.4	240	287331	0.03	0.05	0.08	0.13	0.03	0.05	0.09	0.14	0.03	0.03	0.04	0.06	0.04	0.09	0.23	0.46
364.5	236	287331	0.03	0.05	0.08	0.12	0.03	0.05	0.09	0.14	0.03	0.03	0.04	0.06	0.04	0.09	0.23	0.45
365	4986	287331	0.07	0.23	0.69	0.96	0.08	0.27	0.76	0.98	0.04	0.08	0.21	0.40	0.20	0.80	1.00	1.00
365.1	1216	287331	0.04	0.09	0.23	0.45	0.05	0.10	0.27	0.52	0.03	0.05	0.08	0.13	0.08	0.28	0.79	0.99
365.11	1212	287331	0.04	0.09	0.23	0.45	0.05	0.10	0.26	0.51	0.03	0.05	0.08	0.13	0.08	0.28	0.79	0.99
365.2	742	287331	0.04	0.07	0.16	0.30	0.04	0.08	0.18	0.35	0.03	0.04	0.06	0.10	0.06	0.19	0.58	0.90
366	22347	287331	0.17	0.71	1.00	1.00	0.20	0.79	1.00	1.00	0.07	0.22	0.65	0.94	0.63	1.00	1.00	1.00
366.2	9863	287331	0.10	0.40	0.93	1.00	0.11	0.46	0.96	1.00	0.05	0.12	0.36	0.66	0.34	0.97	1.00	1.00
367	2886	287331	0.06	0.15	0.47	0.80	0.06	0.17	0.53	0.87	0.04	0.06	0.14	0.26	0.13	0.57	0.99	1.00
367.1	1422	287331	0.04	0.10	0.26	0.51	0.05	0.11	0.30	0.58	0.03	0.05	0.09	0.15	0.09	0.32	0.85	0.99
367.2	225	287331	0.03	0.04	0.08	0.12	0.03	0.05	0.08	0.14	0.03	0.03	0.04	0.06	0.04	0.09	0.22	0.43
367.8	278	287331	0.03	0.05	0.08	0.14	0.03	0.05	0.09	0.16	0.03	0.03	0.05	0.06	0.05	0.10	0.26	0.51

367.9	950	287331	0.04	0.08	0.19	0.37	0.04	0.09	0.22	0.42	0.03	0.04	0.07	0.11	0.07	0.23	0.69	0.96
368	3085	287331	0.06	0.16	0.49	0.83	0.06	0.18	0.56	0.89	0.04	0.07	0.14	0.27	0.14	0.60	0.99	1.00
368.1	607	287331	0.04	0.06	0.14	0.25	0.04	0.07	0.15	0.29	0.03	0.04	0.06	0.09	0.06	0.16	0.50	0.84
368.2	723	287331	0.04	0.07	0.15	0.29	0.04	0.07	0.18	0.34	0.03	0.04	0.06	0.10	0.06	0.19	0.57	0.90
368.4	370	287331	0.03	0.05	0.10	0.17	0.03	0.06	0.11	0.20	0.03	0.04	0.05	0.07	0.05	0.12	0.34	0.63
368.9	635	287331	0.04	0.06	0.14	0.26	0.04	0.07	0.16	0.30	0.03	0.04	0.06	0.09	0.06	0.17	0.52	0.85
368.91	222	287331	0.03	0.04	0.08	0.12	0.03	0.05	0.08	0.13	0.03	0.03	0.04	0.06	0.04	0.09	0.22	0.43
369	574	287331	0.04	0.06	0.13	0.24	0.04	0.07	0.15	0.28	0.03	0.04	0.06	0.08	0.06	0.16	0.48	0.82
369.5	241	287331	0.03	0.05	0.08	0.13	0.03	0.05	0.09	0.14	0.03	0.03	0.04	0.06	0.04	0.09	0.24	0.46
370	283	287331	0.03	0.05	0.09	0.14	0.03	0.05	0.09	0.16	0.03	0.03	0.05	0.06	0.05	0.10	0.27	0.52
371	2760	287331	0.06	0.15	0.45	0.78	0.06	0.17	0.52	0.85	0.04	0.06	0.13	0.25	0.13	0.55	0.99	1.00
371.1	485	287331	0.04	0.06	0.12	0.21	0.04	0.06	0.13	0.24	0.03	0.04	0.05	0.08	0.05	0.14	0.42	0.75
371.3	2161	287331	0.05	0.13	0.37	0.68	0.05	0.14	0.43	0.76	0.04	0.06	0.11	0.20	0.11	0.46	0.96	1.00
372	731	287331	0.04	0.07	0.16	0.29	0.04	0.07	0.18	0.34	0.03	0.04	0.06	0.10	0.06	0.19	0.58	0.90
374	5327	287331	0.07	0.24	0.72	0.97	0.08	0.28	0.79	0.99	0.04	0.09	0.22	0.42	0.21	0.82	1.00	1.00
374.1	1101	287331	0.04	0.08	0.21	0.41	0.04	0.09	0.24	0.48	0.03	0.05	0.08	0.12	0.08	0.26	0.75	0.98
374.3	1725	287331	0.05	0.11	0.31	0.59	0.05	0.12	0.35	0.66	0.03	0.05	0.10	0.17	0.10	0.38	0.91	1.00
375	2085	287331	0.05	0.12	0.36	0.67	0.05	0.14	0.41	0.74	0.04	0.06	0.11	0.20	0.11	0.44	0.95	1.00
375.2	840	287331	0.04	0.07	0.17	0.33	0.04	0.08	0.20	0.38	0.03	0.04	0.07	0.10	0.07	0.21	0.64	0.93
377	438	287331	0.03	0.06	0.11	0.19	0.04	0.06	0.12	0.22	0.03	0.04	0.05	0.07	0.05	0.13	0.39	0.71
377.3	234	287331	0.03	0.05	0.08	0.12	0.03	0.05	0.08	0.14	0.03	0.03	0.04	0.06	0.04	0.09	0.23	0.45
378	1329	287331	0.04	0.09	0.25	0.48	0.05	0.10	0.29	0.55	0.03	0.05	0.09	0.14	0.08	0.31	0.83	0.99
378.1	836	287331	0.04	0.07	0.17	0.33	0.04	0.08	0.20	0.38	0.03	0.04	0.07	0.10	0.07	0.21	0.63	0.93
378.2	209	287331	0.03	0.04	0.07	0.12	0.03	0.05	0.08	0.13	0.03	0.03	0.04	0.05	0.04	0.08	0.21	0.41
378.5	264	287331	0.03	0.05	0.08	0.13	0.03	0.05	0.09	0.15	0.03	0.03	0.04	0.06	0.04	0.09	0.25	0.49
379	4107	287331	0.07	0.20	0.61	0.92	0.07	0.23	0.68	0.95	0.04	0.07	0.18	0.34	0.17	0.72	1.00	1.00
379.2	1443	287331	0.05	0.10	0.26	0.51	0.05	0.11	0.31	0.59	0.03	0.05	0.09	0.15	0.09	0.33	0.86	1.00
379.3	2166	287331	0.05	0.13	0.37	0.68	0.05	0.14	0.43	0.76	0.04	0.06	0.11	0.20	0.11	0.46	0.96	1.00
379.5	371	287331	0.03	0.05	0.10	0.17	0.03	0.06	0.11	0.20	0.03	0.04	0.05	0.07	0.05	0.12	0.34	0.63
380	614	287331	0.04	0.06	0.14	0.26	0.04	0.07	0.16	0.30	0.03	0.04	0.06	0.09	0.06	0.17	0.51	0.84
380.1	478	287331	0.04	0.06	0.12	0.21	0.04	0.06	0.13	0.24	0.03	0.04	0.05	0.08	0.05	0.14	0.41	0.74
380.4	323	287331	0.03	0.05	0.09	0.15	0.03	0.05	0.10	0.18	0.03	0.03	0.05	0.06	0.05	0.11	0.30	0.58
381	2003	287331	0.05	0.12	0.35	0.65	0.05	0.13	0.40	0.73	0.03	0.06	0.11	0.19	0.11	0.43	0.95	1.00
381.1	1613	287331	0.05	0.10	0.29	0.56	0.05	0.12	0.33	0.63	0.03	0.05	0.10	0.16	0.09	0.36	0.89	1.00
381.11	739	287331	0.04	0.07	0.16	0.30	0.04	0.07	0.18	0.34	0.03	0.04	0.06	0.10	0.06	0.19	0.58	0.90
381.9	242	287331	0.03	0.05	0.08	0.13	0.03	0.05	0.09	0.14	0.03	0.03	0.04	0.06	0.04	0.09	0.24	0.46
382	288	287331	0.03	0.05	0.09	0.14	0.03	0.05	0.10	0.16	0.03	0.03	0.05	0.06	0.05	0.10	0.27	0.53
383	275	287331	0.03	0.05	0.08	0.14	0.03	0.05	0.09	0.16	0.03	0.03	0.05	0.06	0.05	0.10	0.26	0.51
384	1161	287331	0.04	0.09	0.22	0.43	0.04	0.10	0.26	0.50	0.03	0.05	0.08	0.13	0.08	0.27	0.77	0.98
384.4	894	287331	0.04	0.08	0.18	0.35	0.04	0.08	0.21	0.40	0.03	0.04	0.07	0.11	0.07	0.22	0.66	0.95
385	768	287331	0.04	0.07	0.16	0.31	0.04	0.08	0.18	0.36	0.03	0.04	0.07	0.10	0.06	0.20	0.60	0.91
385.3	521	287331	0.04	0.06	0.12	0.22	0.04	0.06	0.14	0.26	0.03	0.04	0.06	0.08	0.06	0.15	0.44	0.78
386	1729	287331	0.05	0.11	0.31	0.59	0.05	0.12	0.35	0.66	0.03	0.05	0.10	0.17	0.10	0.38	0.91	1.00
386.1	559	287331	0.04	0.06	0.13	0.24	0.04	0.07	0.15	0.27	0.03	0.04	0.06	0.08	0.06	0.15	0.47	0.81
386.2	464	287331	0.04	0.06	0.11	0.20	0.04	0.06	0.13	0.23	0.03	0.04	0.05	0.08	0.05	0.14	0.40	0.73
386.3	710	287331	0.04	0.07	0.15	0.29	0.04	0.07	0.17	0.33	0.03	0.04	0.06	0.10	0.06	0.18	0.57	0.89
386.9	4685	287331	0.07	0.22	0.66	0.95	0.07	0.25	0.74	0.97	0.04	0.08	0.20	0.38	0.19	0.77	1.00	1.00
389	4810	287331	0.07	0.23	0.67	0.95	0.08	0.26	0.75	0.98	0.04	0.08	0.20	0.39	0.19	0.78	1.00	1.00
389.1	499	287331	0.04	0.06	0.12	0.22	0.04	0.06	0.13	0.25	0.03	0.04	0.06	0.08	0.05	0.14	0.43	0.76
389.2	412	287331	0.03	0.05	0.11	0.19	0.04	0.06	0.12	0.21	0.03	0.04	0.05	0.07	0.05	0.13	0.37	0.68
389.4	539	287331	0.04	0.06	0.13	0.23	0.04	0.06	0.14	0.27	0.03	0.04	0.06	0.08	0.06	0.15	0.46	0.79
394	5564	202269	0.07	0.25	0.73	0.97	0.08	0.29	0.80	0.99	0.04	0.09	0.22	0.43	0.22	0.84	1.00	1.00
394.1	680	202269	0.04	0.07	0.15	0.28	0.04	0.07	0.17	0.32	0.03	0.04	0.06	0.09	0.06	0.18	0.55	0.88
394.2	3323	202269	0.06	0.17	0.52	0.85	0.06	0.19	0.59	0.91	0.04	0.07	0.15	0.29	0.15	0.63	1.00	1.00
394.3	1453	202269	0.05	0.10	0.26	0.51	0.05	0.11	0.31	0.59	0.03	0.05	0.09	0.15	0.09	0.33	0.86	1.00
394.7	1335	202269	0.04	0.09	0.25	0.48	0.05	0.10	0.29	0.55	0.03	0.05	0.09	0.14	0.08	0.31	0.83	0.99
395	6317	202269	0.08	0.28	0.78	0.98	0.09	0.32	0.85	0.99	0.04	0.09	0.25	0.48	0.24	0.88	1.00	1.00
395.1	3224	202269	0.06	0.17	0.51	0.84	0.06	0.19	0.58	0.90	0.04	0.07	0.15	0.28	0.14	0.61	0.99	1.00
395.2	2915	202269	0.06	0.15	0.47	0.80	0.06	0.18	0.54	0.87	0.04	0.06	0.14	0.26	0.13	0.57	0.99	1.00
395.6	1554	202269	0.05	0.10	0.28	0.54	0.05	0.11	0.32	0.62	0.03	0.05	0.09	0.16	0.09	0.35	0.88	1.00
396	1382	202269	0.04	0.09	0.25	0.50	0.05	0.11	0.29	0.57	0.03	0.05	0.09	0.15	0.09	0.32	0.84	0.99
401	75408	202269	0.38	0.98	1.00	1.00	0.43	0.99	1.00	1.00	0.12	0.48	0.97	1.00	0.96	1.00	1.00	1.00
401.1	75160	202269	0.37	0.98	1.00	1.00	0.43	0.99	1.00	1.00	0.12	0.48	0.97	1.00	0.96	1.00	1.00	1.00
401.2	1667	202269	0.05	0.11	0.30	0.57	0.05	0.12	0.34	0.65	0.03	0.05	0.10	0.17	0.09	0.37	0.90	1.00
401.21	395	202269	0.03	0.05	0.10	0.18	0.04	0.06	0.12	0.21	0.03	0.04	0.05	0.07	0.05	0.12	0.35	0.66
401.22	1285	202269	0.04	0.09	0.24	0.47	0.05	0.10	0.28	0.54	0.03	0.05	0.08	0.14	0.08	0.30	0.81	0.99
402	1464	202269	0.05	0.10	0.27	0.52	0.05	0.11	0.31	0.59	0.03	0.05	0.09	0.15	0.09	0.33	0.86	1.00
411	30489	202269	0.21	0.81	1.00	1.00	0.24	0.88	1.00	1.00	0.08	0.26	0.75	0.98	0.74	1.00	1.00	1.00
411.1	4478	202269	0.07	0.21	0.64	0.94	0.07	0.24	0.72	0.97	0.04	0.08	0.19	0.37	0.18	0.75	1.00	1.00
411.2	12033	202269	0.11	0.46	0.96	1.00	0.13	0.53	0.98	1.00	0.05	0.14	0.41	0.74	0.40	0.99	1.00	1.00
411.3	15180	202269	0.13	0.55	0.99	1.00	0.15	0.62	1.00	1.00	0.06	0.16	0.49	0.83	0.48	1.00	1.00	1.00
411.4	19684	202269	0.15	0.65	1.00	1.00	0.18	0.73	1.00	1.00	0.06	0.19	0.59	0.90	0.57	1.00	1.00	1.00
411.41	735	202269	0.04	0.07	0.16	0.30	0.04	0.07	0.18	0.34	0.03	0.04	0.06	0.10	0.06	0.19	0.58	0.90
411.8	15139	202269	0.13	0.55	0.99	1.00	0.15	0.62	1.00	1.00	0.06	0.16	0.49	0.83	0.47	1.00	1.00	1.00

411.9	1423	202269	0.04	0.10	0.26	0.51	0.05	0.11	0.30	0.58	0.03	0.05	0.09	0.15	0.09	0.32	0.85	0.99
414	2245	202269	0.05	0.13	0.38	0.70	0.05	0.15	0.44	0.77	0.04	0.06	0.12	0.21	0.11	0.47	0.96	1.00
415	4937	202269	0.07	0.23	0.68	0.95	0.08	0.26	0.76	0.98	0.04	0.08	0.20	0.40	0.20	0.79	1.00	1.00
415.2	976	202269	0.04	0.08	0.19	0.37	0.04	0.09	0.22	0.43	0.03	0.04	0.07	0.12	0.07	0.24	0.70	0.96
415.21	414	202269	0.03	0.05	0.11	0.19	0.04	0.06	0.12	0.21	0.03	0.04	0.05	0.07	0.05	0.13	0.37	0.68
416	3070	202269	0.06	0.16	0.49	0.82	0.06	0.18	0.56	0.88	0.04	0.07	0.14	0.27	0.14	0.59	0.99	1.00
418	26417	202269	0.19	0.76	1.00	1.00	0.21	0.83	1.00	1.00	0.07	0.24	0.70	0.96	0.68	1.00	1.00	1.00
418.1	3619	202269	0.06	0.18	0.55	0.88	0.07	0.21	0.63	0.93	0.04	0.07	0.16	0.31	0.16	0.66	1.00	1.00
420	2276	202269	0.05	0.13	0.38	0.70	0.06	0.15	0.44	0.78	0.04	0.06	0.12	0.21	0.11	0.47	0.97	1.00
420.2	1431	202269	0.04	0.10	0.26	0.51	0.05	0.11	0.30	0.58	0.03	0.05	0.09	0.15	0.09	0.32	0.85	0.99
420.21	209	202269	0.03	0.04	0.07	0.11	0.03	0.05	0.08	0.13	0.03	0.03	0.04	0.05	0.04	0.08	0.21	0.41
420.3	764	202269	0.04	0.07	0.16	0.31	0.04	0.08	0.18	0.35	0.03	0.04	0.07	0.10	0.06	0.20	0.60	0.91
425	1363	202269	0.04	0.09	0.25	0.49	0.05	0.10	0.29	0.56	0.03	0.05	0.09	0.14	0.08	0.31	0.84	0.99
425.1	1307	202269	0.04	0.09	0.24	0.47	0.05	0.10	0.28	0.54	0.03	0.05	0.08	0.14	0.08	0.30	0.82	0.99
426	7651	202269	0.09	0.32	0.85	0.99	0.10	0.38	0.91	1.00	0.05	0.10	0.29	0.55	0.28	0.93	1.00	1.00
426.2	2459	202269	0.05	0.14	0.41	0.74	0.06	0.15	0.47	0.81	0.04	0.06	0.12	0.22	0.12	0.50	0.98	1.00
426.21	1220	202269	0.04	0.09	0.23	0.45	0.05	0.10	0.27	0.52	0.03	0.05	0.08	0.13	0.08	0.28	0.79	0.99
426.23	508	202269	0.04	0.06	0.12	0.22	0.04	0.06	0.14	0.25	0.03	0.04	0.06	0.08	0.05	0.14	0.44	0.77
426.24	659	202269	0.04	0.07	0.14	0.27	0.04	0.07	0.16	0.31	0.03	0.04	0.06	0.09	0.06	0.17	0.53	0.87
426.3	3818	202269	0.06	0.19	0.57	0.90	0.07	0.22	0.65	0.94	0.04	0.07	0.17	0.32	0.16	0.69	1.00	1.00
426.31	1758	202269	0.05	0.11	0.31	0.59	0.05	0.12	0.36	0.67	0.03	0.05	0.10	0.17	0.10	0.38	0.92	1.00
426.32	2030	202269	0.05	0.12	0.35	0.65	0.05	0.14	0.40	0.73	0.03	0.06	0.11	0.19	0.11	0.43	0.95	1.00
426.4	210	202269	0.03	0.04	0.07	0.12	0.03	0.05	0.08	0.13	0.03	0.03	0.04	0.05	0.04	0.08	0.21	0.41
426.9	2715	202269	0.06	0.15	0.44	0.78	0.06	0.17	0.51	0.84	0.04	0.06	0.13	0.24	0.13	0.54	0.99	1.00
426.91	2511	202269	0.05	0.14	0.42	0.74	0.06	0.16	0.48	0.82	0.04	0.06	0.13	0.23	0.12	0.51	0.98	1.00
427	25228	202269	0.18	0.75	1.00	1.00	0.21	0.82	1.00	1.00	0.07	0.23	0.68	0.95	0.67	1.00	1.00	1.00
427.1	3202	202269	0.06	0.16	0.50	0.84	0.06	0.19	0.58	0.90	0.04	0.07	0.15	0.28	0.14	0.61	0.99	1.00
427.11	2326	202269	0.05	0.13	0.39	0.71	0.06	0.15	0.45	0.79	0.04	0.06	0.12	0.21	0.12	0.48	0.97	1.00
427.12	956	202269	0.04	0.08	0.19	0.37	0.04	0.09	0.22	0.43	0.03	0.04	0.07	0.11	0.07	0.23	0.69	0.96
427.2	15491	202269	0.13	0.56	0.99	1.00	0.15	0.63	1.00	1.00	0.06	0.16	0.50	0.83	0.48	1.00	1.00	1.00
427.21	983	202269	0.04	0.08	0.19	0.38	0.04	0.09	0.22	0.44	0.03	0.04	0.07	0.12	0.07	0.24	0.70	0.96
427.3	3400	202269	0.06	0.17	0.53	0.86	0.06	0.20	0.60	0.91	0.04	0.07	0.15	0.29	0.15	0.64	1.00	1.00
427.4	1324	202269	0.04	0.09	0.25	0.48	0.05	0.10	0.28	0.55	0.03	0.05	0.09	0.14	0.08	0.30	0.83	0.99
427.41	365	202269	0.03	0.05	0.10	0.17	0.03	0.06	0.11	0.19	0.03	0.04	0.05	0.07	0.05	0.12	0.33	0.63
427.42	1121	202269	0.04	0.08	0.21	0.42	0.04	0.09	0.25	0.48	0.03	0.05	0.08	0.13	0.08	0.27	0.76	0.98
427.5	998	202269	0.04	0.08	0.20	0.38	0.04	0.09	0.23	0.44	0.03	0.04	0.07	0.12	0.07	0.24	0.71	0.97
427.6	837	202269	0.04	0.07	0.17	0.33	0.04	0.08	0.20	0.38	0.03	0.04	0.07	0.10	0.07	0.21	0.63	0.93
427.7	2398	202269	0.05	0.13	0.40	0.73	0.06	0.15	0.46	0.80	0.04	0.06	0.12	0.22	0.12	0.49	0.97	1.00
427.8	484	202269	0.04	0.06	0.12	0.21	0.04	0.06	0.13	0.24	0.03	0.04	0.05	0.08	0.05	0.14	0.42	0.75
427.9	3694	202269	0.06	0.18	0.56	0.89	0.07	0.21	0.64	0.93	0.04	0.07	0.16	0.31	0.16	0.67	1.00	1.00
428	6185	202269	0.08	0.27	0.77	0.98	0.09	0.32	0.84	0.99	0.04	0.09	0.24	0.47	0.23	0.87	1.00	1.00
428.1	3650	202269	0.06	0.18	0.56	0.88	0.07	0.21	0.63	0.93	0.04	0.07	0.16	0.31	0.16	0.67	1.00	1.00
428.2	4914	202269	0.07	0.23	0.68	0.95	0.08	0.26	0.76	0.98	0.04	0.08	0.20	0.39	0.20	0.79	1.00	1.00
429	756	202269	0.04	0.07	0.16	0.30	0.04	0.08	0.18	0.35	0.03	0.04	0.07	0.10	0.06	0.19	0.59	0.91
429.2	553	202269	0.04	0.06	0.13	0.23	0.04	0.07	0.14	0.27	0.03	0.04	0.06	0.08	0.06	0.15	0.47	0.80
430	1984	202269	0.05	0.12	0.34	0.64	0.05	0.13	0.40	0.72	0.03	0.05	0.11	0.19	0.11	0.42	0.94	1.00
430.1	816	202269	0.04	0.07	0.17	0.32	0.04	0.08	0.19	0.37	0.03	0.04	0.07	0.10	0.07	0.21	0.62	0.93
430.2	855	202269	0.04	0.07	0.17	0.34	0.04	0.08	0.20	0.39	0.03	0.04	0.07	0.11	0.07	0.21	0.64	0.94
430.3	308	202269	0.03	0.05	0.09	0.15	0.03	0.05	0.10	0.17	0.03	0.03	0.05	0.06	0.05	0.10	0.29	0.56
433	9077	202269	0.10	0.37	0.90	1.00	0.11	0.43	0.95	1.00	0.05	0.11	0.33	0.62	0.32	0.96	1.00	1.00
433.1	1354	202269	0.04	0.09	0.25	0.49	0.05	0.10	0.29	0.56	0.03	0.05	0.09	0.14	0.08	0.31	0.83	0.99
433.12	270	202269	0.03	0.05	0.08	0.14	0.03	0.05	0.09	0.15	0.03	0.03	0.05	0.06	0.04	0.10	0.26	0.50
433.2	4355	202269	0.07	0.21	0.63	0.93	0.07	0.24	0.71	0.96	0.04	0.08	0.18	0.36	0.18	0.74	1.00	1.00
433.21	4193	202269	0.07	0.20	0.61	0.92	0.07	0.23	0.69	0.96	0.04	0.08	0.18	0.35	0.17	0.73	1.00	1.00
433.3	3089	202269	0.06	0.16	0.49	0.83	0.06	0.18	0.56	0.89	0.04	0.07	0.14	0.27	0.14	0.60	0.99	1.00
433.31	2115	202269	0.05	0.12	0.36	0.67	0.05	0.14	0.42	0.75	0.04	0.06	0.11	0.20	0.11	0.45	0.95	1.00
433.5	364	202269	0.03	0.05	0.10	0.17	0.03	0.06	0.11	0.19	0.03	0.04	0.05	0.07	0.05	0.12	0.33	0.63
433.8	1333	202269	0.04	0.09	0.25	0.48	0.05	0.10	0.29	0.55	0.03	0.05	0.09	0.14	0.08	0.31	0.83	0.99
440	1453	202269	0.05	0.10	0.26	0.51	0.05	0.11	0.31	0.59	0.03	0.05	0.09	0.15	0.09	0.33	0.86	1.00
440.2	848	202269	0.04	0.07	0.17	0.33	0.04	0.08	0.20	0.39	0.03	0.04	0.07	0.11	0.07	0.21	0.64	0.94
440.9	331	202269	0.03	0.05	0.09	0.16	0.03	0.05	0.10	0.18	0.03	0.03	0.05	0.06	0.05	0.11	0.31	0.59
441	701	202269	0.04	0.07	0.15	0.28	0.04	0.07	0.17	0.33	0.03	0.04	0.06	0.09	0.06	0.18	0.56	0.89
441.1	321	202269	0.03	0.05	0.09	0.15	0.03	0.05	0.10	0.18	0.03	0.03	0.05	0.06	0.05	0.11	0.30	0.57
442	2079	202269	0.05	0.12	0.36	0.66	0.05	0.14	0.41	0.74	0.03	0.06	0.11	0.20	0.11	0.44	0.95	1.00
442.1	1632	202269	0.05	0.10	0.29	0.56	0.05	0.12	0.34	0.64	0.03	0.05	0.10	0.16	0.09	0.36	0.89	1.00
442.11	1047	202269	0.04	0.08	0.20	0.40	0.04	0.09	0.23	0.46	0.03	0.04	0.08	0.12	0.07	0.25	0.73	0.97
442.8	218	202269	0.03	0.04	0.07	0.12	0.03	0.05	0.08	0.13	0.03	0.03	0.04	0.05	0.04	0.09	0.22	0.42
443	4031	202269	0.06	0.20	0.60	0.91	0.07	0.22	0.67	0.95	0.04	0.07	0.17	0.34	0.17	0.71	1.00	1.00
443.1	1162	202269	0.04	0.09	0.22	0.43	0.04	0.10	0.26	0.50	0.03	0.05	0.08	0.13	0.08	0.27	0.77	0.98
443.7	397	202269	0.03	0.05	0.10	0.18	0.04	0.06	0.12	0.21	0.03	0.04	0.05	0.07	0.05	0.12	0.36	0.66
443.9	2679	202269	0.05	0.14	0.44	0.77	0.06	0.16	0.50	0.84	0.04	0.06	0.13	0.24	0.13	0.54	0.98	1.00
444	903	202269	0.04	0.08	0.18	0.35	0.04	0.08	0.21	0.41	0.03	0.04	0.07	0.11	0.07	0.22	0.67	0.95
444.1	560	202269	0.04	0.06	0.13	0.24	0.04	0.07	0.15	0.27	0.03	0.04	0.06	0.08	0.06	0.15	0.47	0.81
446	857	202269	0.04	0.07	0.17	0.34	0.04	0.08	0.20	0.39	0.03	0.04	0.07	0.11	0.07	0.21	0.65	0.94

446.5	417	202269	0.03	0.05	0.11	0.19	0.04	0.06	0.12	0.22	0.03	0.04	0.05	0.07	0.05	0.13	0.37	0.69
446.9	258	202269	0.03	0.05	0.08	0.13	0.03	0.05	0.09	0.15	0.03	0.03	0.04	0.06	0.04	0.09	0.25	0.48
447	1320	202269	0.04	0.09	0.24	0.48	0.05	0.10	0.28	0.55	0.03	0.05	0.09	0.14	0.08	0.30	0.82	0.99
447.1	858	202269	0.04	0.07	0.17	0.34	0.04	0.08	0.20	0.39	0.03	0.04	0.07	0.11	0.07	0.21	0.65	0.94
450	805	202269	0.04	0.07	0.17	0.32	0.04	0.08	0.19	0.37	0.03	0.04	0.07	0.10	0.07	0.20	0.62	0.92
451	3916	202269	0.06	0.19	0.58	0.90	0.07	0.22	0.66	0.95	0.04	0.07	0.17	0.33	0.17	0.70	1.00	1.00
451.2	3612	202269	0.06	0.18	0.55	0.88	0.07	0.21	0.63	0.93	0.04	0.07	0.16	0.31	0.16	0.66	1.00	1.00
452	598	202269	0.04	0.06	0.13	0.25	0.04	0.07	0.15	0.29	0.03	0.04	0.06	0.09	0.06	0.16	0.50	0.83
454	10793	202269	0.11	0.43	0.94	1.00	0.12	0.49	0.97	1.00	0.05	0.13	0.38	0.70	0.37	0.98	1.00	1.00
454.1	10333	202269	0.10	0.41	0.94	1.00	0.11	0.48	0.97	1.00	0.05	0.12	0.36	0.68	0.35	0.98	1.00	1.00
454.11	716	202269	0.04	0.07	0.15	0.29	0.04	0.07	0.17	0.34	0.03	0.04	0.06	0.10	0.06	0.19	0.57	0.89
455	23176	202269	0.17	0.71	1.00	1.00	0.20	0.79	1.00	1.00	0.07	0.22	0.65	0.94	0.63	1.00	1.00	1.00
456	224	202269	0.03	0.04	0.08	0.12	0.03	0.05	0.08	0.14	0.03	0.03	0.04	0.06	0.04	0.09	0.22	0.43
458	6659	202269	0.08	0.29	0.80	0.99	0.09	0.34	0.87	1.00	0.04	0.10	0.26	0.50	0.25	0.89	1.00	1.00
458.1	1622	202269	0.05	0.10	0.29	0.56	0.05	0.12	0.34	0.63	0.03	0.05	0.10	0.16	0.09	0.36	0.89	1.00
458.2	250	202269	0.03	0.05	0.08	0.13	0.03	0.05	0.09	0.15	0.03	0.03	0.04	0.06	0.04	0.09	0.24	0.47
458.9	4073	202269	0.06	0.20	0.60	0.91	0.07	0.23	0.68	0.95	0.04	0.07	0.18	0.34	0.17	0.71	1.00	1.00
459	17142	202269	0.14	0.60	0.99	1.00	0.16	0.67	1.00	1.00	0.06	0.17	0.53	0.87	0.52	1.00	1.00	1.00
459.9	16931	202269	0.14	0.59	0.99	1.00	0.16	0.67	1.00	1.00	0.06	0.17	0.53	0.86	0.51	1.00	1.00	1.00
465	2223	269389	0.05	0.13	0.38	0.69	0.05	0.14	0.44	0.77	0.04	0.06	0.12	0.21	0.11	0.47	0.96	1.00
465.2	841	269389	0.04	0.07	0.17	0.33	0.04	0.08	0.20	0.38	0.03	0.04	0.07	0.11	0.07	0.21	0.64	0.93
470	4226	269389	0.07	0.20	0.62	0.92	0.07	0.23	0.69	0.96	0.04	0.08	0.18	0.35	0.18	0.73	1.00	1.00
471	2933	269389	0.06	0.15	0.47	0.81	0.06	0.18	0.54	0.87	0.04	0.06	0.14	0.26	0.14	0.58	0.99	1.00
472	906	269389	0.04	0.08	0.18	0.35	0.04	0.08	0.21	0.41	0.03	0.04	0.07	0.11	0.07	0.22	0.67	0.95
473	2414	269389	0.05	0.14	0.40	0.73	0.06	0.15	0.47	0.80	0.04	0.06	0.12	0.22	0.12	0.50	0.97	1.00
473.3	263	269389	0.03	0.05	0.08	0.13	0.03	0.05	0.09	0.15	0.03	0.03	0.04	0.06	0.04	0.09	0.25	0.49
473.4	972	269389	0.04	0.08	0.19	0.37	0.04	0.09	0.22	0.43	0.03	0.04	0.07	0.11	0.07	0.24	0.70	0.96
474	1762	269389	0.05	0.11	0.31	0.59	0.05	0.12	0.36	0.67	0.03	0.05	0.10	0.17	0.10	0.39	0.92	1.00
474.1	552	269389	0.04	0.06	0.13	0.23	0.04	0.07	0.14	0.27	0.03	0.04	0.06	0.08	0.06	0.15	0.47	0.80
474.2	971	269389	0.04	0.08	0.19	0.37	0.04	0.09	0.22	0.43	0.03	0.04	0.07	0.11	0.07	0.24	0.70	0.96
475	2395	269389	0.05	0.13	0.40	0.73	0.06	0.15	0.46	0.80	0.04	0.06	0.12	0.22	0.12	0.49	0.97	1.00
476	1225	269389	0.04	0.09	0.23	0.45	0.05	0.10	0.27	0.52	0.03	0.05	0.08	0.13	0.08	0.29	0.80	0.99
477	2412	269389	0.05	0.13	0.40	0.73	0.06	0.15	0.46	0.80	0.04	0.06	0.12	0.22	0.12	0.50	0.97	1.00
478	338	269389	0.03	0.05	0.09	0.16	0.03	0.05	0.10	0.18	0.03	0.04	0.05	0.07	0.05	0.11	0.31	0.59
479	3786	269389	0.06	0.19	0.57	0.89	0.07	0.21	0.65	0.94	0.04	0.07	0.17	0.32	0.16	0.68	1.00	1.00
480	11693	269389	0.11	0.46	0.96	1.00	0.13	0.53	0.98	1.00	0.05	0.14	0.41	0.73	0.39	0.99	1.00	1.00
480.1	7555	269389	0.09	0.32	0.85	0.99	0.10	0.37	0.91	1.00	0.05	0.10	0.29	0.55	0.28	0.93	1.00	1.00
480.11	6741	269389	0.08	0.29	0.81	0.99	0.09	0.34	0.87	1.00	0.04	0.10	0.26	0.51	0.25	0.90	1.00	1.00
480.5	894	269389	0.04	0.08	0.18	0.35	0.04	0.08	0.21	0.40	0.03	0.04	0.07	0.11	0.07	0.22	0.66	0.95
481	418	269389	0.03	0.05	0.11	0.19	0.04	0.06	0.12	0.22	0.03	0.04	0.05	0.07	0.05	0.13	0.37	0.69
483	227	269389	0.03	0.04	0.08	0.12	0.03	0.05	0.08	0.14	0.03	0.03	0.04	0.06	0.04	0.09	0.22	0.44
495	24896	269389	0.18	0.75	1.00	1.00	0.21	0.82	1.00	1.00	0.07	0.23	0.69	0.96	0.67	1.00	1.00	1.00
495.2	229	269389	0.03	0.05	0.08	0.12	0.03	0.05	0.08	0.14	0.03	0.03	0.04	0.06	0.04	0.09	0.23	0.44
496	11361	269389	0.11	0.45	0.96	1.00	0.12	0.52	0.98	1.00	0.05	0.13	0.40	0.72	0.38	0.99	1.00	1.00
496.1	1937	269389	0.05	0.12	0.34	0.64	0.05	0.13	0.39	0.71	0.03	0.05	0.11	0.19	0.10	0.42	0.94	1.00
496.2	3258	269389	0.06	0.17	0.51	0.85	0.06	0.19	0.58	0.90	0.04	0.07	0.15	0.28	0.15	0.62	0.99	1.00
496.21	2997	269389	0.06	0.16	0.48	0.82	0.06	0.18	0.55	0.88	0.04	0.06	0.14	0.26	0.14	0.58	0.99	1.00
496.3	2143	269389	0.05	0.12	0.37	0.68	0.05	0.14	0.42	0.75	0.04	0.06	0.11	0.20	0.11	0.45	0.96	1.00
497	647	269389	0.04	0.06	0.14	0.27	0.04	0.07	0.16	0.31	0.03	0.04	0.06	0.09	0.06	0.17	0.53	0.86
500	459	269389	0.04	0.06	0.11	0.20	0.04	0.06	0.13	0.23	0.03	0.04	0.05	0.08	0.05	0.13	0.40	0.73
500.2	273	269389	0.03	0.05	0.08	0.14	0.03	0.05	0.09	0.16	0.03	0.03	0.05	0.06	0.04	0.10	0.26	0.51
501	852	269389	0.04	0.07	0.17	0.33	0.04	0.08	0.20	0.39	0.03	0.04	0.07	0.11	0.07	0.21	0.64	0.94
502	1036	269389	0.04	0.08	0.20	0.39	0.04	0.09	0.23	0.45	0.03	0.04	0.08	0.12	0.07	0.25	0.73	0.97
503	534	269389	0.04	0.06	0.12	0.23	0.04	0.06	0.14	0.26	0.03	0.04	0.06	0.08	0.06	0.15	0.45	0.79
504	389	269389	0.03	0.05	0.10	0.18	0.04	0.06	0.11	0.20	0.03	0.04	0.05	0.07	0.05	0.12	0.35	0.66
506	1207	269389	0.04	0.09	0.23	0.45	0.05	0.10	0.26	0.51	0.03	0.05	0.08	0.13	0.08	0.28	0.79	0.99
507	6979	269389	0.08	0.30	0.82	0.99	0.09	0.35	0.88	1.00	0.05	0.10	0.27	0.52	0.26	0.91	1.00	1.00
508	2447	269389	0.05	0.14	0.41	0.74	0.06	0.15	0.47	0.81	0.04	0.06	0.12	0.22	0.12	0.50	0.98	1.00
509	3054	269389	0.06	0.16	0.49	0.82	0.06	0.18	0.56	0.88	0.04	0.07	0.14	0.27	0.14	0.59	0.99	1.00
509.1	2479	269389	0.05	0.14	0.41	0.74	0.06	0.16	0.48	0.81	0.04	0.06	0.12	0.23	0.12	0.51	0.98	1.00
509.2	2205	269389	0.05	0.13	0.37	0.69	0.05	0.14	0.43	0.77	0.04	0.06	0.12	0.21	0.11	0.46	0.96	1.00
509.8	444	269389	0.03	0.06	0.11	0.20	0.04	0.06	0.12	0.23	0.03	0.04	0.05	0.07	0.05	0.13	0.39	0.71
510	1171	269389	0.04	0.09	0.22	0.43	0.04	0.10	0.26	0.50	0.03	0.05	0.08	0.13	0.08	0.28	0.78	0.98
512	9349	269389	0.10	0.38	0.91	1.00	0.11	0.44	0.95	1.00	0.05	0.12	0.34	0.64	0.33	0.97	1.00	1.00
512.1	268	269389	0.03	0.05	0.08	0.14	0.03	0.05	0.09	0.15	0.03	0.03	0.05	0.06	0.04	0.10	0.26	0.50
512.2	203	269389	0.03	0.04	0.07	0.11	0.03	0.05	0.08	0.13	0.03	0.03	0.04	0.05	0.04	0.08	0.21	0.40
512.7	6109	269389	0.08	0.27	0.77	0.98	0.09	0.32	0.84	0.99	0.04	0.09	0.24	0.47	0.23	0.87	1.00	1.00
512.8	2986	269389	0.06	0.16	0.48	0.82	0.06	0.18	0.55	0.88	0.04	0.06	0.14	0.26	0.14	0.58	0.99	1.00
512.9	884	269389	0.04	0.07	0.18	0.34	0.04	0.08	0.21	0.40	0.03	0.04	0.07	0.11	0.07	0.22	0.66	0.94
513	603	269389	0.04	0.06	0.14	0.25	0.04	0.07	0.15	0.29	0.03	0.04	0.06	0.09	0.06	0.16	0.50	0.84
513.4	251	269389	0.03	0.05	0.08	0.13	0.03	0.05	0.09	0.15	0.03	0.03	0.04	0.06	0.04	0.09	0.24	0.47
514	2607	269389	0.05	0.14	0.43	0.76	0.06	0.16	0.49	0.83	0.04	0.06	0.13	0.24	0.13	0.53	0.98	1.00
516	2036	269389	0.05	0.12	0.35	0.66	0.05	0.14	0.41	0.73	0.03	0.06	0.11	0.19	0.11	0.43	0.95	1.00
516.1	1948	269389	0.05	0.12	0.34	0.64	0.05	0.13	0.39	0.71	0.03	0.05	0.11	0.19	0.10	0.42	0.94	1.00

519	9890	269389	0.10	0.40	0.93	1.00	0.11	0.46	0.96	1.00	0.05	0.12	0.36	0.66	0.34	0.97	1.00	1.00
519.2	323	269389	0.03	0.05	0.09	0.15	0.03	0.05	0.10	0.18	0.03	0.03	0.05	0.06	0.05	0.11	0.30	0.58
519.8	9307	269389	0.10	0.38	0.91	1.00	0.11	0.44	0.95	1.00	0.05	0.12	0.34	0.64	0.33	0.97	1.00	1.00
519.9	272	269389	0.03	0.05	0.08	0.14	0.03	0.05	0.09	0.16	0.03	0.03	0.05	0.06	0.04	0.10	0.26	0.51
520	2122	191014	0.05	0.12	0.36	0.67	0.05	0.14	0.42	0.75	0.04	0.06	0.11	0.20	0.11	0.45	0.96	1.00
520.2	2041	191014	0.05	0.12	0.35	0.66	0.05	0.14	0.41	0.73	0.03	0.06	0.11	0.19	0.11	0.43	0.95	1.00
521	3012	191014	0.06	0.16	0.48	0.82	0.06	0.18	0.55	0.88	0.04	0.06	0.14	0.26	0.14	0.58	0.99	1.00
521.1	2952	191014	0.06	0.16	0.47	0.81	0.06	0.18	0.54	0.87	0.04	0.06	0.14	0.26	0.14	0.58	0.99	1.00
522	1661	191014	0.05	0.11	0.30	0.57	0.05	0.12	0.34	0.64	0.03	0.05	0.10	0.17	0.09	0.37	0.90	1.00
522.5	1117	191014	0.04	0.08	0.21	0.42	0.04	0.09	0.25	0.48	0.03	0.05	0.08	0.13	0.08	0.26	0.76	0.98
523	1638	191014	0.05	0.10	0.29	0.56	0.05	0.12	0.34	0.64	0.03	0.05	0.10	0.16	0.09	0.36	0.90	1.00
523.3	1133	191014	0.04	0.09	0.22	0.42	0.04	0.09	0.25	0.49	0.03	0.05	0.08	0.13	0.08	0.27	0.76	0.98
523.32	1000	191014	0.04	0.08	0.20	0.38	0.04	0.09	0.23	0.44	0.03	0.04	0.07	0.12	0.07	0.24	0.71	0.97
524	309	191014	0.03	0.05	0.09	0.15	0.03	0.05	0.10	0.17	0.03	0.03	0.05	0.06	0.05	0.10	0.29	0.56
524.3	276	191014	0.03	0.05	0.08	0.14	0.03	0.05	0.09	0.16	0.03	0.03	0.05	0.06	0.05	0.10	0.26	0.51
525	2546	191014	0.05	0.14	0.42	0.75	0.06	0.16	0.48	0.82	0.04	0.06	0.13	0.23	0.12	0.52	0.98	1.00
526	909	191014	0.04	0.08	0.18	0.35	0.04	0.08	0.21	0.41	0.03	0.04	0.07	0.11	0.07	0.22	0.67	0.95
526.1	312	191014	0.03	0.05	0.09	0.15	0.03	0.05	0.10	0.17	0.03	0.03	0.05	0.06	0.05	0.10	0.29	0.56
526.4	229	191014	0.03	0.05	0.08	0.12	0.03	0.05	0.08	0.14	0.03	0.03	0.04	0.06	0.04	0.09	0.23	0.44
526.41	222	191014	0.03	0.04	0.08	0.12	0.03	0.05	0.08	0.13	0.03	0.03	0.04	0.06	0.04	0.09	0.22	0.43
526.9	220	191014	0.03	0.04	0.07	0.12	0.03	0.05	0.08	0.13	0.03	0.03	0.04	0.06	0.04	0.09	0.22	0.43
527	663	191014	0.04	0.07	0.14	0.27	0.04	0.07	0.16	0.31	0.03	0.04	0.06	0.09	0.06	0.18	0.54	0.87
527.2	340	191014	0.03	0.05	0.09	0.16	0.03	0.05	0.10	0.18	0.03	0.04	0.05	0.07	0.05	0.11	0.31	0.60
528	3788	191014	0.06	0.19	0.57	0.89	0.07	0.21	0.65	0.94	0.04	0.07	0.17	0.32	0.16	0.68	1.00	1.00
528.1	475	191014	0.04	0.06	0.12	0.21	0.04	0.06	0.13	0.24	0.03	0.04	0.05	0.08	0.05	0.14	0.41	0.74
528.11	406	191014	0.03	0.05	0.10	0.18	0.04	0.06	0.12	0.21	0.03	0.04	0.05	0.07	0.05	0.12	0.36	0.67
528.5	644	191014	0.04	0.06	0.14	0.26	0.04	0.07	0.16	0.31	0.03	0.04	0.06	0.09	0.06	0.17	0.52	0.86
528.6	298	191014	0.03	0.05	0.09	0.15	0.03	0.05	0.10	0.17	0.03	0.03	0.05	0.06	0.05	0.10	0.28	0.54
528.7	289	191014	0.03	0.05	0.09	0.14	0.03	0.05	0.10	0.16	0.03	0.03	0.05	0.06	0.05	0.10	0.27	0.53
529	1210	191014	0.04	0.09	0.23	0.45	0.05	0.10	0.26	0.51	0.03	0.05	0.08	0.13	0.08	0.28	0.79	0.99
529.1	290	191014	0.03	0.05	0.09	0.14	0.03	0.05	0.10	0.16	0.03	0.03	0.05	0.06	0.05	0.10	0.27	0.53
530	34889	191014	0.22	0.85	1.00	1.00	0.26	0.91	1.00	1.00	0.08	0.29	0.80	0.99	0.78	1.00	1.00	1.00
530.1	32569	191014	0.21	0.83	1.00	1.00	0.25	0.89	1.00	1.00	0.08	0.27	0.77	0.98	0.76	1.00	1.00	1.00
530.11	15750	191014	0.13	0.56	0.99	1.00	0.15	0.64	1.00	1.00	0.06	0.16	0.50	0.84	0.49	1.00	1.00	1.00
530.12	4731	191014	0.07	0.22	0.66	0.95	0.07	0.25	0.74	0.97	0.04	0.08	0.20	0.38	0.19	0.77	1.00	1.00
530.13	3105	191014	0.06	0.16	0.49	0.83	0.06	0.18	0.56	0.89	0.04	0.07	0.14	0.27	0.14	0.60	0.99	1.00
530.14	10069	191014	0.10	0.40	0.93	1.00	0.11	0.47	0.96	1.00	0.05	0.12	0.36	0.67	0.35	0.97	1.00	1.00
530.2	1770	191014	0.05	0.11	0.31	0.60	0.05	0.12	0.36	0.67	0.03	0.05	0.10	0.17	0.10	0.39	0.92	1.00
530.3	1940	191014	0.05	0.12	0.34	0.63	0.05	0.13	0.39	0.71	0.03	0.05	0.11	0.19	0.10	0.42	0.94	1.00
530.5	637	191014	0.04	0.06	0.14	0.26	0.04	0.07	0.16	0.30	0.03	0.04	0.06	0.09	0.06	0.17	0.52	0.85
530.7	303	191014	0.03	0.05	0.09	0.15	0.03	0.05	0.10	0.17	0.03	0.03	0.05	0.06	0.05	0.10	0.28	0.55
530.9	2010	191014	0.05	0.12	0.35	0.65	0.05	0.13	0.40	0.73	0.03	0.06	0.11	0.19	0.11	0.43	0.95	1.00
531	7143	191014	0.08	0.31	0.83	0.99	0.09	0.36	0.89	1.00	0.05	0.10	0.27	0.53	0.26	0.91	1.00	1.00
531.1	638	191014	0.04	0.06	0.14	0.26	0.04	0.07	0.16	0.30	0.03	0.04	0.06	0.09	0.06	0.17	0.52	0.86
531.2	4026	191014	0.06	0.19	0.60	0.91	0.07	0.22	0.67	0.95	0.04	0.07	0.17	0.33	0.17	0.71	1.00	1.00
531.3	2794	191014	0.06	0.15	0.45	0.79	0.06	0.17	0.52	0.85	0.04	0.06	0.13	0.25	0.13	0.55	0.99	1.00
531.4	404	191014	0.03	0.05	0.10	0.18	0.04	0.06	0.12	0.21	0.03	0.04	0.05	0.07	0.05	0.12	0.36	0.67
532	6669	191014	0.08	0.29	0.80	0.99	0.09	0.34	0.87	1.00	0.04	0.10	0.26	0.50	0.25	0.89	1.00	1.00
535	28094	191014	0.19	0.78	1.00	1.00	0.22	0.85	1.00	1.00	0.07	0.25	0.72	0.97	0.70	1.00	1.00	1.00
535.1	1145	191014	0.04	0.09	0.22	0.43	0.04	0.09	0.25	0.49	0.03	0.05	0.08	0.13	0.08	0.27	0.77	0.98
535.2	372	191014	0.03	0.05	0.10	0.17	0.03	0.06	0.11	0.20	0.03	0.04	0.05	0.07	0.05	0.12	0.34	0.64
535.6	7254	191014	0.08	0.31	0.83	0.99	0.09	0.36	0.89	1.00	0.05	0.10	0.27	0.53	0.27	0.92	1.00	1.00
535.8	8264	191014	0.09	0.34	0.88	1.00	0.10	0.40	0.93	1.00	0.05	0.11	0.30	0.58	0.29	0.94	1.00	1.00
536	11052	191014	0.11	0.43	0.95	1.00	0.12	0.50	0.97	1.00	0.05	0.13	0.38	0.70	0.37	0.98	1.00	1.00
536.8	11046	191014	0.11	0.43	0.95	1.00	0.12	0.50	0.97	1.00	0.05	0.13	0.38	0.70	0.37	0.98	1.00	1.00
537	3459	191014	0.06	0.17	0.53	0.87	0.07	0.20	0.61	0.92	0.04	0.07	0.16	0.30	0.15	0.64	1.00	1.00
540	3462	191014	0.06	0.17	0.53	0.87	0.07	0.20	0.61	0.92	0.04	0.07	0.16	0.30	0.15	0.64	1.00	1.00
540.1	3299	191014	0.06	0.17	0.51	0.85	0.06	0.19	0.59	0.91	0.04	0.07	0.15	0.28	0.15	0.62	1.00	1.00
540.11	2801	191014	0.06	0.15	0.45	0.79	0.06	0.17	0.52	0.85	0.04	0.06	0.13	0.25	0.13	0.55	0.99	1.00
550	45454	191014	0.27	0.92	1.00	1.00	0.31	0.96	1.00	1.00	0.09	0.34	0.88	1.00	0.86	1.00	1.00	1.00
550.1	15079	191014	0.13	0.54	0.99	1.00	0.15	0.62	0.99	1.00	0.06	0.16	0.49	0.82	0.47	1.00	1.00	1.00
550.2	26753	191014	0.19	0.77	1.00	1.00	0.22	0.83	1.00	1.00	0.07	0.24	0.70	0.96	0.69	1.00	1.00	1.00
550.3	634	191014	0.04	0.06	0.14	0.26	0.04	0.07	0.16	0.30	0.03	0.04	0.06	0.09	0.06	0.17	0.52	0.85
550.4	3634	191014	0.06	0.18	0.55	0.88	0.07	0.21	0.63	0.93	0.04	0.07	0.16	0.31	0.16	0.66	1.00	1.00
550.5	3384	191014	0.06	0.17	0.52	0.86	0.06	0.20	0.60	0.91	0.04	0.07	0.15	0.29	0.15	0.63	1.00	1.00
550.6	246	191014	0.03	0.05	0.08	0.13	0.03	0.05	0.09	0.14	0.03	0.03	0.04	0.06	0.04	0.09	0.24	0.47
555	4149	191014	0.07	0.20	0.61	0.92	0.07	0.23	0.68	0.96	0.04	0.07	0.18	0.34	0.17	0.72	1.00	1.00
555.1	1621	191014	0.05	0.10	0.29	0.56	0.05	0.12	0.34	0.63	0.03	0.05	0.10	0.16	0.09	0.36	0.89	1.00
555.2	2920	191014	0.06	0.15	0.47	0.80	0.06	0.18	0.54	0.87	0.04	0.06	0.14	0.26	0.13	0.57	0.99	1.00
555.21	541	191014	0.04	0.06	0.13	0.23	0.04	0.06	0.14	0.27	0.03	0.04	0.06	0.08	0.06	0.15	0.46	0.79
556	1084	191014	0.04	0.08	0.21	0.41	0.04	0.09	0.24	0.47	0.03	0.05	0.08	0.12	0.08	0.26	0.75	0.98
556.1	711	191014	0.04	0.07	0.15	0.29	0.04	0.07	0.17	0.33	0.03	0.04	0.06	0.10	0.06	0.18	0.57	0.89
557	287	191014	0.03	0.05	0.09	0.14	0.03	0.05	0.09	0.16	0.03	0.03	0.05	0.06	0.05	0.10	0.27	0.53
557.1	1764	191014	0.05	0.11	0.31	0.59	0.05	0.12	0.36	0.67	0.03	0.05	0.10	0.17	0.10	0.39	0.92	1.00

558	13696	191014	0.12	0.51	0.98	1.00	0.14	0.58	0.99	1.00	0.06	0.15	0.45	0.79	0.44	0.99	1.00	1.00
559	1579	191014	0.05	0.10	0.28	0.55	0.05	0.11	0.33	0.62	0.03	0.05	0.09	0.16	0.09	0.35	0.88	1.00
560	4175	191014	0.07	0.20	0.61	0.92	0.07	0.23	0.69	0.96	0.04	0.07	0.18	0.34	0.17	0.72	1.00	1.00
560.1	602	191014	0.04	0.06	0.14	0.25	0.04	0.07	0.15	0.29	0.03	0.04	0.06	0.09	0.06	0.16	0.50	0.83
560.2	282	191014	0.03	0.05	0.09	0.14	0.03	0.05	0.09	0.16	0.03	0.03	0.05	0.06	0.05	0.10	0.27	0.52
560.3	874	191014	0.04	0.07	0.18	0.34	0.04	0.08	0.20	0.40	0.03	0.04	0.07	0.11	0.07	0.22	0.65	0.94
560.4	3467	191014	0.06	0.17	0.53	0.87	0.07	0.20	0.61	0.92	0.04	0.07	0.16	0.30	0.15	0.64	1.00	1.00
561	16127	191014	0.13	0.57	0.99	1.00	0.15	0.65	1.00	1.00	0.06	0.17	0.51	0.85	0.49	1.00	1.00	1.00
561.1	536	191014	0.04	0.06	0.13	0.23	0.04	0.06	0.14	0.26	0.03	0.04	0.06	0.08	0.06	0.15	0.45	0.79
561.2	1767	191014	0.05	0.11	0.31	0.59	0.05	0.12	0.36	0.67	0.03	0.05	0.10	0.17	0.10	0.39	0.92	1.00
562	28363	191014	0.20	0.79	1.00	1.00	0.22	0.85	1.00	1.00	0.07	0.25	0.72	0.97	0.71	1.00	1.00	1.00
562.1	28326	191014	0.20	0.79	1.00	1.00	0.22	0.85	1.00	1.00	0.07	0.25	0.72	0.97	0.71	1.00	1.00	1.00
563	10878	191014	0.11	0.43	0.94	1.00	0.12	0.49	0.97	1.00	0.05	0.13	0.38	0.70	0.37	0.98	1.00	1.00
564	22341	191014	0.17	0.70	1.00	1.00	0.19	0.77	1.00	1.00	0.07	0.21	0.63	0.93	0.62	1.00	1.00	1.00
564.1	5580	191014	0.07	0.25	0.73	0.97	0.08	0.29	0.80	0.99	0.04	0.09	0.22	0.44	0.22	0.84	1.00	1.00
564.8	1743	191014	0.05	0.11	0.31	0.59	0.05	0.12	0.36	0.67	0.03	0.05	0.10	0.17	0.10	0.38	0.91	1.00
564.9	15641	191014	0.13	0.56	0.99	1.00	0.15	0.63	1.00	1.00	0.06	0.16	0.50	0.84	0.48	1.00	1.00	1.00
565	14352	191014	0.13	0.53	0.98	1.00	0.14	0.60	0.99	1.00	0.06	0.15	0.47	0.81	0.45	1.00	1.00	1.00
565.1	7409	191014	0.09	0.32	0.84	0.99	0.09	0.37	0.90	1.00	0.05	0.10	0.28	0.54	0.27	0.92	1.00	1.00
567	949	191014	0.04	0.08	0.19	0.37	0.04	0.09	0.22	0.42	0.03	0.04	0.07	0.11	0.07	0.23	0.69	0.96
568	3567	191014	0.06	0.18	0.55	0.88	0.07	0.20	0.62	0.92	0.04	0.07	0.16	0.30	0.16	0.66	1.00	1.00
568.1	3346	191014	0.06	0.17	0.52	0.85	0.06	0.19	0.59	0.91	0.04	0.07	0.15	0.29	0.15	0.63	1.00	1.00
569	4313	191014	0.07	0.21	0.62	0.93	0.07	0.24	0.70	0.96	0.04	0.08	0.18	0.35	0.18	0.74	1.00	1.00
569.1	208	191014	0.03	0.04	0.07	0.11	0.03	0.05	0.08	0.13	0.03	0.03	0.04	0.05	0.04	0.08	0.21	0.41
569.2	515	191014	0.04	0.06	0.12	0.22	0.04	0.06	0.14	0.26	0.03	0.04	0.06	0.08	0.06	0.15	0.44	0.77
571	4206	191014	0.07	0.20	0.61	0.92	0.07	0.23	0.69	0.96	0.04	0.08	0.18	0.35	0.17	0.73	1.00	1.00
571.5	3329	191014	0.06	0.17	0.52	0.85	0.06	0.19	0.59	0.91	0.04	0.07	0.15	0.29	0.15	0.63	1.00	1.00
571.51	749	191014	0.04	0.07	0.16	0.30	0.04	0.08	0.18	0.35	0.03	0.04	0.06	0.10	0.06	0.19	0.59	0.91
571.6	258	191014	0.03	0.05	0.08	0.13	0.03	0.05	0.09	0.15	0.03	0.03	0.04	0.06	0.04	0.09	0.25	0.48
571.8	1140	191014	0.04	0.09	0.22	0.42	0.04	0.09	0.25	0.49	0.03	0.05	0.08	0.13	0.08	0.27	0.77	0.98
571.81	589	191014	0.04	0.06	0.13	0.25	0.04	0.07	0.15	0.28	0.03	0.04	0.06	0.09	0.06	0.16	0.49	0.83
572	1656	191014	0.05	0.11	0.29	0.57	0.05	0.12	0.34	0.64	0.03	0.05	0.10	0.17	0.09	0.37	0.90	1.00
573	1712	191014	0.05	0.11	0.30	0.58	0.05	0.12	0.35	0.66	0.03	0.05	0.10	0.17	0.10	0.38	0.91	1.00
573.3	295	191014	0.03	0.05	0.09	0.15	0.03	0.05	0.10	0.16	0.03	0.03	0.05	0.06	0.05	0.10	0.28	0.54
573.5	914	191014	0.04	0.08	0.18	0.35	0.04	0.08	0.21	0.41	0.03	0.04	0.07	0.11	0.07	0.22	0.67	0.95
573.7	3714	191014	0.06	0.18	0.56	0.89	0.07	0.21	0.64	0.93	0.04	0.07	0.16	0.31	0.16	0.67	1.00	1.00
573.9	214	191014	0.03	0.04	0.07	0.12	0.03	0.05	0.08	0.13	0.03	0.03	0.04	0.05	0.04	0.08	0.21	0.42
574	15179	191014	0.13	0.55	0.99	1.00	0.15	0.62	1.00	1.00	0.06	0.16	0.49	0.82	0.47	1.00	1.00	1.00
574.1	12924	191014	0.12	0.49	0.97	1.00	0.13	0.56	0.99	1.00	0.05	0.14	0.43	0.77	0.42	0.99	1.00	1.00
574.11	1537	191014	0.05	0.10	0.28	0.54	0.05	0.11	0.32	0.61	0.03	0.05	0.09	0.16	0.09	0.34	0.88	1.00
574.12	5200	191014	0.07	0.24	0.70	0.96	0.08	0.27	0.78	0.98	0.04	0.08	0.21	0.41	0.20	0.81	1.00	1.00
574.2	2550	191014	0.05	0.14	0.42	0.75	0.06	0.16	0.48	0.82	0.04	0.06	0.13	0.23	0.12	0.52	0.98	1.00
574.3	2574	191014	0.05	0.14	0.42	0.75	0.06	0.16	0.49	0.82	0.04	0.06	0.13	0.23	0.12	0.52	0.98	1.00
575	3989	191014	0.06	0.19	0.59	0.91	0.07	0.22	0.67	0.95	0.04	0.07	0.17	0.33	0.17	0.70	1.00	1.00
575.1	599	191014	0.04	0.06	0.13	0.25	0.04	0.07	0.15	0.29	0.03	0.04	0.06	0.09	0.06	0.16	0.50	0.83
575.2	850	191014	0.04	0.07	0.17	0.33	0.04	0.08	0.20	0.39	0.03	0.04	0.07	0.11	0.07	0.21	0.64	0.94
575.6	437	191014	0.03	0.06	0.11	0.19	0.04	0.06	0.12	0.22	0.03	0.04	0.05	0.07	0.05	0.13	0.38	0.71
575.7	1372	191014	0.04	0.09	0.25	0.49	0.05	0.11	0.29	0.56	0.03	0.05	0.09	0.14	0.09	0.31	0.84	0.99
575.8	1096	191014	0.04	0.08	0.21	0.41	0.04	0.09	0.24	0.47	0.03	0.05	0.08	0.12	0.08	0.26	0.75	0.98
575.9	360	191014	0.03	0.05	0.10	0.17	0.03	0.06	0.11	0.19	0.03	0.04	0.05	0.07	0.05	0.11	0.33	0.62
577	2747	191014	0.06	0.15	0.45	0.78	0.06	0.17	0.51	0.85	0.04	0.06	0.13	0.25	0.13	0.55	0.99	1.00
577.1	1925	191014	0.05	0.12	0.33	0.63	0.05	0.13	0.39	0.71	0.03	0.05	0.11	0.19	0.10	0.41	0.94	1.00
577.2	473	191014	0.04	0.06	0.12	0.21	0.04	0.06	0.13	0.24	0.03	0.04	0.05	0.08	0.05	0.14	0.41	0.74
577.3	388	191014	0.03	0.05	0.10	0.18	0.04	0.06	0.11	0.20	0.03	0.04	0.05	0.07	0.05	0.12	0.35	0.65
578	19880	191014	0.15	0.65	1.00	1.00	0.18	0.73	1.00	1.00	0.06	0.19	0.59	0.91	0.57	1.00	1.00	1.00
578.1	1882	191014	0.05	0.11	0.33	0.62	0.05	0.13	0.38	0.70	0.03	0.05	0.10	0.18	0.10	0.41	0.93	1.00
578.2	2514	191014	0.05	0.14	0.42	0.74	0.06	0.16	0.48	0.82	0.04	0.06	0.13	0.23	0.12	0.51	0.98	1.00
578.8	11473	191014	0.11	0.45	0.95	1.00	0.12	0.51	0.98	1.00	0.05	0.13	0.39	0.72	0.38	0.99	1.00	1.00
578.9	6068	191014	0.08	0.27	0.77	0.98	0.08	0.31	0.84	0.99	0.04	0.09	0.24	0.46	0.23	0.86	1.00	1.00
579	1543	191014	0.05	0.10	0.28	0.54	0.05	0.11	0.32	0.61	0.03	0.05	0.09	0.16	0.09	0.35	0.88	1.00
579.2	447	191014	0.03	0.06	0.11	0.20	0.04	0.06	0.12	0.23	0.03	0.04	0.05	0.07	0.05	0.13	0.39	0.72
579.8	1887	191014	0.05	0.11	0.33	0.62	0.05	0.13	0.38	0.70	0.03	0.05	0.10	0.18	0.10	0.41	0.93	1.00
580	1984	234338	0.05	0.12	0.34	0.64	0.05	0.13	0.40	0.72	0.03	0.05	0.11	0.19	0.11	0.42	0.94	1.00
580.1	906	234338	0.04	0.08	0.18	0.35	0.04	0.08	0.21	0.41	0.03	0.04	0.07	0.11	0.07	0.22	0.67	0.95
580.14	723	234338	0.04	0.07	0.15	0.29	0.04	0.07	0.18	0.34	0.03	0.04	0.06	0.10	0.06	0.19	0.57	0.90
580.2	1042	234338	0.04	0.08	0.20	0.39	0.04	0.09	0.23	0.46	0.03	0.04	0.08	0.12	0.07	0.25	0.73	0.97
580.3	301	234338	0.03	0.05	0.09	0.15	0.03	0.05	0.10	0.17	0.03	0.03	0.05	0.06	0.05	0.10	0.28	0.55
580.32	286	234338	0.03	0.05	0.09	0.14	0.03	0.05	0.09	0.16	0.03	0.03	0.05	0.06	0.05	0.10	0.27	0.53
585	10995	234338	0.11	0.43	0.95	1.00	0.12	0.50	0.98	1.00	0.05	0.13	0.38	0.71	0.37	0.98	1.00	1.00
585.1	6203	234338	0.08	0.28	0.78	0.98	0.09	0.32	0.85	0.99	0.04	0.09	0.24	0.48	0.24	0.87	1.00	1.00
585.2	1430	234338	0.04	0.10	0.26	0.51	0.05	0.11	0.30	0.58	0.03	0.05	0.09	0.15	0.09	0.32	0.85	0.99
585.3	5710	234338	0.08	0.26	0.74	0.98	0.08	0.30	0.82	0.99	0.04	0.09	0.23	0.45	0.22	0.85	1.00	1.00
585.31	507	234338	0.04	0.06	0.12	0.22	0.04	0.06	0.14	0.25	0.03	0.04	0.06	0.08	0.05	0.14	0.43	0.77
585.32	426	234338	0.03	0.05	0.11	0.19	0.04	0.06	0.12	0.22	0.03	0.04	0.05	0.07	0.05	0.13	0.38	0.69

585.33	3054	234338	0.06	0.16	0.49	0.82	0.06	0.18	0.56	0.88	0.04	0.07	0.14	0.27	0.14	0.59	0.99	1.00
585.34	455	234338	0.04	0.06	0.11	0.20	0.04	0.06	0.13	0.23	0.03	0.04	0.05	0.07	0.05	0.13	0.40	0.72
585.4	343	234338	0.03	0.05	0.09	0.16	0.03	0.05	0.11	0.18	0.03	0.04	0.05	0.07	0.05	0.11	0.31	0.60
586	3536	234338	0.06	0.18	0.54	0.87	0.07	0.20	0.62	0.92	0.04	0.07	0.16	0.30	0.15	0.65	1.00	1.00
586.1	232	234338	0.03	0.05	0.08	0.12	0.03	0.05	0.08	0.14	0.03	0.03	0.04	0.06	0.04	0.09	0.23	0.45
586.11	200	234338	0.03	0.04	0.07	0.11	0.03	0.05	0.08	0.13	0.03	0.03	0.04	0.05	0.04	0.08	0.20	0.39
586.2	1393	234338	0.04	0.10	0.26	0.50	0.05	0.11	0.30	0.57	0.03	0.05	0.09	0.15	0.09	0.32	0.84	0.99
586.4	936	234338	0.04	0.08	0.19	0.36	0.04	0.08	0.21	0.42	0.03	0.04	0.07	0.11	0.07	0.23	0.68	0.96
587	334	234338	0.03	0.05	0.09	0.16	0.03	0.05	0.10	0.18	0.03	0.04	0.05	0.06	0.05	0.11	0.31	0.59
590	1361	234338	0.04	0.09	0.25	0.49	0.05	0.10	0.29	0.56	0.03	0.05	0.09	0.14	0.08	0.31	0.84	0.99
591	12543	234338	0.12	0.48	0.97	1.00	0.13	0.55	0.99	1.00	0.05	0.14	0.43	0.76	0.41	0.99	1.00	1.00
592	2885	234338	0.06	0.15	0.46	0.80	0.06	0.17	0.53	0.87	0.04	0.06	0.14	0.26	0.13	0.57	0.99	1.00
592.1	2766	234338	0.06	0.15	0.45	0.78	0.06	0.17	0.52	0.85	0.04	0.06	0.13	0.25	0.13	0.55	0.99	1.00
592.12	833	234338	0.04	0.07	0.17	0.33	0.04	0.08	0.20	0.38	0.03	0.04	0.07	0.10	0.07	0.21	0.63	0.93
592.13	233	234338	0.03	0.05	0.08	0.12	0.03	0.05	0.08	0.14	0.03	0.03	0.04	0.06	0.04	0.09	0.23	0.45
593	14542	234338	0.13	0.54	0.98	1.00	0.14	0.61	0.99	1.00	0.06	0.16	0.48	0.81	0.46	1.00	1.00	1.00
594	6165	234338	0.08	0.27	0.78	0.98	0.09	0.32	0.84	0.99	0.04	0.09	0.24	0.47	0.24	0.87	1.00	1.00
594.1	3198	234338	0.06	0.16	0.50	0.84	0.06	0.19	0.58	0.90	0.04	0.07	0.15	0.28	0.14	0.61	0.99	1.00
594.2	767	234338	0.04	0.07	0.16	0.31	0.04	0.08	0.18	0.35	0.03	0.04	0.07	0.10	0.06	0.20	0.60	0.91
594.3	2243	234338	0.05	0.13	0.38	0.70	0.05	0.15	0.44	0.77	0.04	0.06	0.12	0.21	0.11	0.47	0.96	1.00
594.8	1754	234338	0.05	0.11	0.31	0.59	0.05	0.12	0.36	0.67	0.03	0.05	0.10	0.17	0.10	0.38	0.91	1.00
595	2075	234338	0.05	0.12	0.36	0.66	0.05	0.14	0.41	0.74	0.03	0.06	0.11	0.20	0.11	0.44	0.95	1.00
596	9275	234338	0.10	0.38	0.91	1.00	0.11	0.44	0.95	1.00	0.05	0.12	0.34	0.64	0.33	0.96	1.00	1.00
596.1	1831	234338	0.05	0.11	0.32	0.61	0.05	0.13	0.37	0.69	0.03	0.05	0.10	0.18	0.10	0.40	0.93	1.00
596.5	1431	234338	0.04	0.10	0.26	0.51	0.05	0.11	0.30	0.58	0.03	0.05	0.09	0.15	0.09	0.32	0.85	0.99
597	3888	234338	0.06	0.19	0.58	0.90	0.07	0.22	0.66	0.94	0.04	0.07	0.17	0.33	0.17	0.69	1.00	1.00
597.1	3159	234338	0.06	0.16	0.50	0.84	0.06	0.19	0.57	0.89	0.04	0.07	0.15	0.28	0.14	0.61	0.99	1.00
598	3286	234338	0.06	0.17	0.51	0.85	0.06	0.19	0.59	0.90	0.04	0.07	0.15	0.28	0.15	0.62	1.00	1.00
598.9	299	234338	0.03	0.05	0.09	0.15	0.03	0.05	0.10	0.17	0.03	0.03	0.05	0.06	0.05	0.10	0.28	0.54
599	17536	234338	0.14	0.61	0.99	1.00	0.16	0.69	1.00	1.00	0.06	0.18	0.55	0.88	0.53	1.00	1.00	1.00
599.2	7478	234338	0.09	0.32	0.85	0.99	0.10	0.37	0.90	1.00	0.05	0.10	0.28	0.55	0.27	0.92	1.00	1.00
599.3	1104	234338	0.04	0.08	0.21	0.41	0.04	0.09	0.24	0.48	0.03	0.05	0.08	0.12	0.08	0.26	0.75	0.98
599.4	8031	234338	0.09	0.34	0.87	1.00	0.10	0.39	0.92	1.00	0.05	0.11	0.30	0.58	0.29	0.94	1.00	1.00
599.5	3626	234338	0.06	0.18	0.55	0.88	0.07	0.21	0.63	0.93	0.04	0.07	0.16	0.31	0.16	0.66	1.00	1.00
599.9	1683	234338	0.05	0.11	0.30	0.57	0.05	0.12	0.35	0.65	0.03	0.05	0.10	0.17	0.10	0.37	0.90	1.00
600	11447	114193	0.11	0.43	0.95	1.00	0.12	0.50	0.97	1.00	0.05	0.13	0.38	0.70	0.37	0.98	1.00	1.00
601	2119	114193	0.05	0.12	0.36	0.67	0.05	0.14	0.42	0.75	0.04	0.06	0.11	0.20	0.11	0.44	0.95	1.00
601.1	1527	114193	0.05	0.10	0.27	0.53	0.05	0.11	0.32	0.61	0.03	0.05	0.09	0.16	0.09	0.34	0.87	1.00
601.11	231	114193	0.03	0.05	0.08	0.12	0.03	0.05	0.08	0.14	0.03	0.03	0.04	0.06	0.04	0.09	0.23	0.44
601.12	908	114193	0.04	0.08	0.18	0.35	0.04	0.08	0.21	0.41	0.03	0.04	0.07	0.11	0.07	0.22	0.67	0.95
601.4	283	114193	0.03	0.05	0.09	0.14	0.03	0.05	0.09	0.16	0.03	0.03	0.05	0.06	0.05	0.10	0.27	0.52
601.8	291	114193	0.03	0.05	0.09	0.14	0.03	0.05	0.10	0.16	0.03	0.03	0.05	0.06	0.05	0.10	0.27	0.53
602	1292	114193	0.04	0.09	0.24	0.47	0.05	0.10	0.28	0.54	0.03	0.05	0.08	0.14	0.08	0.30	0.81	0.99
603	2075	117407	0.05	0.12	0.35	0.66	0.05	0.14	0.41	0.74	0.03	0.06	0.11	0.20	0.11	0.44	0.95	1.00
603.1	1274	117407	0.04	0.09	0.24	0.46	0.05	0.10	0.27	0.53	0.03	0.05	0.08	0.14	0.08	0.29	0.81	0.99
604	3185	117407	0.06	0.16	0.50	0.83	0.06	0.19	0.57	0.89	0.04	0.07	0.15	0.27	0.14	0.60	0.99	1.00
604.1	2635	117407	0.05	0.14	0.43	0.76	0.06	0.16	0.49	0.83	0.04	0.06	0.13	0.24	0.13	0.53	0.98	1.00
604.3	752	117407	0.04	0.07	0.16	0.30	0.04	0.08	0.18	0.35	0.03	0.04	0.06	0.10	0.06	0.19	0.59	0.91
605	482	117407	0.04	0.06	0.12	0.21	0.04	0.06	0.13	0.24	0.03	0.04	0.05	0.08	0.05	0.14	0.42	0.75
608	2620	117407	0.05	0.14	0.43	0.76	0.06	0.16	0.49	0.83	0.04	0.06	0.13	0.23	0.12	0.52	0.98	1.00
610	2479	116931	0.05	0.14	0.41	0.74	0.06	0.15	0.47	0.81	0.04	0.06	0.12	0.22	0.12	0.50	0.98	1.00
610.1	805	116931	0.04	0.07	0.17	0.32	0.04	0.08	0.19	0.37	0.03	0.04	0.07	0.10	0.07	0.20	0.62	0.92
610.2	275	116931	0.03	0.05	0.08	0.14	0.03	0.05	0.09	0.16	0.03	0.03	0.05	0.06	0.04	0.10	0.26	0.51
610.3	266	116931	0.03	0.05	0.08	0.13	0.03	0.05	0.09	0.15	0.03	0.03	0.05	0.06	0.04	0.10	0.25	0.50
610.4	1353	116931	0.04	0.09	0.25	0.49	0.05	0.10	0.29	0.56	0.03	0.05	0.09	0.14	0.08	0.31	0.83	0.99
610.8	644	116931	0.04	0.06	0.14	0.26	0.04	0.07	0.16	0.31	0.03	0.04	0.06	0.09	0.06	0.17	0.52	0.86
611	1363	234338	0.04	0.09	0.25	0.49	0.05	0.10	0.29	0.56	0.03	0.05	0.09	0.14	0.09	0.31	0.84	0.99
611.3	1305	234338	0.04	0.09	0.24	0.47	0.05	0.10	0.28	0.54	0.03	0.05	0.08	0.14	0.08	0.30	0.82	0.99
612	788	234338	0.04	0.07	0.16	0.31	0.04	0.08	0.19	0.36	0.03	0.04	0.07	0.10	0.07	0.20	0.61	0.92
612.2	715	234338	0.04	0.07	0.15	0.29	0.04	0.07	0.17	0.33	0.03	0.04	0.06	0.10	0.06	0.19	0.57	0.89
613	1712	234338	0.05	0.11	0.30	0.58	0.05	0.12	0.35	0.66	0.03	0.05	0.10	0.17	0.10	0.38	0.91	1.00
613.1	615	234338	0.04	0.06	0.14	0.26	0.04	0.07	0.16	0.30	0.03	0.04	0.06	0.09	0.06	0.17	0.51	0.84
613.7	614	234338	0.04	0.06	0.14	0.25	0.04	0.07	0.16	0.30	0.03	0.04	0.06	0.09	0.06	0.17	0.51	0.84
613.8	289	234338	0.03	0.05	0.09	0.14	0.03	0.05	0.10	0.16	0.03	0.03	0.05	0.06	0.05	0.10	0.27	0.53
614	5602	116931	0.07	0.25	0.73	0.97	0.08	0.29	0.80	0.99	0.04	0.09	0.22	0.43	0.21	0.83	1.00	1.00
614.1	2293	116931	0.05	0.13	0.38	0.70	0.06	0.15	0.44	0.78	0.04	0.06	0.12	0.21	0.11	0.47	0.97	1.00
614.3	802	116931	0.04	0.07	0.17	0.32	0.04	0.08	0.19	0.37	0.03	0.04	0.07	0.10	0.07	0.20	0.62	0.92
614.32	352	116931	0.03	0.05	0.10	0.16	0.03	0.05	0.11	0.19	0.03	0.04	0.05	0.07	0.05	0.11	0.32	0.61
614.33	308	116931	0.03	0.05	0.09	0.15	0.03	0.05	0.10	0.17	0.03	0.03	0.05	0.06	0.05	0.10	0.29	0.56
614.4	245	116931	0.03	0.05	0.08	0.13	0.03	0.05	0.09	0.14	0.03	0.03	0.04	0.06	0.04	0.09	0.24	0.47
614.5	2642	116931	0.05	0.14	0.43	0.76	0.06	0.16	0.49	0.83	0.04	0.06	0.13	0.24	0.13	0.53	0.98	1.00
614.51	1116	116931	0.04	0.08	0.21	0.42	0.04	0.09	0.25	0.48	0.03	0.05	0.08	0.13	0.08	0.26	0.76	0.98
614.52	591	116931	0.04	0.06	0.13	0.25	0.04	0.07	0.15	0.29	0.03	0.04	0.06	0.09	0.06	0.16	0.49	0.83
614.53	658	116931	0.04	0.07	0.14	0.27	0.04	0.07	0.16	0.31	0.03	0.04	0.06	0.09	0.06	0.17	0.53	0.87

614.54	228	116931	0.03	0.04	0.08	0.12	0.03	0.05	0.08	0.14	0.03	0.03	0.04	0.06	0.04	0.09	0.22	0.44
615	3495	116931	0.06	0.17	0.53	0.86	0.07	0.20	0.61	0.92	0.04	0.07	0.16	0.30	0.15	0.64	1.00	1.00
617	1252	234338	0.04	0.09	0.23	0.46	0.05	0.10	0.27	0.53	0.03	0.05	0.08	0.14	0.08	0.29	0.80	0.99
618	10694	116931	0.10	0.41	0.93	1.00	0.11	0.47	0.97	1.00	0.05	0.12	0.36	0.68	0.35	0.98	1.00	1.00
618.1	6724	116931	0.08	0.29	0.80	0.99	0.09	0.33	0.86	1.00	0.04	0.09	0.25	0.49	0.25	0.89	1.00	1.00
618.2	4836	116931	0.07	0.22	0.67	0.95	0.08	0.26	0.74	0.97	0.04	0.08	0.20	0.38	0.19	0.78	1.00	1.00
618.5	459	116931	0.04	0.06	0.11	0.20	0.04	0.06	0.13	0.23	0.03	0.04	0.05	0.08	0.05	0.13	0.40	0.73
618.6	629	116931	0.04	0.06	0.14	0.26	0.04	0.07	0.16	0.30	0.03	0.04	0.06	0.09	0.06	0.17	0.51	0.85
619	8298	116931	0.09	0.34	0.87	1.00	0.10	0.39	0.92	1.00	0.05	0.11	0.30	0.57	0.29	0.94	1.00	1.00
619.1	683	116931	0.04	0.07	0.15	0.28	0.04	0.07	0.17	0.32	0.03	0.04	0.06	0.09	0.06	0.18	0.55	0.88
619.2	3020	116931	0.06	0.16	0.48	0.81	0.06	0.18	0.55	0.88	0.04	0.06	0.14	0.26	0.14	0.58	0.99	1.00
619.3	2536	116931	0.05	0.14	0.42	0.75	0.06	0.16	0.48	0.82	0.04	0.06	0.13	0.23	0.12	0.51	0.98	1.00
619.4	1535	116931	0.05	0.10	0.28	0.53	0.05	0.11	0.32	0.61	0.03	0.05	0.09	0.16	0.09	0.34	0.87	1.00
619.5	1209	116931	0.04	0.09	0.23	0.44	0.05	0.10	0.26	0.51	0.03	0.05	0.08	0.13	0.08	0.28	0.79	0.99
621	970	116931	0.04	0.08	0.19	0.37	0.04	0.09	0.22	0.43	0.03	0.04	0.07	0.11	0.07	0.24	0.70	0.96
622	9429	116931	0.10	0.37	0.91	1.00	0.11	0.43	0.95	1.00	0.05	0.12	0.33	0.63	0.32	0.96	1.00	1.00
622.1	6913	116931	0.08	0.29	0.81	0.99	0.09	0.34	0.87	1.00	0.04	0.10	0.26	0.50	0.25	0.90	1.00	1.00
622.2	2951	116931	0.06	0.15	0.47	0.81	0.06	0.18	0.54	0.87	0.04	0.06	0.14	0.26	0.13	0.57	0.99	1.00
623	1089	116931	0.04	0.08	0.21	0.41	0.04	0.09	0.24	0.47	0.03	0.05	0.08	0.12	0.08	0.26	0.75	0.98
624	805	116931	0.04	0.07	0.17	0.32	0.04	0.08	0.19	0.37	0.03	0.04	0.07	0.10	0.07	0.20	0.62	0.92
624.1	254	116931	0.03	0.05	0.08	0.13	0.03	0.05	0.09	0.15	0.03	0.03	0.04	0.06	0.04	0.09	0.25	0.48
624.2	440	116931	0.03	0.06	0.11	0.20	0.04	0.06	0.12	0.22	0.03	0.04	0.05	0.07	0.05	0.13	0.39	0.71
624.9	5074	116931	0.07	0.23	0.69	0.96	0.08	0.27	0.76	0.98	0.04	0.08	0.20	0.40	0.20	0.79	1.00	1.00
625	2526	116931	0.05	0.14	0.41	0.74	0.06	0.16	0.48	0.81	0.04	0.06	0.12	0.23	0.12	0.51	0.98	1.00
625.1	1007	116931	0.04	0.08	0.20	0.38	0.04	0.09	0.23	0.44	0.03	0.04	0.07	0.12	0.07	0.24	0.71	0.97
626	15577	116931	0.13	0.54	0.98	1.00	0.14	0.61	0.99	1.00	0.06	0.16	0.48	0.82	0.46	1.00	1.00	1.00
626.1	13278	116931	0.12	0.48	0.97	1.00	0.13	0.55	0.99	1.00	0.05	0.14	0.43	0.76	0.41	0.99	1.00	1.00
626.12	8228	116931	0.09	0.34	0.87	1.00	0.10	0.39	0.92	1.00	0.05	0.11	0.30	0.57	0.29	0.94	1.00	1.00
626.13	1646	116931	0.05	0.10	0.29	0.56	0.05	0.12	0.34	0.64	0.03	0.05	0.10	0.16	0.09	0.36	0.90	1.00
626.14	3244	116931	0.06	0.16	0.50	0.84	0.06	0.19	0.58	0.90	0.04	0.07	0.15	0.28	0.14	0.61	0.99	1.00
626.2	1487	116931	0.05	0.10	0.27	0.52	0.05	0.11	0.31	0.60	0.03	0.05	0.09	0.15	0.09	0.33	0.86	1.00
626.8	1139	116931	0.04	0.09	0.22	0.42	0.04	0.09	0.25	0.49	0.03	0.05	0.08	0.13	0.08	0.27	0.76	0.98
627	9346	116931	0.10	0.37	0.90	1.00	0.11	0.43	0.95	1.00	0.05	0.11	0.33	0.62	0.32	0.96	1.00	1.00
627.1	7915	116931	0.09	0.33	0.85	0.99	0.10	0.38	0.91	1.00	0.05	0.10	0.29	0.56	0.28	0.93	1.00	1.00
627.3	1078	116931	0.04	0.08	0.21	0.40	0.04	0.09	0.24	0.47	0.03	0.05	0.08	0.12	0.08	0.26	0.74	0.97
627.4	252	116931	0.03	0.05	0.08	0.13	0.03	0.05	0.09	0.15	0.03	0.03	0.04	0.06	0.04	0.09	0.24	0.48
628	4147	116931	0.06	0.20	0.60	0.91	0.07	0.23	0.68	0.95	0.04	0.07	0.18	0.34	0.17	0.71	1.00	1.00
634	4525	169668	0.07	0.21	0.64	0.94	0.07	0.25	0.72	0.97	0.04	0.08	0.19	0.37	0.18	0.75	1.00	1.00
634.1	1038	169668	0.04	0.08	0.20	0.39	0.04	0.09	0.23	0.45	0.03	0.04	0.08	0.12	0.07	0.25	0.73	0.97
634.3	272	169668	0.03	0.05	0.08	0.14	0.03	0.05	0.09	0.16	0.03	0.03	0.05	0.06	0.04	0.10	0.26	0.51
635	1680	169668	0.05	0.11	0.30	0.57	0.05	0.12	0.35	0.65	0.03	0.05	0.10	0.17	0.10	0.37	0.90	1.00
635.2	739	169668	0.04	0.07	0.16	0.30	0.04	0.07	0.18	0.34	0.03	0.04	0.06	0.10	0.06	0.19	0.58	0.90
635.3	1054	169668	0.04	0.08	0.20	0.40	0.04	0.09	0.24	0.46	0.03	0.04	0.08	0.12	0.07	0.25	0.73	0.97
636	2079	169668	0.05	0.12	0.36	0.66	0.05	0.14	0.41	0.74	0.03	0.06	0.11	0.20	0.11	0.44	0.95	1.00
636.2	508	169668	0.04	0.06	0.12	0.22	0.04	0.06	0.14	0.25	0.03	0.04	0.06	0.08	0.05	0.14	0.44	0.77
636.3	872	169668	0.04	0.07	0.18	0.34	0.04	0.08	0.20	0.39	0.03	0.04	0.07	0.11	0.07	0.22	0.65	0.94
642	946	169668	0.04	0.08	0.19	0.36	0.04	0.08	0.22	0.42	0.03	0.04	0.07	0.11	0.07	0.23	0.69	0.96
642.1	259	169668	0.03	0.05	0.08	0.13	0.03	0.05	0.09	0.15	0.03	0.03	0.04	0.06	0.04	0.09	0.25	0.49
644	677	169668	0.04	0.07	0.15	0.28	0.04	0.07	0.17	0.32	0.03	0.04	0.06	0.09	0.06	0.18	0.55	0.87
645	1054	325859	0.04	0.08	0.20	0.40	0.04	0.09	0.24	0.46	0.03	0.04	0.08	0.12	0.07	0.25	0.73	0.97
646	1868	169668	0.05	0.11	0.33	0.62	0.05	0.13	0.38	0.69	0.03	0.05	0.10	0.18	0.10	0.40	0.93	1.00
647.1	226	169668	0.03	0.04	0.08	0.12	0.03	0.05	0.08	0.14	0.03	0.03	0.04	0.06	0.04	0.09	0.22	0.44
650	1568	325859	0.05	0.10	0.28	0.55	0.05	0.11	0.33	0.62	0.03	0.05	0.09	0.16	0.09	0.35	0.88	1.00
651	299	325859	0.03	0.05	0.09	0.15	0.03	0.05	0.10	0.17	0.03	0.03	0.05	0.06	0.05	0.10	0.28	0.54
652	1428	325859	0.04	0.10	0.26	0.51	0.05	0.11	0.30	0.58	0.03	0.05	0.09	0.15	0.09	0.32	0.85	0.99
653	1257	169668	0.04	0.09	0.24	0.46	0.05	0.10	0.27	0.53	0.03	0.05	0.08	0.14	0.08	0.29	0.81	0.99
654.1	1034	169668	0.04	0.08	0.20	0.39	0.04	0.09	0.23	0.45	0.03	0.04	0.08	0.12	0.07	0.25	0.73	0.97
654.2	258	169668	0.03	0.05	0.08	0.13	0.03	0.05	0.09	0.15	0.03	0.03	0.04	0.06	0.04	0.09	0.25	0.48
655	3585	169668	0.06	0.18	0.55	0.88	0.07	0.20	0.62	0.93	0.04	0.07	0.16	0.30	0.16	0.66	1.00	1.00
656	499	325859	0.04	0.06	0.12	0.22	0.04	0.06	0.13	0.25	0.03	0.04	0.06	0.08	0.05	0.14	0.43	0.76
661	1819	325859	0.05	0.11	0.32	0.61	0.05	0.13	0.37	0.69	0.03	0.05	0.10	0.18	0.10	0.40	0.92	1.00
663	511	325859	0.04	0.06	0.12	0.22	0.04	0.06	0.14	0.25	0.03	0.04	0.06	0.08	0.05	0.14	0.44	0.77
665	4394	325859	0.07	0.21	0.64	0.93	0.07	0.24	0.71	0.97	0.04	0.08	0.19	0.36	0.18	0.75	1.00	1.00
669	2212	169668	0.05	0.13	0.37	0.69	0.05	0.14	0.43	0.77	0.04	0.06	0.12	0.21	0.11	0.46	0.96	1.00
674	217	169668	0.03	0.04	0.07	0.12	0.03	0.05	0.08	0.13	0.03	0.03	0.04	0.05	0.04	0.08	0.22	0.42
681	7381	289596	0.09	0.32	0.84	0.99	0.10	0.37	0.90	1.00	0.05	0.10	0.28	0.54	0.27	0.92	1.00	1.00
681.1	550	289596	0.04	0.06	0.13	0.23	0.04	0.07	0.14	0.27	0.03	0.04	0.06	0.08	0.06	0.15	0.46	0.80
681.2	509	289596	0.04	0.06	0.12	0.22	0.04	0.06	0.14	0.25	0.03	0.04	0.06	0.08	0.05	0.14	0.44	0.77
681.3	5521	289596	0.07	0.25	0.73	0.97	0.08	0.29	0.80	0.99	0.04	0.09	0.22	0.44	0.22	0.84	1.00	1.00
681.5	5524	289596	0.07	0.25	0.73	0.97	0.08	0.29	0.80	0.99	0.04	0.09	0.22	0.44	0.22	0.84	1.00	1.00
681.6	5489	289596	0.07	0.25	0.73	0.97	0.08	0.29	0.80	0.99	0.04	0.09	0.22	0.43	0.22	0.83	1.00	1.00
681.7	623	289596	0.04	0.06	0.14	0.26	0.04	0.07	0.16	0.30	0.03	0.04	0.06	0.09	0.06	0.17	0.51	0.85
686	1364	289596	0.04	0.09	0.25	0.49	0.05	0.10	0.29	0.56	0.03	0.05	0.09	0.14	0.09	0.31	0.84	0.99
686.1	2170	289596	0.05	0.13	0.37	0.68	0.05	0.14	0.43	0.76	0.04	0.06	0.11	0.20	0.11	0.46	0.96	1.00

686.3	510	289596	0.04	0.06	0.12	0.22	0.04	0.06	0.14	0.25	0.03	0.04	0.06	0.08	0.05	0.14	0.44	0.77
686.4	337	289596	0.03	0.05	0.09	0.16	0.03	0.05	0.10	0.18	0.03	0.04	0.05	0.07	0.05	0.11	0.31	0.59
687	1302	289596	0.04	0.09	0.24	0.47	0.05	0.10	0.28	0.54	0.03	0.05	0.08	0.14	0.08	0.30	0.82	0.99
687.1	2100	289596	0.05	0.12	0.36	0.67	0.05	0.14	0.42	0.75	0.04	0.06	0.11	0.20	0.11	0.45	0.95	1.00
687.2	947	289596	0.04	0.08	0.19	0.37	0.04	0.09	0.22	0.42	0.03	0.04	0.07	0.11	0.07	0.23	0.69	0.96
687.4	2787	289596	0.06	0.15	0.45	0.79	0.06	0.17	0.52	0.85	0.04	0.06	0.13	0.25	0.13	0.55	0.99	1.00
689	5767	289596	0.08	0.26	0.75	0.98	0.08	0.30	0.82	0.99	0.04	0.09	0.23	0.45	0.22	0.85	1.00	1.00
690	3042	289596	0.06	0.16	0.49	0.82	0.06	0.18	0.56	0.88	0.04	0.07	0.14	0.27	0.14	0.59	0.99	1.00
690.1	3024	289596	0.06	0.16	0.48	0.82	0.06	0.18	0.55	0.88	0.04	0.06	0.14	0.27	0.14	0.59	0.99	1.00
694	952	289596	0.04	0.08	0.19	0.37	0.04	0.09	0.22	0.42	0.03	0.04	0.07	0.11	0.07	0.23	0.69	0.96
694.2	762	289596	0.04	0.07	0.16	0.30	0.04	0.08	0.18	0.35	0.03	0.04	0.07	0.10	0.06	0.20	0.60	0.91
695	2344	289596	0.05	0.13	0.39	0.72	0.06	0.15	0.45	0.79	0.04	0.06	0.12	0.22	0.12	0.49	0.97	1.00
695.3	312	289596	0.03	0.05	0.09	0.15	0.03	0.05	0.10	0.17	0.03	0.03	0.05	0.06	0.05	0.10	0.29	0.56
695.4	391	288757	0.03	0.05	0.10	0.18	0.04	0.06	0.11	0.20	0.03	0.04	0.05	0.07	0.05	0.12	0.35	0.66
695.42	324	288757	0.03	0.05	0.09	0.16	0.03	0.05	0.10	0.18	0.03	0.03	0.05	0.06	0.05	0.11	0.30	0.58
695.7	724	289596	0.04	0.07	0.15	0.29	0.04	0.07	0.18	0.34	0.03	0.04	0.06	0.10	0.06	0.19	0.57	0.90
695.8	231	289596	0.03	0.05	0.08	0.12	0.03	0.05	0.08	0.14	0.03	0.03	0.04	0.06	0.04	0.09	0.23	0.44
695.9	486	289596	0.04	0.06	0.12	0.21	0.04	0.06	0.13	0.24	0.03	0.04	0.05	0.08	0.05	0.14	0.42	0.75
696	2369	286399	0.05	0.13	0.40	0.72	0.06	0.15	0.46	0.80	0.04	0.06	0.12	0.22	0.12	0.49	0.97	1.00
696.4	2308	286399	0.05	0.13	0.39	0.71	0.06	0.15	0.45	0.78	0.04	0.06	0.12	0.21	0.12	0.48	0.97	1.00
696.41	1756	286399	0.05	0.11	0.31	0.59	0.05	0.12	0.36	0.67	0.03	0.05	0.10	0.17	0.10	0.39	0.92	1.00
696.42	711	286399	0.04	0.07	0.15	0.29	0.04	0.07	0.17	0.33	0.03	0.04	0.06	0.10	0.06	0.18	0.57	0.89
697	522	289596	0.04	0.06	0.12	0.22	0.04	0.06	0.14	0.26	0.03	0.04	0.06	0.08	0.06	0.15	0.45	0.78
698	767	289596	0.04	0.07	0.16	0.31	0.04	0.08	0.18	0.36	0.03	0.04	0.07	0.10	0.06	0.20	0.60	0.91
700	280	289596	0.03	0.05	0.08	0.14	0.03	0.05	0.09	0.16	0.03	0.03	0.05	0.06	0.05	0.10	0.27	0.52
701	4868	289596	0.07	0.23	0.68	0.95	0.08	0.26	0.75	0.98	0.04	0.08	0.20	0.39	0.20	0.79	1.00	1.00
701.2	2159	289596	0.05	0.13	0.37	0.68	0.05	0.14	0.43	0.76	0.04	0.06	0.11	0.20	0.11	0.46	0.96	1.00
701.3	478	289596	0.04	0.06	0.12	0.21	0.04	0.06	0.13	0.24	0.03	0.04	0.05	0.08	0.05	0.14	0.41	0.74
701.4	205	289596	0.03	0.04	0.07	0.11	0.03	0.05	0.08	0.13	0.03	0.03	0.04	0.05	0.04	0.08	0.21	0.40
701.5	447	289596	0.04	0.06	0.11	0.20	0.04	0.06	0.12	0.23	0.03	0.04	0.05	0.07	0.05	0.13	0.39	0.72
702	5494	289596	0.07	0.25	0.73	0.97	0.08	0.29	0.80	0.99	0.04	0.09	0.22	0.43	0.22	0.83	1.00	1.00
702.1	2726	289596	0.06	0.15	0.44	0.78	0.06	0.17	0.51	0.85	0.04	0.06	0.13	0.24	0.13	0.55	0.99	1.00
702.2	2973	289596	0.06	0.16	0.48	0.81	0.06	0.18	0.55	0.88	0.04	0.06	0.14	0.26	0.14	0.58	0.99	1.00
703	345	289596	0.03	0.05	0.10	0.16	0.03	0.05	0.11	0.19	0.03	0.04	0.05	0.07	0.05	0.11	0.32	0.60
703.1	837	289596	0.04	0.07	0.17	0.33	0.04	0.08	0.20	0.38	0.03	0.04	0.07	0.10	0.07	0.21	0.64	0.93
704	4724	289596	0.07	0.22	0.67	0.95	0.08	0.26	0.74	0.97	0.04	0.08	0.20	0.38	0.19	0.78	1.00	1.00
705.8	569	289596	0.04	0.06	0.13	0.24	0.04	0.07	0.15	0.28	0.03	0.04	0.06	0.08	0.06	0.16	0.48	0.81
706	7950	289596	0.09	0.34	0.87	1.00	0.10	0.39	0.92	1.00	0.05	0.11	0.30	0.57	0.29	0.94	1.00	1.00
706.2	7878	289596	0.09	0.34	0.87	1.00	0.10	0.39	0.92	1.00	0.05	0.11	0.30	0.57	0.29	0.94	1.00	1.00
707	2310	289596	0.05	0.13	0.39	0.71	0.06	0.15	0.45	0.79	0.04	0.06	0.12	0.21	0.12	0.48	0.97	1.00
707.1	1184	289596	0.04	0.09	0.22	0.44	0.04	0.10	0.26	0.50	0.03	0.05	0.08	0.13	0.08	0.28	0.78	0.98
707.2	1199	289596	0.04	0.09	0.23	0.44	0.05	0.10	0.26	0.51	0.03	0.05	0.08	0.13	0.08	0.28	0.79	0.99
709	3306	289596	0.06	0.17	0.52	0.85	0.06	0.19	0.59	0.91	0.04	0.07	0.15	0.29	0.15	0.63	1.00	1.00
709.2	512	289596	0.04	0.06	0.12	0.22	0.04	0.06	0.14	0.25	0.03	0.04	0.06	0.08	0.05	0.15	0.44	0.77
709.7	2592	289596	0.05	0.14	0.43	0.76	0.06	0.16	0.49	0.83	0.04	0.06	0.13	0.23	0.12	0.53	0.98	1.00
710	620	295120	0.04	0.06	0.14	0.26	0.04	0.07	0.16	0.30	0.03	0.04	0.06	0.09	0.06	0.17	0.51	0.85
710.1	602	295120	0.04	0.06	0.14	0.25	0.04	0.07	0.15	0.29	0.03	0.04	0.06	0.09	0.06	0.16	0.50	0.84
710.19	444	295120	0.03	0.06	0.11	0.20	0.04	0.06	0.12	0.23	0.03	0.04	0.05	0.07	0.05	0.13	0.39	0.71
711	433	295120	0.03	0.06	0.11	0.19	0.04	0.06	0.12	0.22	0.03	0.04	0.05	0.07	0.05	0.13	0.38	0.70
711.1	333	295120	0.03	0.05	0.09	0.16	0.03	0.05	0.10	0.18	0.03	0.04	0.05	0.06	0.05	0.11	0.31	0.59
714	4896	294409	0.07	0.23	0.68	0.95	0.08	0.26	0.76	0.98	0.04	0.08	0.20	0.40	0.20	0.79	1.00	1.00
714.1	4369	294409	0.07	0.21	0.63	0.93	0.07	0.24	0.71	0.96	0.04	0.08	0.19	0.36	0.18	0.75	1.00	1.00
715	1641	294409	0.05	0.11	0.29	0.57	0.05	0.12	0.34	0.64	0.03	0.05	0.10	0.17	0.09	0.36	0.90	1.00
715.2	399	294409	0.03	0.05	0.10	0.18	0.04	0.06	0.12	0.21	0.03	0.04	0.05	0.07	0.05	0.12	0.36	0.67
716	38203	295120	0.25	0.90	1.00	1.00	0.29	0.94	1.00	1.00	0.09	0.32	0.85	0.99	0.84	1.00	1.00	1.00
716.2	16046	295120	0.14	0.58	0.99	1.00	0.15	0.66	1.00	1.00	0.06	0.17	0.52	0.85	0.50	1.00	1.00	1.00
716.9	38053	295120	0.25	0.90	1.00	1.00	0.29	0.94	1.00	1.00	0.09	0.32	0.85	0.99	0.83	1.00	1.00	1.00
717	1354	336121	0.04	0.09	0.25	0.49	0.05	0.10	0.29	0.56	0.03	0.05	0.09	0.14	0.08	0.31	0.83	0.99
720	4105	322347	0.07	0.20	0.61	0.92	0.07	0.23	0.68	0.96	0.04	0.07	0.18	0.34	0.17	0.72	1.00	1.00
721	8311	322347	0.09	0.35	0.88	1.00	0.10	0.41	0.93	1.00	0.05	0.11	0.31	0.59	0.30	0.95	1.00	1.00
721.1	7917	322347	0.09	0.34	0.87	1.00	0.10	0.39	0.92	1.00	0.05	0.11	0.30	0.57	0.29	0.94	1.00	1.00
721.2	204	322347	0.03	0.04	0.07	0.11	0.03	0.05	0.08	0.13	0.03	0.03	0.04	0.05	0.04	0.08	0.21	0.40
721.8	384	322347	0.03	0.05	0.10	0.18	0.04	0.06	0.11	0.20	0.03	0.04	0.05	0.07	0.05	0.12	0.35	0.65
722	6764	322347	0.08	0.30	0.81	0.99	0.09	0.34	0.88	1.00	0.05	0.10	0.26	0.51	0.25	0.90	1.00	1.00
722.1	495	322347	0.04	0.06	0.12	0.21	0.04	0.06	0.13	0.25	0.03	0.04	0.05	0.08	0.05	0.14	0.43	0.76
722.6	2865	322347	0.06	0.15	0.46	0.80	0.06	0.17	0.53	0.86	0.04	0.06	0.14	0.25	0.13	0.57	0.99	1.00
722.7	398	322347	0.03	0.05	0.10	0.18	0.04	0.06	0.12	0.21	0.03	0.04	0.05	0.07	0.05	0.12	0.36	0.67
722.9	4097	322347	0.06	0.20	0.61	0.92	0.07	0.23	0.68	0.95	0.04	0.07	0.18	0.34	0.17	0.72	1.00	1.00
723	537	322347	0.04	0.06	0.13	0.23	0.04	0.06	0.14	0.26	0.03	0.04	0.06	0.08	0.06	0.15	0.46	0.79
724	1156	322347	0.04	0.09	0.22	0.43	0.04	0.10	0.25	0.50	0.03	0.05	0.08	0.13	0.08	0.27	0.77	0.98
724.1	358	322347	0.03	0.05	0.10	0.17	0.03	0.06	0.11	0.19	0.03	0.04	0.05	0.07	0.05	0.11	0.33	0.62
724.2	361	322347	0.03	0.05	0.10	0.17	0.03	0.06	0.11	0.19	0.03	0.04	0.05	0.07	0.05	0.11	0.33	0.62
724.9	723	322347	0.04	0.07	0.15	0.29	0.04	0.07	0.18	0.34	0.03	0.04	0.06	0.10	0.06	0.19	0.57	0.90
726	14369	306383	0.13	0.54	0.98	1.00	0.14	0.61	0.99	1.00	0.06	0.16	0.48	0.82	0.46	1.00	1.00	1.00

726.1	9643	306383	0.10	0.40	0.92	1.00	0.11	0.46	0.96	1.00	0.05	0.12	0.35	0.66	0.34	0.97	1.00	1.00
726.2	527	306383	0.04	0.06	0.12	0.23	0.04	0.06	0.14	0.26	0.03	0.04	0.06	0.08	0.06	0.15	0.45	0.78
726.3	822	306383	0.04	0.07	0.17	0.32	0.04	0.08	0.19	0.38	0.03	0.04	0.07	0.10	0.07	0.21	0.63	0.93
726.4	3462	306383	0.06	0.17	0.54	0.87	0.07	0.20	0.61	0.92	0.04	0.07	0.16	0.30	0.15	0.65	1.00	1.00
727	12755	306383	0.12	0.49	0.97	1.00	0.13	0.56	0.99	1.00	0.05	0.14	0.44	0.77	0.42	0.99	1.00	1.00
727.1	5004	306383	0.07	0.23	0.69	0.96	0.08	0.27	0.77	0.98	0.04	0.08	0.21	0.40	0.20	0.80	1.00	1.00
727.4	2903	306383	0.06	0.15	0.47	0.80	0.06	0.18	0.54	0.87	0.04	0.06	0.14	0.26	0.13	0.57	0.99	1.00
727.5	587	306383	0.04	0.06	0.13	0.25	0.04	0.07	0.15	0.28	0.03	0.04	0.06	0.09	0.06	0.16	0.49	0.83
727.6	3877	306383	0.06	0.19	0.58	0.90	0.07	0.22	0.66	0.94	0.04	0.07	0.17	0.33	0.17	0.69	1.00	1.00
728	832	306383	0.04	0.07	0.17	0.33	0.04	0.08	0.20	0.38	0.03	0.04	0.07	0.10	0.07	0.21	0.63	0.93
728.2	402	306383	0.03	0.05	0.10	0.18	0.04	0.06	0.12	0.21	0.03	0.04	0.05	0.07	0.05	0.12	0.36	0.67
728.7	3729	306383	0.06	0.19	0.57	0.89	0.07	0.21	0.64	0.94	0.04	0.07	0.17	0.32	0.16	0.68	1.00	1.00
728.71	3406	306383	0.06	0.17	0.53	0.86	0.06	0.20	0.60	0.92	0.04	0.07	0.15	0.29	0.15	0.64	1.00	1.00
729	6003	306383	0.08	0.27	0.77	0.98	0.08	0.31	0.84	0.99	0.04	0.09	0.24	0.47	0.23	0.86	1.00	1.00
729.1	835	306383	0.04	0.07	0.17	0.33	0.04	0.08	0.20	0.38	0.03	0.04	0.07	0.10	0.07	0.21	0.63	0.93
731	290	320016	0.03	0.05	0.09	0.14	0.03	0.05	0.10	0.16	0.03	0.03	0.05	0.06	0.05	0.10	0.27	0.53
731.1	239	320016	0.03	0.05	0.08	0.13	0.03	0.05	0.09	0.14	0.03	0.03	0.04	0.06	0.04	0.09	0.23	0.46
732	461	320016	0.04	0.06	0.11	0.20	0.04	0.06	0.13	0.23	0.03	0.04	0.05	0.08	0.05	0.13	0.40	0.73
732.1	205	320016	0.03	0.04	0.07	0.11	0.03	0.05	0.08	0.13	0.03	0.03	0.04	0.05	0.04	0.08	0.21	0.40
733	2100	320016	0.05	0.12	0.36	0.67	0.05	0.14	0.42	0.75	0.04	0.06	0.11	0.20	0.11	0.45	0.95	1.00
733.2	295	320016	0.03	0.05	0.09	0.15	0.03	0.05	0.10	0.17	0.03	0.03	0.05	0.06	0.05	0.10	0.28	0.54
733.4	518	320016	0.04	0.06	0.12	0.22	0.04	0.06	0.14	0.26	0.03	0.04	0.06	0.08	0.06	0.15	0.44	0.78
733.8	1175	320016	0.04	0.09	0.22	0.44	0.04	0.10	0.26	0.50	0.03	0.05	0.08	0.13	0.08	0.28	0.78	0.98
735	8929	324005	0.10	0.37	0.90	1.00	0.11	0.43	0.95	1.00	0.05	0.11	0.33	0.62	0.32	0.96	1.00	1.00
735.1	215	324005	0.03	0.04	0.07	0.12	0.03	0.05	0.08	0.13	0.03	0.03	0.04	0.05	0.04	0.08	0.21	0.42
735.2	4749	324005	0.07	0.22	0.67	0.95	0.08	0.26	0.75	0.98	0.04	0.08	0.20	0.39	0.19	0.78	1.00	1.00
735.21	1813	324005	0.05	0.11	0.32	0.61	0.05	0.13	0.37	0.68	0.03	0.05	0.10	0.18	0.10	0.40	0.92	1.00
735.23	1423	324005	0.04	0.10	0.26	0.51	0.05	0.11	0.30	0.58	0.03	0.05	0.09	0.15	0.09	0.32	0.85	0.99
735.3	6000	324005	0.08	0.27	0.77	0.98	0.08	0.31	0.84	0.99	0.04	0.09	0.24	0.47	0.23	0.87	1.00	1.00
736	1764	324005	0.05	0.11	0.31	0.60	0.05	0.12	0.36	0.67	0.03	0.05	0.10	0.17	0.10	0.39	0.92	1.00
736.2	234	324005	0.03	0.05	0.08	0.12	0.03	0.05	0.08	0.14	0.03	0.03	0.04	0.06	0.04	0.09	0.23	0.45
737	1419	324005	0.04	0.10	0.26	0.51	0.05	0.11	0.30	0.58	0.03	0.05	0.09	0.15	0.09	0.32	0.85	0.99
737.1	213	324005	0.03	0.04	0.07	0.12	0.03	0.05	0.08	0.13	0.03	0.03	0.04	0.05	0.04	0.08	0.21	0.42
737.3	1174	324005	0.04	0.09	0.22	0.44	0.04	0.10	0.26	0.50	0.03	0.05	0.08	0.13	0.08	0.28	0.78	0.98
738	1991	324005	0.05	0.12	0.34	0.65	0.05	0.13	0.40	0.72	0.03	0.06	0.11	0.19	0.11	0.43	0.94	1.00
738.4	1644	324005	0.05	0.11	0.29	0.57	0.05	0.12	0.34	0.64	0.03	0.05	0.10	0.17	0.09	0.36	0.90	1.00
739	496	324005	0.04	0.06	0.12	0.21	0.04	0.06	0.13	0.25	0.03	0.04	0.06	0.08	0.05	0.14	0.43	0.76
740	50335	287140	0.31	0.95	1.00	1.00	0.35	0.98	1.00	1.00	0.10	0.39	0.92	1.00	0.91	1.00	1.00	1.00
740.1	31034	287140	0.22	0.83	1.00	1.00	0.25	0.89	1.00	1.00	0.08	0.27	0.78	0.98	0.76	1.00	1.00	1.00
740.11	8866	287140	0.10	0.37	0.90	1.00	0.11	0.43	0.94	1.00	0.05	0.11	0.33	0.62	0.32	0.96	1.00	1.00
740.12	634	287140	0.04	0.06	0.14	0.26	0.04	0.07	0.16	0.30	0.03	0.04	0.06	0.09	0.06	0.17	0.52	0.85
740.2	4353	287140	0.07	0.21	0.63	0.93	0.07	0.24	0.71	0.96	0.04	0.08	0.19	0.36	0.18	0.74	1.00	1.00
740.9	28265	287140	0.20	0.80	1.00	1.00	0.23	0.87	1.00	1.00	0.08	0.26	0.74	0.97	0.73	1.00	1.00	1.00
741	3744	331152	0.06	0.19	0.57	0.89	0.07	0.21	0.64	0.94	0.04	0.07	0.17	0.32	0.16	0.68	1.00	1.00
741.2	590	331152	0.04	0.06	0.13	0.25	0.04	0.07	0.15	0.29	0.03	0.04	0.06	0.09	0.06	0.16	0.49	0.83
741.4	1400	331152	0.04	0.10	0.26	0.50	0.05	0.11	0.30	0.57	0.03	0.05	0.09	0.15	0.09	0.32	0.85	0.99
742	2856	331152	0.06	0.15	0.46	0.80	0.06	0.17	0.53	0.86	0.04	0.06	0.14	0.25	0.13	0.57	0.99	1.00
742.1	297	331152	0.03	0.05	0.09	0.15	0.03	0.05	0.10	0.17	0.03	0.03	0.05	0.06	0.05	0.10	0.28	0.54
742.2	383	331152	0.03	0.05	0.10	0.18	0.04	0.06	0.11	0.20	0.03	0.04	0.05	0.07	0.05	0.12	0.35	0.65
742.8	970	331152	0.04	0.08	0.19	0.37	0.04	0.09	0.22	0.43	0.03	0.04	0.07	0.11	0.07	0.24	0.70	0.96
742.9	2384	331152	0.05	0.13	0.40	0.72	0.06	0.15	0.46	0.80	0.04	0.06	0.12	0.22	0.12	0.49	0.97	1.00
743	7552	329923	0.09	0.32	0.85	0.99	0.10	0.38	0.91	1.00	0.05	0.10	0.29	0.55	0.28	0.93	1.00	1.00
743.1	6241	329923	0.08	0.28	0.78	0.98	0.09	0.32	0.85	0.99	0.04	0.09	0.25	0.48	0.24	0.88	1.00	1.00
743.11	6220	329923	0.08	0.28	0.78	0.98	0.09	0.32	0.85	0.99	0.04	0.09	0.25	0.48	0.24	0.88	1.00	1.00
743.2	571	329923	0.04	0.06	0.13	0.24	0.04	0.07	0.15	0.28	0.03	0.04	0.06	0.08	0.06	0.16	0.48	0.82
743.21	325	329923	0.03	0.05	0.09	0.16	0.03	0.05	0.10	0.18	0.03	0.03	0.05	0.06	0.05	0.11	0.30	0.58
743.9	836	329923	0.04	0.07	0.17	0.33	0.04	0.08	0.20	0.38	0.03	0.04	0.07	0.10	0.07	0.21	0.64	0.93
745	7733	329742	0.09	0.33	0.86	1.00	0.10	0.38	0.91	1.00	0.05	0.11	0.29	0.56	0.28	0.93	1.00	1.00
747	3002	329064	0.06	0.16	0.48	0.82	0.06	0.18	0.55	0.88	0.04	0.06	0.14	0.26	0.14	0.59	0.99	1.00
747.1	2826	329064	0.06	0.15	0.46	0.79	0.06	0.17	0.53	0.86	0.04	0.06	0.14	0.25	0.13	0.56	0.99	1.00
747.11	576	329064	0.04	0.06	0.13	0.24	0.04	0.07	0.15	0.28	0.03	0.04	0.06	0.08	0.06	0.16	0.48	0.82
747.12	270	329064	0.03	0.05	0.08	0.14	0.03	0.05	0.09	0.15	0.03	0.03	0.05	0.06	0.04	0.10	0.26	0.50
747.13	2015	329064	0.05	0.12	0.35	0.65	0.05	0.13	0.40	0.73	0.03	0.06	0.11	0.19	0.11	0.43	0.95	1.00
749	295	329064	0.03	0.05	0.09	0.15	0.03	0.05	0.10	0.17	0.03	0.03	0.05	0.06	0.05	0.10	0.28	0.54
750	706	329064	0.04	0.07	0.15	0.29	0.04	0.07	0.17	0.33	0.03	0.04	0.06	0.09	0.06	0.18	0.56	0.89
750.1	351	329064	0.03	0.05	0.10	0.16	0.03	0.05	0.11	0.19	0.03	0.04	0.05	0.07	0.05	0.11	0.32	0.61
750.13	204	329064	0.03	0.04	0.07	0.11	0.03	0.05	0.08	0.13	0.03	0.03	0.04	0.05	0.04	0.08	0.21	0.40
750.2	348	329064	0.03	0.05	0.10	0.16	0.03	0.05	0.11	0.19	0.03	0.04	0.05	0.07	0.05	0.11	0.32	0.61
750.21	222	329064	0.03	0.04	0.08	0.12	0.03	0.05	0.08	0.13	0.03	0.03	0.04	0.06	0.04	0.09	0.22	0.43
751	1465	329064	0.05	0.10	0.27	0.52	0.05	0.11	0.31	0.59	0.03	0.05	0.09	0.15	0.09	0.33	0.86	1.00
751.1	633	329064	0.04	0.06	0.14	0.26	0.04	0.07	0.16	0.30	0.03	0.04	0.06	0.09	0.06	0.17	0.52	0.85
751.11	392	176821	0.03	0.05	0.10	0.18	0.04	0.06	0.11	0.20	0.03	0.04	0.05	0.07	0.05	0.12	0.35	0.66
751.12	205	152243	0.03	0.04	0.07	0.11	0.03	0.05	0.08	0.13	0.03	0.03	0.04	0.05	0.04	0.08	0.21	0.40
751.2	801	329064	0.04	0.07	0.17	0.32	0.04	0.08	0.19	0.37	0.03	0.04	0.07	0.10	0.07	0.20	0.62	0.92

751.21	394	329064	0.03	0.05	0.10	0.18	0.04	0.06	0.11	0.21	0.03	0.04	0.05	0.07	0.05	0.12	0.35	0.66
751.22	333	329064	0.03	0.05	0.09	0.16	0.03	0.05	0.10	0.18	0.03	0.04	0.05	0.06	0.05	0.11	0.31	0.59
752	1689	329064	0.05	0.11	0.30	0.58	0.05	0.12	0.35	0.65	0.03	0.05	0.10	0.17	0.10	0.37	0.91	1.00
752.1	1613	329064	0.05	0.10	0.29	0.56	0.05	0.12	0.34	0.63	0.03	0.05	0.10	0.16	0.09	0.36	0.89	1.00
754	1491	329064	0.05	0.10	0.27	0.53	0.05	0.11	0.31	0.60	0.03	0.05	0.09	0.15	0.09	0.34	0.87	1.00
755	432	329064	0.03	0.06	0.11	0.19	0.04	0.06	0.12	0.22	0.03	0.04	0.05	0.07	0.05	0.13	0.38	0.70
755.1	222	329064	0.03	0.04	0.08	0.12	0.03	0.05	0.08	0.13	0.03	0.03	0.04	0.06	0.04	0.09	0.22	0.43
756	590	329064	0.04	0.06	0.13	0.25	0.04	0.07	0.15	0.29	0.03	0.04	0.06	0.09	0.06	0.16	0.49	0.83
756.5	425	329064	0.03	0.05	0.11	0.19	0.04	0.06	0.12	0.22	0.03	0.04	0.05	0.07	0.05	0.13	0.38	0.69
759	223	329064	0.03	0.04	0.08	0.12	0.03	0.05	0.08	0.14	0.03	0.03	0.04	0.06	0.04	0.09	0.22	0.43
760	10889	257697	0.11	0.43	0.95	1.00	0.12	0.50	0.97	1.00	0.05	0.13	0.38	0.70	0.37	0.98	1.00	1.00
761	1764	257697	0.05	0.11	0.31	0.60	0.05	0.12	0.36	0.67	0.03	0.05	0.10	0.17	0.10	0.39	0.92	1.00
764	2339	257697	0.05	0.13	0.39	0.72	0.06	0.15	0.45	0.79	0.04	0.06	0.12	0.22	0.12	0.48	0.97	1.00
765	505	257697	0.04	0.06	0.12	0.22	0.04	0.06	0.14	0.25	0.03	0.04	0.06	0.08	0.05	0.14	0.43	0.77
766	1265	257697	0.04	0.09	0.24	0.46	0.05	0.10	0.27	0.53	0.03	0.05	0.08	0.14	0.08	0.29	0.81	0.99
770	1789	257697	0.05	0.11	0.31	0.60	0.05	0.12	0.36	0.68	0.03	0.05	0.10	0.18	0.10	0.39	0.92	1.00
771	776	257697	0.04	0.07	0.16	0.31	0.04	0.08	0.19	0.36	0.03	0.04	0.07	0.10	0.06	0.20	0.60	0.91
771.1	4680	257697	0.07	0.22	0.66	0.95	0.07	0.25	0.74	0.97	0.04	0.08	0.20	0.38	0.19	0.77	1.00	1.00
772	676	257697	0.04	0.07	0.15	0.28	0.04	0.07	0.17	0.32	0.03	0.04	0.06	0.09	0.06	0.18	0.54	0.87
773	5984	257697	0.08	0.27	0.76	0.98	0.08	0.31	0.83	0.99	0.04	0.09	0.24	0.46	0.23	0.86	1.00	1.00
780	212	257697	0.03	0.04	0.07	0.12	0.03	0.05	0.08	0.13	0.03	0.03	0.04	0.05	0.04	0.08	0.21	0.41
781	9086	257697	0.10	0.38	0.91	1.00	0.11	0.43	0.95	1.00	0.05	0.12	0.33	0.63	0.32	0.96	1.00	1.00
782.3	1941	257697	0.05	0.12	0.34	0.64	0.05	0.13	0.39	0.71	0.03	0.05	0.11	0.19	0.10	0.42	0.94	1.00
783	3998	257697	0.06	0.19	0.59	0.91	0.07	0.22	0.67	0.95	0.04	0.07	0.17	0.33	0.17	0.71	1.00	1.00
785	37426	257697	0.24	0.89	1.00	1.00	0.28	0.93	1.00	1.00	0.09	0.31	0.84	0.99	0.82	1.00	1.00	1.00
788	9030	257697	0.10	0.37	0.91	1.00	0.11	0.43	0.95	1.00	0.05	0.12	0.33	0.63	0.32	0.96	1.00	1.00
789	11420	257697	0.11	0.45	0.96	1.00	0.12	0.52	0.98	1.00	0.05	0.13	0.40	0.72	0.39	0.99	1.00	1.00
790.6	6857	257697	0.08	0.30	0.82	0.99	0.09	0.35	0.88	1.00	0.05	0.10	0.26	0.51	0.26	0.90	1.00	1.00
791	645	257697	0.04	0.06	0.14	0.27	0.04	0.07	0.16	0.31	0.03	0.04	0.06	0.09	0.06	0.17	0.53	0.86
793.2	608	257697	0.04	0.06	0.14	0.25	0.04	0.07	0.15	0.29	0.03	0.04	0.06	0.09	0.06	0.16	0.50	0.84
798	3438	257697	0.06	0.17	0.53	0.86	0.07	0.20	0.61	0.92	0.04	0.07	0.16	0.30	0.15	0.64	1.00	1.00
798.1	607	257697	0.04	0.06	0.14	0.25	0.04	0.07	0.15	0.29	0.03	0.04	0.06	0.09	0.06	0.16	0.50	0.84
800	6862	243043	0.08	0.30	0.82	0.99	0.09	0.35	0.88	1.00	0.05	0.10	0.26	0.51	0.26	0.90	1.00	1.00
800.1	1926	243043	0.05	0.12	0.33	0.63	0.05	0.13	0.39	0.71	0.03	0.05	0.11	0.19	0.10	0.41	0.94	1.00
800.2	888	243043	0.04	0.08	0.18	0.35	0.04	0.08	0.21	0.40	0.03	0.04	0.07	0.11	0.07	0.22	0.66	0.95
800.3	1976	243043	0.05	0.12	0.34	0.64	0.05	0.13	0.40	0.72	0.03	0.05	0.11	0.19	0.11	0.42	0.94	1.00
800.4	689	243043	0.04	0.07	0.15	0.28	0.04	0.07	0.17	0.32	0.03	0.04	0.06	0.09	0.06	0.18	0.55	0.88
801	1992	243043	0.05	0.12	0.34	0.65	0.05	0.13	0.40	0.72	0.03	0.05	0.11	0.19	0.11	0.43	0.94	1.00
801.1	1220	243043	0.04	0.09	0.23	0.45	0.05	0.10	0.27	0.52	0.03	0.05	0.08	0.13	0.08	0.28	0.79	0.99
802	759	243043	0.04	0.07	0.16	0.30	0.04	0.08	0.18	0.35	0.03	0.04	0.07	0.10	0.06	0.19	0.59	0.91
803	8356	243043	0.09	0.35	0.88	1.00	0.10	0.41	0.93	1.00	0.05	0.11	0.31	0.59	0.30	0.95	1.00	1.00
803.1	1610	243043	0.05	0.10	0.29	0.56	0.05	0.12	0.33	0.63	0.03	0.05	0.10	0.16	0.09	0.36	0.89	1.00
803.2	5165	243043	0.07	0.24	0.70	0.96	0.08	0.27	0.78	0.98	0.04	0.08	0.21	0.41	0.20	0.81	1.00	1.00
803.3	1581	243043	0.05	0.10	0.28	0.55	0.05	0.11	0.33	0.62	0.03	0.05	0.09	0.16	0.09	0.35	0.89	1.00
804	3156	243043	0.06	0.16	0.50	0.84	0.06	0.19	0.57	0.89	0.04	0.07	0.15	0.28	0.14	0.61	0.99	1.00
805	1685	243043	0.05	0.11	0.30	0.58	0.05	0.12	0.35	0.65	0.03	0.05	0.10	0.17	0.10	0.37	0.90	1.00
807	1555	243043	0.05	0.10	0.28	0.54	0.05	0.11	0.32	0.62	0.03	0.05	0.09	0.16	0.09	0.35	0.88	1.00
809	10448	243043	0.10	0.42	0.94	1.00	0.12	0.48	0.97	1.00	0.05	0.13	0.37	0.69	0.36	0.98	1.00	1.00
817	219	243043	0.03	0.04	0.07	0.12	0.03	0.05	0.08	0.13	0.03	0.03	0.04	0.05	0.04	0.09	0.22	0.43
818	667	243043	0.04	0.07	0.15	0.27	0.04	0.07	0.17	0.32	0.03	0.04	0.06	0.09	0.06	0.18	0.54	0.87
819	2986	243043	0.06	0.16	0.48	0.81	0.06	0.18	0.55	0.88	0.04	0.06	0.14	0.26	0.14	0.58	0.99	1.00
830	1954	243043	0.05	0.12	0.34	0.64	0.05	0.13	0.39	0.72	0.03	0.05	0.11	0.19	0.10	0.42	0.94	1.00
835	13967	243043	0.12	0.52	0.98	1.00	0.14	0.59	0.99	1.00	0.06	0.15	0.46	0.80	0.45	1.00	1.00	1.00
836	498	243043	0.04	0.06	0.12	0.22	0.04	0.06	0.13	0.25	0.03	0.04	0.06	0.08	0.05	0.14	0.43	0.76
840	866	243043	0.04	0.07	0.18	0.34	0.04	0.08	0.20	0.39	0.03	0.04	0.07	0.11	0.07	0.22	0.65	0.94
840.3	457	243043	0.04	0.06	0.11	0.20	0.04	0.06	0.13	0.23	0.03	0.04	0.05	0.08	0.05	0.13	0.40	0.72
850	5032	243043	0.07	0.23	0.69	0.96	0.08	0.27	0.77	0.98	0.04	0.08	0.21	0.40	0.20	0.80	1.00	1.00
851	17270	243043	0.14	0.60	0.99	1.00	0.16	0.68	1.00	1.00	0.06	0.18	0.54	0.87	0.53	1.00	1.00	1.00
853	494	243043	0.04	0.06	0.12	0.21	0.04	0.06	0.13	0.25	0.03	0.04	0.05	0.08	0.05	0.14	0.43	0.76
854	2535	243043	0.05	0.14	0.42	0.75	0.06	0.16	0.48	0.82	0.04	0.06	0.13	0.23	0.12	0.52	0.98	1.00
857	1436	243043	0.05	0.10	0.26	0.51	0.05	0.11	0.30	0.58	0.03	0.05	0.09	0.15	0.09	0.33	0.85	0.99
858	3502	243043	0.06	0.18	0.54	0.87	0.07	0.20	0.61	0.92	0.04	0.07	0.16	0.30	0.15	0.65	1.00	1.00
859	3509	243043	0.06	0.18	0.54	0.87	0.07	0.20	0.61	0.92	0.04	0.07	0.16	0.30	0.15	0.65	1.00	1.00
870	3784	243043	0.06	0.19	0.57	0.89	0.07	0.21	0.65	0.94	0.04	0.07	0.17	0.32	0.16	0.68	1.00	1.00
870.1	522	243043	0.04	0.06	0.12	0.22	0.04	0.06	0.14	0.26	0.03	0.04	0.06	0.08	0.06	0.15	0.45	0.78
870.3	2938	243043	0.06	0.15	0.47	0.81	0.06	0.18	0.54	0.87	0.04	0.06	0.14	0.26	0.14	0.58	0.99	1.00
870.4	247	243043	0.03	0.05	0.08	0.13	0.03	0.05	0.09	0.15	0.03	0.03	0.04	0.06	0.04	0.09	0.24	0.47
870.5	374	243043	0.03	0.05	0.10	0.17	0.03	0.06	0.11	0.20	0.03	0.04	0.05	0.07	0.05	0.12	0.34	0.64
871	4405	243043	0.07	0.21	0.64	0.93	0.07	0.24	0.71	0.97	0.04	0.08	0.19	0.36	0.18	0.75	1.00	1.00
872	435	243043	0.03	0.06	0.11	0.19	0.04	0.06	0.12	0.22	0.03	0.04	0.05	0.07	0.05	0.13	0.38	0.70
873	250	243043	0.03	0.05	0.08	0.13	0.03	0.05	0.09	0.15	0.03	0.03	0.04	0.06	0.04	0.09	0.24	0.47
907	1285	243043	0.04	0.09	0.24	0.47	0.05	0.10	0.28	0.54	0.03	0.05	0.08	0.14	0.08	0.30	0.81	0.99
915	4691	243043	0.07	0.22	0.66	0.95	0.07	0.25	0.74	0.97	0.04	0.08	0.20	0.38	0.19	0.77	1.00	1.00
916	1533	243043	0.05	0.10	0.28	0.54	0.05	0.11	0.32	0.61	0.03	0.05	0.09	0.16	0.09	0.34	0.88	1.00

938	368	288850	0.03	0.05	0.10	0.17	0.03	0.06	0.11	0.20	0.03	0.04	0.05	0.07	0.05	0.12	0.33	0.63
938.2	256	288850	0.03	0.05	0.08	0.13	0.03	0.05	0.09	0.15	0.03	0.03	0.04	0.06	0.04	0.09	0.25	0.48
939	2227	288850	0.05	0.13	0.38	0.70	0.05	0.14	0.44	0.77	0.04	0.06	0.12	0.21	0.11	0.47	0.96	1.00
941	259	241796	0.03	0.05	0.08	0.13	0.03	0.05	0.09	0.15	0.03	0.03	0.04	0.06	0.04	0.09	0.25	0.49
946	527	241796	0.04	0.06	0.12	0.23	0.04	0.06	0.14	0.26	0.03	0.04	0.06	0.08	0.06	0.15	0.45	0.78
947	541	288850	0.04	0.06	0.13	0.23	0.04	0.06	0.14	0.27	0.03	0.04	0.06	0.08	0.06	0.15	0.46	0.79
949	397	241796	0.03	0.05	0.10	0.18	0.04	0.06	0.12	0.21	0.03	0.04	0.05	0.07	0.05	0.12	0.36	0.66
958	531	243043	0.04	0.06	0.12	0.23	0.04	0.06	0.14	0.26	0.03	0.04	0.06	0.08	0.06	0.15	0.45	0.79
960	18479	243043	0.15	0.63	1.00	1.00	0.17	0.71	1.00	1.00	0.06	0.19	0.57	0.89	0.55	1.00	1.00	1.00
960.2	16028	243043	0.14	0.57	0.99	1.00	0.15	0.65	1.00	1.00	0.06	0.17	0.51	0.85	0.50	1.00	1.00	1.00
961	411	243043	0.03	0.05	0.11	0.19	0.04	0.06	0.12	0.21	0.03	0.04	0.05	0.07	0.05	0.12	0.37	0.68
961.1	842	243043	0.04	0.07	0.17	0.33	0.04	0.08	0.20	0.38	0.03	0.04	0.07	0.11	0.07	0.21	0.64	0.93
962	482	243043	0.04	0.06	0.12	0.21	0.04	0.06	0.13	0.24	0.03	0.04	0.05	0.08	0.05	0.14	0.42	0.75
962.3	357	243043	0.03	0.05	0.10	0.17	0.03	0.05	0.11	0.19	0.03	0.04	0.05	0.07	0.05	0.11	0.33	0.62
963	2626	243043	0.05	0.14	0.43	0.76	0.06	0.16	0.50	0.83	0.04	0.06	0.13	0.24	0.13	0.53	0.98	1.00
964	391	243043	0.03	0.05	0.10	0.18	0.04	0.06	0.11	0.20	0.03	0.04	0.05	0.07	0.05	0.12	0.35	0.66
964.1	298	243043	0.03	0.05	0.09	0.15	0.03	0.05	0.10	0.17	0.03	0.03	0.05	0.06	0.05	0.10	0.28	0.54
965	6181	243043	0.08	0.27	0.78	0.98	0.09	0.32	0.84	0.99	0.04	0.09	0.24	0.47	0.24	0.87	1.00	1.00
965.1	1370	243043	0.04	0.09	0.25	0.49	0.05	0.11	0.29	0.56	0.03	0.05	0.09	0.14	0.09	0.31	0.84	0.99
965.3	378	243043	0.03	0.05	0.10	0.17	0.04	0.06	0.11	0.20	0.03	0.04	0.05	0.07	0.05	0.12	0.34	0.64
966	507	243043	0.04	0.06	0.12	0.22	0.04	0.06	0.14	0.25	0.03	0.04	0.06	0.08	0.05	0.14	0.43	0.77
967	515	243043	0.04	0.06	0.12	0.22	0.04	0.06	0.14	0.26	0.03	0.04	0.06	0.08	0.06	0.15	0.44	0.77
969	1748	243043	0.05	0.11	0.31	0.59	0.05	0.12	0.36	0.67	0.03	0.05	0.10	0.17	0.10	0.38	0.91	1.00
971	511	243043	0.04	0.06	0.12	0.22	0.04	0.06	0.14	0.25	0.03	0.04	0.06	0.08	0.05	0.14	0.44	0.77
972	828	243043	0.04	0.07	0.17	0.33	0.04	0.08	0.19	0.38	0.03	0.04	0.07	0.10	0.07	0.21	0.63	0.93
973	211	243043	0.03	0.04	0.07	0.12	0.03	0.05	0.08	0.13	0.03	0.03	0.04	0.05	0.04	0.08	0.21	0.41
977	5864	243043	0.08	0.26	0.76	0.98	0.08	0.30	0.83	0.99	0.04	0.09	0.23	0.45	0.23	0.86	1.00	1.00
979	692	243043	0.04	0.07	0.15	0.28	0.04	0.07	0.17	0.33	0.03	0.04	0.06	0.09	0.06	0.18	0.55	0.88
981	262	243043	0.03	0.05	0.08	0.13	0.03	0.05	0.09	0.15	0.03	0.03	0.04	0.06	0.04	0.09	0.25	0.49
990	3850	243043	0.06	0.19	0.58	0.90	0.07	0.22	0.65	0.94	0.04	0.07	0.17	0.32	0.16	0.69	1.00	1.00
994	3970	243043	0.06	0.19	0.59	0.91	0.07	0.22	0.67	0.95	0.04	0.07	0.17	0.33	0.17	0.70	1.00	1.00
994.2	3970	243043	0.06	0.19	0.59	0.91	0.07	0.22	0.67	0.95	0.04	0.07	0.17	0.33	0.17	0.70	1.00	1.00
**Total no of phecodes with power >80%**			0	17	151	319	0	24	178	367	0	0	10	60	10	186	557	801

Supplementary Table 6: Associations of each genetic instrument (PCSK9, HMGCR, NPC1L1, and LDLR) with cardiovascular related biomarkers (from the IVWMR analyses).

	PCSK9 genetic instrument			HMGCR genetic instrument			NPC1L1 genetic instrument			LDLR genetic instrument		
	**beta (95% CI)**	**P**	**F-statistic**	**beta (95% CI)**	**P**	**F-statistic**	**beta (95% CI)**	**P**	**F-statistic**	**beta (95% CI)**	**P**	**F-statistic**
**Cholesterol (mmol/L)**	-0.02 (-0.02, -0.02)	3.3E-62	324.08	-0.03 (-0.03, -0.03)	1.7E-132	421.95	-0.02 (-0.02, -0.02)	4.2E-27	61.88	-0.03 (-0.03, -0.02)	6.1E-38	1649.14
**Direct Low-Density Lipoprotein (mmol/L)**	-0.02 (-0.02, -0.01)	1.8E-61	400.03	-0.02 (-0.02, -0.02)	4.6E-170	481.80	-0.02 (-0.02, -0.01)	2.8E-36	84.42	-0.02 (-0.02, -0.02)	4.4E-58	2077.99
**HDL-Cholesterol (mmol/L)**	0.00 (-0.00, 0.00)	0.21	1.16	-0.00 (-0.00, -0.00)	7.5E-07	8.77	0.00 (0.00, 0.00)	2.3E-03	4.97	0.00 (-0.00, 0.00)	0.57	1.21
**Triglyceride (mmol/L)**	0.00 (-0.00, 0.00)	0.06	2.99	-0.00 (-0.00, 0.00)	0.17	1.50	-0.00 (-0.01, 0.00)	0.05	5.81	-0.00 (-0.00, 0.00)	0.32	0.99
**Apolipoprotein A (g/L)**	0.00 (0.00, 0.00)	1.6E-05	5.70	0.00 (0.00, 0.00)	2.7E-03	2.07	0.00 (0.00, 0.00)	2.1E-04	6.05	0.00 (0.00, 0.00)	7.2E-09	47.04
**Apolipoprotein B (g/L)**	-0.00 (-0.00, -0.00)	9.7E-57	378.48	-0.01 (-0.01, -0.00)	5.3E-154	371.08	-0.00 (-0.00, -0.00)	1.9E-26	61.09	-0.01 (-0.01, -0.01)	6.5E-67	2011.47
**C-reactive Protein (mg/L)**	0.01 (0.00, 0.02)	4.7E-03	6.35	0.00 (-0.00, 0.01)	0.19	0.57	0.01 (-0.02, 0.04)	0.42	2.38	-0.00 (-0.00, 0.00)	0.88	0.18
**Lipoprotein (a) (nmol/L)**	-0.05 (-0.11, 0.02)	0.15	1.24	-0.11 (-0.18, -0.04)	2.5E-03	3.37	-0.00 (-0.17, 0.17)	0.98	0.05	-0.09 (-0.14, -0.04)	1.0E-03	9.54

Supplementary Table 7: Associations between each LDL-C lowering genetic risk score and known confounders.

	PCSK9 GRS		HMGCR GRS		NPC1L1 GRS		LDLR GRS	
	**OR/beta (95% CI)**	**P**	**OR/beta (95% CI)**	**P**	**OR/beta (95% CI)**	**P**	**OR/beta (95% CI)**	**P**
**Age[a]**	-0.002 (-0.013, 0.009)	0.76	-0.015 (-0.025, -0.004)[c]	0.007/ 0.049[b]	-0.005 (-0.025, 0.015)	0.64	-0.027 (-0.050, -0.004)	0.023
**Sex**	-0.002 (-0.005, 0.0004)	0.091	0.0005 (-0.002, 0.003)	0.74	-0.002 (-0.007, 0.003)	0.54	0.0006 (-0.005, 0.007)	0.83
**Smoking**	-0.0009 (-0.004, 0.002)	0.56	0.002 (-0.001, 0.004)	0.31	-0.0004 (-0.006, 0.005)	0.88	0.004 (-0.002, 0.010)	0.20
**Alcohol**	0.003 (-0.003, 0.008)	0.34	0.002 (-0.003, 0.008)	0.38	0.003 (-0.007, 0.014)	0.51	0.0002 (-0.012, 0.012)	0.98
**Physical activity**	0.001 (-0.001, 0.004)	0.34	-0.001 (-0.004, 0.002)	0.51	-0.001 (-0.006, 0.005)	0.81	-0.003 (-0.009, 0.003)	0.27
**Education**	0.0001 (-0.003, 0.003)	0.97	-0.002 (-0.005, 0.0003)	0.08	-0.002 (-0.007, 0.003)	0.45	-0.004 (-0.010, 0.002)	0.17
**Townsend deprivation index**	0.004 (0.0008, 0.006)	0.01 / 0.07[b]	-0.0003 (-0.003, 0.002)	0.82	0.003 (-0.002, 0.008)	0.29	0.003 (-0.003, 0.009)	0.40

[a] Effect estimate for models with age is from linear regression· All other estimates from logistic regression where variables were grouped as: Smoking (never vs. ex-or current smoker). Alcohol consumption (never vs. ex- or current drinker), physical activity (below vs. above the median MET/week), education (College or University degree vs. other levels including none), and Townsend deprivation index (less deprived vs more deprived; using a median). All adjusted for assessment center, types of SNP array, 15 principal components.

[b] Bonferroni corrected. Bonferroni threshold = 0.007.

[c] Odds per one allele higher, adjusted for LDL-C = -0.009 (-0.020, 0.002), P = 0.1

Supplementary Table 8: MR analyses using MR IVW, weighted medium, weighted mode, MR-Egger and MR-PRESSO approaches, for 13 disease outcomes identified in the PheWAS of LDL-C lowering variants under FDR corrected thresholds.

						MR IVW		Weighted median		Weighted mode		MR-Egger		MR-PRESSO			
**GRS**	**Phecode**	**Description**	**Cases, n**	**Controls, n**	**P_PheWAS**	**OR (95% CI)**	**P**	**OR (95% CI)**	**P**	**OR (95% CI)**	**P**	**OR (95% CI)**	**P**	**OR (95% CI)**	**P**	$P_{pleiotropy}$	$P_{distortion}$
PCSK9	272	Disorders of lipoid metabolism	35143	261466	1.0E-11	0.98 (0.98, 0.98)	4.3E-25	0.98 (0.97, 0.98)	1.4E-16	0.98 (0.97, 0.99)	1.2E-03	0.99 (0.95, 1.02)	1.5E-01	0.98 (0.98, 0.98)	4.2E-06	0.38	
	272.1	Hyperlipidemia	35033	261466	6.6E-12	0.98 (0.98, 0.98)	1.7E-25	0.98 (0.97, 0.98)	1.6E-17	0.98 (0.97, 0.99)	1.0E-03	0.99 (0.95, 1.02)	1.4E-01	0.98 (0.98, 0.98)	4.2E-06	0.38	
	272.11	Hypercholesterolemia	32554	261466	4.2E-11	0.98 (0.98, 0.98)	3.3E-24	0.98 (0.98, 0.99)	3.2E-16	0.98 (0.97, 0.99)	8.2E-04	0.99 (0.96, 1.02)	2.2E-01	0.98 (0.97, 0.98)	1.4E-05	0.27	
	401.2	Hypertensive heart and/or renal disease	1667	202269	1.6E-04	0.96 (0.94, 0.97)	9.7E-08	0.96 (0.94, 0.98)	8.8E-05	0.97 (0.92, 1.02)	7.2E-02	0.95 (0.82, 1.09)	1.7E-01	0.96 (0.93, 0.98)	1.1E-03	0.80	
	411	Ischemic Heart Disease	30489	202269	3.3E-06	0.99 (0.98, 0.99)	9.7E-10	0.99 (0.98, 0.99)	1.1E-07	0.98 (0.97, 1.00)	8.5E-03	0.99 (0.96, 1.03)	4.5E-01	0.99 (0.98, 0.99)	8.4E-04	0.50	
	411.1	Unstable angina (intermediate coronary syndrome)	4478	202269	7.5E-01	1.00 (0.99, 1.01)	9.8E-01	1.00 (0.99, 1.02)	4.6E-01	1.01 (0.98, 1.04)	5.1E-01	1.02 (0.93, 1.12)	4.4E-01	1.00 (0.98, 1.02)	9.9E-01	0.42	
	411.2	Myocardial infarction	12033	202269	1.3E-02	0.99 (0.98, 1.00)	6.7E-03	0.99 (0.98, 1.00)	1.3E-02	0.99 (0.97, 1.01)	1.5E-01	1.01 (0.96, 1.07)	3.9E-01	0.99 (0.98, 1.00)	3.5E-02	0.13	
	411.3	Angina pectoris	15180	202269	8.6E-06	0.98 (0.98, 0.99)	5.6E-10	0.98 (0.97, 0.99)	9.2E-08	0.98 (0.97, 0.99)	4.7E-03	0.99 (0.94, 1.04)	2.8E-01	0.98 (0.98, 0.99)	6.1E-05	0.71	
	411.4	Coronary atherosclerosis	19684	202269	9.4E-04	0.99 (0.98, 0.99)	1.6E-05	0.99 (0.98, 0.99)	1.7E-04	0.99 (0.97, 1.00)	3.2E-02	1.00 (0.95, 1.04)	7.6E-01	0.99 (0.98, 1.00)	2.8E-03	0.45	
	411.8	Other chronic ischemic heart disease, unspecified	15139	202269	2.0E-04	0.99 (0.98, 0.99)	8.9E-07	0.99 (0.98, 0.99)	1.2E-04	0.98 (0.96, 1.00)	1.0E-02	1.00 (0.95, 1.05)	7.0E-01	0.99 (0.98, 0.99)	2.9E-04	0.42	
	442.11	Abdominal aortic aneurysm	1047	202269	2.4E-01	0.98 (0.96, 1.01)	2.9E-01	0.99 (0.96, 1.02)	4.6E-01	1.00 (0.92, 1.08)	8.8E-01	0.94 (0.73, 1.21)	3.3E-01	0.98 (0.94, 1.03)	3.3E-01	0.43	
	443	Peripheral vascular disease	4031	202269	1.6E-01	0.99 (0.98, 1.00)	8.7E-02	0.99 (0.98, 1.00)	1.2E-01	0.99 (0.96, 1.02)	2.2E-01	0.98 (0.90, 1.08)	4.6E-01	0.99 (0.98, 1.00)	3.1E-02	0.72	
	561.1	Diarrhea	536	191014	6.8E-01	1.01 (0.98, 1.04)	6.8E-01	0.99 (0.96, 1.03)	7.9E-01	1.00 (0.93, 1.06)	8.6E-01	0.93 (0.72, 1.18)	2.3E-01	1.01 (0.97, 1.04)	6.2E-01	0.19	
HMGCR	272	Disorders of lipoid metabolism	35143	261466	2.2E-08	0.98 (0.97, 0.98)	3.1E-26	0.98 (0.97, 0.98)	3.3E-17	0.98 (0.97, 0.99)	9.4E-04	0.97 (0.92, 1.02)	6.5E-02	0.98 (0.97, 0.98)	3.5E-06	0.51	
	272.1	Hyperlipidemia	35033	261466	2.2E-08	0.98 (0.97, 0.98)	2.2E-26	0.98 (0.97, 0.98)	1.5E-16	0.98 (0.97, 0.99)	8.2E-04	0.97 (0.92, 1.02)	6.4E-02	0.98 (0.97, 0.98)	3.4E-06	0.50	
	272.11	Hypercholesterolemia	32554	261466	1.5E-08	0.98 (0.97, 0.98)	9.0E-26	0.98 (0.97, 0.98)	8.9E-16	0.98 (0.96, 0.99)	1.2E-03	0.97 (0.91, 1.03)	7.1E-02	0.98 (0.97, 0.98)	1.0E-05	0.55	
	401.2	Hypertensive heart and/or renal disease	1667	202269	4.9E-01	0.98 (0.96, 1.00)	6.7E-02	0.98 (0.96, 1.00)	1.1E-01	0.98 (0.94, 1.03)	2.3E-01	0.94 (0.73, 1.21)	3.4E-01	0.98 (0.95, 1.01)	1.3E-01	0.47	
	411	Ischemic Heart Disease	30489	202269	4.9E-02	0.99 (0.98, 1.00)	6.7E-03	0.99 (0.99, 1.00)	1.8E-02	0.99 (0.98, 1.00)	6.3E-02	0.97 (0.88, 1.06)	2.2E-01	0.99 (0.98, 1.00)	4.2E-02	0.38	
	411.1	Unstable angina (intermediate coronary syndrome)	4478	202269	5.9E-01	0.99 (0.98, 1.00)	2.5E-01	0.99 (0.98, 1.01)	2.8E-01	0.99 (0.96, 1.02)	3.3E-01	0.98 (0.85, 1.14)	6.6E-01	0.99 (0.98, 1.01)	2.5E-01	0.79	
	411.2	Myocardial infarction	12033	202269	3.0E-01	0.99 (0.98, 1.00)	1.0E-01	0.99 (0.98, 1.00)	1.9E-01	0.99 (0.98, 1.01)	3.5E-01	0.97 (0.86, 1.08)	2.4E-01	0.99 (0.98, 1.01)	1.7E-01	0.33	
	411.3	Angina pectoris	15180	202269	5.0E-03	0.98 (0.97, 0.99)	3.4E-08	0.98 (0.97, 0.99)	4.8E-06	0.98 (0.97, 1.00)	1.3E-02	0.97 (0.88, 1.05)	1.6E-01	0.98 (0.97, 0.99)	2.7E-03	0.46	
	411.4	Coronary atherosclerosis	19684	202269	1.5E-02	0.99 (0.98, 0.99)	1.9E-05	0.99 (0.98, 1.00)	1.6E-03	0.99 (0.97, 1.00)	4.2E-02	0.96 (0.89, 1.04)	1.0E-01	0.99 (0.98, 1.00)	7.9E-03	0.26	
	411.8	Other chronic ischemic heart disease, unspecified	15139	202269	1.5E-01	0.99 (0.98, 1.00)	4.9E-03	0.99 (0.98, 1.00)	4.3E-02	0.99 (0.98, 1.01)	1.3E-01	0.98 (0.89, 1.08)	4.2E-01	0.99 (0.98, 1.00)	3.7E-02	0.67	
	442.11	Abdominal aortic aneurysm	1047	202269	8.2E-02	0.96 (0.94, 0.99)	1.3E-03	0.96 (0.94, 0.99)	4.0E-03	0.96 (0.91, 1.02)	8.0E-02	0.98 (0.73, 1.31)	7.9E-01	0.96 (0.94, 0.99)	6.4E-03	0.80	
	443	Peripheral vascular disease	4031	202269	6.5E-01	0.99 (0.98, 1.01)	3.0E-01	1.00 (0.98, 1.01)	6.9E-01	1.00 (0.97, 1.02)	6.3E-01	0.95 (0.80, 1.14)	3.1E-01	0.99 (0.97, 1.02)	3.4E-01	0.38	
	561.1	Diarrhea	536	191014	1.1E-01	0.96 (0.93, 0.99)	5.1E-03	0.97 (0.93, 1.01)	1.7E-01	0.97 (0.90, 1.05)	2.9E-01	1.06 (0.71, 1.58)	5.6E-01	0.96 (0.91, 1.00)	2.5E-02	0.32	
NPC1L1	272	Disorders of lipoid metabolism	35143	261466	3.4E-03	0.98 (0.97, 0.99)	1.4E-03	0.98 (0.97, 1.00)	7.5E-03	0.98 (0.96, 1.01)	1.1E-01	1.03 (0.84, 1.27)	5.7E-01	0.98 (0.97, 1.00)	4.5E-02	0.41	
	272.1	Hyperlipidemia	35033	261466	3.4E-03	0.98 (0.97, 0.99)	1.3E-03	0.98 (0.97, 1.00)	6.3E-03	0.98 (0.96, 1.01)	9.5E-02	1.03 (0.84, 1.27)	6.0E-01	0.98 (0.97, 1.00)	3.9E-02	0.43	
	272.11	Hypercholesterolemia	32554	261466	2.1E-03	0.98 (0.97, 0.99)	7.4E-04	0.98 (0.97, 1.00)	7.7E-03	0.98 (0.96, 1.01)	9.2E-02	1.02 (0.83, 1.26)	7.1E-01	0.98 (0.97, 1.00)	2.4E-02	0.50	
	401.2	Hypertensive heart and/or renal disease	1667	202269	8.1E-02	0.95 (0.91, 0.99)	1.1E-02	0.94 (0.89, 0.99)	1.5E-02	0.98 (0.89, 1.07)	4.9E-01	0.96 (0.39, 2.33)	8.6E-01	0.95 (0.89, 1.00)	6.1E-02	0.96	
	411	Ischemic Heart Disease	30489	202269	3.8E-02	0.98 (0.96, 1.00)	1.0E-01	0.97 (0.96, 0.99)	3.6E-03	0.97 (0.94, 1.00)	5.3E-02	1.06 (0.69, 1.62)	6.3E-01	0.98 (0.95, 1.02)	2.0E-01	0.54	
	411.1	Unstable angina (intermediate coronary syndrome)	4478	202269	3.9E-01	0.98 (0.93, 1.03)	4.9E-01	0.95 (0.92, 0.99)	1.8E-02	0.95 (0.90, 1.01)	7.6E-02	1.20 (0.40, 3.59)	5.5E-01	0.98 (0.90, 1.07)	5.4E-01	0.51	
	411.2	Myocardial infarction	12033	202269	9.8E-02	0.98 (0.96, 1.01)	1.4E-01	0.99 (0.97, 1.02)	4.2E-01	0.96 (0.92, 1.00)	6.3E-02	1.00 (0.57, 1.78)	9.9E-01	0.98 (0.94, 1.02)	2.4E-01	0.89	
	411.3	Angina pectoris	15180	202269	1.2E-01	0.98 (0.97, 1.00)	8.0E-02	0.97 (0.96, 0.99)	1.1E-02	0.97 (0.94, 1.01)	9.4E-02	1.07 (0.74, 1.54)	5.4E-01	0.98 (0.95, 1.01)	1.8E-01	0.45	
	411.4	Coronary atherosclerosis	19684	202269	2.1E-02	0.98 (0.96, 1.00)	5.8E-02	0.97 (0.95, 0.99)	1.8E-03	0.96 (0.93, 1.00)	4.4E-02	1.06 (0.65, 1.74)	6.6E-01	0.98 (0.94, 1.02)	1.5E-01	0.55	
	411.8	Other chronic ischemic heart disease, unspecified	15139	202269	4.7E-02	0.98 (0.96, 1.00)	4.2E-02	0.98 (0.96, 1.00)	3.8E-02	0.97 (0.93, 1.01)	8.3E-02	1.09 (0.75, 1.58)	4.1E-01	0.98 (0.95, 1.01)	1.3E-01	0.32	
	442.11	Abdominal aortic aneurysm	1047	202269	1.4E-01	0.95 (0.90, 1.01)	1.0E-01	0.95 (0.89, 1.02)	1.6E-01	0.95 (0.84, 1.07)	2.6E-01	1.00 (0.34, 2.95)	1.0E+00	0.95 (0.94, 0.97)	4.9E-03	0.87	
	443	Peripheral vascular disease	4031	202269	5.5E-01	1.01 (0.98, 1.04)	4.6E-01	1.02 (0.98, 1.05)	3.8E-01	1.02 (0.96, 1.08)	3.8E-01	1.13 (0.64, 1.98)	4.6E-01	1.01 (0.98, 1.04)	3.2E-01	0.49	
	561.1	Diarrhea	536	191014	1.0E-04	1.23 (1.14, 1.33)	4.0E-07	1.23 (1.12, 1.35)	2.0E-05	1.23 (1.03, 1.47)	3.4E-02	1.62 (0.31, 8.35)	3.3E-01	1.23 (1.14, 1.33)	3.6E-03	0.54	
LDLR	272	Disorders of lipoid metabolism	35143	261466	1.7E-51	0.97 (0.97, 0.97)	4.2E-67	0.97 (0.97, 0.97)	9.5E-50	0.97 (0.96, 0.98)	5.8E-03	0.97 (0.96, 0.99)	7.8E-02			0.99	
	272.1	Hyperlipidemia	35033	261466	2.1E-51	0.97 (0.97, 0.97)	5.6E-67	0.97 (0.97, 0.97)	1.3E-52	0.97 (0.96, 0.98)	4.7E-03	0.97 (0.96, 0.99)	7.6E-02			0.99	
	272.11	Hypercholesterolemia	32554	261466	2.8E-53	0.97 (0.97, 0.97)	1.7E-69	0.97 (0.97, 0.97)	8.4E-55	0.97 (0.96, 0.98)	5.2E-03	0.97 (0.95, 0.99)	8.3E-02			0.96	
	401.2	Hypertensive heart and/or renal disease	1667	202269	2.8E-01	0.99 (0.98, 1.00)	2.0E-01	0.99 (0.98, 1.00)	1.8E-01	0.99 (0.97, 1.02)	3.3E-01	0.99 (0.94, 1.04)	5.1E-01			0.81	
	411	Ischemic Heart Disease	30489	202269	1.0E-12	0.98 (0.98, 0.99)	3.6E-14	0.99 (0.98, 0.99)	6.4E-13	0.99 (0.98, 0.99)	2.1E-02	0.99 (0.97, 1.01)	2.1E-01			0.85	
	411.1	Unstable angina (intermediate coronary syndrome)	4478	202269	9.3E-05	0.98 (0.97, 0.99)	3.8E-03	0.98 (0.97, 0.99)	3.4E-05	0.98 (0.97, 1.00)	8.2E-02	0.99 (0.96, 1.03)	5.9E-01			0.27	
	411.2	Myocardial infarction	12033	202269	7.2E-10	0.98 (0.97, 0.99)	1.3E-12	0.98 (0.98, 0.99)	4.0E-09	0.98 (0.97, 0.99)	3.2E-02	0.98 (0.96, 1.01)	2.2E-01			0.71	
	411.3	Angina pectoris	15180	202269	9.1E-08	0.98 (0.98, 0.99)	7.2E-10	0.98 (0.98, 0.99)	6.3E-09	0.99 (0.98, 0.99)	3.6E-02	0.99 (0.97, 1.01)	2.0E-01			0.73	
	411.4	Coronary atherosclerosis	19684	202269	1.2E-15	0.98 (0.97, 0.98)	3.7E-20	0.98 (0.98, 0.98)	2.2E-17	0.98 (0.97, 0.99)	1.6E-02	0.98 (0.96, 1.00)	1.3E-01			0.66	
	411.8	Other chronic ischemic heart disease, unspecified	15139	202269	8.5E-10	0.98 (0.98, 0.99)	2.2E-12	0.98 (0.98, 0.99)	1.2E-10	0.98 (0.97, 0.99)	2.9E-02	0.98 (0.96, 1.00)	1.7E-01			0.85	
	442.11	Abdominal aortic aneurysm	1047	202269	6.1E-05	0.96 (0.94, 0.98)	4.4E-06	0.96 (0.94, 0.97)	4.2E-06	0.95 (0.92, 0.99)	5.4E-02	0.95 (0.88, 1.03)	2.4E-01			0.78	
	443	Peripheral vascular disease	4031	202269	7.6E-05	0.98 (0.97, 0.99)	5.5E-04	0.98 (0.97, 0.99)	4.3E-05	0.98 (0.97, 1.00)	8.9E-02	0.99 (0.96, 1.03)	4.9E-01			0.32	
	561.1	Diarrhea	536	191014	3.9E-01	0.99 (0.96, 1.01)	3.2E-01	0.98 (0.96, 1.01)	1.9E-01	0.98 (0.94, 1.03)	3.4E-01	0.97 (0.88, 1.07)	3.9E-01			0.49	

* MR-PRESSO estimates could not be calculated for the LDLR genetic risk score because there are only 3 genes.

Supplementary Table 9: Replication of MR analyses for disease phenotypes available in the MR-Base repository.

						MR IVW		Weighted median	Weighted mode	MR-Egger	MR-PRESSO			
**GRS**	**Disease phenotype**	**Consortium**	**Harmonized SNP, n**	**Cases, n**	**Controls, n**	**OR (95% CI)**	**P**	**OR (95% CI)**	**OR (95% CI)**	**OR (95% CI)**	**Outliers**	**OR (95% CI) $_{outliers\ removed}$**	**$P_{pleiotropy}$**	**$P_{distortion}$**
PCSK9	Pure hypercholesterolaemia	FinnGen*	4	3262	91366	0.97 (0.95, 1.00)	0.03	0.96 (0.94, 0.99)	0.96 (0.91, 1.02)	1.02 (0.78, 1.35)	None	0.97 (0.94, 1.01)	0.11	NA
	Hyperlipidaemia, other/unspecified	FinnGen	4	1539	91366	0.98 (0.95, 1.02)	0.42	0.98 (0.94, 1.01)	0.96 (0.88, 1.05)	1.12 (0.80, 1.59)	None	0.98 (0.92, 1.05)	0.13	NA
	Angina pectoris	FinnGen	4	6382	85760	0.99 (0.97, 1.01)	0.33	0.99 (0.98, 1.01)	1.00 (0.96, 1.04)	1.07 (0.90, 1.27)	None	0.99 (0.96, 1.02)	0.07	NA
	Hypertensive heart and/or renal disease	FinnGen	4	1798	74345	0.98 (0.95, 1.01)	0.22	0.97 (0.94, 1.00)	0.96 (0.89, 1.05)	1.02 (0.69, 1.53)	None	0.98 (0.93, 1.03)	0.16	NA
	Ischemic heart diseases	FinnGen	4	10739	85760	0.98 (0.97, 1.00)	0.01	0.99 (0.97, 1.00)	0.99 (0.96, 1.03)	1.04 (0.90, 1.20)	None	0.98 (0.96, 1.01)	0.06	NA
HMGCR	Pure hypercholesterolaemia	FinnGen	5	3262	91366	0.96 (0.95, 0.98)	3.5E-05	0.97 (0.95, 0.98)	0.97 (0.93, 1.00)	0.97 (0.71, 1.33)	None	0.96 (0.96, 0.97)	0.92	NA
	Hyperlipidaemia, other/unspecified	FinnGen	5	1539	91366	0.97 (0.95, 1.00)	0.04	0.97 (0.95, 1.00)	0.98 (0.93, 1.03)	1.04 (0.66, 1.62)	None	0.97 (0.95, 1.00)	0.59	NA
LDLR	Pure hypercholesterolaemia	FinnGen	3	3262	91366	0.96 (0.94, 0.98)	1.7E-04	0.97 (0.95, 0.98)	0.97 (0.95, 0.99)	0.98 (0.94, 1.02)	Insufficient no. of SNPs	NA	0.24	NA
	Hyperlipidaemia, other/unspecified	FinnGen	3	1539	91366	0.97 (0.96, 0.99)	3.6E-04	0.97 (0.95, 0.99)	0.97 (0.94, 1.00)	0.96 (0.90, 1.02)	Insufficient no. of SNPs	NA	0.49	NA
	Angina pectoris	FinnGen	3	6382	85760	0.99 (0.98, 1.00)	0.01	0.99 (0.98, 1.00)	0.99 (0.98, 1.01)	0.99 (0.96, 1.03)	Insufficient no. of SNPs	NA	0.75	NA
	Aortic aneurysm	FinnGen	3	791	92349	1.00 (0.98, 1.02)	0.79	1.00 (0.98, 1.03)	1.01 (0.97, 1.05)	1.02 (0.94, 1.11)	Insufficient no. of SNPs	NA	0.49	NA
	Coronary atherosclerosis	FinnGen	3	7661	85760	0.99 (0.98, 1.00)	0.003	0.99 (0.98, 0.99)	0.99 (0.98, 1.00)	1.00 (0.97, 1.03)	Insufficient no. of SNPs	NA	0.34	NA
	Hypertensive heart and/or renal disease	FinnGen	3	1798	74345	1.00 (0.98, 1.01)	0.51	0.99 (0.98, 1.01)	0.99 (0.96, 1.02)	0.99 (0.93, 1.05)	Insufficient no. of SNPs	NA	0.59	NA
	Ischemic heart diseases	FinnGen	3	10739	85760	0.99 (0.98, 1.00)	0.02	0.99 (0.98, 1.00)	0.99 (0.98, 1.01)	1.00 (0.97, 1.02)	Insufficient no. of SNPs	NA	0.55	NA
	Myocardial infarction	FinnGen	3	4065	85760	0.98 (0.97, 1.00)	0.06	0.99 (0.97, 1.00)	0.99 (0.97, 1.01)	1.00 (0.96, 1.04)	Insufficient no. of SNPs	NA	0.27	NA
	Unstable angina pectoris	FinnGen	3	2296	89324	0.98 (0.97, 0.99)	0.002	0.98 (0.97, 0.99)	0.98 (0.96, 1.00)	0.98 (0.93, 1.03)	Insufficient no. of SNPs	NA	0.83	NA

NA: not applicable

* The FinnGen study contains genome information (from Finnish biobanks) and linked digital healthcare data (from national health registries), collected from >300,000 participants in Finland.

Supplementary Table 10: List of 52 serum, cardiac imaging, brain imaging and clinical biomarkers.

	Biomarker	Unit	Sample type	Method of Data Collection or Analysis
**Serum biomarkers**	Cholesterol	mmol/L	Serum	CHO-POD
	Direct Low-Density Lipoprotein	mmol/L	Serum	Enzymatic selective protection
	HDL-Cholesterol	mmol/L	Serum	Enzyme immuno-inhibition
	Triglyceride	mmol/L	Serum	GPO-POD
	Apolipoprotein A	g/L	Serum	Immunoturbidimetric
	Apolipoprotein B	g/L	Serum	Immunoturbidimetric
	C-reactive Protein	mg/L	Serum	Immunoturbidimetric - high sensitivity
	Lipoprotein (a)	nmol/L	Serum	Immunoturbidimetric
	Vitamin D	nmol/L	Serum	CLIA
	Rheumatoid factor	IU/mL	Serum	Immunoturbidimetric
	Alkaline Phosphatase	U/L	Serum	AMP (IFCC)
	Calcium	μmol/L	Serum	Arsenazo III
	SHBG	nmol/L	Serum	Two-step sandwich immunoassay
	Testosterone	pmol/L	Serum	One step competitive
	Oestradiol	pmol/L	Serum	Two-step competitive
	IGF-1	pmol/L	Serum	CLIA
	Glycated Haemoglobin (HbA1c)	mmol/mol	Red blood cells (RBC)	HPLC
	Glucose	mg/dL	Serum	Hexokinase
	Cystatin C	mg/L	Serum	Latex enhanced immunoturbidimetric
	Creatinine	μmol/L	Serum	Enzymatic
	Total protein	g/L	Serum	Biuret
	Urea	mmol/L	Serum	GLDH, kinetic
	Phosphate	mmol/L	Serum	Phophomolybdate complex
	Urate	μmol/L	Serum	Uricase PAP
	Creatinine (urine)	mmol/L	Urine	Enzymatic
	Sodium (urine)	mmol/L	Urine	ISE
	Potassium (urine)	mmol/L	Urine	ISE
	Albumin	g/L	Serum	BCG
	Direct Bilirubin	nmol/L	Serum	DPD
	Total Bilirubin	μmol/L	Serum	Photometric colour
	Gamma Glutamyltransferase	U/L	Serum	IFCC
	Alanine aminotransferase	U/L	Serum	IFCC
	Aspartate aminotransferase	U/L	Serum	IFCC
**Cardiac imaging biomarkers**	Augmentation index for PWA	%	Cardiac imaging	Pulse wave analysis
	Central augmentation pressure during PWA	mmHg	Cardiac imaging	Pulse wave analysis
	Mean carotid IMT (intima-medial thickness) at 240 degrees	micrometres	Cardiac imaging	Carotid ultrasound
	Cardiac output	L/min	Cardiac imaging	Left ventricular size and function derived from Heart MRI data
	LV end systolic volume	mL	Cardiac imaging	Left ventricular size and function derived from Heart MRI data
	LV end diastolic volume	mL	Cardiac imaging	Left ventricular size and function derived from Heart MRI data
	LV ejection fraction	%	Cardiac imaging	Left ventricular size and function derived from Heart MRI data
**Brain imaging biomarkers**	Total brain volume	cm^3	Brain imaging	T1 structural brain MRI
	Grey matter volume	cm^3	Brain imaging	T1 structural brain MRI
	White matter volume	cm^3	Brain imaging	T1 structural brain MRI
	Hippocampal volume	mm^3	Brain imaging	T1 structural brain MRI
	White matter hyperintensity volume	mm^3	Brain imaging	T1 + T2_FLAIR brain MRI
**Clinical**	Body mass index (BMI)	kg/m^2	Anthropometric	Baseline impedance measures
	Body fat percentage (%)	%	Anthropometric	Baseline impedance measures
	Forced expiratory volume (FEV1)	mL	Spirometry	Vitalograph Pneumotrac 6800 breath spirometry
	Forced vital capacity (FVC)	mL	Spirometry	Vitalograph Pneumotrac 6800 breath spirometry
	FEV1/FVC ratio		Spirometry	Vitalograph Pneumotrac 6800 breath spirometry
	Systolic blood pressure	mmHg	Clinical	Automated reading on an Omron device
	Diastolic blood pressure	mmHg	Clinical	Automated reading on an Omron device

Supplementary Table 11: MR analyses using MR IVW, weighted medium, weighted mode, MR-Egger and MR-PRESSO approaches, for all 52 serum, urine, cardiac imaging, brain imaging and clinical biomarkers, using the PCSK9 genetic instrument.

			MR IVW		Weighted median		Weighted mode		MR-Egger		MR-PRESSO			
GRS	Biomarker	Sample size, n	beta (95% CI)	P	beta (95% CI)	P	beta (95% CI)	P	beta (95% CI)	P	beta (95% CI)	P	$P_{pleiotropy}$	$P_{distortion}$
PCSK9	Cholesterol	321,748	-0.02 (-0.02, -0.02)	3.3E-62	-0.02 (-0.02, -0.02)	7.3E-81	-0.02 (-0.02, -0.01)	7.2E-06	-0.01 (-0.03, 0.00)	0.02	-0.02 (-0.02, -0.01)	3.0E-06	0.24	NA
	Direct Low-Density Lipoprotein	321,141	-0.02 (-0.02, -0.01)	1.8E-61	-0.02 (-0.02, -0.01)	8.7E-101	-0.02 (-0.02, -0.01)	3.3E-06	-0.01 (-0.03, 0.00)	0.02	-0.02 (-0.02, -0.01)	3.1E-06	0.40	0.17
	HDL-Cholesterol	294,531	0.00 (-0.00, 0.00)	0.21	0.00 (-0.00, 0.00)	0.21	0.00 (-0.00, 0.00)	0.28	0.00 (-0.00, 0.00)	0.90	0.00 (-0.00, 0.00)	0.26	0.68	NA
	Triglyceride	321,484	0.00 (-0.00, 0.00)	0.06	0.00 (-0.00, 0.00)	0.19	0.00 (-0.00, 0.00)	0.48	0.00 (-0.01, 0.01)	0.98	0.00 (-0.00, 0.00)	0.11	0.64	NA
	Apolipoprotein A	292,911	0.00 (0.00, 0.00)	1.6E-05	0.00 (0.00, 0.00)	2.4E-03	0.00 (-0.00, 0.00)	0.11	0.00 (-0.00, 0.00)	0.25	0.00 (0.00, 0.00)	4.5E-03	0.81	NA
	Apolipoprotein B	320,189	-0.00 (-0.00, -0.00)	9.7E-57	-0.00 (-0.00, -0.00)	6.1E-102	-0.00 (-0.00, -0.00)	2.9E-06	-0.00 (-0.01, 0.00)	0.02	-0.00 (-0.00, -0.00)	4.0E-06	0.47	0.12
	C-reactive Protein	321,051	0.01 (0.00, 0.02)	4.7E-03	0.01 (0.00, 0.02)	0.01	0.01 (-0.01, 0.02)	0.22	0.02 (-0.04, 0.08)	0.28	0.01 (-0.00, 0.02)	0.03	0.61	NA
	Lipoprotein (a)	255,820	-0.05 (-0.11, 0.02)	0.15	-0.03 (-0.11, 0.05)	0.44	-0.01 (-0.19, 0.16)	0.81	0.05 (-0.50, 0.60)	0.73	-0.05 (-0.11, 0.01)	0.04	0.48	NA
	Vitamin D	307,609	0.04 (0.01, 0.06)	2.0E-03	0.03 (0.00, 0.06)	0.04	0.03 (-0.05, 0.11)	0.25	0.07 (-0.14, 0.28)	0.20	0.04 (0.00, 0.07)	0.01	0.50	NA
	Rheumatoid factor	28,705	0.06 (-0.01, 0.14)	0.09	0.07 (-0.02, 0.16)	0.12	0.07 (-0.12, 0.26)	0.27	0.15 (-0.50, 0.80)	0.37	0.06 (-0.00, 0.13)	0.02	0.59	NA
	Alkaline Phosphatase	321,762	0.03 (-0.00, 0.06)	0.08	0.02 (-0.02, 0.06)	0.28	0.02 (-0.08, 0.12)	0.52	0.07 (-0.19, 0.33)	0.32	0.03 (-0.02, 0.07)	0.12	0.52	NA
	Calcium	294,549	-0.00 (-0.00, -0.00)	0.04	-0.00 (-0.00, 0.00)	0.10	-0.00 (-0.00, 0.00)	0.28	-0.00 (-0.00, 0.00)	0.67	-0.00 (-0.00, 0.00)	0.08	0.96	NA
	SHBG	291,824	0.03 (0.00, 0.07)	0.03	0.04 (-0.00, 0.08)	0.06	0.04 (-0.05, 0.12)	0.23	0.04 (-0.23, 0.30)	0.59	0.03 (0.00, 0.07)	0.02	0.99	NA
	Testosterone	291,707	0.00 (-0.00, 0.01)	0.23	0.00 (-0.00, 0.01)	0.62	0.00 (-0.01, 0.01)	0.90	0.01 (-0.02, 0.04)	0.16	0.00 (-0.00, 0.01)	0.28	0.23	NA
	Oestradiol	50,478	0.86 (-0.34, 2.07)	0.16	1.26 (-0.32, 2.84)	0.12	1.37 (-2.46, 5.21)	0.30	-2.74 (-13.05, 7.57)	0.30	0.86 (-1.08, 2.80)	0.21	0.18	NA
	IGF-1	320,022	-0.01 (-0.02, -0.00)	6.0E-03	-0.01 (-0.01, 0.00)	0.14	-0.01 (-0.02, 0.01)	0.32	-0.01 (-0.07, 0.04)	0.42	-0.01 (-0.02, 0.00)	0.02	0.87	NA
	Glycated Haemoglobin (HbA1c)	321,610	-0.00 (-0.01, 0.00)	0.46	-0.00 (-0.01, 0.01)	0.38	-0.01 (-0.02, 0.01)	0.36	-0.02 (-0.08, 0.04)	0.21	-0.00 (-0.01, 0.01)	0.44	0.26	NA
	Glucose	294,346	-0.00 (-0.01, 0.00)	0.46	-0.00 (-0.01, 0.01)	0.38	-0.01 (-0.02, 0.01)	0.36	-0.02 (-0.08, 0.04)	0.21	-0.00 (-0.01, 0.01)	0.44	0.10	NA
	Cystatin C	321,727	-0.00 (-0.00, 0.00)	0.05	-0.00 (-0.00, 0.00)	0.13	-0.00 (-0.00, 0.00)	0.35	0.00 (-0.00, 0.00)	0.68	-0.00 (-0.00, 0.00)	0.06	0.38	NA
	Creatinine	321,583	-0.00 (-0.00, 0.00)	0.28	-0.00 (-0.00, 0.00)	0.79	0.00 (-0.00, 0.00)	0.93	0.00 (-0.00, 0.00)	1.00	-0.00 (-0.00, 0.00)	0.32	0.80	NA
	Total protein	294,331	-0.01 (-0.02, -0.00)	0.02	-0.01 (-0.01, -0.00)	8.0E-03	-0.01 (-0.02, 0.00)	0.06	-0.02 (-0.09, 0.05)	0.25	-0.01 (-0.02, 0.00)	0.06	0.48	NA
	Urea	321,537	-0.00 (-0.00, 0.00)	0.08	-0.00 (-0.00, 0.00)	0.06	-0.00 (-0.01, 0.00)	0.35	0.00 (-0.02, 0.02)	0.82	-0.00 (-0.01, 0.00)	0.14	0.53	NA
	Phosphate	294,096	-0.00 (-0.00, 0.00)	0.65	-0.00 (-0.00, 0.00)	0.50	-0.00 (-0.00, 0.00)	0.65	0.00 (-0.00, 0.00)	0.18	-0.00 (-0.00, 0.00)	0.62	0.15	NA
	Urate	321,334	0.13 (0.05, 0.21)	1.3E-03	0.13 (0.04, 0.23)	7.8E-03	0.18 (-0.04, 0.40)	0.04	0.20 (-0.47, 0.88)	0.25	0.13 (0.05, 0.21)	2.7E-03	0.65	NA
	Creatinine (urine)	327,791	-7.95 (-14.13, -1.77)	0.01	-10.01 (-17.81, -2.21)	0.01	-12.01 (-28.66, 4.63)	0.06	-11.88 (-64.86, 41.11)	0.38	-7.95 (-16.63, 0.73)	0.03	0.75	NA
	Sodium (urine)	327,096	-0.02 (-0.07, 0.03)	0.44	-0.04 (-0.09, 0.02)	0.23	-0.04 (-0.16, 0.09)	0.36	-0.03 (-0.44, 0.38)	0.78	-0.02 (-0.08, 0.05)	0.40	0.92	NA
	Potassium (urine)	327,082	-0.04 (-0.09, 0.01)	0.15	-0.03 (-0.08, 0.02)	0.23	-0.02 (-0.12, 0.09)	0.64	-0.08 (-0.57, 0.41)	0.50	-0.04 (-0.12, 0.05)	0.20	0.71	NA
	Albumin	294,657	-0.00 (-0.00, 0.00)	0.88	0.00 (-0.00, 0.01)	0.73	0.00 (-0.01, 0.01)	0.76	0.00 (-0.04, 0.04)	0.87	-0.00 (-0.01, 0.01)	0.88	0.84	NA
	Direct Bilirubin	273,743	0.00 (0.00, 0.00)	5.6E-03	0.00 (0.00, 0.00)	0.05	0.00 (-0.00, 0.00)	0.28	0.00 (-0.01, 0.01)	0.79	0.00 (0.00, 0.00)	5.4E-03	0.67	NA
	Total Bilirubin	320,401	-0.00 (-0.01, 0.00)	0.31	-0.00 (-0.01, 0.00)	0.31	-0.00 (-0.02, 0.01)	0.39	-0.00 (-0.05, 0.04)	0.64	-0.00 (-0.01, 0.00)	0.14	0.82	NA
	Gamma Glutamyltransferase	321,588	0.07 (0.02, 0.12)	8.8E-03	0.06 (0.00, 0.12)	0.05	0.06 (-0.05, 0.16)	0.15	-0.09 (-0.50, 0.31)	0.38	0.07 (-0.01, 0.15)	0.04	0.14	NA
	Alanine aminotransferase	321,623	0.03 (0.01, 0.04)	7.9E-04	0.03 (0.01, 0.05)	0.01	0.03 (-0.02, 0.07)	0.09	0.01 (-0.13, 0.15)	0.78	0.03 (0.00, 0.05)	0.01	0.60	NA
	Aspartate aminotransferase	320,556	0.01 (-0.00, 0.02)	0.12	0.01 (-0.00, 0.03)	0.17	0.01 (-0.02, 0.04)	0.26	-0.01 (-0.11, 0.09)	0.72	0.01 (-0.01, 0.03)	0.14	0.45	NA
	Augmentation index for PWA	20,387	0.02 (-0.01, 0.06)	0.23	0.04 (-0.01, 0.09)	0.11	0.05 (-0.07, 0.17)	0.23	0.07 (-0.26, 0.40)	0.39	0.02 (-0.03, 0.08)	0.24	0.54	NA
	Central augmentation pressure during PWA	20,169	0.03 (-0.00, 0.06)	0.08	0.04 (-0.00, 0.08)	0.08	0.05 (-0.05, 0.14)	0.19	0.09 (-0.18, 0.37)	0.21	0.03 (-0.02, 0.07)	0.09	0.36	NA
	Mean carotid IMT (intima-medial thickness) at 240°	17,863	-0.81 (-1.50, -0.11)	0.02	-0.93 (-1.74, -0.12)	0.02	-0.81 (-2.84, 1.23)	0.25	-1.60 (-7.90, 4.70)	0.33	-0.81 (-1.93, 0.32)	0.06	0.60	NA
	Cardiac output (L/min)	3,350	-0.00 (-0.01, 0.01)	0.46	-0.01 (-0.02, 0.01)	0.45	-0.01 (-0.04, 0.02)	0.46	-0.03 (-0.13, 0.06)	0.18	-0.00 (-0.02, 0.01)	0.40	0.22	NA
	LV end systolic volume (mL)	3,358	0.05 (-0.15, 0.25)	0.61	0.03 (-0.21, 0.26)	0.82	0.02 (-0.46, 0.50)	0.90	-0.67 (-2.28, 0.95)	0.14	0.05 (-0.27, 0.38)	0.63	0.10	NA
	LV end diastolic volume (mL)	3,362	-0.10 (-0.44, 0.24)	0.55	-0.14 (-0.54, 0.27)	0.51	-0.14 (-0.90, 0.62)	0.57	-1.20 (-3.89, 1.49)	0.11	-0.10 (-0.65, 0.45)	0.57	0.13	NA
	LV ejection fraction (%)	3,331	-0.05 (-0.12, 0.01)	0.09	-0.03 (-0.10, 0.04)	0.37	-0.03 (-0.18, 0.12)	0.51	0.11 (-0.43, 0.65)	0.42	-0.05 (-0.13, 0.02)	0.05	0.23	NA
	Total brain volume	27,117	0.01 (-0.22, 0.24)	0.93	0.16 (-0.14, 0.45)	0.30	0.19 (-0.40, 0.79)	0.35	0.44 (-1.55, 2.44)	0.38	0.01 (-0.35, 0.37)	0.93	0.38	NA
	Grey matter volume	27,117	-0.05 (-0.19, 0.09)	0.46	-0.11 (-0.29, 0.07)	0.23	-0.19 (-0.69, 0.30)	0.26	-0.09 (-1.35, 1.17)	0.77	-0.05 (-0.27, 0.16)	0.47	0.91	NA
	White matter volume	27,117	0.06 (-0.10, 0.23)	0.44	0.07 (-0.13, 0.27)	0.49	0.07 (-0.43, 0.58)	0.67	0.53 (-0.75, 1.82)	0.13	0.06 (-0.20, 0.33)	0.47	0.17	NA
	Hippocampal volume	27,106	-0.00 (-0.01, 0.00)	0.44	-0.00 (-0.01, 0.00)	0.07	-0.00 (-0.01, 0.01)	0.30	-0.01 (-0.05, 0.04)	0.60	-0.00 (-0.01, 0.01)	0.47	0.71	NA
	White matter hyperintensity volume	26,783	0.04 (0.01, 0.07)	0.02	0.05 (0.01, 0.09)	0.02	0.06 (-0.05, 0.17)	0.13	-0.01 (-0.28, 0.26)	0.88	0.04 (-0.01, 0.08)	0.03	0.47	NA
	Body mass index (BMI)	336,381	0.00 (-0.00, 0.01)	0.14	0.00 (-0.00, 0.01)	0.17	0.00 (-0.01, 0.02)	0.33	0.01 (-0.04, 0.05)	0.63	0.00 (0.00, 0.01)	4.5E-04	0.90	NA
	Body fat percentage (%)	331,383	0.01 (0.00, 0.02)	2.2E-03	0.01 (0.00, 0.02)	0.03	0.01 (-0.01, 0.03)	0.22	0.01 (-0.05, 0.07)	0.38	0.01 (0.00, 0.02)	1.2E-03	0.86	NA
	Forced expiratory volume (FEV1)	255,647	-1.49 (-2.21, -0.78)	4.3E-05	-1.49 (-2.31, -0.67)	3.6E-04	-1.47 (-3.33, 0.40)	0.05	-1.13 (-7.26, 5.00)	0.46	-1.49 (-1.97, -1.02)	5.8E-05	0.80	NA
	Forced vital capacity (FVC)	255,647	-1.42 (-2.29, -0.54)	1.5E-03	-1.40 (-2.51, -0.30)	0.01	-1.33 (-3.63, 0.98)	0.12	-0.93 (-8.45, 6.58)	0.62	-1.42 (-2.65, -0.19)	0.01	0.79	NA
	FEV1/FVC ratio	255,647	-0.00 (-0.00, 0.00)	0.11	-0.00 (-0.00, -0.00)	0.04	-0.00 (-0.00, 0.00)	0.16	-0.00 (-0.00, 0.00)	0.64	-0.00 (-0.00, 0.00)	0.16	0.91	NA
	Systolic blood pressure	307,868	0.00 (-0.02, 0.03)	0.77	0.02 (-0.01, 0.05)	0.20	0.02 (-0.03, 0.08)	0.26	0.04 (-0.21, 0.29)	0.54	0.00 (-0.04, 0.05)	0.78	0.57	NA
	Diastolic blood pressure	307,876	-0.00 (-0.02, 0.02)	0.94	0.01 (-0.01, 0.02)	0.54	0.01 (-0.03, 0.04)	0.60	0.02 (-0.13, 0.18)	0.57	-0.00 (-0.03, 0.03)	0.94	0.55	NA

Supplementary Table 12: MR analyses using MR IVW, weighted medium, weighted mode, MR-Egger and MR-PRESSO approaches, for all 52 serum, urine, cardiac imaging, brain imaging and clinical biomarkers, using the HMGCR genetic instrument.

			**MR IVW**		**Weighted median**		**Weighted mode**		**MR-Egger**		**MR-PRESSO**			
**GRS**	**Biomarker**	**Sample size, n**	**beta (95% CI)**	**P**	**beta (95% CI)**	**P**	**beta (95% CI)**	**P**	**beta (95% CI)**	**P**	**beta (95% CI)**	**P**	**$P_{pleiotropy}$**	**$P_{distortion}$**
HMGCR	Cholesterol	321,748	-0.03 (-0.03, -0.03)	1.7E-132	-0.03 (-0.03, -0.03)	1.6E-73	-0.03 (-0.03, -0.02)	1.7E-05	-0.04 (-0.06, -0.01)	4.3E-03	-0.03 (-0.03, -0.02)	2.1E-06	0.25	NA
	Direct Low-Density Lipoprotein	321,141	-0.02 (-0.02, -0.02)	4.6E-170	-0.02 (-0.03, -0.02)	5.8E-75	-0.02 (-0.03, -0.02)	1.5E-05	-0.03 (-0.05, -0.01)	3.0E-03	-0.02 (-0.02, -0.02)	1.1E-06	0.25	NA
	HDL-Cholesterol	294,531	-0.00 (-0.00, -0.00)	7.5E-07	-0.00 (-0.00, -0.00)	1.0E-05	-0.00 (-0.00, -0.00)	0.01	-0.00 (-0.01, 0.01)	0.73	-0.00 (-0.00, -0.00)	4.3E-03	0.71	NA
	Triglyceride	321,484	-0.00 (-0.00, 0.00)	0.17	-0.00 (-0.00, 0.00)	0.13	-0.00 (-0.00, 0.00)	0.14	-0.01 (-0.03, 0.01)	0.05	-0.00 (-0.00, 0.00)	0.22	0.07	NA
	Apolipoprotein A	292,911	0.00 (0.00, 0.00)	2.7E-03	0.00 (0.00, 0.00)	0.04	0.00 (-0.00, 0.00)	0.20	-0.00 (-0.00, 0.00)	0.92	0.00 (0.00, 0.00)	0.01	0.57	NA
	Apolipoprotein B	320,189	-0.01 (-0.01, -0.00)	5.3E-154	-0.01 (-0.01, -0.00)	1.8E-62	-0.01 (-0.01, -0.00)	2.1E-05	-0.01 (-0.01, -0.00)	1.8E-03	-0.01 (-0.01, -0.00)	1.4E-06	0.14	NA
	C-reactive Protein	321,051	0.00 (-0.00, 0.01)	0.19	0.00 (-0.00, 0.01)	0.46	0.00 (-0.01, 0.01)	0.54	-0.02 (-0.09, 0.05)	0.32	0.00 (-0.00, 0.01)	0.22	0.24	NA
	Lipoprotein (a)	255,820	-0.11 (-0.18, -0.04)	2.5E-03	-0.11 (-0.19, -0.02)	0.01	-0.11 (-0.28, 0.06)	0.10	-0.06 (-0.98, 0.86)	0.78	-0.11 (-0.14, -0.09)	2.6E-05	0.83	NA
	Vitamin D	307,609	-0.04 (-0.07, -0.01)	3.5E-03	-0.04 (-0.07, 0.00)	0.03	-0.04 (-0.11, 0.02)	0.09	0.00 (-0.35, 0.35)	0.97	-0.04 (-0.08, -0.00)	0.02	0.61	NA
	Rheumatoid factor	28,705	0.04 (-0.04, 0.13)	0.34	0.05 (-0.06, 0.17)	0.35	0.06 (-0.15, 0.28)	0.38	0.22 (-0.92, 1.37)	0.44	0.04 (-0.10, 0.18)	0.38	0.52	NA
	Alkaline Phosphatase	321,762	-0.07 (-0.11, -0.04)	1.9E-05	-0.07 (-0.11, -0.02)	2.4E-03	-0.06 (-0.15, 0.02)	0.06	-0.06 (-0.50, 0.37)	0.56	-0.07 (-0.12, -0.03)	2.6E-03	0.92	NA
	Calcium	294,549	-0.00 (-0.00, -0.00)	2.1E-15	-0.00 (-0.00, -0.00)	1.4E-09	-0.00 (-0.00, -0.00)	3.2E-03	-0.00 (-0.00, 0.00)	0.14	-0.00 (-0.00, -0.00)	1.4E-04	0.66	NA
	SHBG	291,824	-0.11 (-0.14, -0.08)	3.9E-10	-0.13 (-0.17, -0.09)	3.0E-09	-0.13 (-0.22, -0.05)	4.7E-03	-0.18 (-0.62, 0.26)	0.15	-0.11 (-0.16, -0.06)	1.1E-03	0.53	NA
	Testosterone	291,707	-0.01 (-0.01, -0.01)	9.0E-09	-0.01 (-0.01, -0.01)	1.8E-05	-0.01 (-0.02, -0.00)	0.02	-0.01 (-0.06, 0.04)	0.47	-0.01 (-0.01, -0.01)	2.2E-05	0.86	NA
	Oestradiol	50,478	-1.57 (-2.92, -0.22)	0.02	-1.78 (-3.40, -0.16)	0.03	-1.95 (-5.31, 1.41)	0.12	-3.16 (-20.48, 14.15)	0.48	-1.57 (-2.70, -0.44)	6.8E-03	0.71	NA
	IGF-1	320,022	-0.01 (-0.02, -0.00)	0.01	-0.01 (-0.02, 0.00)	0.10	-0.01 (-0.02, 0.01)	0.24	-0.01 (-0.10, 0.08)	0.74	-0.01 (-0.02, 0.00)	0.04	0.95	NA
	Glycated Haemoglobin (HbA1c)	321,610	0.03 (0.01, 0.04)	3.7E-06	0.03 (0.02, 0.04)	1.4E-06	0.03 (0.01, 0.04)	6.0E-03	0.01 (-0.15, 0.16)	0.89	0.03 (0.01, 0.04)	5.7E-03	0.58	NA
	Glucose	294,346	0.00 (-0.00, 0.00)	0.08	0.00 (-0.00, 0.00)	0.16	0.00 (-0.00, 0.01)	0.29	-0.00 (-0.02, 0.02)	0.90	0.00 (0.00, 0.00)	6.4E-03	0.68	NA
	Cystatin C	321,727	0.00 (-0.00, 0.00)	0.19	0.00 (-0.00, 0.00)	0.14	0.00 (-0.00, 0.00)	0.22	-0.00 (-0.00, 0.00)	0.94	0.00 (-0.00, 0.00)	0.25	0.78	NA
	Creatinine	321,583	0.00 (0.00, 0.00)	0.02	0.00 (0.00, 0.00)	0.01	0.00 (-0.00, 0.00)	0.06	0.00 (-0.00, 0.00)	0.26	0.00 (-0.00, 0.00)	0.07	0.41	NA
	Total protein	294,331	-0.01 (-0.01, 0.00)	0.06	-0.01 (-0.01, -0.00)	0.02	-0.01 (-0.02, 0.00)	0.09	-0.03 (-0.10, 0.04)	0.11	-0.01 (-0.02, 0.00)	0.12	0.17	NA
	Urea	321,537	0.00 (-0.00, 0.00)	0.25	0.00 (-0.00, 0.00)	0.22	0.00 (-0.00, 0.01)	0.25	0.00 (-0.02, 0.03)	0.50	0.00 (-0.00, 0.00)	0.06	0.61	NA
	Phosphate	294,096	0.00 (-0.00, 0.00)	0.92	-0.00 (-0.00, 0.00)	0.92	-0.00 (-0.00, 0.00)	0.81	-0.00 (-0.00, 0.00)	0.25	0.00 (-0.00, 0.00)	0.89	0.24	NA
	Urate	321,334	-0.06 (-0.15, 0.03)	0.17	-0.05 (-0.15, 0.05)	0.35	-0.05 (-0.25, 0.15)	0.48	-0.11 (-1.24, 1.02)	0.69	-0.06 (-0.16, 0.04)	0.10	0.86	NA
	Creatinine (urine)	327,791	13.74 (6.74, 20.74)	1.2E-04	14.81 (5.88, 23.75)	1.2E-03	16.82 (-0.16, 33.80)	0.03	53.32 (-35.74, 142.38)	0.06	13.74 (2.37, 25.11)	0.01	0.12	NA
	Sodium (urine)	327,096	0.18 (0.13, 0.23)	6.4E-11	0.17 (0.10, 0.23)	1.9E-06	0.16 (0.04, 0.29)	9.0E-03	0.06 (-0.63, 0.75)	0.73	0.18 (0.12, 0.24)	2.3E-04	0.49	NA
	Potassium (urine)	327,082	0.05 (0.01, 0.10)	0.02	0.04 (-0.02, 0.09)	0.17	0.04 (-0.06, 0.13)	0.25	0.25 (-0.31, 0.80)	0.13	0.05 (-0.02, 0.12)	0.07	0.20	NA
	Albumin	294,657	-0.00 (-0.01, -0.00)	0.02	-0.00 (-0.01, -0.00)	0.05	-0.00 (-0.01, 0.00)	0.15	-0.00 (-0.05, 0.04)	0.70	-0.00 (-0.01, -0.00)	2.9E-03	0.98	NA
	Direct Bilirubin	273,743	0.00 (0.00, 0.00)	1.2E-06	0.00 (0.00, 0.00)	1.7E-04	0.00 (-0.00, 0.01)	0.03	0.00 (-0.01, 0.02)	0.70	0.00 (0.00, 0.00)	2.2E-03	0.70	NA
	Total Bilirubin	320,401	-0.00 (-0.01, 0.00)	0.13	-0.00 (-0.01, 0.00)	0.21	-0.00 (-0.02, 0.01)	0.32	-0.00 (-0.07, 0.07)	1.00	-0.00 (-0.01, 0.00)	0.07	0.81	NA
	Gamma Glutamyltransferase	321,588	0.01 (-0.04, 0.07)	0.60	0.01 (-0.05, 0.08)	0.69	0.01 (-0.11, 0.14)	0.76	-0.10 (-0.79, 0.58)	0.55	0.01 (-0.04, 0.07)	0.45	0.49	NA
	Alanine aminotransferase	321,623	0.01 (-0.01, 0.03)	0.33	0.01 (-0.01, 0.03)	0.34	0.01 (-0.03, 0.05)	0.49	0.00 (-0.22, 0.23)	0.99	0.01 (0.00, 0.01)	5.3E-03	0.88	NA
	Aspartate aminotransferase	320,556	-0.02 (-0.04, -0.01)	4.5E-04	-0.02 (-0.04, -0.01)	8.1E-03	-0.02 (-0.05, 0.02)	0.15	0.02 (-0.16, 0.19)	0.67	-0.02 (-0.04, -0.01)	2.0E-03	0.34	NA
	Augmentation index for PWA	20,387	-0.02 (-0.07, 0.02)	0.27	-0.02 (-0.07, 0.03)	0.41	-0.01 (-0.11, 0.09)	0.78	0.06 (-0.50, 0.61)	0.69	-0.02 (-0.07, 0.02)	0.11	0.56	NA
	Central augmentation pressure during PWA	20,169	0.02 (-0.02, 0.06)	0.27	0.02 (-0.03, 0.06)	0.50	0.02 (-0.07, 0.10)	0.52	-0.04 (-0.51, 0.43)	0.72	0.02 (-0.03, 0.07)	0.21	0.59	NA
	Mean carotid IMT (intima-medial thickness) at 240°	17,863	-0.54 (-1.26, 0.18)	0.14	-0.61 (-1.48, 0.25)	0.16	-0.64 (-2.45, 1.18)	0.31	-1.43 (-10.67, 7.81)	0.54	-0.54 (-1.16, 0.09)	0.04	0.69	NA
	Cardiac output (L/min)	3,350	-0.01 (-0.02, 0.00)	0.05	-0.01 (-0.02, 0.01)	0.34	-0.01 (-0.03, 0.02)	0.46	0.04 (-0.11, 0.19)	0.32	-0.01 (-0.03, 0.00)	0.06	0.21	NA
	LV end systolic volume (mL)	3,358	0.15 (-0.06, 0.36)	0.16	0.12 (-0.14, 0.37)	0.36	0.12 (-0.37, 0.61)	0.47	-0.33 (-3.01, 2.35)	0.62	0.15 (-0.14, 0.44)	0.15	0.47	NA
	LV end diastolic volume (mL)	3,362	0.10 (-0.25, 0.45)	0.58	0.14 (-0.30, 0.57)	0.53	0.14 (-0.66, 0.93)	0.61	-0.04 (-4.51, 4.43)	0.97	0.10 (-0.29, 0.49)	0.45	0.90	NA
	LV ejection fraction (%)	3,331	-0.06 (-0.14, 0.01)	0.10	-0.03 (-0.12, 0.05)	0.44	-0.03 (-0.18, 0.12)	0.56	0.22 (-0.66, 1.11)	0.34	-0.06 (-0.18, 0.06)	0.16	0.23	NA
	Total brain volume	27,117	0.31 (-0.02, 0.65)	0.07	0.15 (-0.19, 0.49)	0.38	0.11 (-0.45, 0.66)	0.57	-0.90 (-4.94, 3.13)	0.39	0.31 (-0.24, 0.86)	0.13	0.26	NA
	Grey matter volume	27,117	0.14 (-0.01, 0.30)	0.07	0.11 (-0.09, 0.31)	0.26	0.08 (-0.31, 0.47)	0.56	0.09 (-2.09, 2.28)	0.86	0.14 (-0.10, 0.39)	0.12	0.92	NA
	White matter volume	27,117	0.17 (-0.05, 0.39)	0.13	0.01 (-0.21, 0.23)	0.93	0.04 (-0.34, 0.41)	0.77	-1.00 (-3.16, 1.17)	0.12	0.17 (-0.19, 0.53)	0.19	0.08	NA
	Hippocampal volume	27,106	0.01 (0.00, 0.01)	6.1E-03	0.00 (-0.00, 0.01)	0.13	0.00 (-0.01, 0.01)	0.29	-0.00 (-0.06, 0.05)	0.92	0.01 (0.00, 0.01)	0.02	0.59	NA
	White matter hyperintensity volume	26,783	0.02 (-0.02, 0.05)	0.30	0.02 (-0.02, 0.06)	0.34	0.02 (-0.06, 0.10)	0.42	-0.02 (-0.47, 0.43)	0.89	0.02 (-0.03, 0.06)	0.24	0.76	NA
	Body mass index (BMI)	336,381	0.04 (0.03, 0.06)	1.3E-10	0.04 (0.03, 0.05)	3.5E-19	0.04 (0.02, 0.06)	6.3E-04	0.01 (-0.17, 0.18)	0.90	0.04 (0.02, 0.07)	1.3E-03	0.38	NA
	Body fat percentage (%)	331,383	0.05 (0.03, 0.07)	2.0E-07	0.05 (0.03, 0.06)	1.4E-13	0.05 (0.02, 0.07)	1.0E-03	-0.01 (-0.24, 0.23)	0.92	0.05 (0.02, 0.08)	3.5E-03	0.37	NA
	Forced expiratory volume (FEV1)	255,647	-0.32 (-1.24, 0.60)	0.50	-0.23 (-1.18, 0.72)	0.64	-0.24 (-2.18, 1.71)	0.71	-2.40 (-14.77, 9.98)	0.45	-0.32 (-1.81, 1.18)	0.53	0.50	NA
	Forced vital capacity (FVC)	255,647	-0.74 (-1.91, 0.43)	0.22	-0.46 (-1.68, 0.76)	0.46	-0.29 (-2.66, 2.08)	0.71	-1.62 (-18.31, 15.08)	0.70	-0.74 (-2.65, 1.17)	0.27	0.83	NA
	FEV1/FVC ratio	255,647	0.00 (-0.00, 0.00)	0.14	0.00 (-0.00, 0.00)	0.27	0.00 (-0.00, 0.00)	0.57	-0.00 (-0.00, 0.00)	0.36	0.00 (-0.00, 0.00)	0.10	0.26	NA
	Systolic blood pressure	307,868	0.02 (-0.01, 0.05)	0.11	0.03 (-0.00, 0.06)	0.06	0.03 (-0.03, 0.09)	0.21	-0.13 (-0.42, 0.17)	0.14	0.02 (-0.02, 0.07)	0.17	0.09	NA
	Diastolic blood pressure	307,876	-0.00 (-0.02, 0.01)	0.77	-0.01 (-0.03, 0.01)	0.31	-0.01 (-0.04, 0.02)	0.32	-0.06 (-0.26, 0.14)	0.26	-0.00 (-0.03, 0.02)	0.78	0.27	NA

Supplementary Table 13: MR analyses using MR IVW, weighted medium, weighted mode, MR-Egger and MR-PRESSO approaches, for all 52 serum, urine, cardiac imaging, brain imaging and clinical biomarkers, using the NPC1L1 genetic instrument.

			MR IVW		Weighted median		Weighted mode		MR-Egger		MR-PRESSO			
GRS	Biomarker	Sample size, n	beta (95% CI)	P	beta (95% CI)	P	beta (95% CI)	P	beta (95% CI)	P	beta (95% CI)	P	$P_{pleiotropy}$	$P_{distortion}$
NPC1L1	Cholesterol	321,748	-0.02 (-0.02, -0.02)	4.2E-27	-0.02 (-0.03, -0.01)	9.2E-14	-0.02 (-0.03, -0.01)	7.8E-03	-0.03 (-0.09, 0.04)	0.25	-0.02 (-0.02, -0.02)	2.9E-04	0.74	NA
	Direct Low-Density Lipoprotein	321,141	-0.02 (-0.02, -0.01)	2.8E-36	-0.02 (-0.02, -0.01)	1.3E-16	-0.02 (-0.03, -0.01)	5.2E-03	-0.02 (-0.08, 0.03)	0.25	-0.02 (-0.02, -0.01)	6.9E-04	0.84	NA
	HDL-Cholesterol	294,531	0.00 (0.00, 0.00)	2.3E-03	0.00 (0.00, 0.00)	3.1E-04	0.00 (-0.00, 0.01)	0.07	-0.00 (-0.03, 0.02)	0.72	0.00 (-0.00, 0.00)	0.05	0.53	NA
	Triglyceride	321,484	-0.00 (-0.01, 0.00)	0.05	-0.00 (-0.01, 0.00)	0.08	-0.00 (-0.01, 0.00)	0.18	0.03 (-0.03, 0.09)	0.18	-0.00 (-0.01, 0.00)	0.15	0.15	NA
	Apolipoprotein A	292,911	0.00 (0.00, 0.00)	2.1E-04	0.00 (0.00, 0.00)	8.7E-04	0.00 (-0.00, 0.00)	0.05	0.00 (-0.01, 0.02)	0.78	0.00 (0.00, 0.00)	0.01	0.93	NA
	Apolipoprotein B	320,189	-0.00 (-0.00, -0.00)	1.9E-26	-0.00 (-0.01, -0.00)	4.7E-14	-0.00 (-0.01, -0.00)	8.1E-03	-0.00 (-0.02, 0.01)	0.30	-0.00 (-0.00, -0.00)	6.0E-04	0.85	NA
	C-reactive Protein	321,051	0.01 (-0.02, 0.04)	0.42	0.00 (-0.01, 0.02)	0.63	-0.00 (-0.03, 0.03)	0.82	0.05 (-0.67, 0.77)	0.80	0.01 (-0.04, 0.06)	0.48	0.85	NA
	Lipoprotein (a)	255,820	-0.00 (-0.17, 0.17)	0.98	0.03 (-0.18, 0.24)	0.78	0.02 (-0.35, 0.39)	0.85	1.00 (-2.41, 4.41)	0.34	-0.00 (-0.24, 0.23)	0.98	0.33	NA
	Vitamin D	307,609	-0.01 (-0.10, 0.08)	0.81	-0.05 (-0.13, 0.03)	0.24	0.06 (-0.09, 0.22)	0.27	0.04 (-2.04, 2.12)	0.94	-0.01 (-0.15, 0.13)	0.82	0.93	NA
	Rheumatoid factor	28,705	-0.01 (-0.21, 0.20)	0.93	0.04 (-0.20, 0.27)	0.76	0.05 (-0.38, 0.48)	0.72	0.17 (-3.89, 4.24)	0.87	-0.01 (-0.28, 0.26)	0.92	0.86	NA
	Alkaline Phosphatase	321,762	-0.09 (-0.23, 0.04)	0.17	-0.07 (-0.19, 0.05)	0.23	-0.20 (-0.40, -0.01)	0.05	0.06 (-3.10, 3.23)	0.94	-0.09 (-0.31, 0.12)	0.26	0.85	NA
	Calcium	294,549	-0.00 (-0.00, 0.00)	0.53	-0.00 (-0.00, 0.00)	0.81	-0.00 (-0.00, 0.00)	0.07	-0.00 (-0.01, 0.01)	0.98	-0.00 (-0.00, 0.00)	0.57	0.98	NA
	SHBG	291,824	-0.08 (-0.24, 0.08)	0.34	-0.11 (-0.22, -0.01)	0.03	-0.10 (-0.28, 0.08)	0.17	-1.20 (-2.96, 0.57)	0.10	-0.08 (-0.34, 0.18)	0.41	0.11	NA
	Testosterone	291,707	-0.01 (-0.02, 0.01)	0.34	-0.01 (-0.02, 0.00)	0.15	-0.01 (-0.03, 0.01)	0.25	-0.11 (-0.28, 0.06)	0.11	-0.01 (-0.03, 0.02)	0.41	0.12	NA
	Oestradiol	50,478	-0.65 (-4.41, 3.12)	0.74	0.27 (-3.64, 4.18)	0.89	1.03 (-5.66, 7.72)	0.66	20.60 (-43.42, 84.62)	0.30	-0.65 (-6.77, 5.47)	0.76	0.29	NA
	IGF-1	320,022	0.01 (-0.03, 0.05)	0.58	0.01 (-0.01, 0.04)	0.42	0.04 (-0.00, 0.08)	0.06	0.10 (-0.77, 0.98)	0.67	0.01 (-0.05, 0.07)	0.62	0.70	NA
	Glycated Haemoglobin (HbA1c)	321,610	0.05 (0.03, 0.08)	2.5E-05	0.04 (0.02, 0.07)	5.4E-04	0.04 (-0.00, 0.08)	0.06	0.18 (-0.29, 0.65)	0.25	0.05 (0.01, 0.09)	0.02	0.37	NA
	Glucose	294,346	0.00 (-0.00, 0.01)	0.57	0.00 (-0.00, 0.01)	0.83	-0.00 (-0.01, 0.01)	0.76	0.04 (-0.04, 0.12)	0.16	0.00 (-0.01, 0.01)	0.61	0.17	NA
	Cystatin C	321,727	-0.00 (-0.00, 0.00)	0.57	0.00 (-0.00, 0.00)	0.99	0.00 (-0.00, 0.00)	0.78	0.00 (-0.01, 0.01)	0.93	-0.00 (-0.00, 0.00)	0.53	0.88	NA
	Creatinine	321,583	0.00 (-0.00, 0.00)	0.73	0.00 (-0.00, 0.00)	0.33	0.00 (-0.00, 0.00)	0.18	-0.00 (-0.00, 0.00)	0.73	0.00 (-0.00, 0.00)	0.75	0.71	NA
	Total protein	294,331	-0.00 (-0.02, 0.02)	0.97	0.00 (-0.02, 0.02)	0.92	-0.02 (-0.05, 0.01)	0.16	-0.02 (-0.52, 0.48)	0.87	-0.00 (-0.03, 0.03)	0.97	0.87	NA
	Urea	321,537	0.00 (-0.00, 0.01)	0.09	0.00 (-0.00, 0.01)	0.10	0.00 (-0.00, 0.01)	0.18	0.01 (-0.07, 0.09)	0.59	0.00 (-0.00, 0.01)	0.08	0.70	NA
	Phosphate	294,096	-0.00 (-0.00, 0.00)	0.50	-0.00 (-0.00, 0.00)	0.72	0.00 (-0.00, 0.00)	0.74	0.00 (-0.01, 0.01)	0.65	-0.00 (-0.00, 0.00)	0.52	0.61	NA
	Urate	321,334	0.28 (0.07, 0.49)	0.01	0.28 (0.03, 0.53)	0.03	0.32 (-0.16, 0.79)	0.13	0.52 (-3.67, 4.71)	0.65	0.28 (0.05, 0.51)	0.03	0.83	NA
	Creatinine (urine)	327,791	17.07 (0.37, 33.76)	0.05	20.24 (0.59, 39.89)	0.04	20.81 (-17.38, 59.01)	0.18	-44.09 (-373.59, 285.41)	0.62	17.07 (-0.93, 35.06)	0.06	0.51	NA
	Sodium (urine)	327,096	-0.05 (-0.18, 0.08)	0.45	-0.08 (-0.23, 0.07)	0.31	-0.08 (-0.38, 0.22)	0.45	-0.72 (-3.26, 1.82)	0.35	-0.05 (-0.21, 0.11)	0.41	0.37	NA
	Potassium (urine)	327,082	0.06 (-0.04, 0.17)	0.24	0.07 (-0.05, 0.18)	0.26	0.07 (-0.15, 0.30)	0.38	-0.26 (-2.30, 1.78)	0.63	0.06 (-0.03, 0.15)	0.11	0.56	NA
	Albumin	294,657	-0.01 (-0.02, 0.01)	0.37	-0.00 (-0.02, 0.01)	0.52	-0.02 (-0.03, 0.00)	0.08	-0.01 (-0.31, 0.29)	0.88	-0.01 (-0.03, 0.01)	0.44	0.94	NA
	Direct Bilirubin	273,743	0.00 (-0.00, 0.00)	0.99	-0.00 (-0.00, 0.00)	0.61	-0.00 (-0.01, 0.01)	0.66	0.01 (-0.04, 0.07)	0.43	0.00 (-0.00, 0.00)	0.99	0.43	NA
	Total Bilirubin	320,401	-0.01 (-0.02, 0.01)	0.24	-0.01 (-0.02, 0.01)	0.50	-0.01 (-0.03, 0.02)	0.61	0.01 (-0.25, 0.28)	0.84	-0.01 (-0.02, 0.00)	0.14	0.75	NA
	Gamma Glutamyltransferase	321,588	-0.06 (-0.21, 0.09)	0.44	-0.02 (-0.17, 0.13)	0.79	-0.04 (-0.31, 0.23)	0.68	1.01 (-1.51, 3.53)	0.23	-0.06 (-0.30, 0.18)	0.49	0.21	NA
	Alanine aminotransferase	321,623	-0.05 (-0.14, 0.05)	0.32	-0.04 (-0.10, 0.03)	0.24	-0.09 (-0.21, 0.03)	0.10	0.50 (-0.91, 1.92)	0.27	-0.05 (-0.19, 0.10)	0.40	0.23	NA
	Aspartate aminotransferase	320,556	-0.00 (-0.06, 0.06)	0.99	-0.03 (-0.07, 0.02)	0.25	-0.04 (-0.10, 0.03)	0.17	0.19 (-1.25, 1.64)	0.62	-0.00 (-0.11, 0.10)	0.99	0.62	NA
	Augmentation index for PWA	20,387	0.01 (-0.11, 0.12)	0.88	0.06 (-0.07, 0.18)	0.38	0.07 (-0.15, 0.28)	0.40	-0.24 (-2.87, 2.39)	0.73	0.01 (-0.18, 0.19)	0.89	0.72	NA
	Central augmentation pressure during PWA	20,169	-0.04 (-0.13, 0.05)	0.38	-0.01 (-0.11, 0.10)	0.91	-0.00 (-0.19, 0.18)	0.97	-0.25 (-1.97, 1.47)	0.60	-0.04 (-0.15, 0.07)	0.33	0.65	NA
	Mean carotid IMT (intima-medial thickness) at 240°	17,863	0.35 (-1.39, 2.08)	0.69	0.50 (-1.42, 2.42)	0.61	0.63 (-3.12, 4.38)	0.63	3.28 (-31.01, 37.57)	0.72	0.35 (-0.64, 1.33)	0.35	0.75	NA
	Cardiac output (L/min)	3,350	0.00 (-0.04, 0.04)	0.86	0.01 (-0.03, 0.04)	0.73	-0.03 (-0.09, 0.04)	0.25	-0.03 (-1.02, 0.96)	0.91	0.00 (-0.06, 0.07)	0.87	0.90	NA
	LV end systolic volume (mL)	3,358	-0.64 (-1.15, -0.12)	0.01	-0.57 (-1.15, 0.02)	0.06	-0.47 (-1.56, 0.63)	0.27	-2.68 (-12.84, 7.48)	0.37	-0.64 (-1.38, 0.11)	0.07	0.48	NA
	LV end diastolic volume (mL)	3,362	-0.69 (-1.55, 0.16)	0.11	-0.84 (-1.82, 0.14)	0.09	-0.84 (-2.72, 1.04)	0.25	-4.06 (-21.01, 12.90)	0.41	-0.69 (-1.47, 0.09)	0.07	0.48	NA
	LV ejection fraction (%)	3,331	0.13 (-0.14, 0.39)	0.35	0.04 (-0.16, 0.25)	0.67	0.01 (-0.36, 0.38)	0.95	0.60 (-5.75, 6.95)	0.72	0.13 (-0.31, 0.56)	0.42	0.78	NA
	Total brain volume	27,117	-0.14 (-0.83, 0.55)	0.70	0.01 (-0.74, 0.77)	0.97	0.19 (-1.15, 1.53)	0.68	-1.21 (-17.51, 15.08)	0.78	-0.14 (-1.26, 0.98)	0.72	0.80	NA
	Grey matter volume	27,117	0.05 (-0.33, 0.42)	0.82	0.15 (-0.30, 0.59)	0.52	0.24 (-0.56, 1.03)	0.41	-0.94 (-8.41, 6.53)	0.64	0.05 (-0.45, 0.54)	0.79	0.63	NA
	White matter volume	27,117	-0.18 (-0.59, 0.23)	0.38	-0.13 (-0.63, 0.36)	0.60	0.01 (-0.84, 0.86)	0.98	-0.28 (-10.14, 9.59)	0.91	-0.18 (-0.85, 0.48)	0.45	0.97	NA
	Hippocampal volume	27,106	0.00 (-0.01, 0.01)	0.77	0.00 (-0.01, 0.01)	0.78	0.01 (-0.01, 0.03)	0.34	0.02 (-0.19, 0.23)	0.73	0.00 (-0.01, 0.02)	0.76	0.75	NA
	White matter hyperintensity volume	26,783	-0.03 (-0.13, 0.07)	0.54	-0.00 (-0.10, 0.10)	0.98	0.00 (-0.18, 0.19)	0.95	0.69 (-0.97, 2.34)	0.22	-0.03 (-0.19, 0.13)	0.58	0.20	NA
	Body mass index (BMI)	336,381	-0.01 (-0.05, 0.03)	0.68	-0.01 (-0.03, 0.01)	0.26	-0.02 (-0.05, 0.02)	0.23	0.18 (-0.60, 0.95)	0.43	-0.01 (-0.07, 0.06)	0.71	0.41	NA
	Body fat percentage (%)	331,383	-0.01 (-0.06, 0.04)	0.59	-0.02 (-0.05, 0.01)	0.18	-0.03 (-0.08, 0.02)	0.15	0.21 (-0.80, 1.22)	0.46	-0.01 (-0.10, 0.07)	0.63	0.44	NA
	Forced expiratory volume (FEV1)	255,647	-1.17 (-4.22, 1.88)	0.45	-0.09 (-2.38, 2.20)	0.94	-0.08 (-3.93, 3.78)	0.95	18.56 (-23.75, 60.87)	0.20	-1.17 (-6.13, 3.79)	0.51	0.18	NA
	Forced vital capacity (FVC)	255,647	-0.93 (-3.88, 2.02)	0.54	0.06 (-2.73, 2.84)	0.97	0.08 (-4.86, 5.03)	0.96	19.09 (-27.65, 65.83)	0.22	-0.93 (-5.72, 3.86)	0.58	0.20	NA
	FEV1/FVC ratio	255,647	-0.00 (-0.00, 0.00)	0.67	0.00 (-0.00, 0.00)	0.93	0.00 (-0.00, 0.00)	0.92	0.00 (-0.00, 0.01)	0.27	-0.00 (-0.00, 0.00)	0.70	0.25	NA
	Systolic blood pressure	307,868	-0.05 (-0.11, -0.00)	0.06	-0.04 (-0.11, 0.02)	0.21	-0.04 (-0.16, 0.08)	0.34	0.18 (-0.90, 1.27)	0.54	-0.05 (-0.11, 0.00)	0.06	0.44	NA
	Diastolic blood pressure	307,876	0.03 (-0.02, 0.09)	0.25	0.02 (-0.02, 0.08)	0.23	0.07 (-0.02, 0.15)	0.09	-0.28 (-1.33, 0.77)	0.37	-0.03 (-0.77, 1.33)	0.33	0.32	NA

Supplementary Table 14: MR analyses using MR IVW, weighted medium, weighted mode, MR-Egger and MR-PRESSO approaches, for all 52 serum, urine, cardiac imaging, brain imaging and clinical biomarkers, using the LDLR genetic instrument.

			MR IVW		Weighted median		Weighted mode		MR-Egger		MR-PRESSO			
**GRS**	**Biomarker**	**Sample size, n**	**beta (95% CI)**	**P**	**beta (95% CI)**	**P**	**beta (95% CI)**	**P**	**beta (95% CI)**	**P**	**beta (95% CI)**	**P**	**$P_{pleiotropy}$**	**$P_{distortion}$**
LDLR	Cholesterol	321,748	-0.03 (-0.03, -0.02)	6.1E-38	-0.03 (-0.03, -0.03)	2.0E-246	-0.03 (-0.03, -0.02)	8.2E-04	-0.03 (-0.04, -0.02)	0.07	NA	NA	0.42	NA
	Direct Low-Density Lipoprotein	321,141	-0.02 (-0.02, -0.02)	4.4E-58	-0.02 (-0.02, -0.02)	1.2E-289	-0.02 (-0.02, -0.02)	6.9E-04	-0.02 (-0.03, -0.02)	0.06	NA	NA	0.43	NA
	HDL-Cholesterol	294,531	0.00 (-0.00, 0.00)	0.57	0.00 (-0.00, 0.00)	0.52	0.00 (-0.00, 0.00)	0.97	-0.00 (-0.00, 0.00)	0.37	NA	NA	0.23	NA
	Triglyceride	321,484	-0.00 (-0.00, 0.00)	0.32	-0.00 (-0.00, 0.00)	0.26	-0.00 (-0.00, 0.00)	0.36	-0.00 (-0.00, 0.00)	0.45	NA	NA	0.60	NA
	Apolipoprotein A	292,911	0.00 (0.00, 0.00)	7.2E-09	0.00 (0.00, 0.00)	1.1E-14	0.00 (0.00, 0.00)	0.03	0.00 (-0.00, 0.00)	0.20	NA	NA	0.29	NA
	Apolipoprotein B	320,189	-0.01 (-0.01, -0.01)	6.5E-67	-0.01 (-0.01, -0.01)	7.9E-280	-0.01 (-0.01, -0.01)	8.7E-04	-0.01 (-0.01, -0.00)	0.06	NA	NA	0.55	NA
	C-reactive Protein	321,051	-0.00 (-0.00, 0.00)	0.88	-0.00 (-0.00, 0.00)	0.95	0.00 (-0.01, 0.01)	1.00	-0.00 (-0.02, 0.02)	0.98	NA	NA	0.97	NA
	Lipoprotein (a)	255,820	-0.09 (-0.14, -0.04)	1.0E-03	-0.10 (-0.15, -0.04)	6.4E-04	-0.10 (-0.20, 0.00)	0.09	-0.11 (-0.32, 0.10)	0.26	NA	NA	0.68	NA
	Vitamin D	307,609	0.07 (0.03, 0.12)	1.4E-03	0.08 (0.06, 0.11)	1.4E-13	0.09 (0.05, 0.12)	0.02	0.11 (-0.04, 0.26)	0.20	NA	NA	0.42	NA
	Rheumatoid factor	28,705	0.03 (-0.03, 0.09)	0.36	0.04 (-0.02, 0.11)	0.21	0.04 (-0.07, 0.16)	0.35	0.06 (-0.19, 0.31)	0.51	NA	NA	0.68	NA
	Alkaline Phosphatase	321,762	-0.03 (-0.06, -0.00)	0.03	-0.02 (-0.05, 0.00)	0.07	-0.02 (-0.07, 0.02)	0.25	-0.01 (-0.15, 0.12)	0.72	NA	NA	0.63	NA
	Calcium	294,549	0.00 (-0.00, 0.00)	0.33	0.00 (-0.00, 0.00)	0.42	0.00 (-0.00, 0.00)	0.57	0.00 (-0.00, 0.00)	0.90	NA	NA	0.72	NA
	SHBG	291,824	0.02 (-0.01, 0.05)	0.13	0.02 (-0.01, 0.05)	0.21	0.02 (-0.03, 0.06)	0.37	0.01 (-0.09, 0.11)	0.70	NA	NA	0.75	NA
	Testosterone	291,707	0.00 (-0.00, 0.00)	0.29	0.00 (-0.00, 0.00)	0.21	0.00 (-0.00, 0.01)	0.34	0.00 (-0.01, 0.01)	0.49	NA	NA	0.69	NA
	Oestradiol	50,478	0.50 (-0.57, 1.56)	0.36	0.32 (-0.71, 1.35)	0.54	0.21 (-1.53, 1.94)	0.74	-0.54 (-4.48, 3.41)	0.66	NA	NA	0.40	NA
	IGF-1	320,022	-0.02 (-0.03, 0.00)	0.07	-0.02 (-0.02, -0.01)	1.0E-07	-0.02 (-0.03, -0.01)	0.04	-0.02 (-0.11, 0.07)	0.53	NA	NA	0.87	NA
	Glycated Haemoglobin (HbA1c)	321,610	0.00 (-0.00, 0.01)	0.54	0.00 (-0.00, 0.01)	0.44	0.00 (-0.01, 0.01)	0.55	0.00 (-0.02, 0.03)	0.63	NA	NA	0.77	NA
	Glucose	294,346	0.00 (-0.00, 0.00)	0.46	0.00 (-0.00, 0.00)	0.50	0.00 (-0.00, 0.00)	0.75	-0.00 (-0.00, 0.00)	0.96	NA	NA	0.67	NA
	Cystatin C	321,727	-0.00 (-0.00, 0.00)	0.87	-0.00 (-0.00, 0.00)	0.83	-0.00 (-0.00, 0.00)	0.84	-0.00 (-0.00, 0.00)	0.84	NA	NA	0.87	NA
	Creatinine	321,583	0.00 (-0.00, 0.00)	0.07	0.00 (-0.00, 0.00)	0.11	0.00 (-0.00, 0.00)	0.32	0.00 (-0.00, 0.00)	0.69	NA	NA	0.83	NA
	Total protein	294,331	-0.00 (-0.01, 0.00)	0.23	-0.00 (-0.01, 0.00)	0.12	-0.01 (-0.01, 0.00)	0.13	-0.01 (-0.03, 0.01)	0.23	NA	NA	0.29	NA
	Urea	321,537	0.00 (-0.00, 0.00)	0.76	-0.00 (-0.00, 0.00)	0.92	-0.00 (-0.00, 0.00)	0.84	-0.00 (-0.01, 0.01)	0.83	NA	NA	0.71	NA
	Phosphate	294,096	0.00 (-0.00, 0.00)	0.66	0.00 (-0.00, 0.00)	0.78	0.00 (-0.00, 0.00)	0.84	-0.00 (-0.00, 0.00)	0.97	NA	NA	0.79	NA
	Urate	321,334	0.06 (-0.01, 0.12)	0.09	0.06 (-0.00, 0.13)	0.07	0.07 (-0.05, 0.19)	0.20	0.11 (-0.15, 0.37)	0.32	NA	NA	0.48	NA
	Creatinine (urine)	327,791	6.04 (-2.16, 14.23)	0.15	4.38 (-1.19, 9.94)	0.12	3.63 (-5.65, 12.92)	0.34	-0.23 (-31.60, 31.15)	0.98	NA	NA	0.49	NA
	Sodium (urine)	327,096	0.08 (0.03, 0.13)	0.00	0.07 (0.02, 0.11)	3.8E-03	0.06 (-0.01, 0.14)	0.12	0.05 (-0.19, 0.29)	0.54	NA	NA	0.67	NA
	Potassium (urine)	327,082	-0.01 (-0.04, 0.02)	0.49	-0.01 (-0.04, 0.02)	0.55	-0.02 (-0.08, 0.04)	0.37	-0.03 (-0.16, 0.09)	0.44	NA	NA	0.51	NA
	Albumin	294,657	0.00 (-0.00, 0.00)	0.10	0.00 (-0.00, 0.01)	0.09	0.00 (-0.00, 0.01)	0.27	0.00 (-0.01, 0.01)	0.46	NA	NA	0.86	NA
	Direct Bilirubin	273,743	0.00 (0.00, 0.00)	9.8E-09	0.00 (0.00, 0.00)	2.6E-07	0.00 (0.00, 0.00)	0.05	0.00 (-0.00, 0.01)	0.25	NA	NA	0.52	NA
	Total Bilirubin	320,401	-0.00 (-0.01, 0.00)	0.36	-0.00 (-0.01, 0.00)	0.38	-0.00 (-0.01, 0.00)	0.27	-0.01 (-0.02, 0.01)	0.34	NA	NA	0.39	NA
	Gamma Glutamyltransferase	321,588	0.03 (-0.05, 0.12)	0.45	0.02 (-0.02, 0.06)	0.38	0.01 (-0.06, 0.08)	0.67	-0.05 (-0.22, 0.12)	0.42	NA	NA	0.24	NA
	Alanine aminotransferase	321,623	0.01 (0.00, 0.03)	0.04	0.01 (0.00, 0.03)	0.05	0.01 (-0.01, 0.04)	0.27	0.01 (-0.04, 0.06)	0.63	NA	NA	0.67	NA
	Aspartate aminotransferase	320,556	0.01 (-0.00, 0.02)	0.13	0.01 (-0.00, 0.02)	0.14	0.01 (-0.01, 0.02)	0.40	-0.00 (-0.04, 0.04)	0.90	NA	NA	0.38	NA
	Augmentation index for PWA	20,387	0.01 (-0.02, 0.04)	0.56	0.01 (-0.03, 0.04)	0.62	0.01 (-0.05, 0.07)	0.69	0.01 (-0.12, 0.13)	0.86	NA	NA	0.92	NA
	Central augmentation pressure during PWA	20,169	0.01 (-0.02, 0.04)	0.46	0.01 (-0.02, 0.03)	0.73	0.00 (-0.04, 0.05)	0.80	-0.01 (-0.11, 0.10)	0.81	NA	NA	0.54	NA
	Mean carotid IMT (intima-medial thickness) at 240°	17,863	-0.54 (-1.08, -0.01)	0.05	-0.53 (-1.08, 0.02)	0.06	-0.52 (-1.52, 0.48)	0.24	-0.50 (-2.60, 1.60)	0.49	NA	NA	0.93	NA
	Cardiac output (L/min)	3,350	-0.00 (-0.01, 0.01)	0.71	-0.01 (-0.02, 0.00)	0.31	-0.01 (-0.02, 0.01)	0.39	-0.01 (-0.07, 0.05)	0.62	NA	NA	0.65	NA
	LV end systolic volume (mL)	3,358	-0.09 (-0.24, 0.07)	0.29	-0.09 (-0.26, 0.07)	0.28	-0.11 (-0.40, 0.19)	0.37	-0.15 (-0.77, 0.48)	0.50	NA	NA	0.71	NA
	LV end diastolic volume (mL)	3,362	-0.10 (-0.36, 0.17)	0.48	-0.11 (-0.38, 0.15)	0.41	-0.14 (-0.63, 0.36)	0.47	-0.22 (-1.26, 0.81)	0.52	NA	NA	0.64	NA
	LV ejection fraction (%)	3,331	0.01 (-0.04, 0.06)	0.65	0.01 (-0.05, 0.06)	0.85	0.01 (-0.10, 0.11)	0.89	-0.00 (-0.21, 0.20)	0.97	NA	NA	0.78	NA
	Total brain volume	27,117	0.10 (-0.21, 0.41)	0.53	0.03 (-0.18, 0.24)	0.78	0.01 (-0.34, 0.36)	0.93	-0.12 (-1.38, 1.13)	0.75	NA	NA	0.53	NA
	Grey matter volume	27,117	-0.03 (-0.18, 0.12)	0.73	-0.04 (-0.17, 0.08)	0.49	-0.07 (-0.29, 0.15)	0.41	-0.16 (-0.63, 0.31)	0.38	NA	NA	0.37	NA
	White matter volume	27,117	0.13 (-0.04, 0.29)	0.14	0.09 (-0.05, 0.23)	0.22	0.08 (-0.16, 0.32)	0.41	0.04 (-0.77, 0.84)	0.87	NA	NA	0.67	NA
	Hippocampal volume	27,106	0.00 (-0.00, 0.00)	0.39	0.00 (-0.00, 0.00)	0.56	0.00 (-0.01, 0.01)	0.70	0.00 (-0.01, 0.01)	0.85	NA	NA	0.81	NA
	White matter hyperintensity volume	26,783	-0.02 (-0.05, 0.00)	0.06	-0.02 (-0.05, 0.01)	0.16	-0.02 (-0.07, 0.03)	0.34	-0.02 (-0.12, 0.09)	0.64	NA	NA	0.71	NA
	Body mass index (BMI)	336,381	0.00 (-0.00, 0.01)	0.43	0.00 (-0.00, 0.01)	0.05	0.01 (-0.00, 0.01)	0.15	0.01 (-0.01, 0.03)	0.24	NA	NA	0.28	NA
	Body fat percentage (%)	331,383	0.00 (-0.00, 0.01)	0.33	0.01 (-0.00, 0.01)	0.09	0.01 (-0.00, 0.02)	0.18	0.01 (-0.01, 0.04)	0.25	NA	NA	0.30	NA
	Forced expiratory volume (FEV1)	255,647	0.95 (-0.53, 2.44)	0.21	0.58 (-0.06, 1.22)	0.07	0.52 (-0.53, 1.58)	0.26	-0.04 (-6.40, 6.31)	0.98	NA	NA	0.56	NA
	Forced vital capacity (FVC)	255,647	1.23 (-0.47, 2.92)	0.16	1.13 (0.35, 1.91)	4.5E-03	0.64 (-0.80, 2.07)	0.29	-0.42 (-4.16, 3.31)	0.71	NA	NA	0.26	NA
	FEV1/FVC ratio	255,647	-0.00 (-0.00, 0.00)	0.97	0.00 (-0.00, 0.00)	0.65	0.00 (-0.00, 0.00)	0.63	0.00 (-0.00, 0.00)	0.79	NA	NA	0.73	NA
	Systolic blood pressure	307,868	-0.02 (-0.05, 0.01)	0.14	-0.02 (-0.04, 0.00)	0.05	-0.02 (-0.05, 0.01)	0.21	-0.02 (-0.16, 0.12)	0.63	NA	NA	0.95	NA
	Diastolic blood pressure	307,876	0.99 (-0.00, 0.02)	0.12	0.99 (0.00, 0.02)	0.07	0.99 (-0.00, 0.03)	0.22	0.99 (-0.02, 0.05)	0.50	NA	NA	0.77	NA

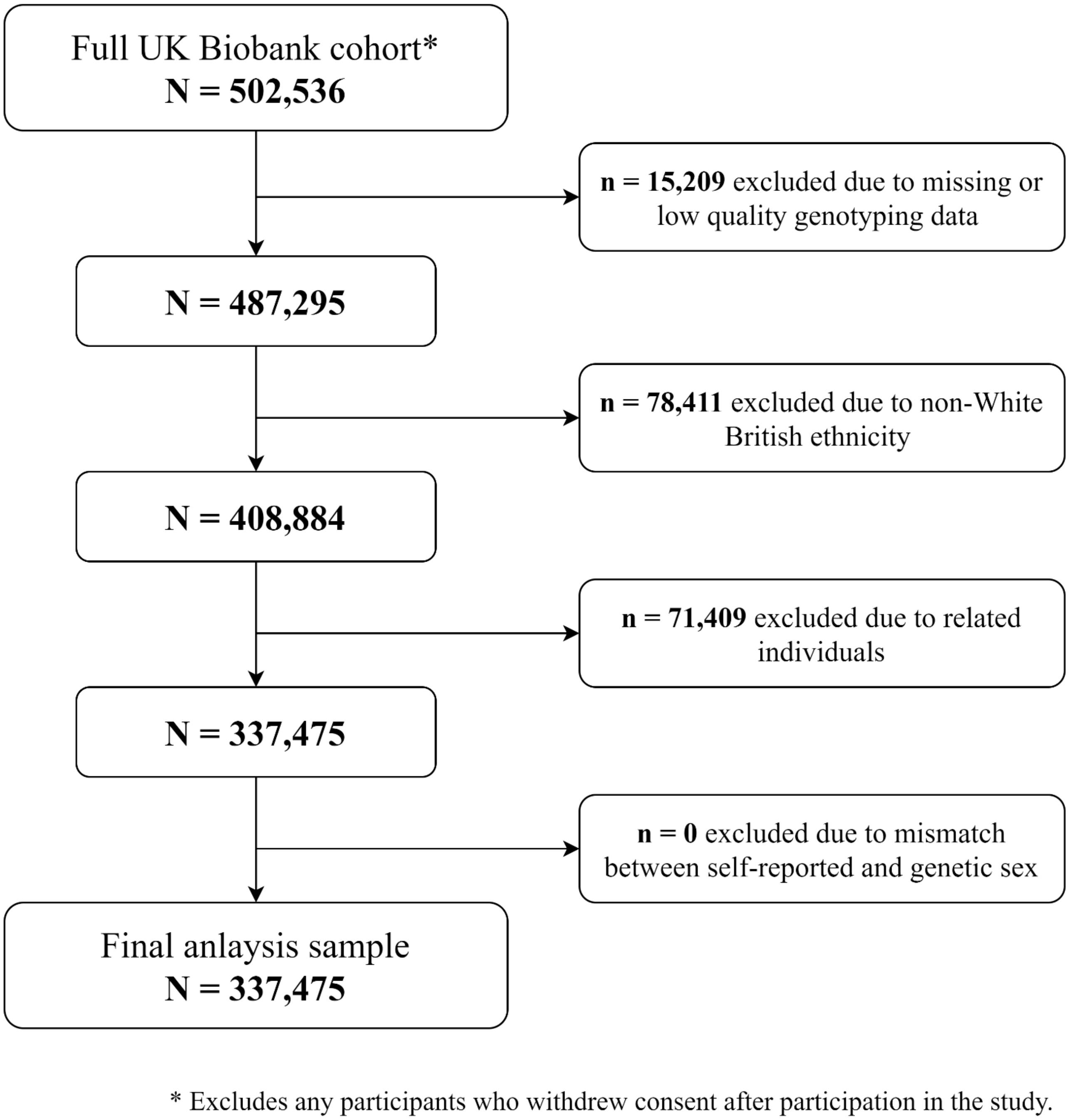

Supplementary Figure 1: Participant flowchart showing the sample restriction from the full UK Biobank cohort to the final analysis sample for the MR-PheWAS analysis.

Unstable angina (411.1)

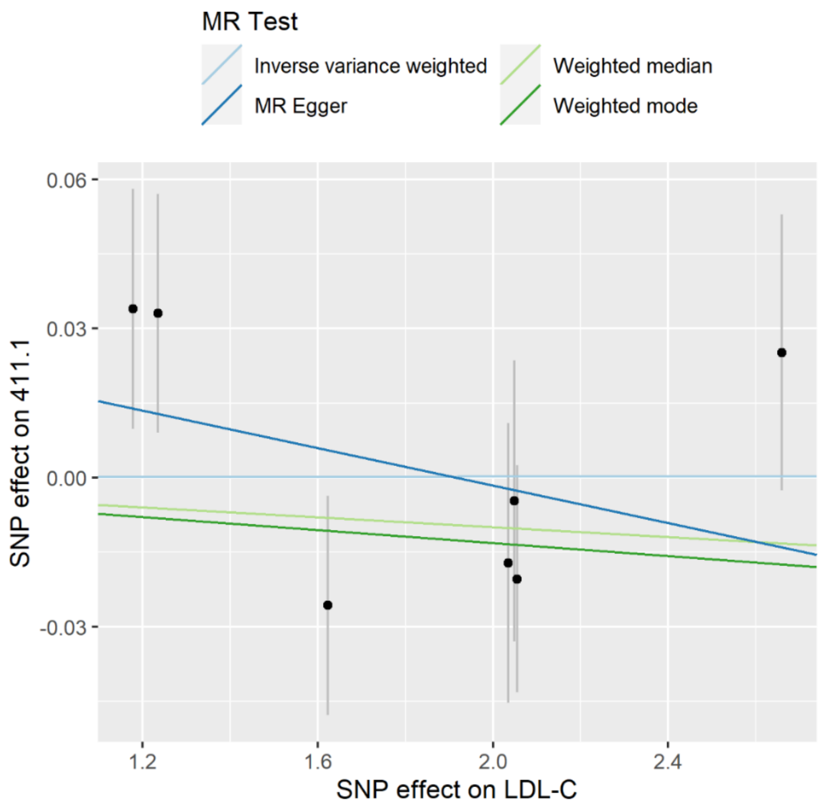

Plot A

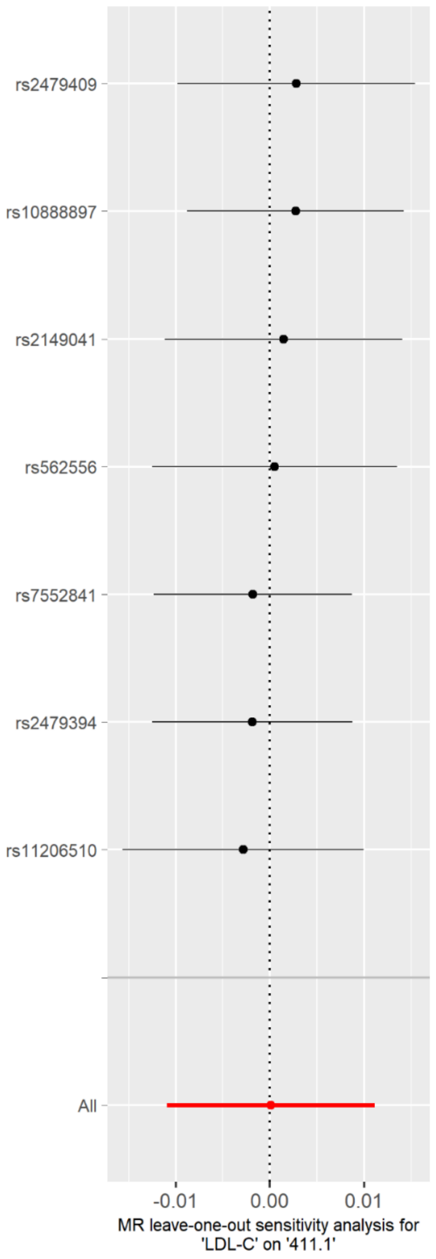

Plot B

Plot A shows the scatter plot of the variant-outcome association estimates for each genetic variant, against the overall estimates for the variant-exposure (i.e. LDL-C lowering) association using different MR methods.

Plot B displays the leave-one-out analysis, which compares the effect estimates when excluding each of the genetic variants in the GRS, one at a time, to the overall estimate including all variants.

Myocardial infarction (411.2)

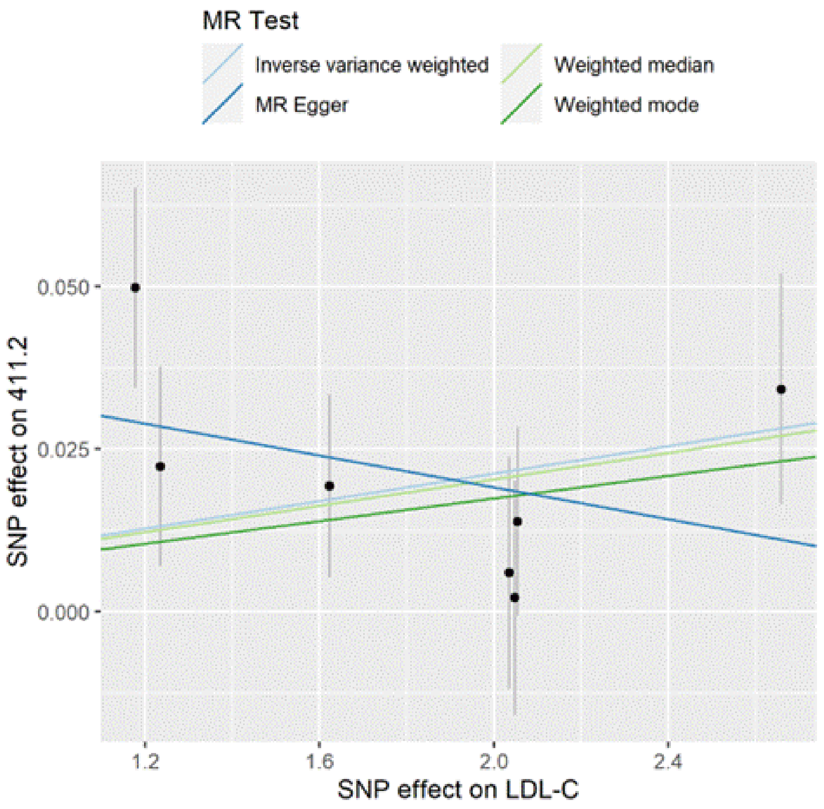

Plot A

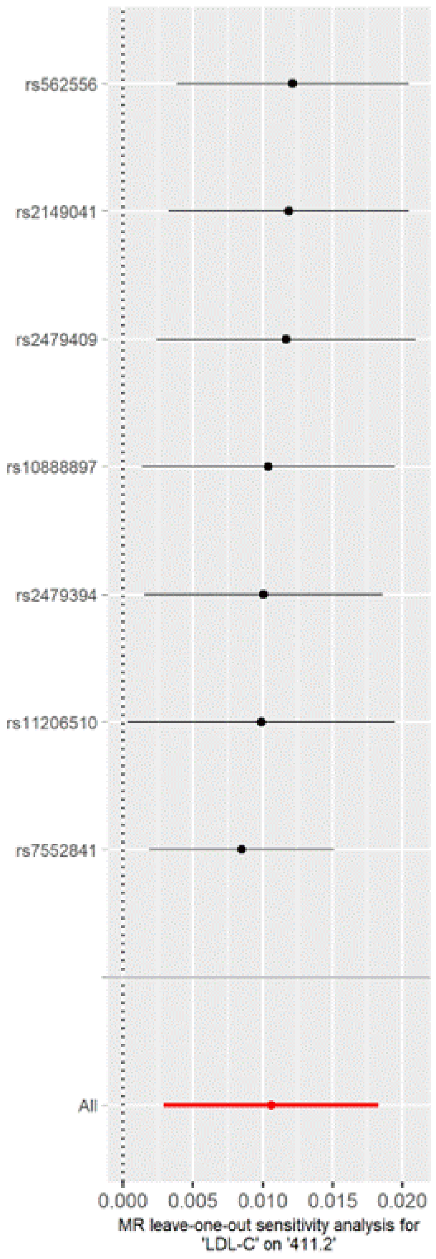

Plot B

Plot A shows the scatter plot of the variant-outcome association estimates for each genetic variant, against the overall estimates for the variant-exposure (i.e. LDL-C lowering) association using different MR methods.

Plot B displays the leave-one-out analysis, which compares the effect estimates when excluding each of the genetic variants in the GRS, one at a time, to the overall estimate including all variants.

Angina pectoris (411.3)

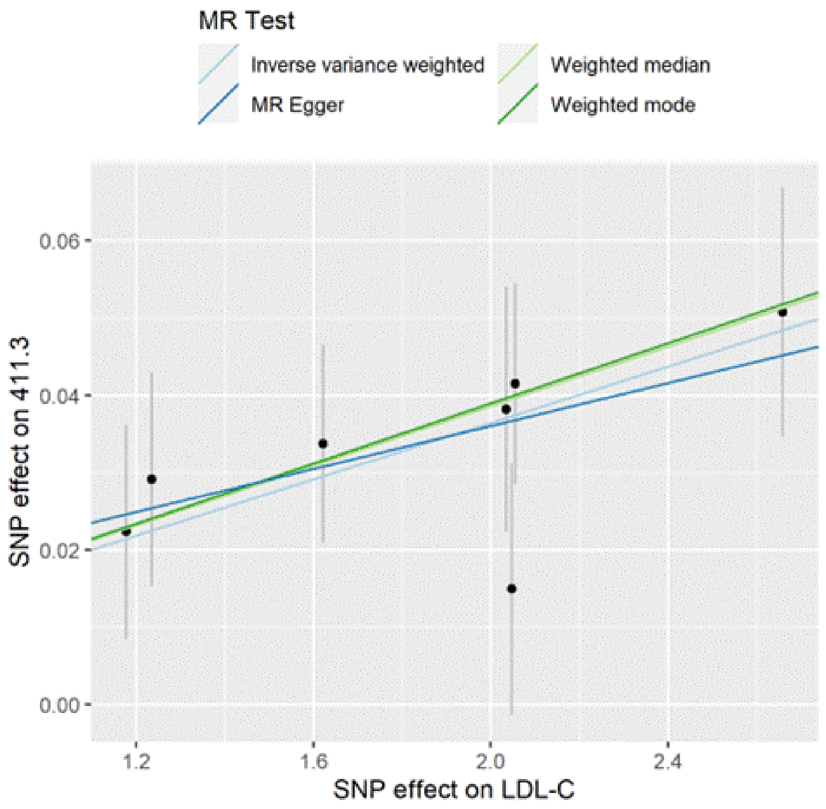

Plot A

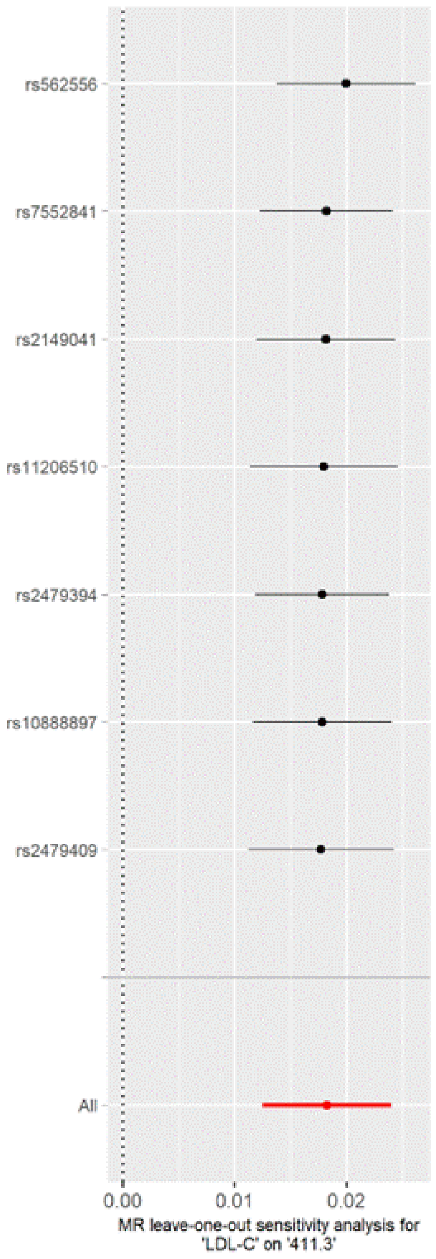

Plot B

Plot A shows the scatter plot of the variant-outcome association estimates for each genetic variant, against the overall estimates for the variant-exposure (i.e. LDL-C lowering) association using different MR methods.

Plot B displays the leave-one-out analysis, which compares the effect estimates when excluding each of the genetic variants in the GRS, one at a time, to the overall estimate including all variants.

Coronary atherosclerosis (411.4)

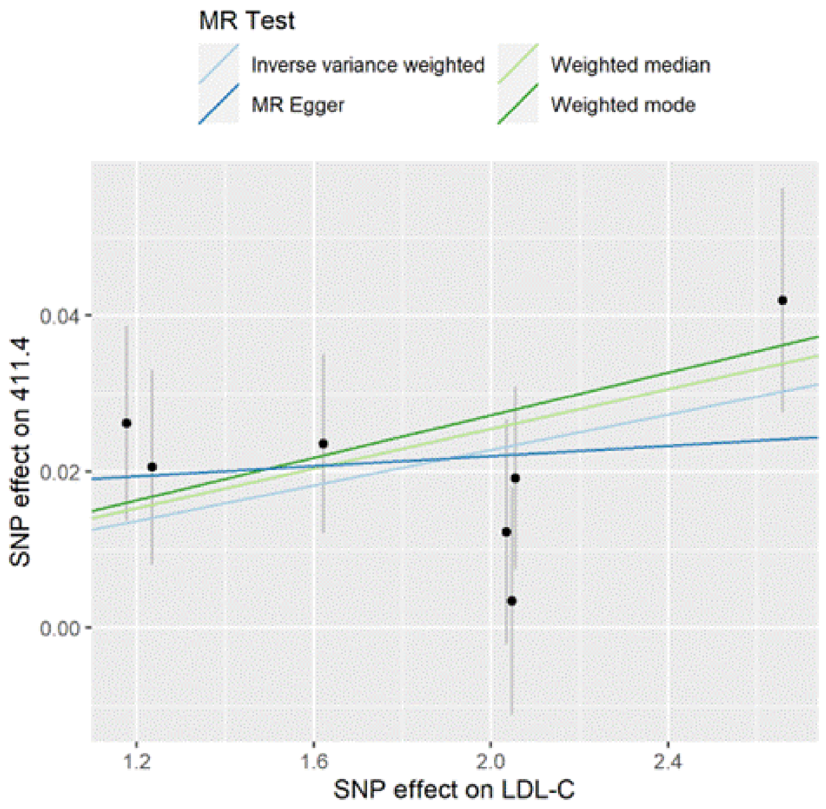

Plot A

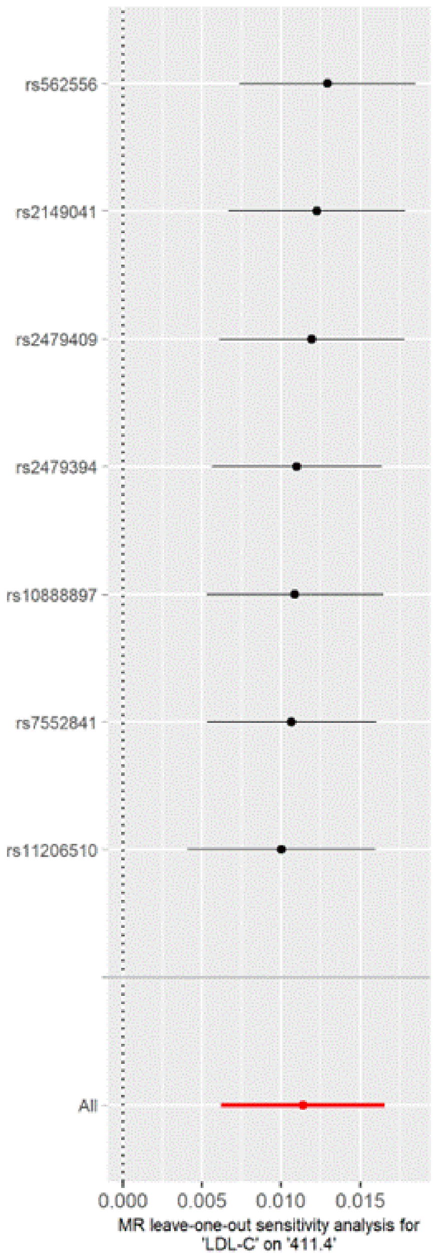

Plot B

Plot A shows the scatter plot of the variant-outcome association estimates for each genetic variant, against the overall estimates for the variant-exposure (i.e. LDL-C lowering) association using different MR methods.

Plot B displays the leave-one-out analysis, which compares the effect estimates when excluding each of the genetic variants in the GRS, one at a time, to the overall estimate including all variants.

Other chronic ischemic heart diseases (411.8)

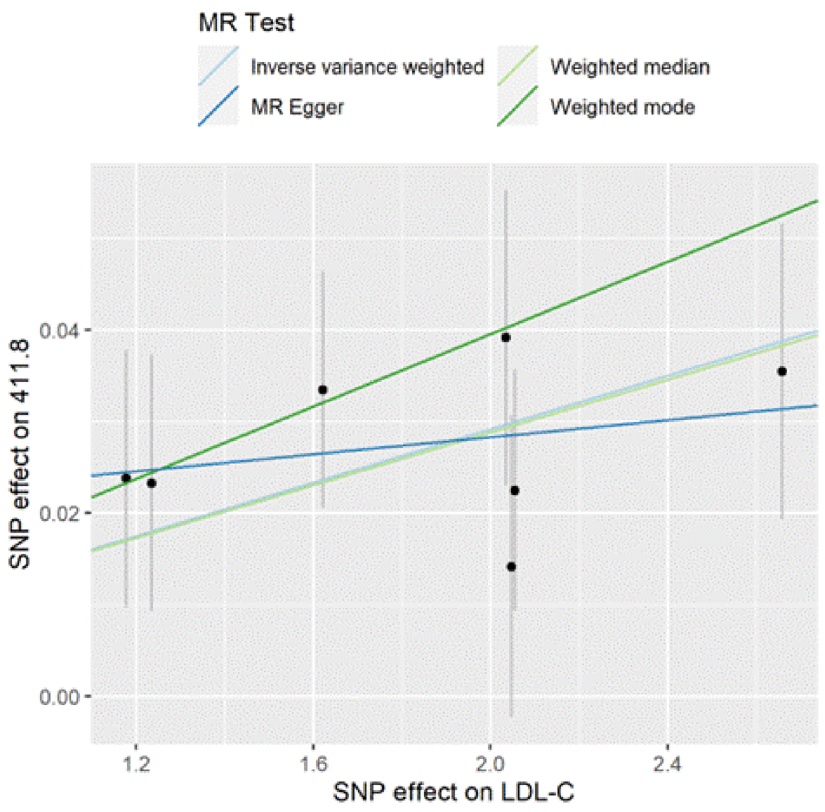

Plot A

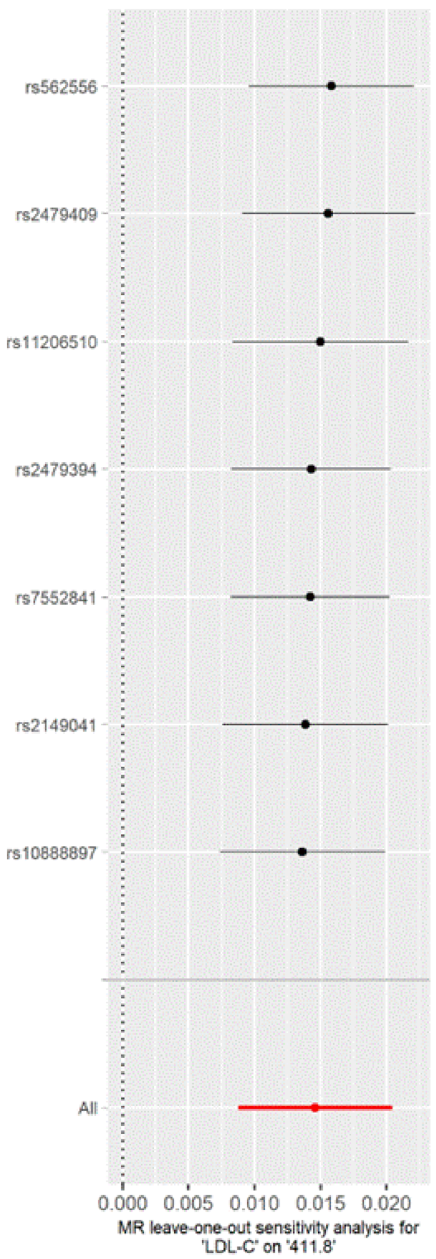

Plot B

Plot A shows the scatter plot of the variant-outcome association estimates for each genetic variant, against the overall estimates for the variant-exposure (i.e. LDL-C lowering) association using different MR methods.

Plot B displays the leave-one-out analysis, which compares the effect estimates when excluding each of the genetic variants in the GRS, one at a time, to the overall estimate including all variants.

Hypertensive heart and/or renal disease (401.2)

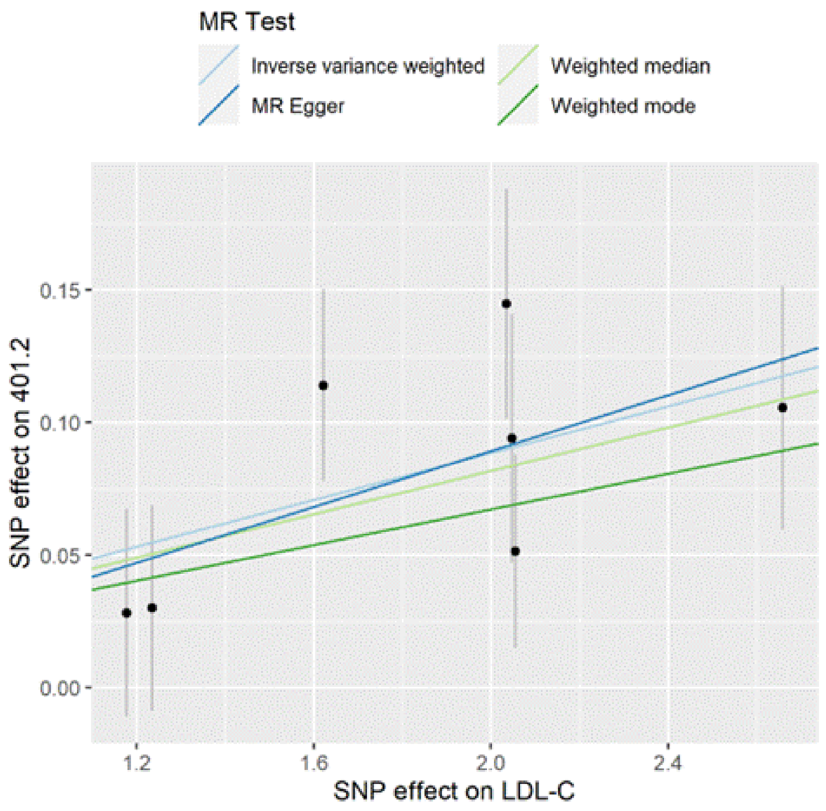

Plot A

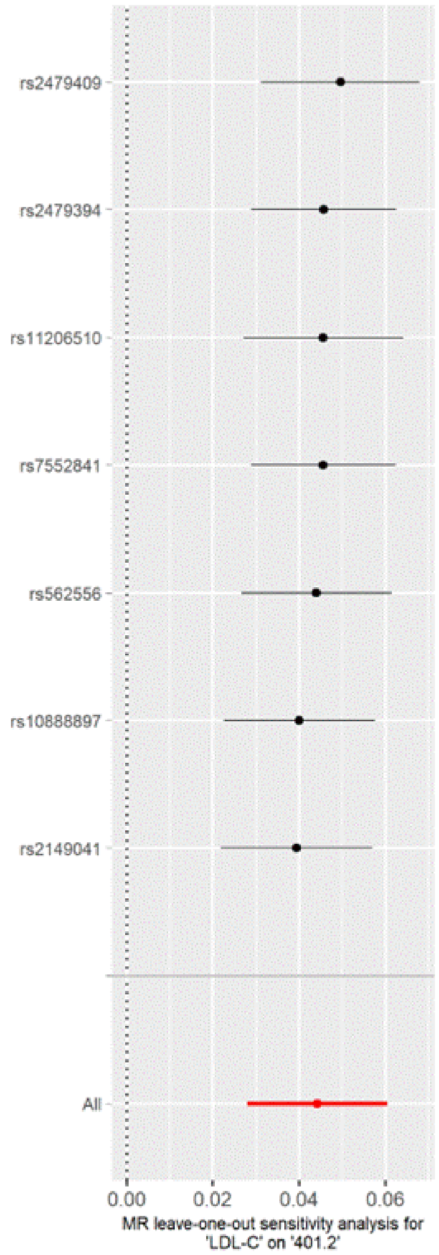

Plot B

Plot A shows the scatter plot of the variant-outcome association estimates for each genetic variant, against the overall estimates for the variant-exposure (i.e. LDL-C lowering) association using different MR methods.

Plot B displays the leave-one-out analysis, which compares the effect estimates when excluding each of the genetic variants in the GRS, one at a time, to the overall estimate including all variants.

Abdominal aortic aneurysm (442.11)

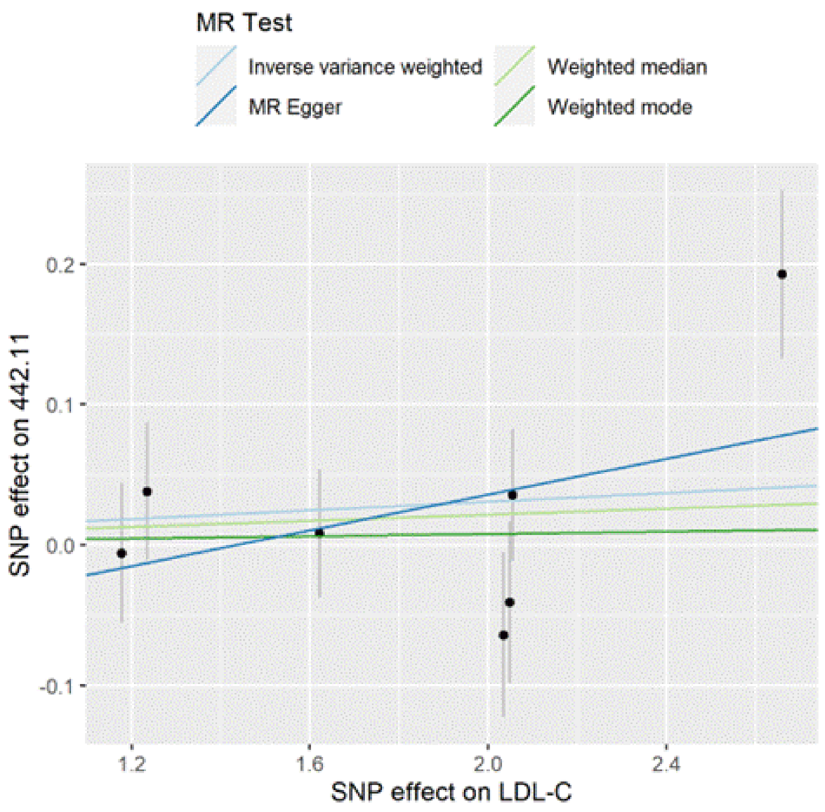

Plot A

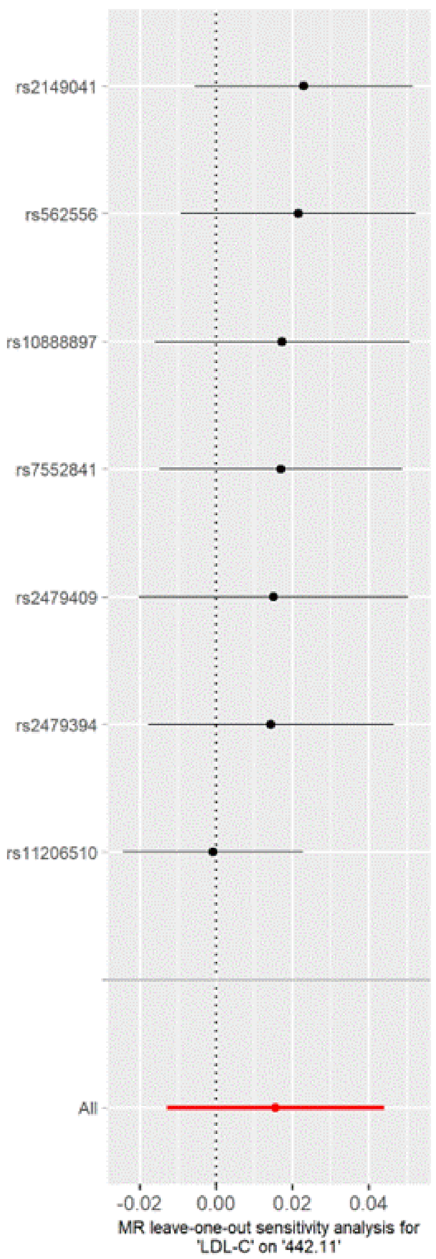

Plot B

Plot A shows the scatter plot of the variant-outcome association estimates for each genetic variant, against the overall estimates for the variant-exposure (i.e. LDL-C lowering) association using different MR methods.

Plot B displays the leave-one-out analysis, which compares the effect estimates when excluding each of the genetic variants in the GRS, one at a time, to the overall estimate including all variants.

Peripheral vascular disease (443)

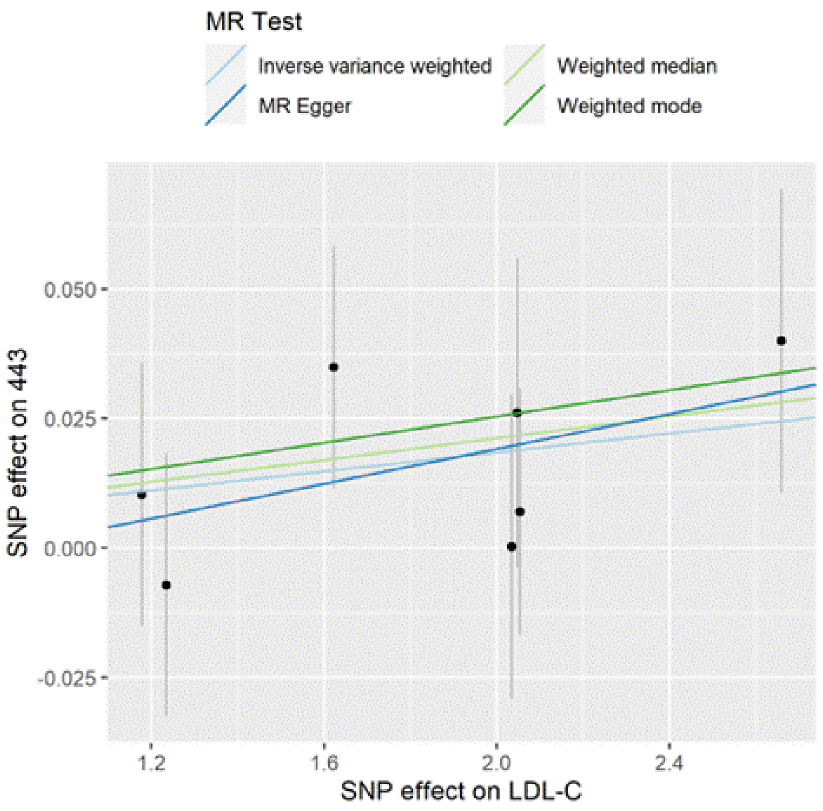

Plot A

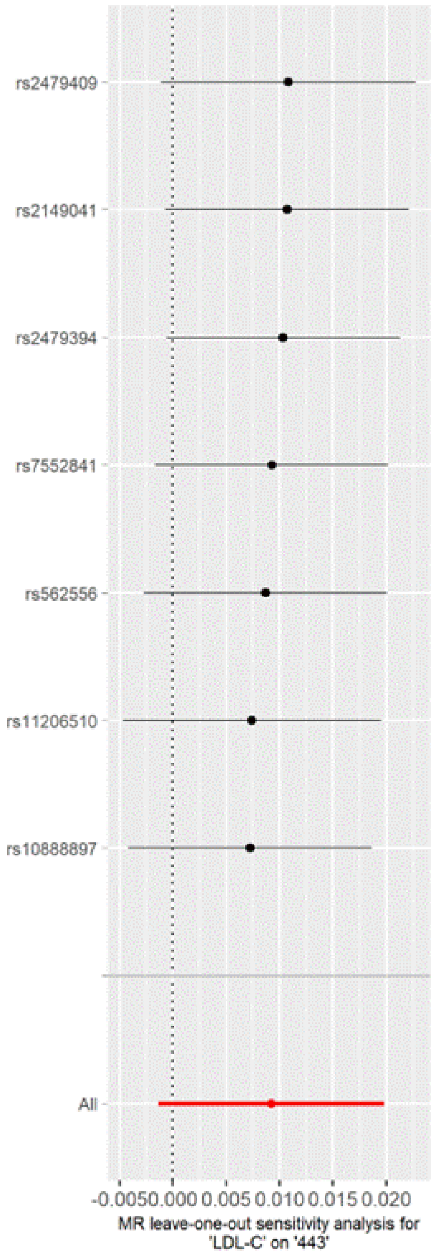

Plot B

Plot A shows the scatter plot of the variant-outcome association estimates for each genetic variant, against the overall estimates for the variant-exposure (i.e. LDL-C lowering) association using different MR methods.

Plot B displays the leave-one-out analysis, which compares the effect estimates when excluding each of the genetic variants in the GRS, one at a time, to the overall estimate including all variants.

Hypercholesterolemia (272.11)

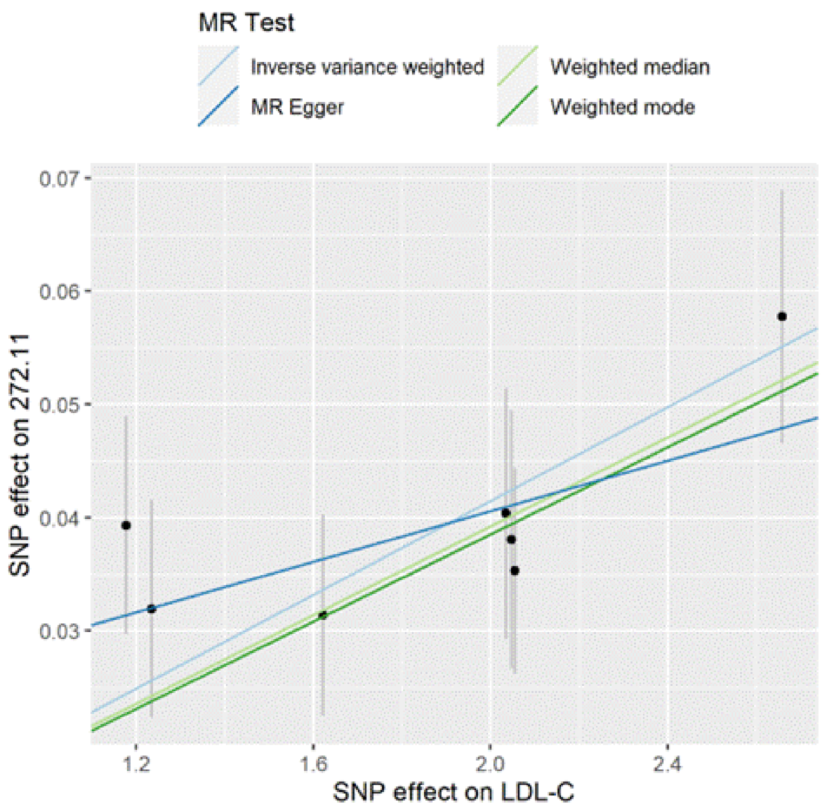

Plot A

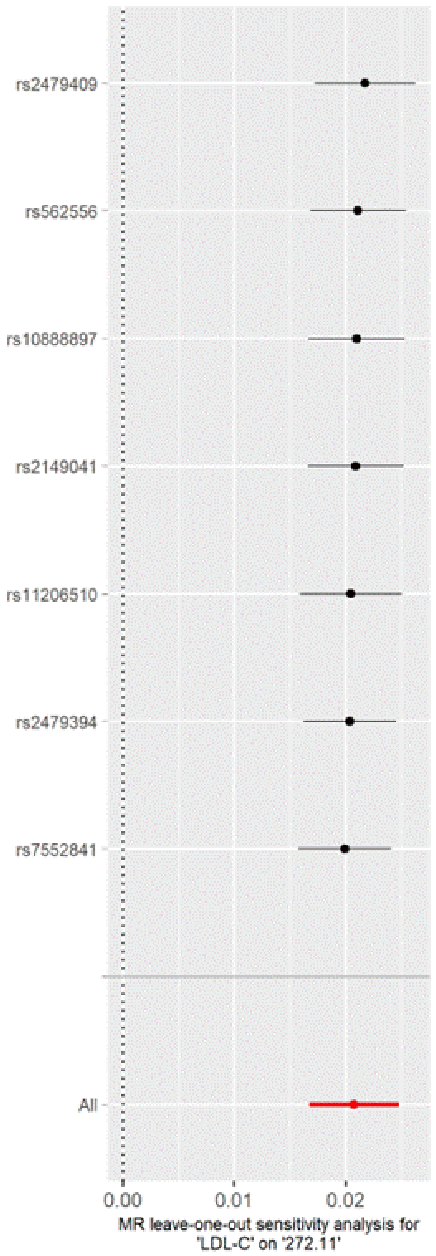

Plot B

Plot A shows the scatter plot of the variant-outcome association estimates for each genetic variant, against the overall estimates for the variant-exposure (i.e. LDL-C lowering) association using different MR methods.

Plot B displays the leave-one-out analysis, which compares the effect estimates when excluding each of the genetic variants in the GRS, one at a time, to the overall estimate including all variants.

Diarrhea (561.1)

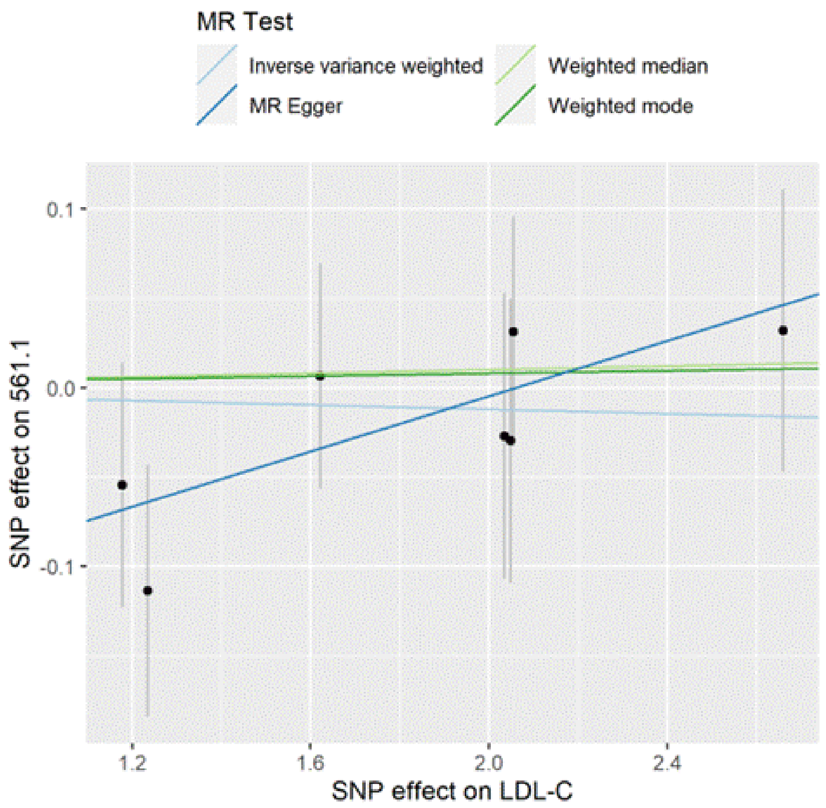

Plot A

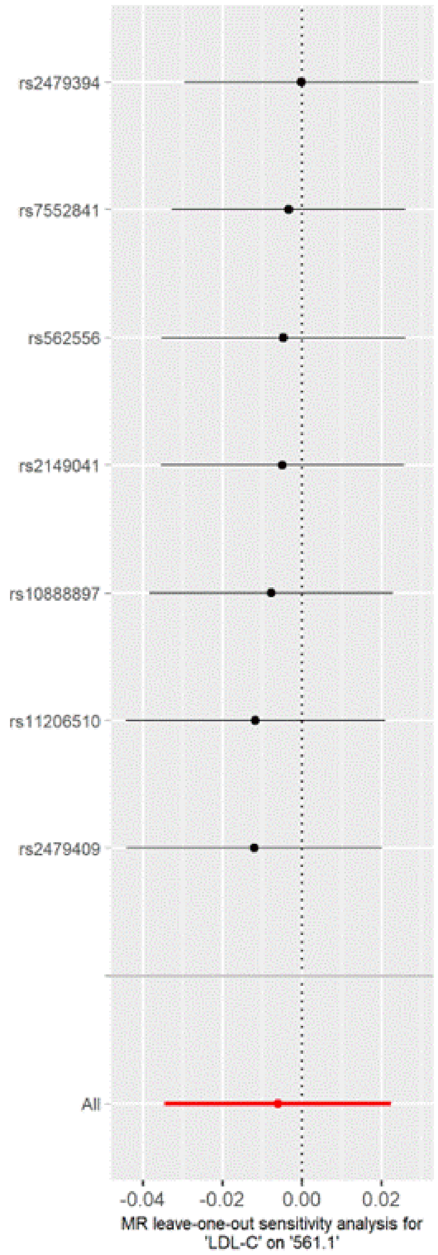

Plot B

Plot A shows the scatter plot of the variant-outcome association estimates for each genetic variant, against the overall estimates for the variant-exposure (i.e. LDL-C lowering) association using different MR methods.

Plot B displays the leave-one-out analysis, which compares the effect estimates when excluding each of the genetic variants in the GRS, one at a time, to the overall estimate including all variants.

Supplementary Figure 2: Plots for the 10 distinct LDL-C-disease associations significant under FDR correction, for the PCSK9 genetic risk score.

Unstable angina (411.1)

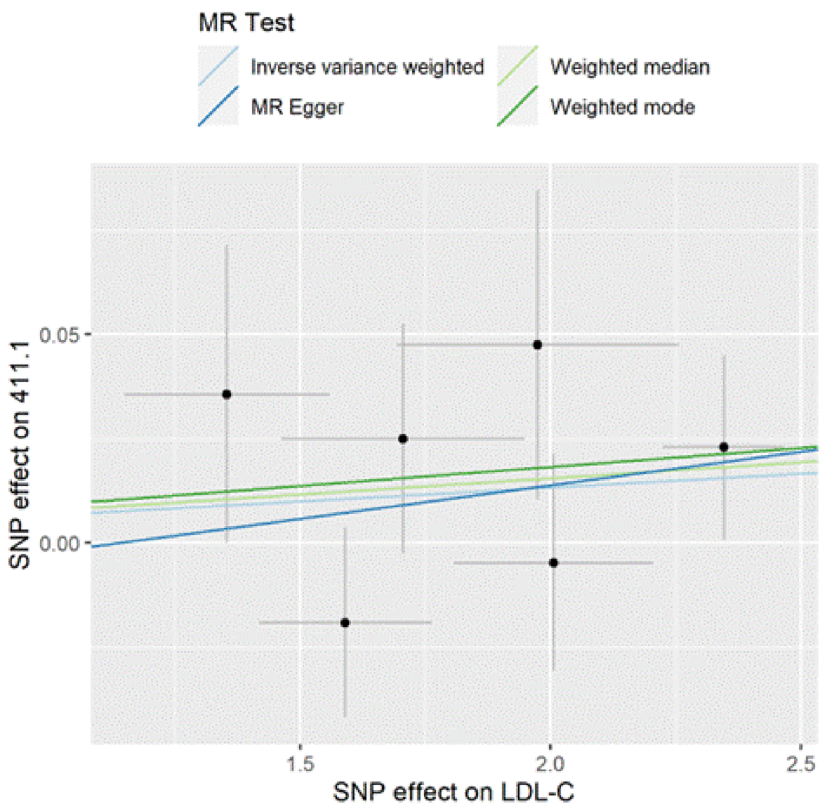

Plot A

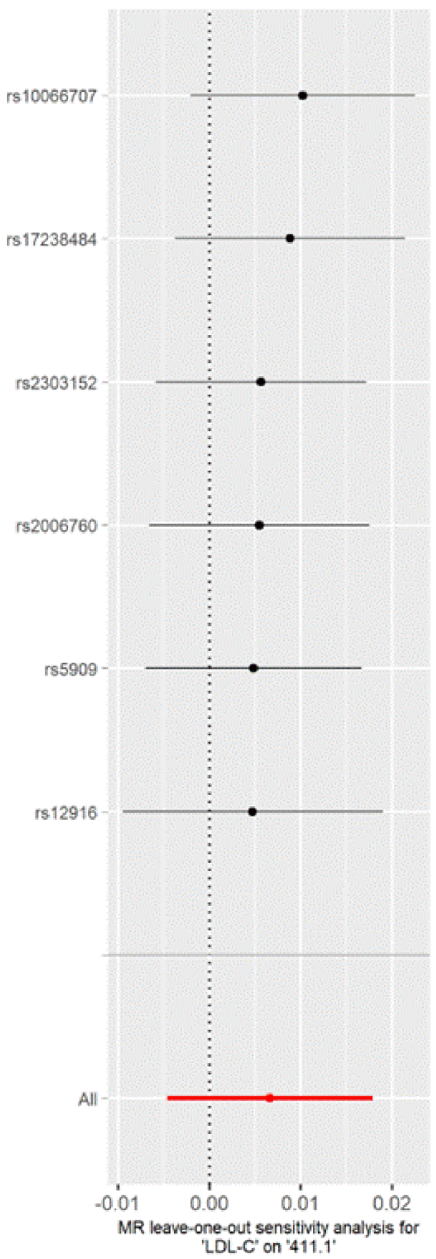

Plot B

Plot A shows the scatter plot of the variant-outcome association estimates for each genetic variant, against the overall estimates for the variant-exposure (i.e. LDL-C lowering) association using different MR methods.

Plot B displays the leave-one-out analysis, which compares the effect estimates when excluding each of the genetic variants in the GRS, one at a time, to the overall estimate including all variants.

Myocardial infarction (411.2)

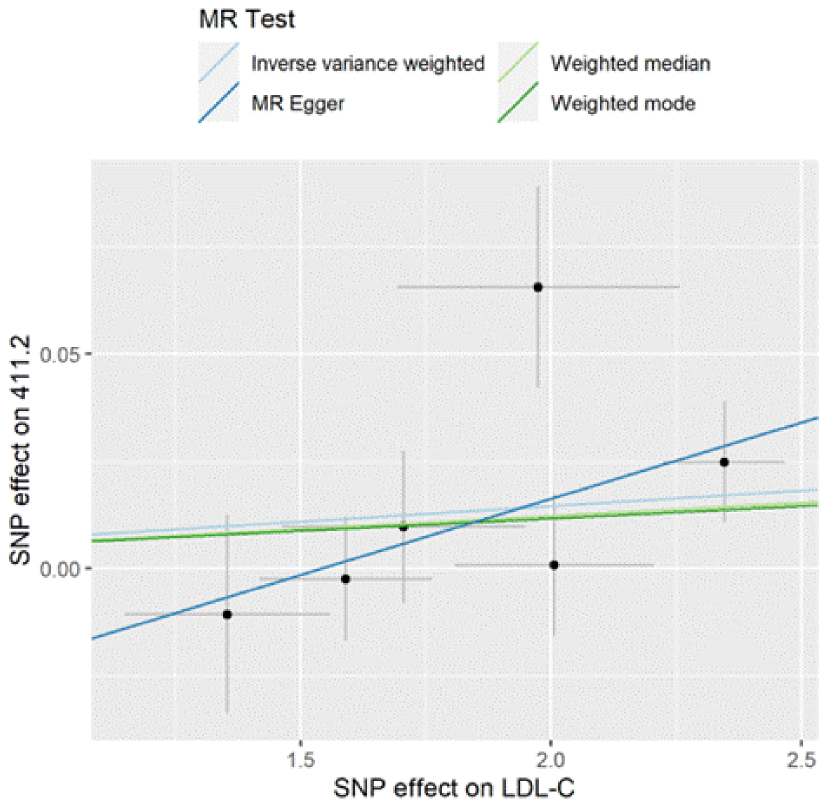

Plot A

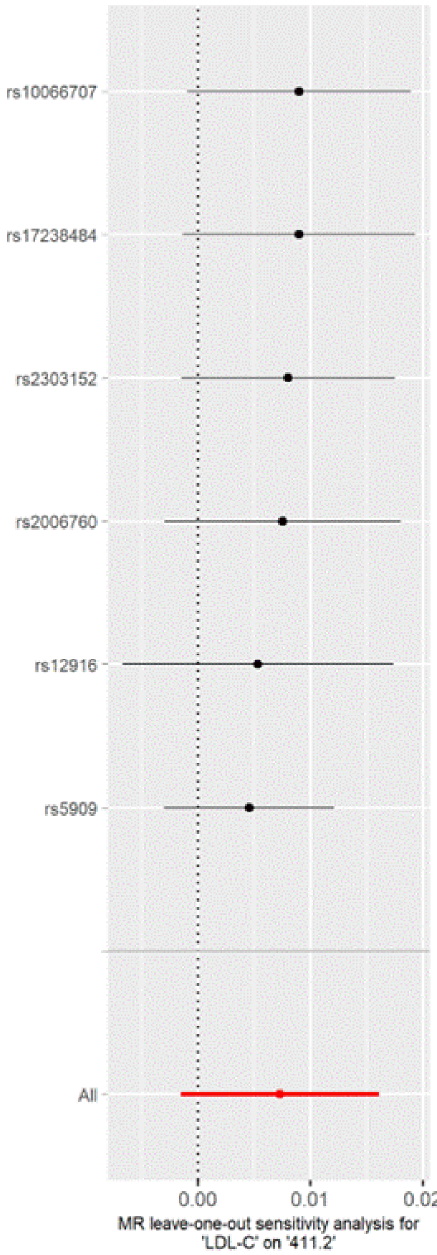

Plot B

Plot A shows the scatter plot of the variant-outcome association estimates for each genetic variant, against the overall estimates for the variant-exposure (i.e. LDL-C lowering) association using different MR methods.

Plot B displays the leave-one-out analysis, which compares the effect estimates when excluding each of the genetic variants in the GRS, one at a time, to the overall estimate including all variants.

Angina pectoris (411.3)

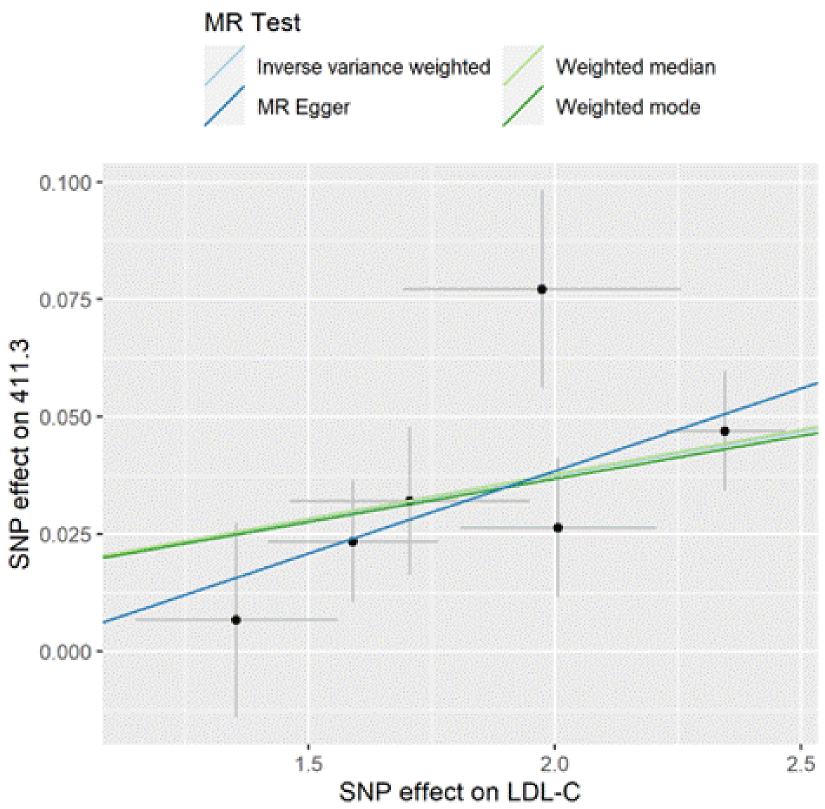

Plot A

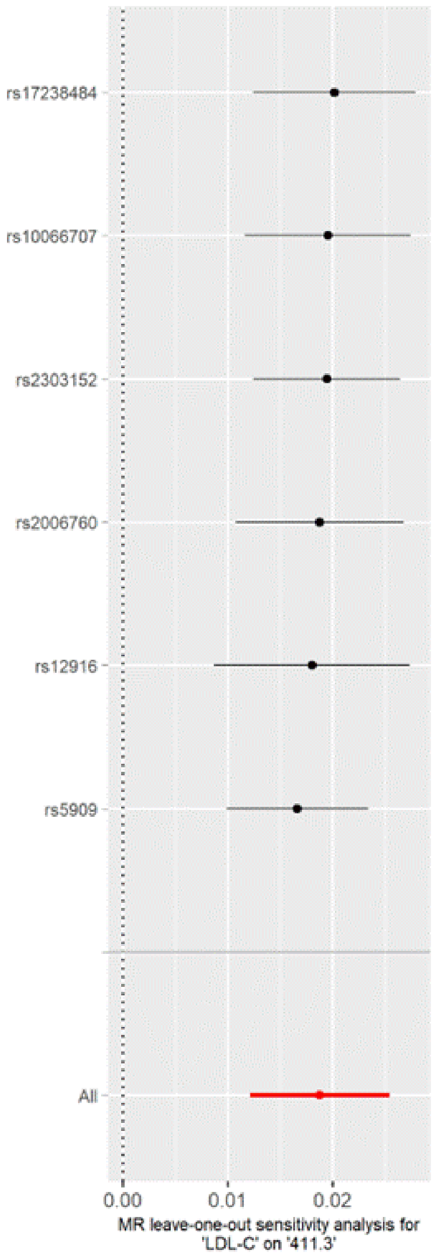

Plot B

Plot A shows the scatter plot of the variant-outcome association estimates for each genetic variant, against the overall estimates for the variant-exposure (i.e. LDL-C lowering) association using different MR methods.

Plot B displays the leave-one-out analysis, which compares the effect estimates when excluding each of the genetic variants in the GRS, one at a time, to the overall estimate including all variants.

Coronary atherosclerosis (411.4)

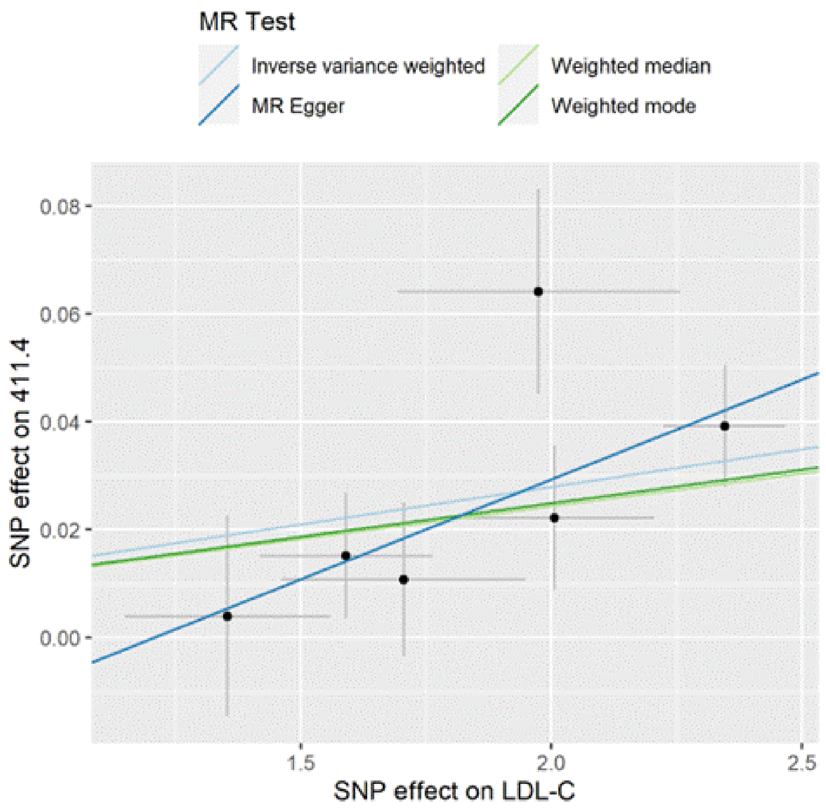

Plot A

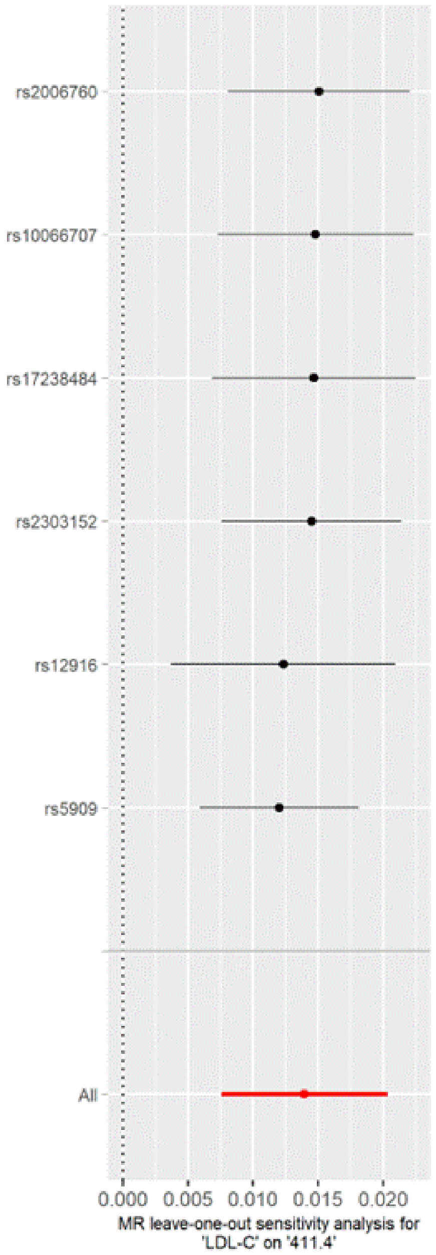

Plot B

Plot A shows the scatter plot of the variant-outcome association estimates for each genetic variant, against the overall estimates for the variant-exposure (i.e. LDL-C lowering) association using different MR methods.

Plot B displays the leave-one-out analysis, which compares the effect estimates when excluding each of the genetic variants in the GRS, one at a time, to the overall estimate including all variants.

Other chronic ischemic heart diseases (411.8)

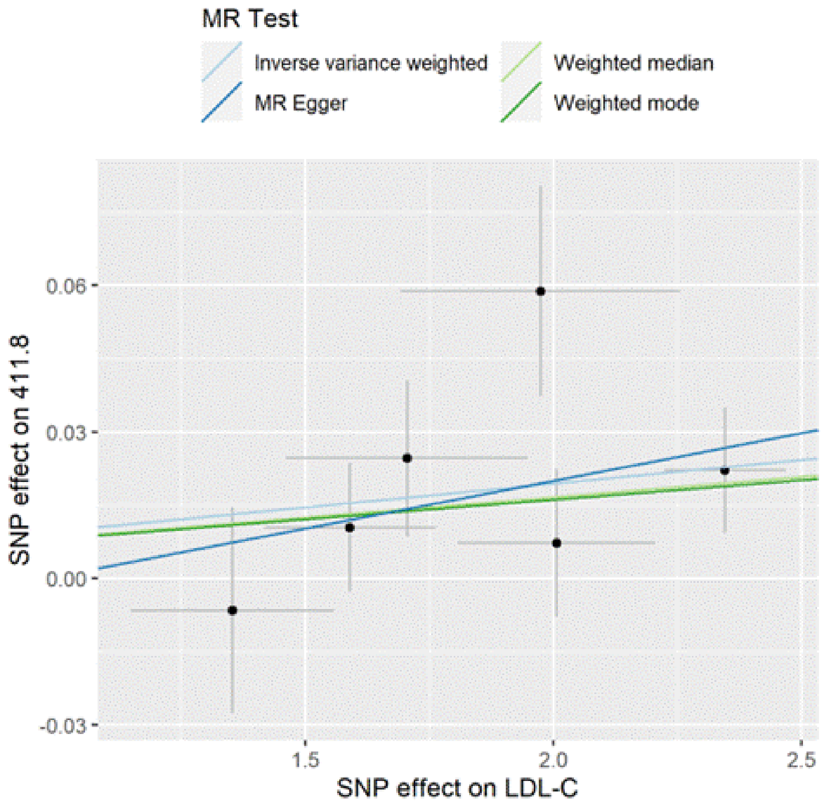

Plot A

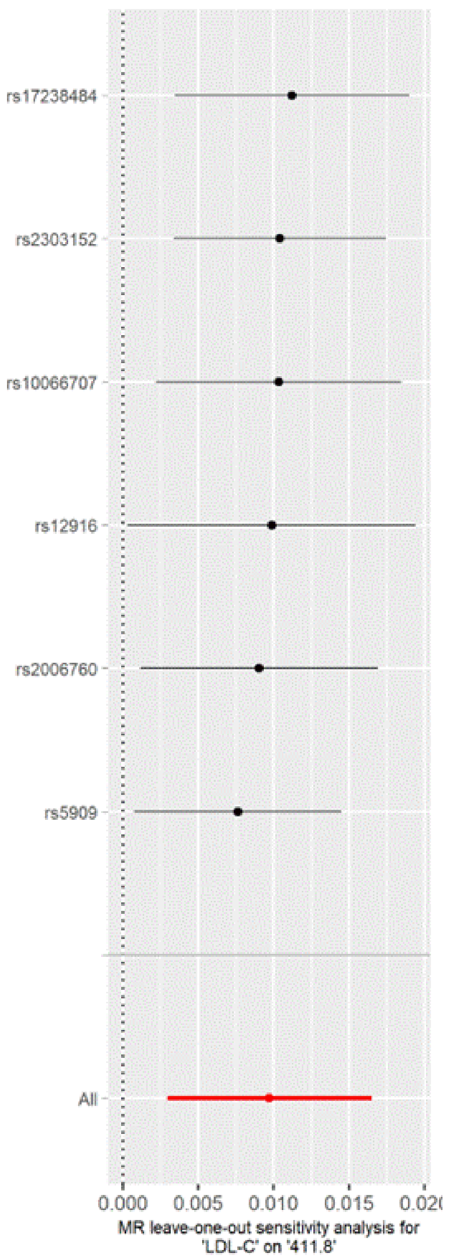

Plot B

Plot A shows the scatter plot of the variant-outcome association estimates for each genetic variant, against the overall estimates for the variant-exposure (i.e. LDL-C lowering) association using different MR methods.

Plot B displays the leave-one-out analysis, which compares the effect estimates when excluding each of the genetic variants in the GRS, one at a time, to the overall estimate including all variants.

Hypertensive heart and/or renal disease (401.2)

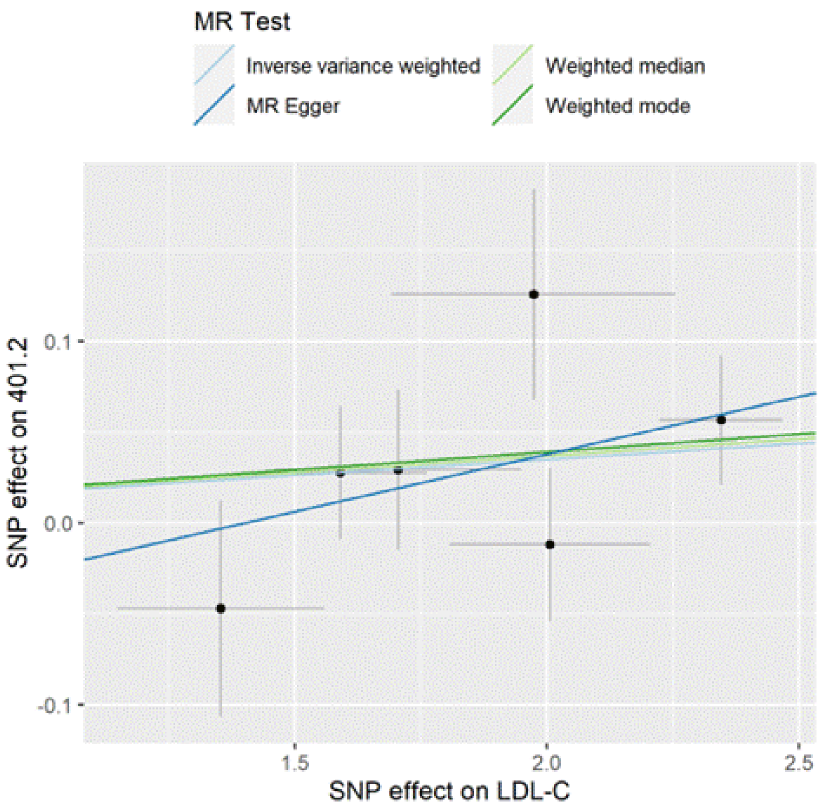

Plot A

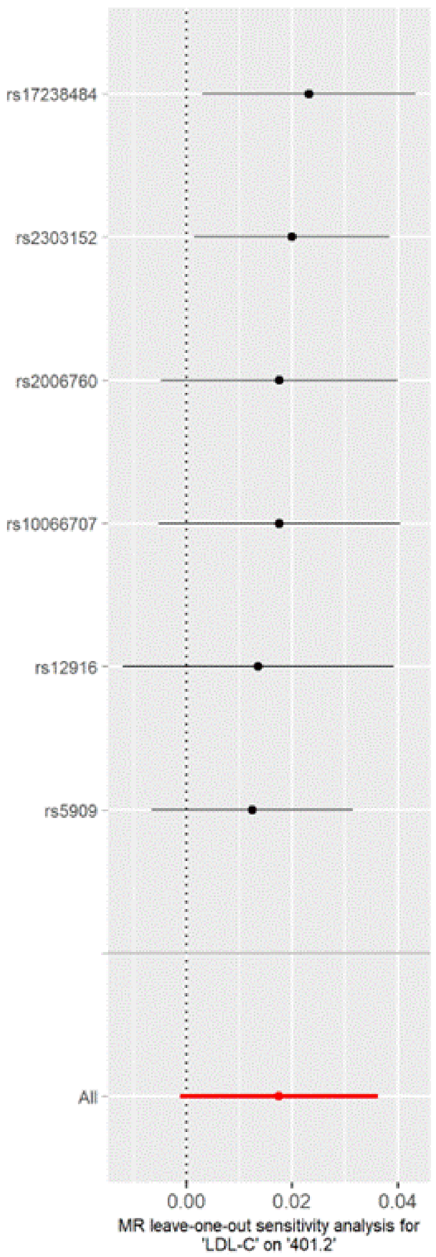

Plot B

Plot A shows the scatter plot of the variant-outcome association estimates for each genetic variant, against the overall estimates for the variant-exposure (i.e. LDL-C lowering) association using different MR methods.

Plot B displays the leave-one-out analysis, which compares the effect estimates when excluding each of the genetic variants in the GRS, one at a time, to the overall estimate including all variants.

Abdominal aortic aneurysm (442.11)

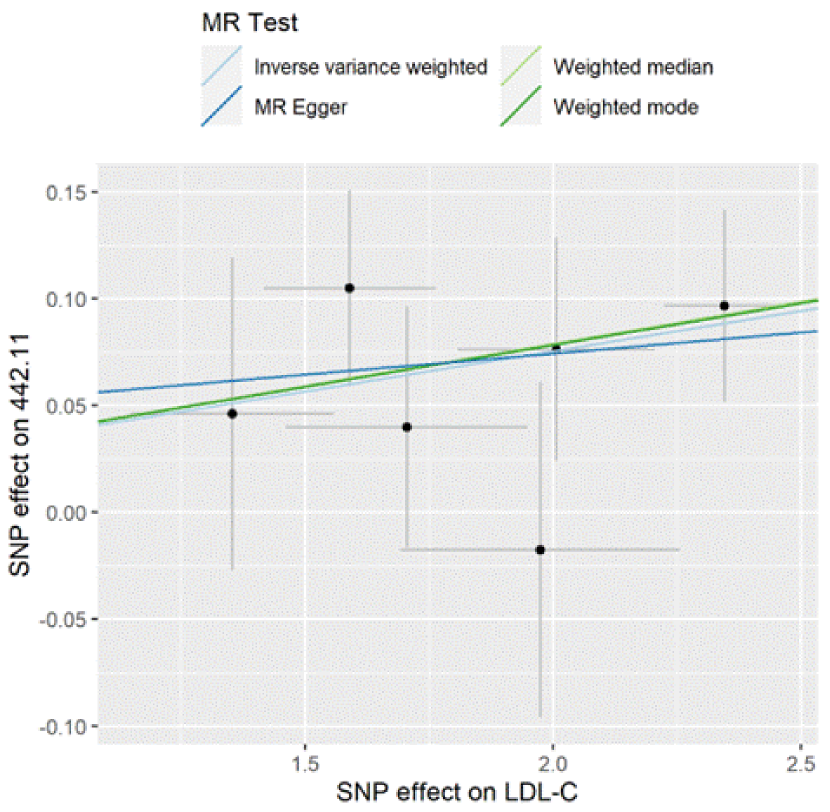

Plot A

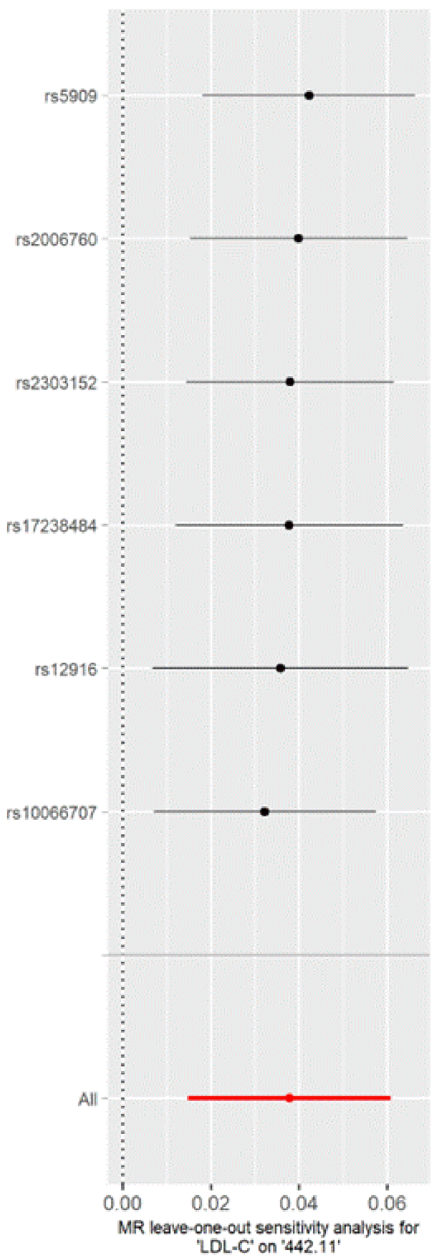

Plot B

Plot A shows the scatter plot of the variant-outcome association estimates for each genetic variant, against the overall estimates for the variant-exposure (i.e. LDL-C lowering) association using different MR methods.

Plot B displays the leave-one-out analysis, which compares the effect estimates when excluding each of the genetic variants in the GRS, one at a time, to the overall estimate including all variants.

Peripheral vascular disease (443)

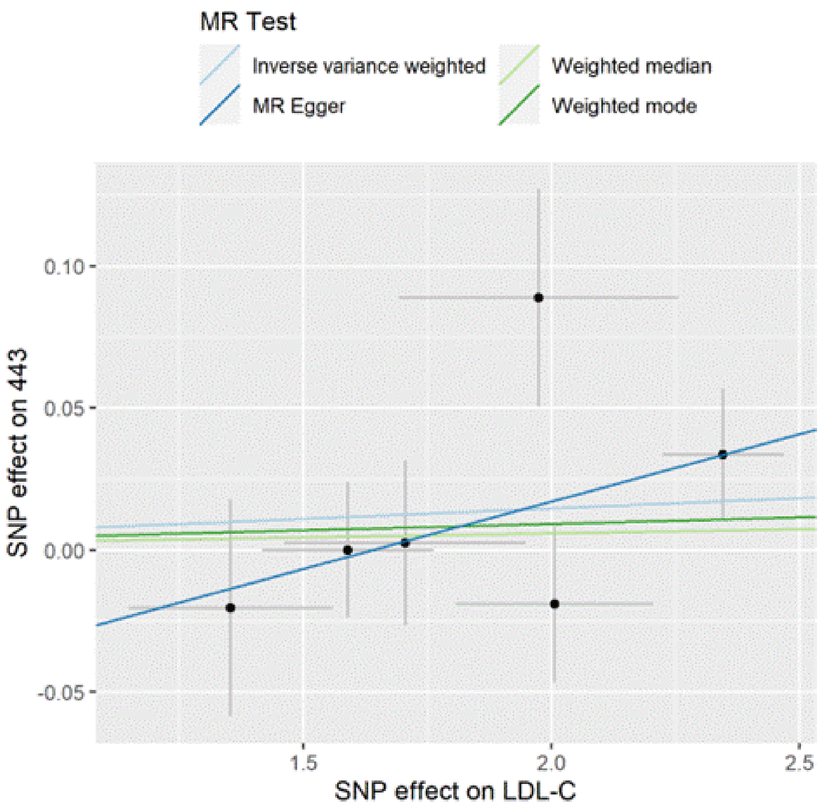

Plot A

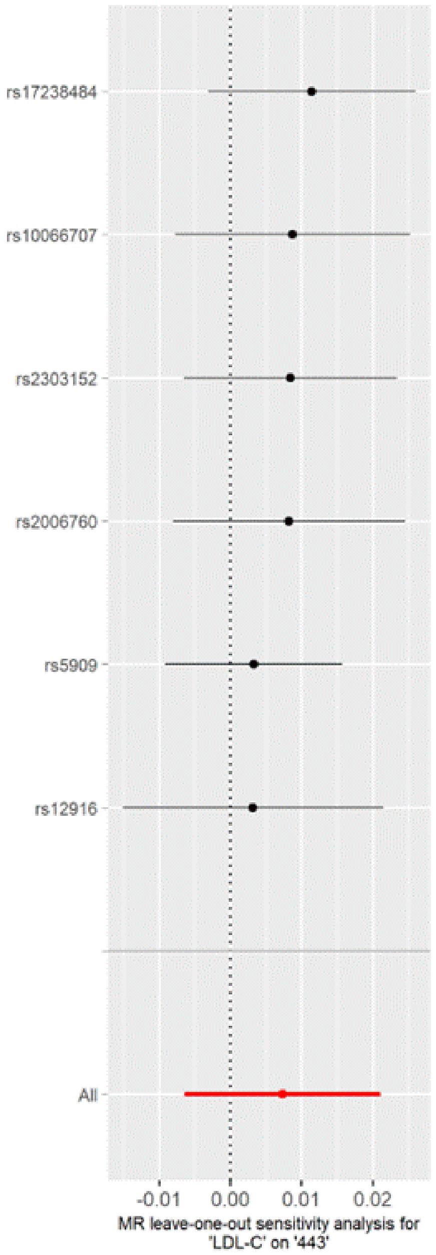

Plot B

Plot A shows the scatter plot of the variant-outcome association estimates for each genetic variant, against the overall estimates for the variant-exposure (i.e. LDL-C lowering) association using different MR methods.

Plot B displays the leave-one-out analysis, which compares the effect estimates when excluding each of the genetic variants in the GRS, one at a time, to the overall estimate including all variants.

Hypercholesterolemia (272.11)

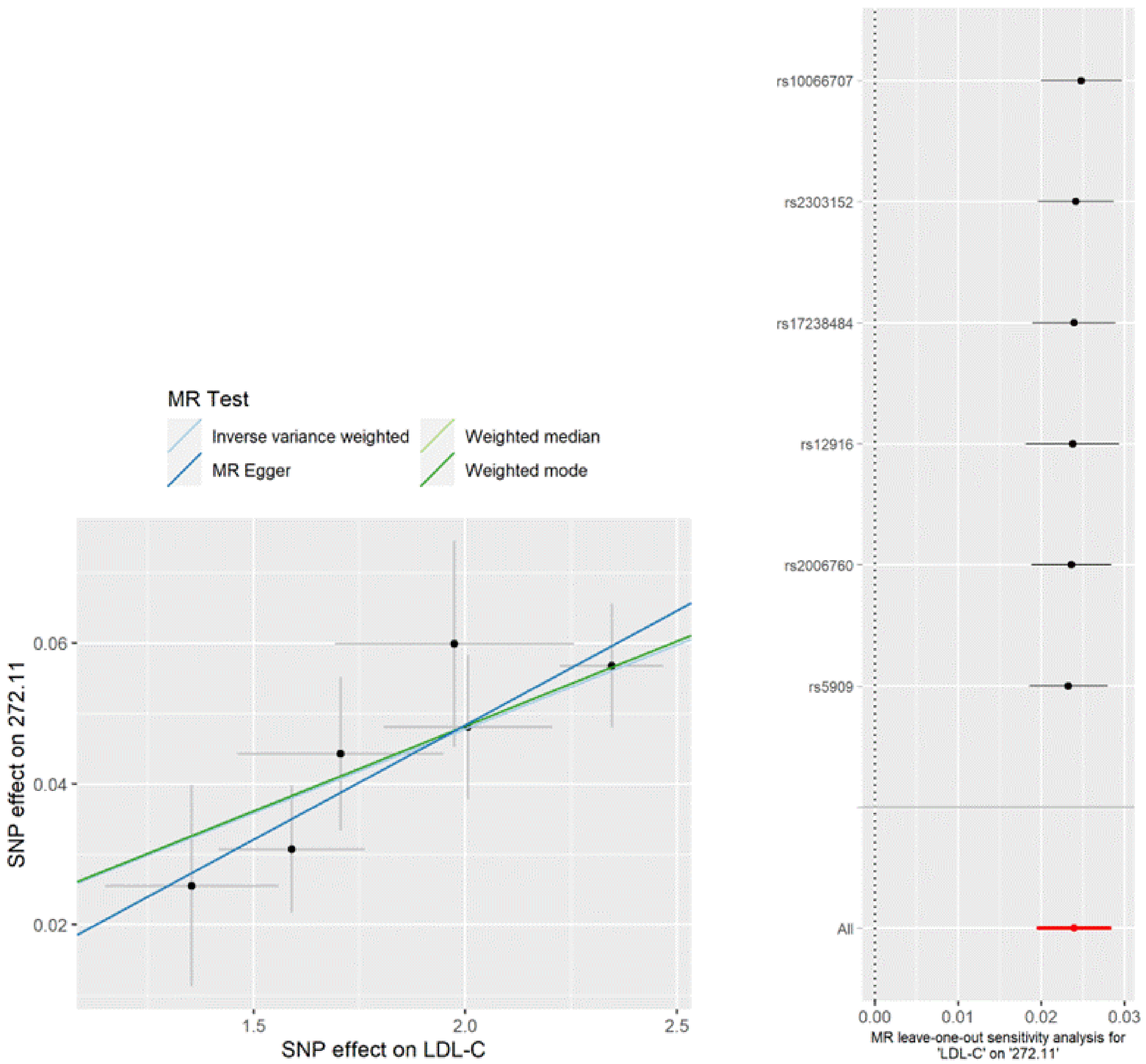

Plot A **Plot B**

Plot A shows the scatter plot of the variant-outcome association estimates for each genetic variant, against the overall estimates for the variant-exposure (i.e. LDL-C lowering) association using different MR methods.

Plot B displays the leave-one-out analysis, which compares the effect estimates when excluding each of the genetic variants in the GRS, one at a time, to the overall estimate including all variants.

Diarrhea (561.1)

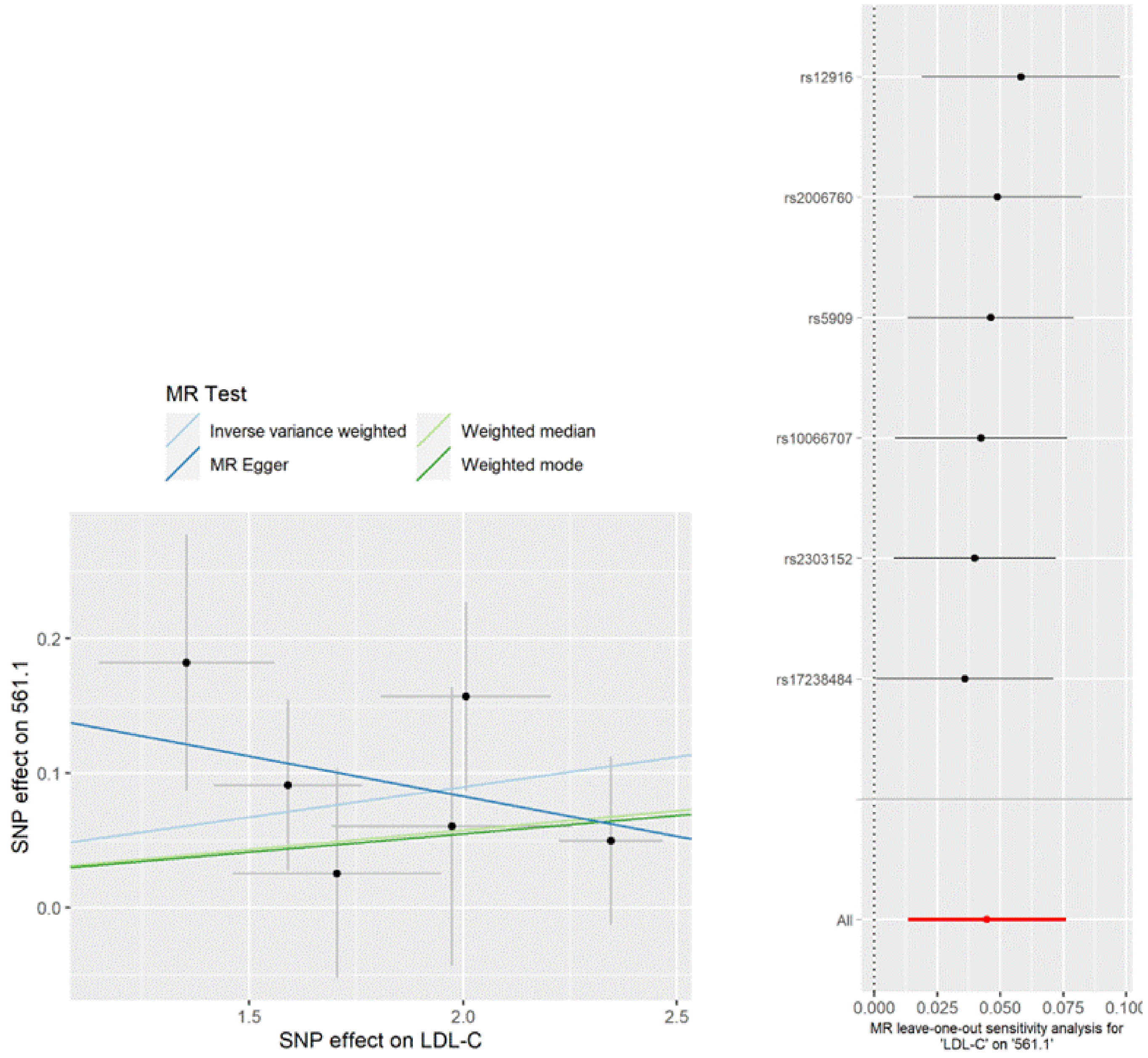

Plot A **Plot B**

Plot A shows the scatter plot of the variant-outcome association estimates for each genetic variant, against the overall estimates for the variant-exposure (i.e. LDL-C lowering) association using different MR methods.

Plot B displays the leave-one-out analysis, which compares the effect estimates when excluding each of the genetic variants in the GRS, one at a time, to the overall estimate including all variants.

Supplementary Figure 3: Plots for the 10 distinct LDL-C-disease associations significant under FDR correction, for the HMGCR genetic risk score.

Unstable angina (411.1)

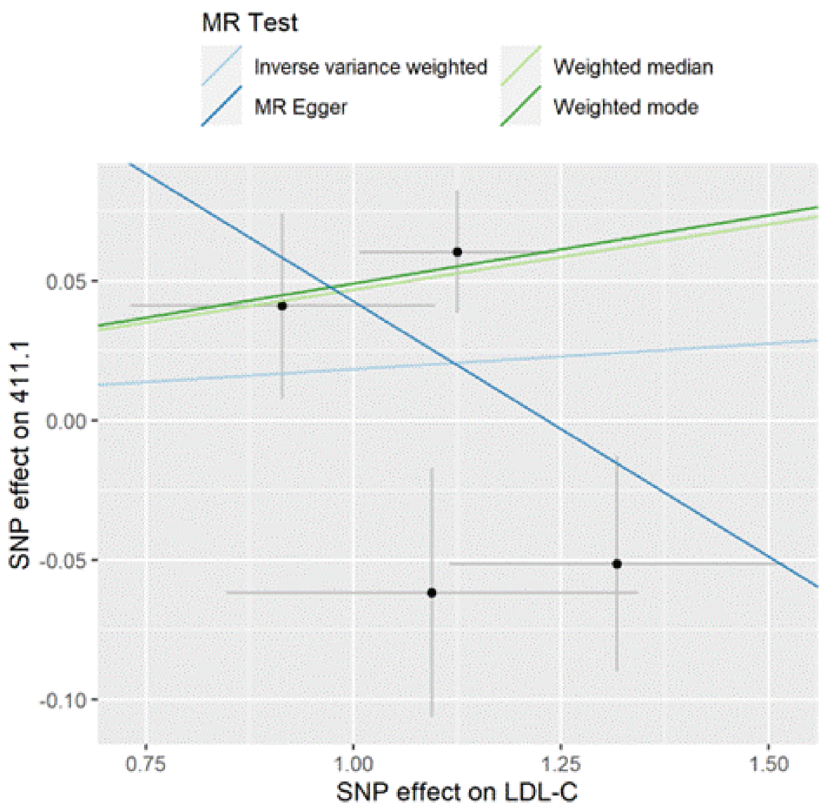

Plot A

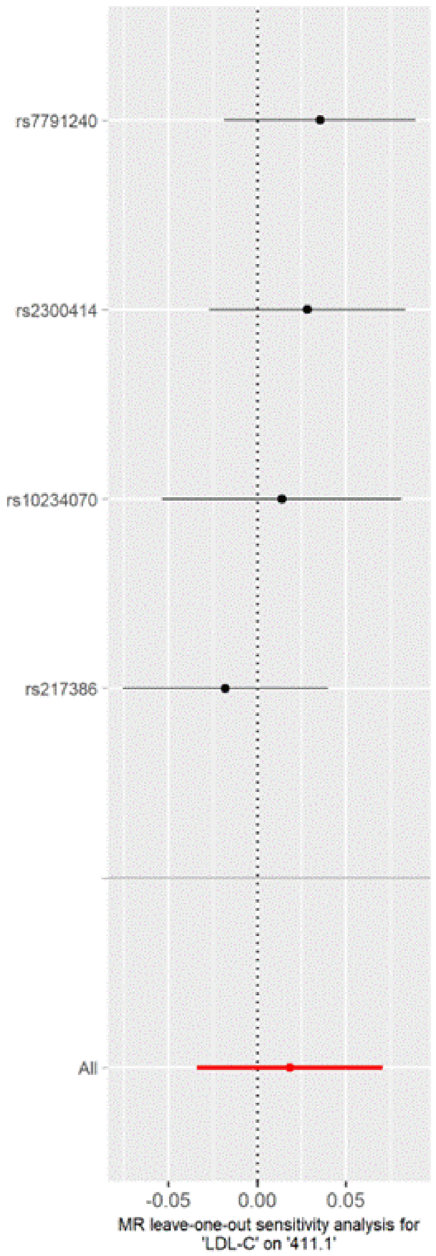

Plot B

Plot A shows the scatter plot of the variant-outcome association estimates for each genetic variant, against the overall estimates for the variant-exposure (i.e. LDL-C lowering) association using different MR methods.

Plot B displays the leave-one-out analysis, which compares the effect estimates when excluding each of the genetic variants in the GRS, one at a time, to the overall estimate including all variants.

Myocardial infarction (411.2)

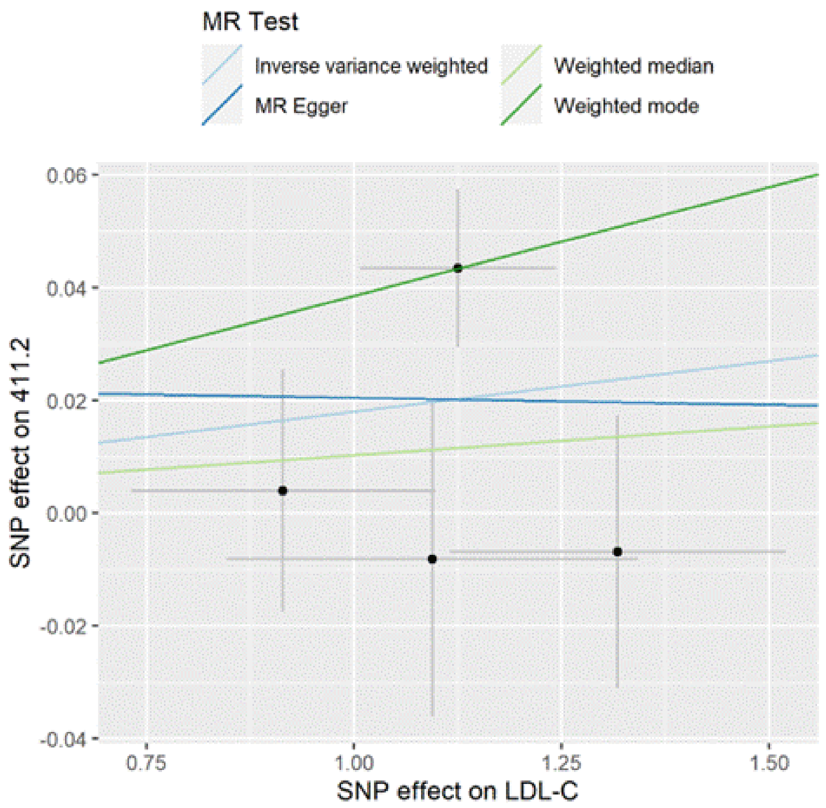

Plot A

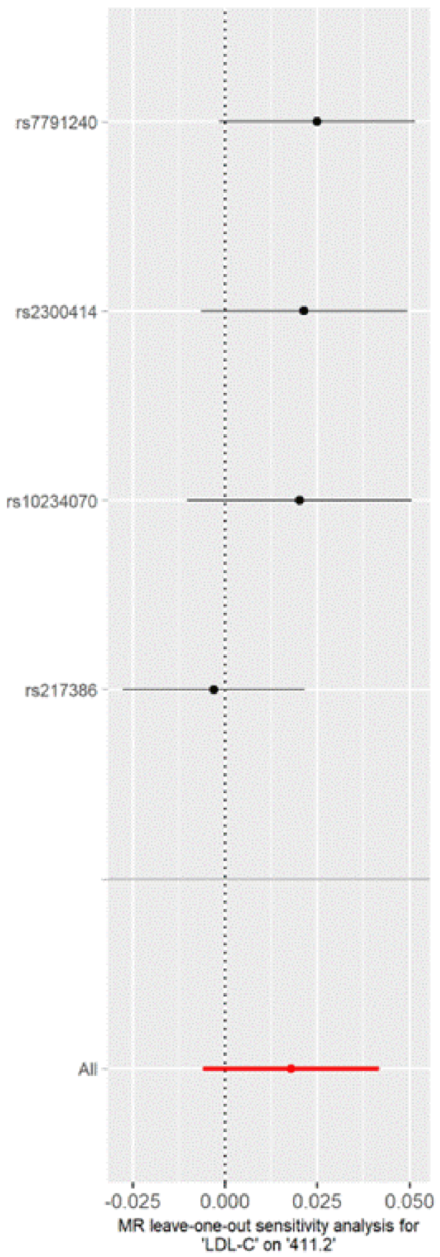

Plot B

Plot A shows the scatter plot of the variant-outcome association estimates for each genetic variant, against the overall estimates for the variant-exposure (i.e. LDL-C lowering) association using different MR methods.

Plot B displays the leave-one-out analysis, which compares the effect estimates when excluding each of the genetic variants in the GRS, one at a time, to the overall estimate including all variants.

Angina pectoris (411.3)

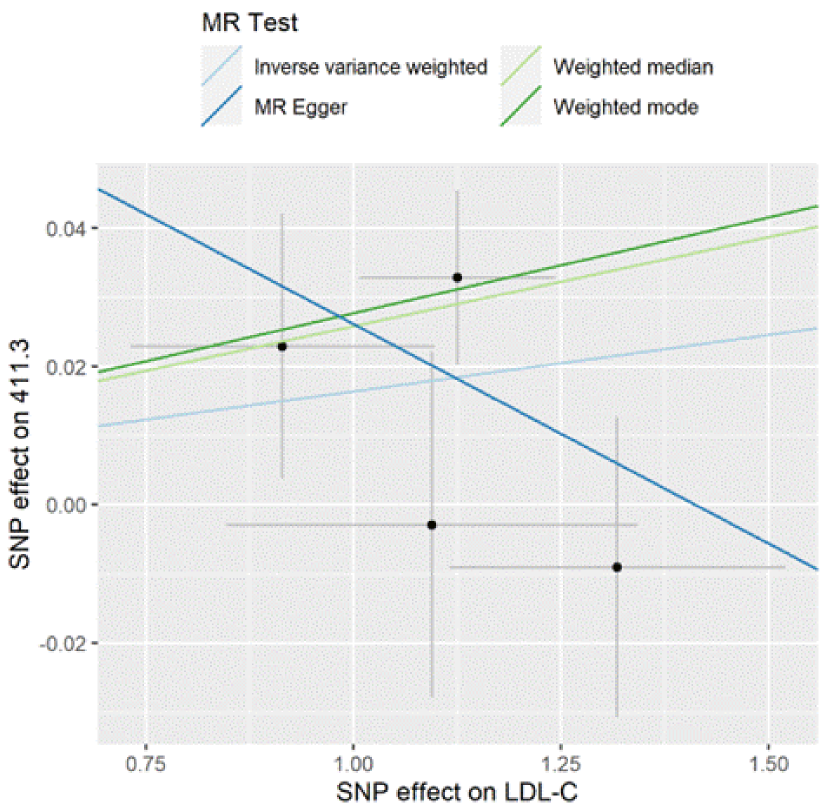

Plot A

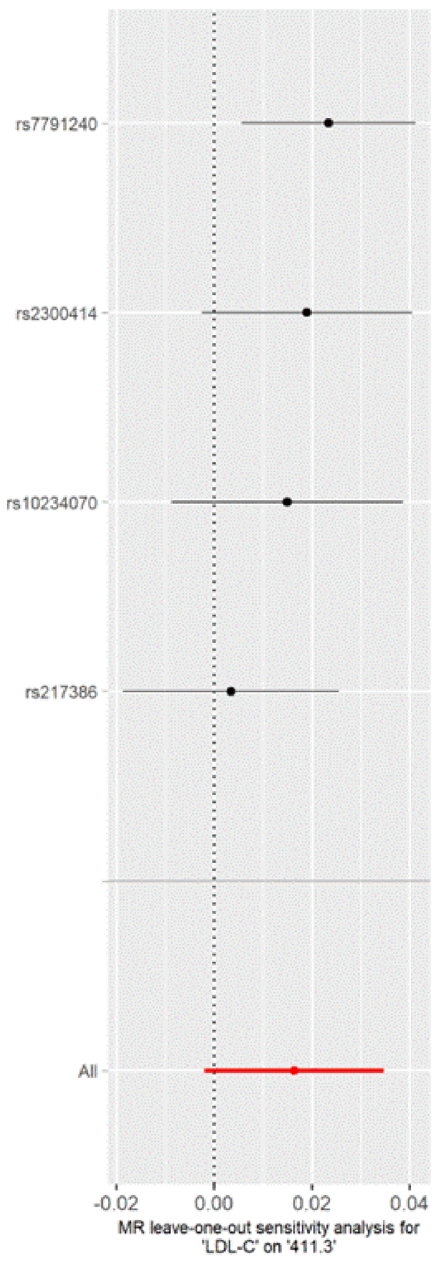

Plot B

Plot A shows the scatter plot of the variant-outcome association estimates for each genetic variant, against the overall estimates for the variant-exposure (i.e. LDL-C lowering) association using different MR methods.

Plot B displays the leave-one-out analysis, which compares the effect estimates when excluding each of the genetic variants in the GRS, one at a time, to the overall estimate including all variants.

Coronary atherosclerosis (411.4)

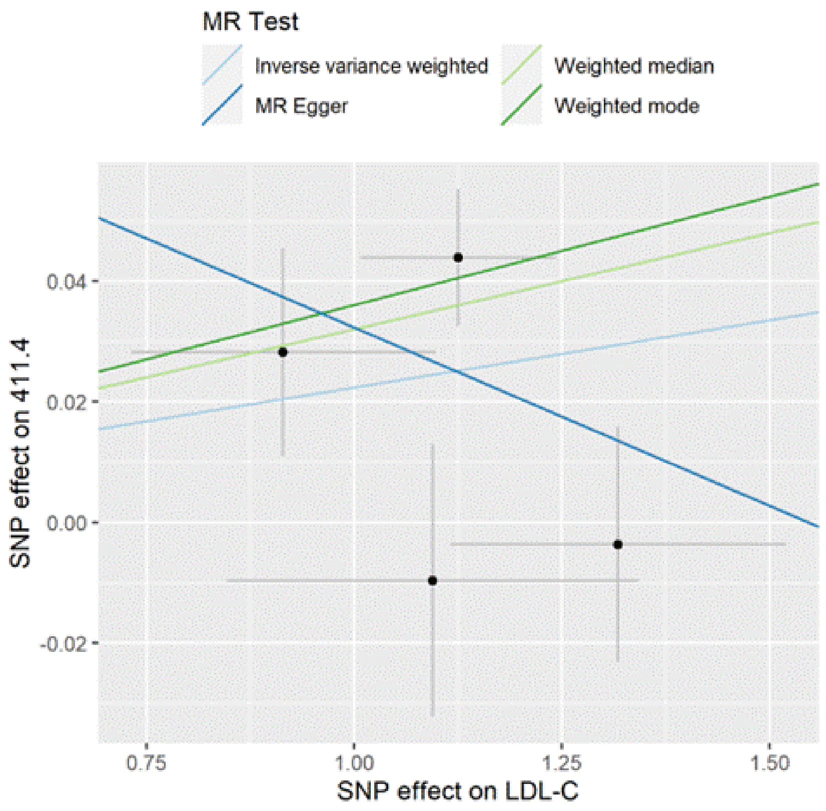

Plot A

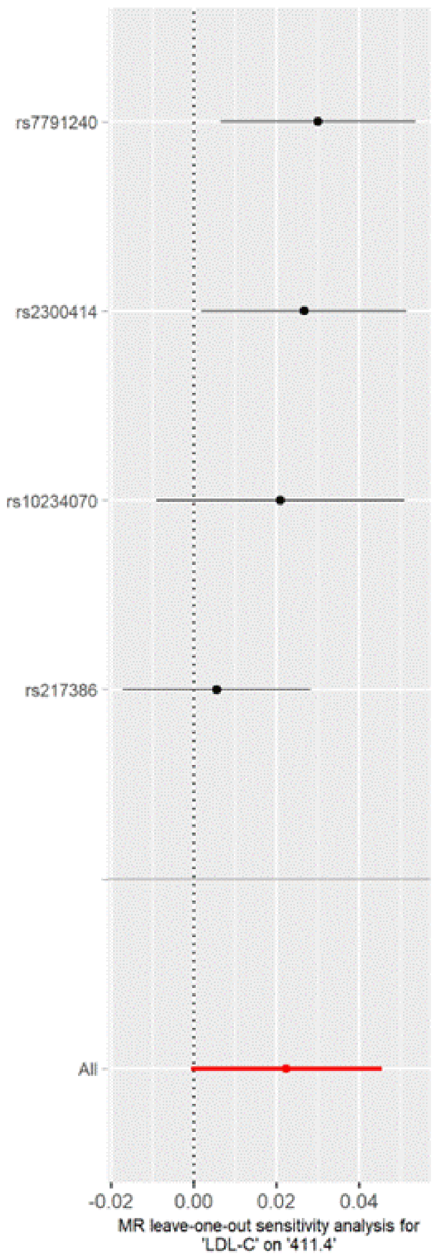

Plot B

Plot A shows the scatter plot of the variant-outcome association estimates for each genetic variant, against the overall estimates for the variant-exposure (i.e. LDL-C lowering) association using different MR methods.

Plot B displays the leave-one-out analysis, which compares the effect estimates when excluding each of the genetic variants in the GRS, one at a time, to the overall estimate including all variants.

Other chronic ischemic heart diseases (411.8)

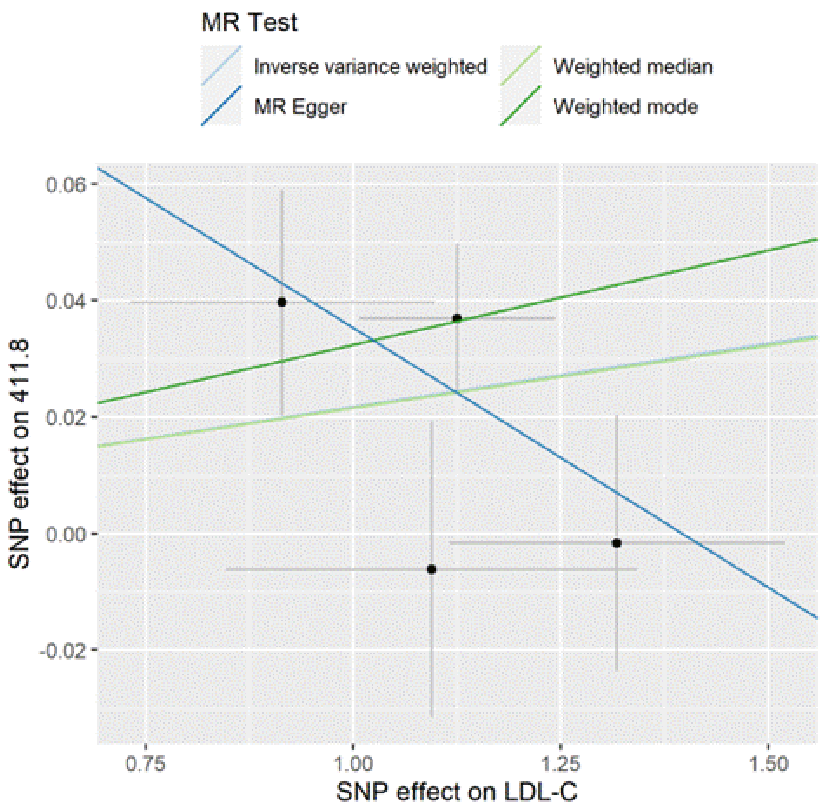

Plot A

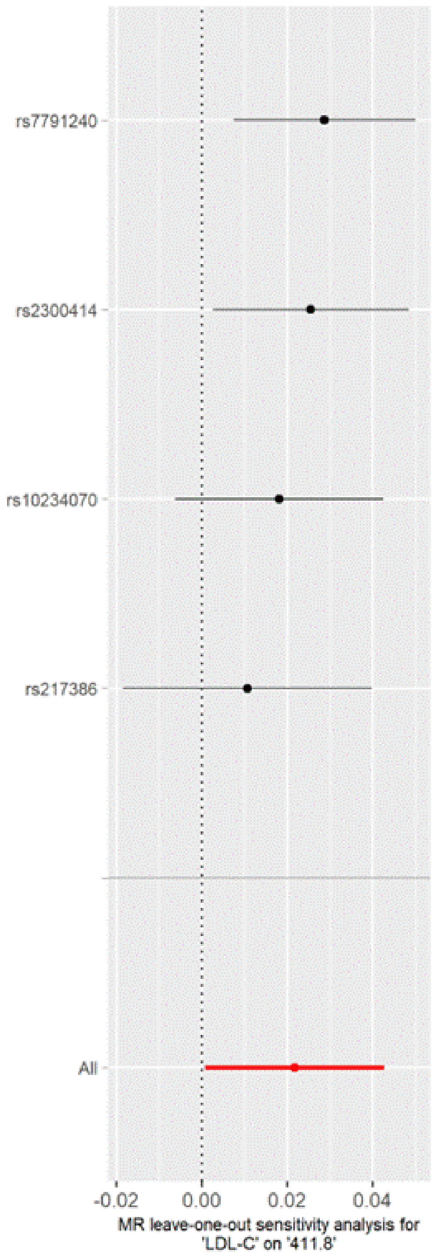

Plot B

Plot A shows the scatter plot of the variant-outcome association estimates for each genetic variant, against the overall estimates for the variant-exposure (i.e. LDL-C lowering) association using different MR methods.

Plot B displays the leave-one-out analysis, which compares the effect estimates when excluding each of the genetic variants in the GRS, one at a time, to the overall estimate including all variants.

Hypertensive heart and/or renal disease (401.2)

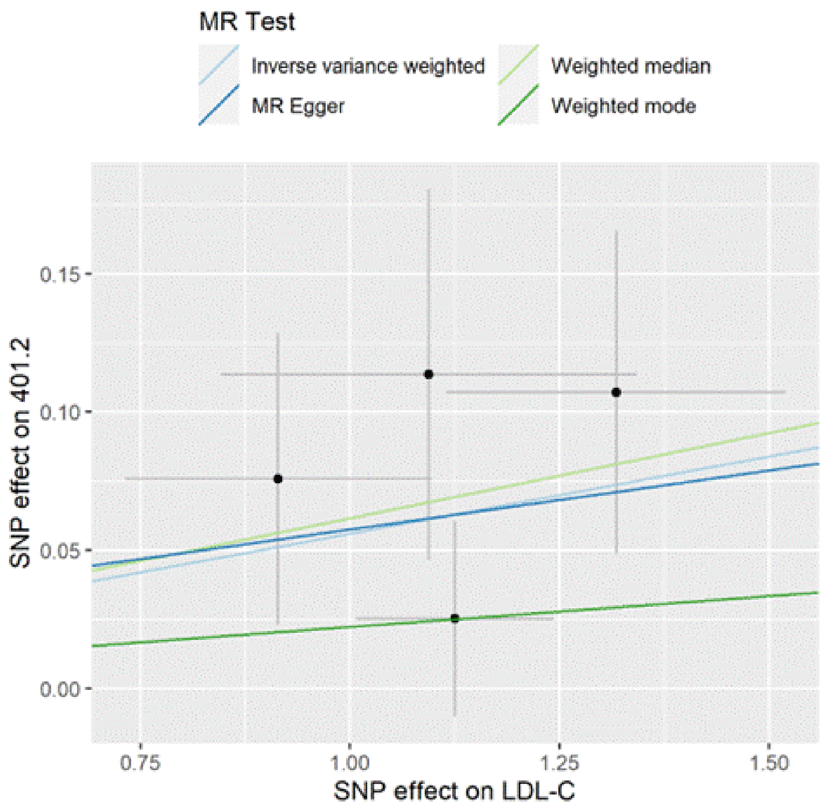

Plot A

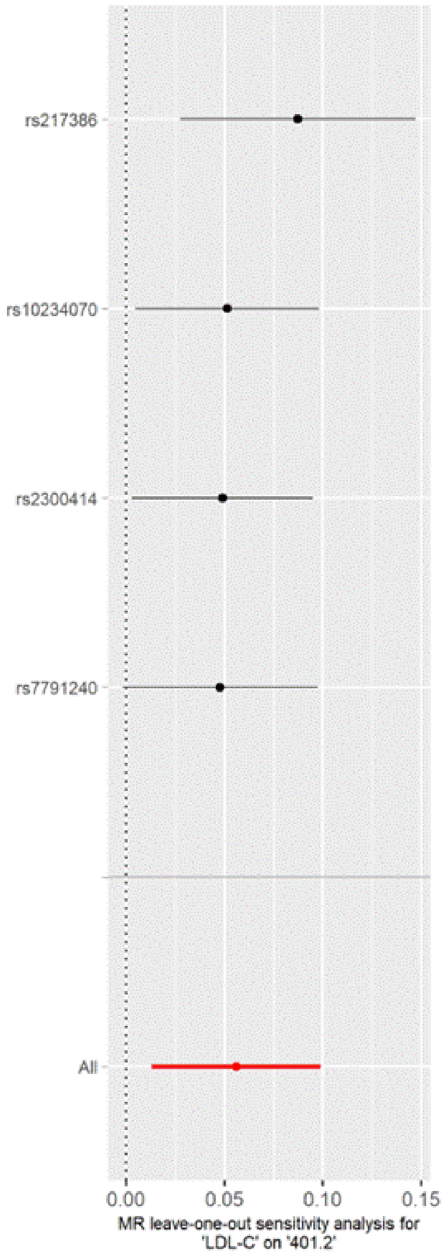

Plot B

Plot A shows the scatter plot of the variant-outcome association estimates for each genetic variant, against the overall estimates for the variant-exposure (i.e. LDL-C lowering) association using different MR methods.

Plot B displays the leave-one-out analysis, which compares the effect estimates when excluding each of the genetic variants in the GRS, one at a time, to the overall estimate including all variants.

Abdominal aortic aneurysm (442.11)

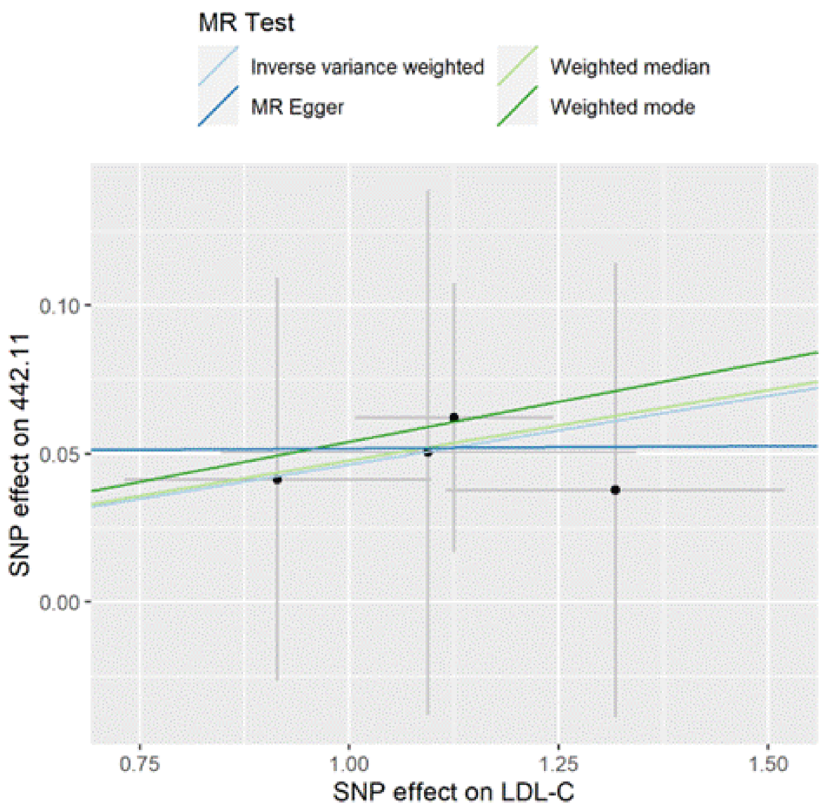

Plot A

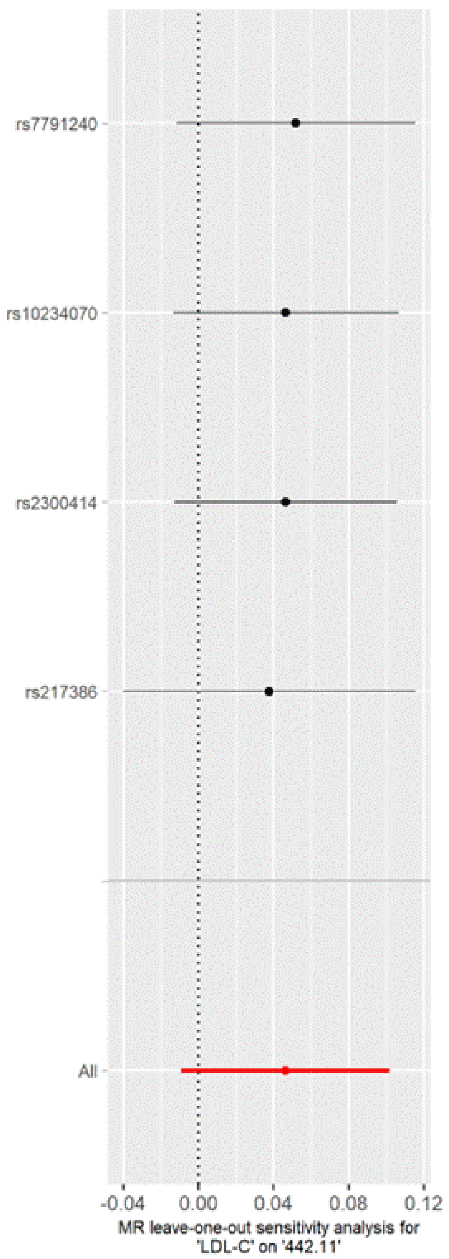

Plot B

Plot A shows the scatter plot of the variant-outcome association estimates for each genetic variant, against the overall estimates for the variant-exposure (i.e. LDL-C lowering) association using different MR methods.

Plot B displays the leave-one-out analysis, which compares the effect estimates when excluding each of the genetic variants in the GRS, one at a time, to the overall estimate including all variants.

Peripheral vascular disease (443)

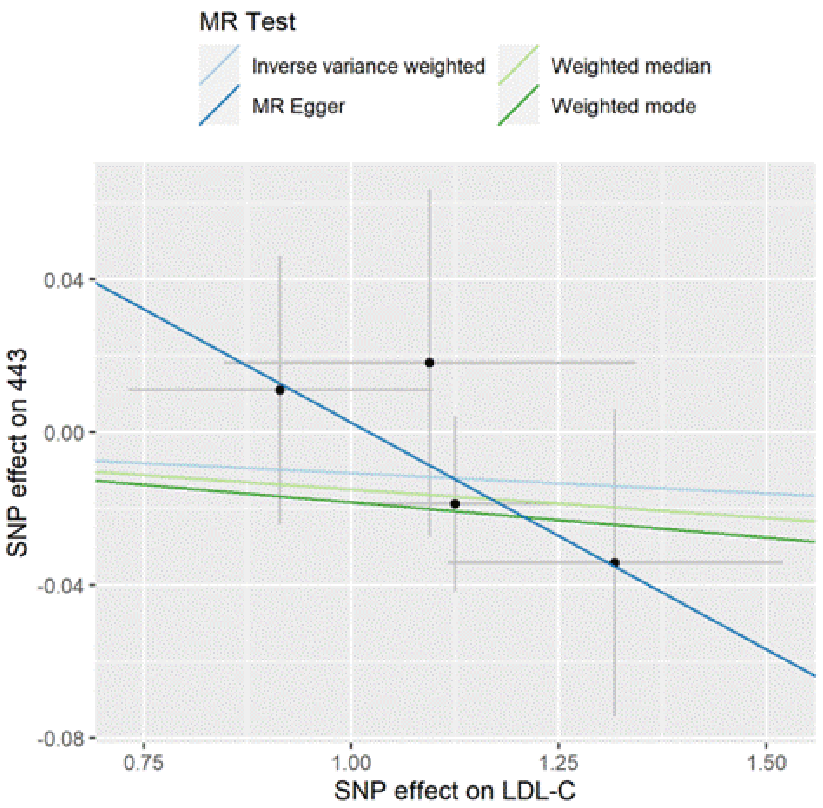

Plot A

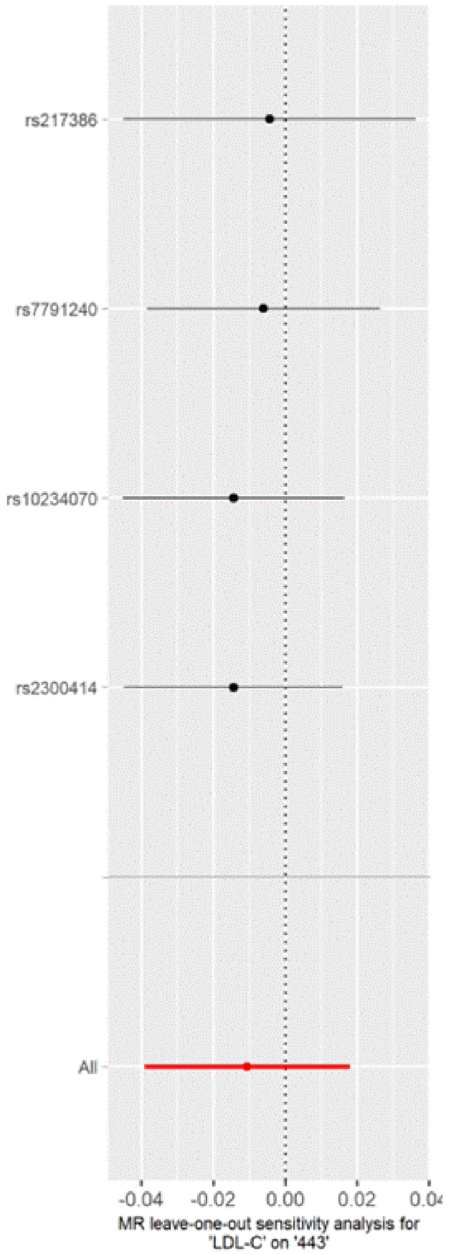

Plot B

Plot A shows the scatter plot of the variant-outcome association estimates for each genetic variant, against the overall estimates for the variant-exposure (i.e. LDL-C lowering) association using different MR methods.

Plot B displays the leave-one-out analysis, which compares the effect estimates when excluding each of the genetic variants in the GRS, one at a time, to the overall estimate including all variants.

Hypercholesterolemia (272.11)

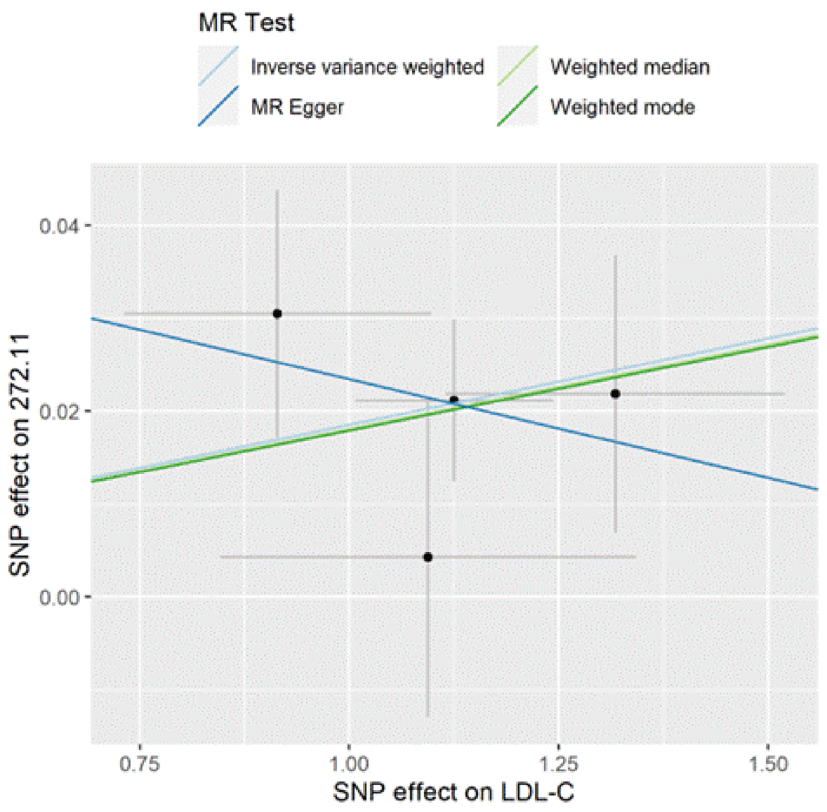

Plot A

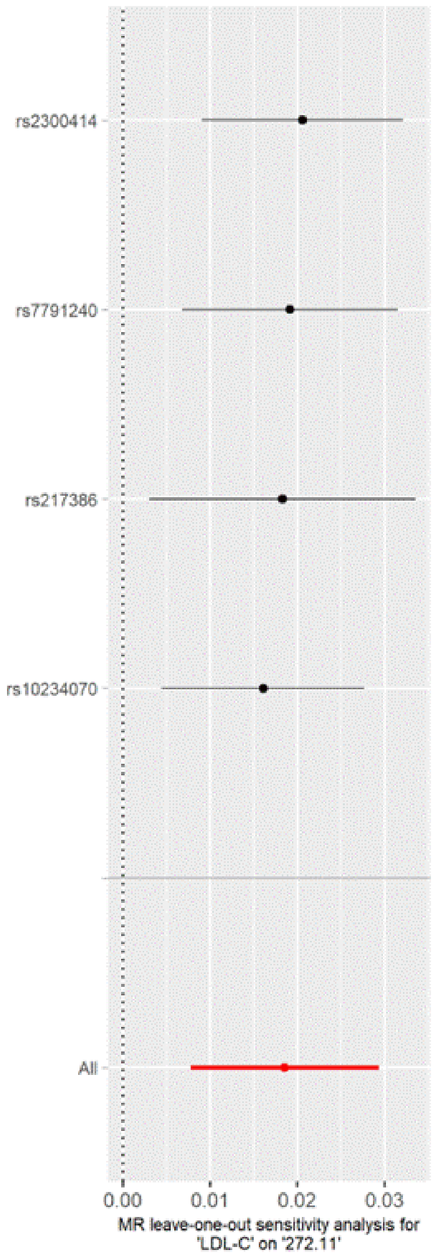

Plot B

Plot A shows the scatter plot of the variant-outcome association estimates for each genetic variant, against the overall estimates for the variant-exposure (i.e. LDL-C lowering) association using different MR methods.

Plot B displays the leave-one-out analysis, which compares the effect estimates when excluding each of the genetic variants in the GRS, one at a time, to the overall estimate including all variants.

Diarrhea (561.1)

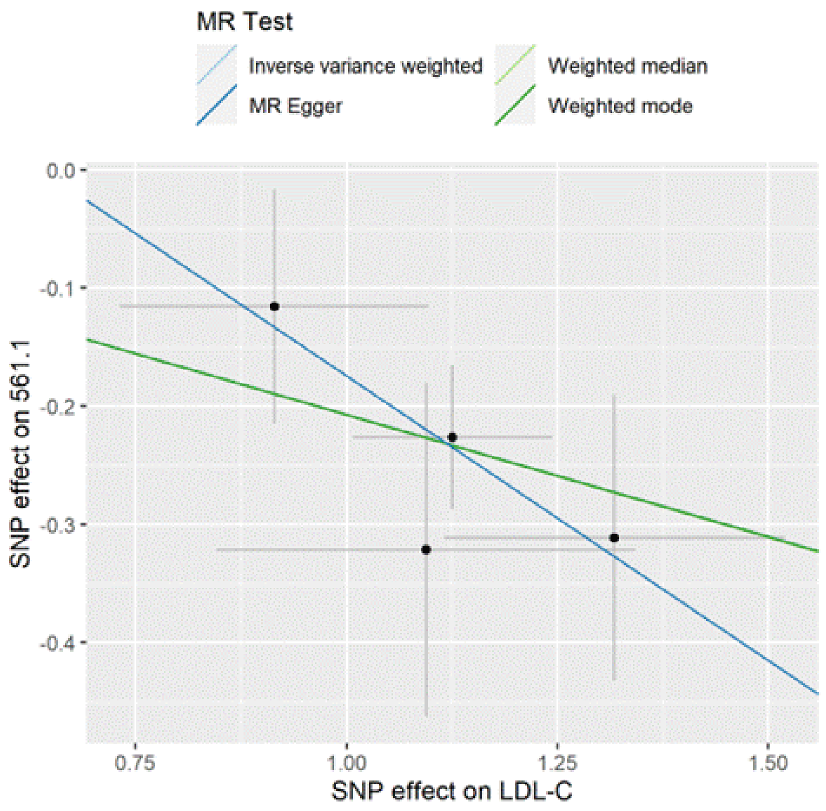

Plot A

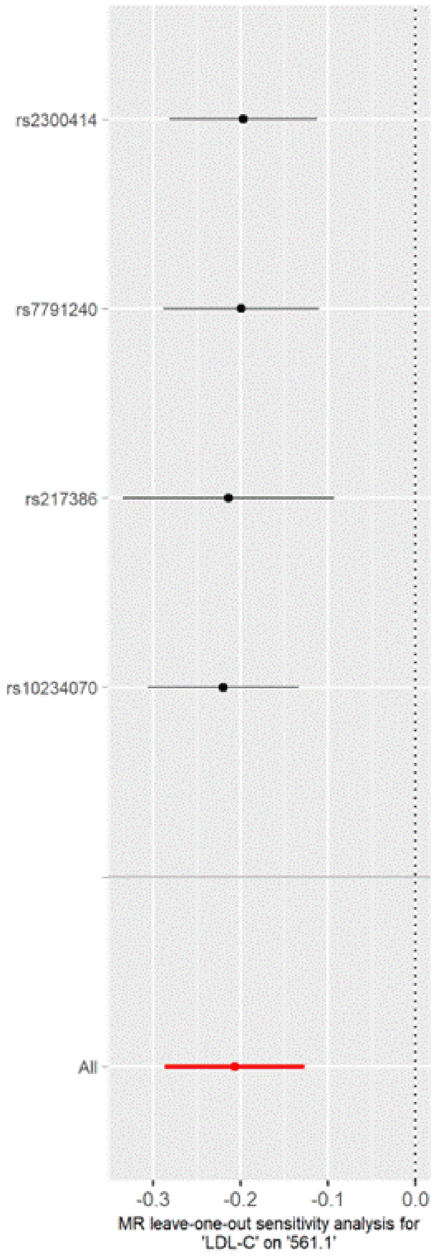

Plot B

Plot A shows the scatter plot of the variant-outcome association estimates for each genetic variant, against the overall estimates for the variant-exposure (i.e. LDL-C lowering) association using different MR methods.

Plot B displays the leave-one-out analysis, which compares the effect estimates when excluding each of the genetic variants in the GRS, one at a time, to the overall estimate including all variants.

Supplementary Figure 4: Plots for the 10 distinct LDL-C-disease associations significant under FDR correction, for the NPC1L1 genetic risk score.

Unstable angina (411.1)

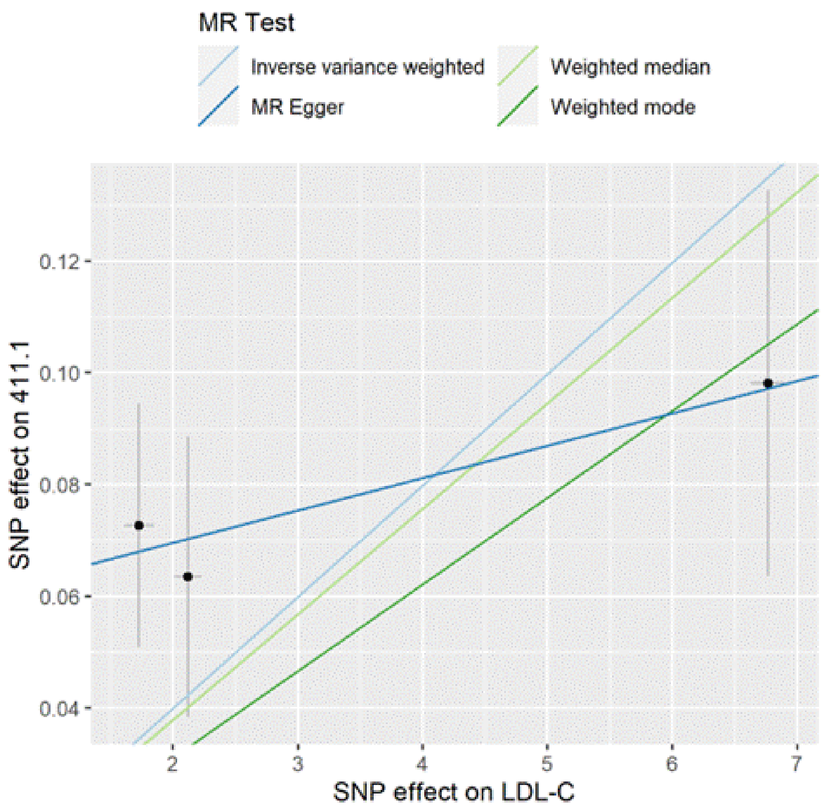

Plot A

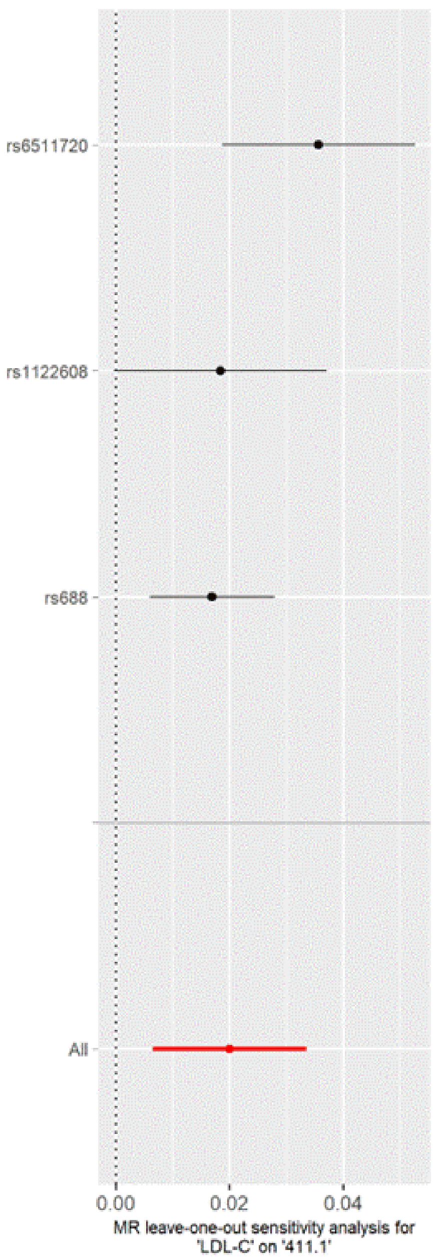

Plot B

Plot A shows the scatter plot of the variant-outcome association estimates for each genetic variant, against the overall estimates for the variant-exposure (i.e. LDL-C lowering) association using different MR methods.

Plot B displays the leave-one-out analysis, which compares the effect estimates when excluding each of the genetic variants in the GRS, one at a time, to the overall estimate including all variants.

Myocardial infarction (411.2)

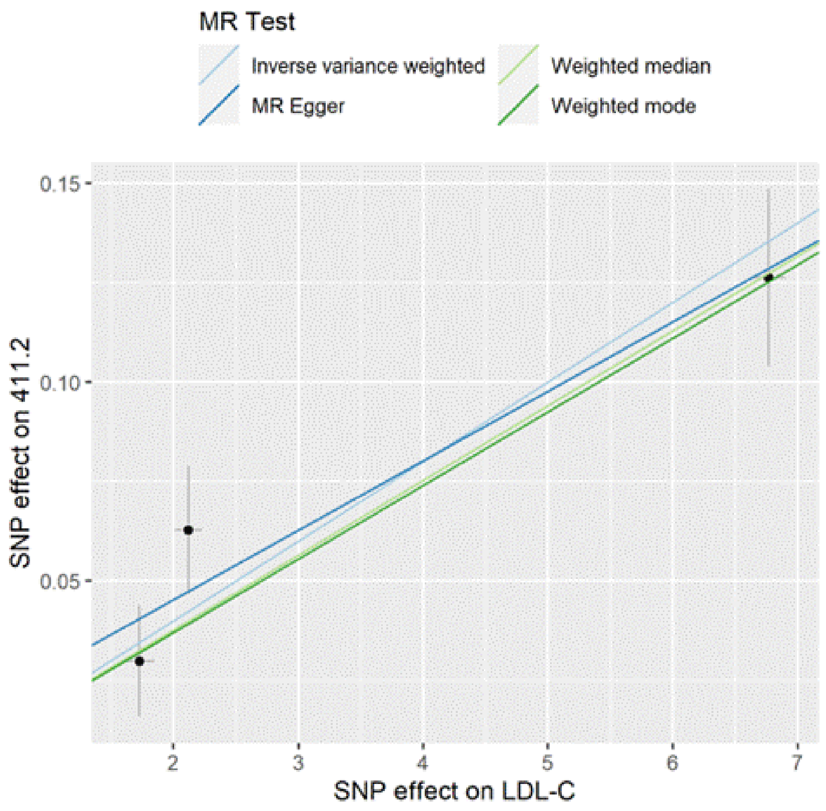

Plot A

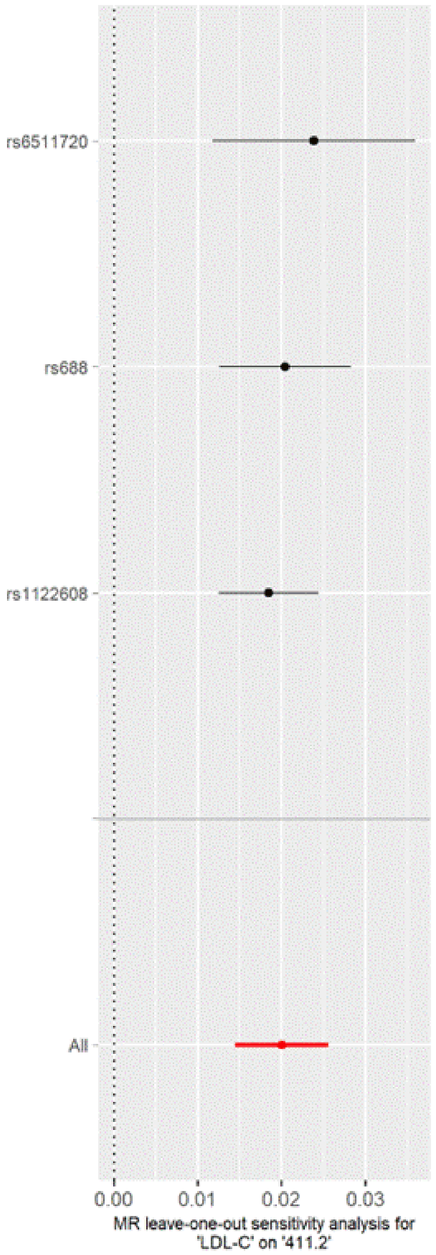

Plot B

Plot A shows the scatter plot of the variant-outcome association estimates for each genetic variant, against the overall estimates for the variant-exposure (i.e. LDL-C lowering) association using different MR methods.

Plot B displays the leave-one-out analysis, which compares the effect estimates when excluding each of the genetic variants in the GRS, one at a time, to the overall estimate including all variants.

Angina pectoris (411.3)

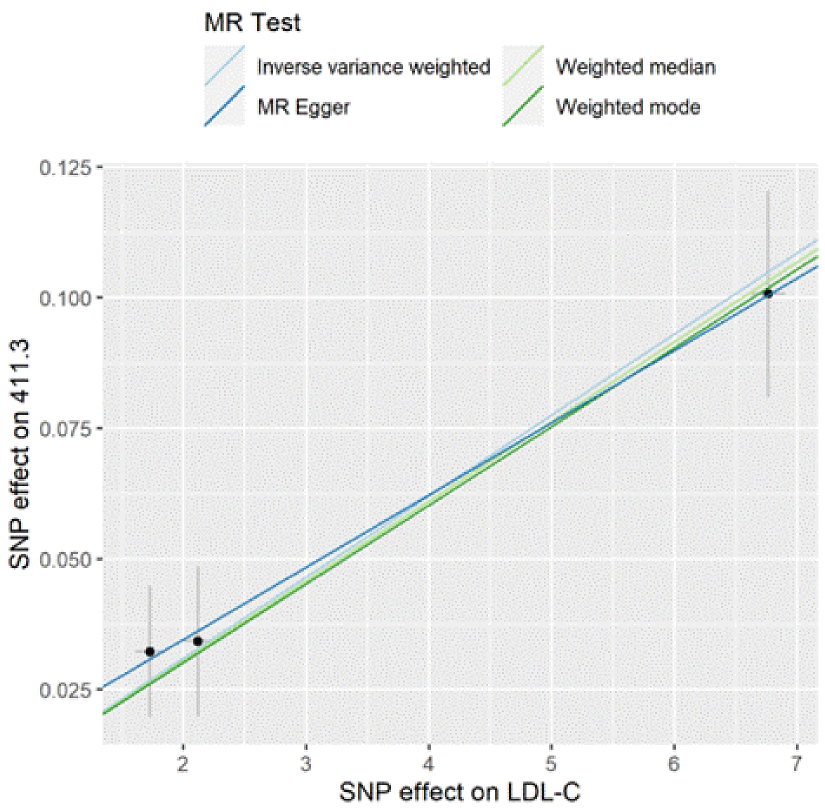

Plot A

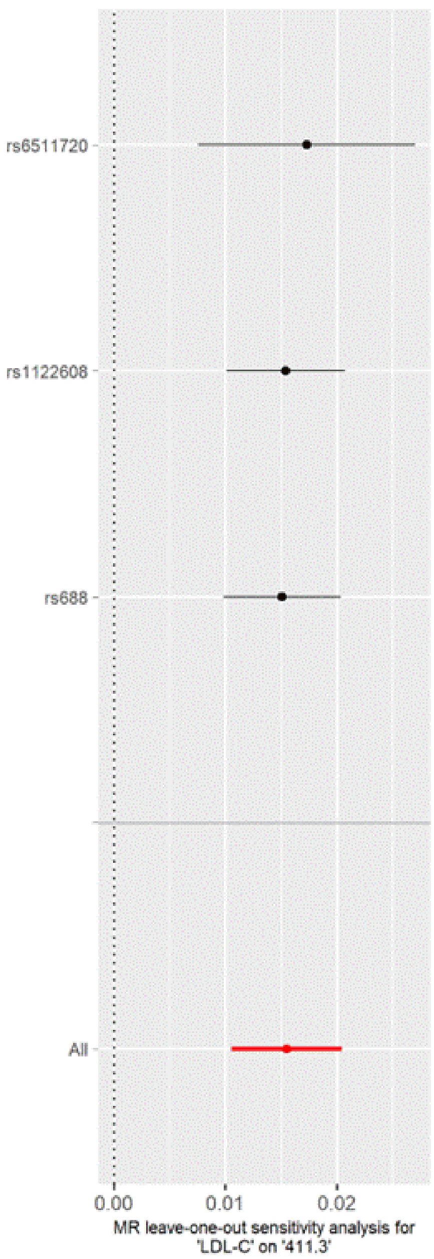

Plot B

Plot A shows the scatter plot of the variant-outcome association estimates for each genetic variant, against the overall estimates for the variant-exposure (i.e. LDL-C lowering) association using different MR methods.

Plot B displays the leave-one-out analysis, which compares the effect estimates when excluding each of the genetic variants in the GRS, one at a time, to the overall estimate including all variants.

Coronary atherosclerosis (411.4)

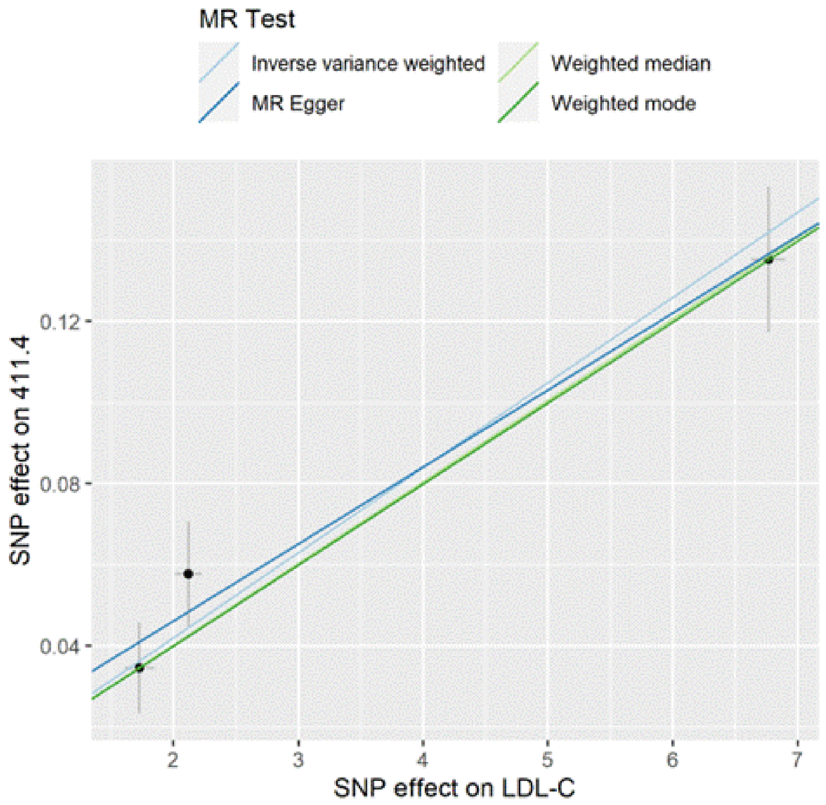

Plot A

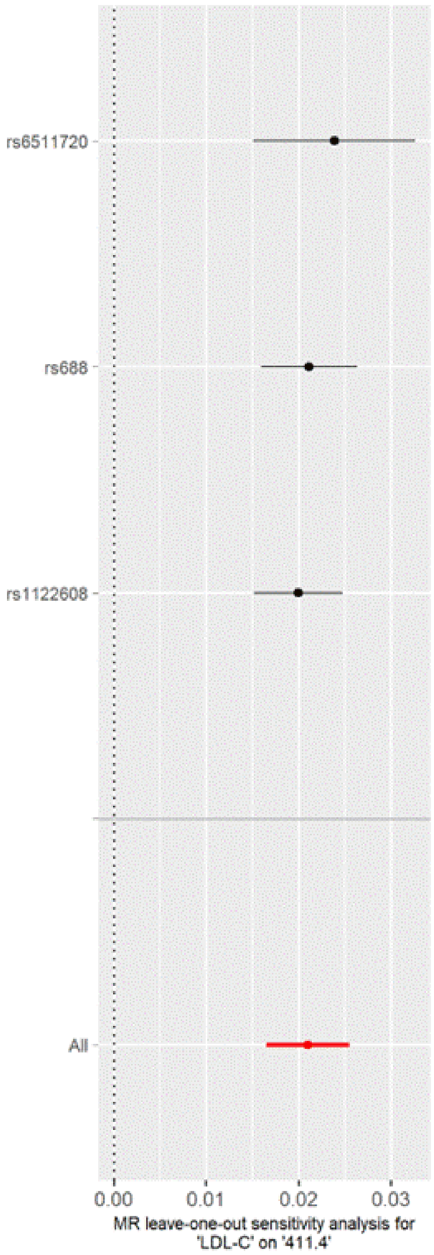

Plot B

Plot A shows the scatter plot of the variant-outcome association estimates for each genetic variant, against the overall estimates for the variant-exposure (i.e. LDL-C lowering) association using different MR methods.

Plot B displays the leave-one-out analysis, which compares the effect estimates when excluding each of the genetic variants in the GRS, one at a time, to the overall estimate including all variants.

Other chronic ischemic heart disease (411.8)

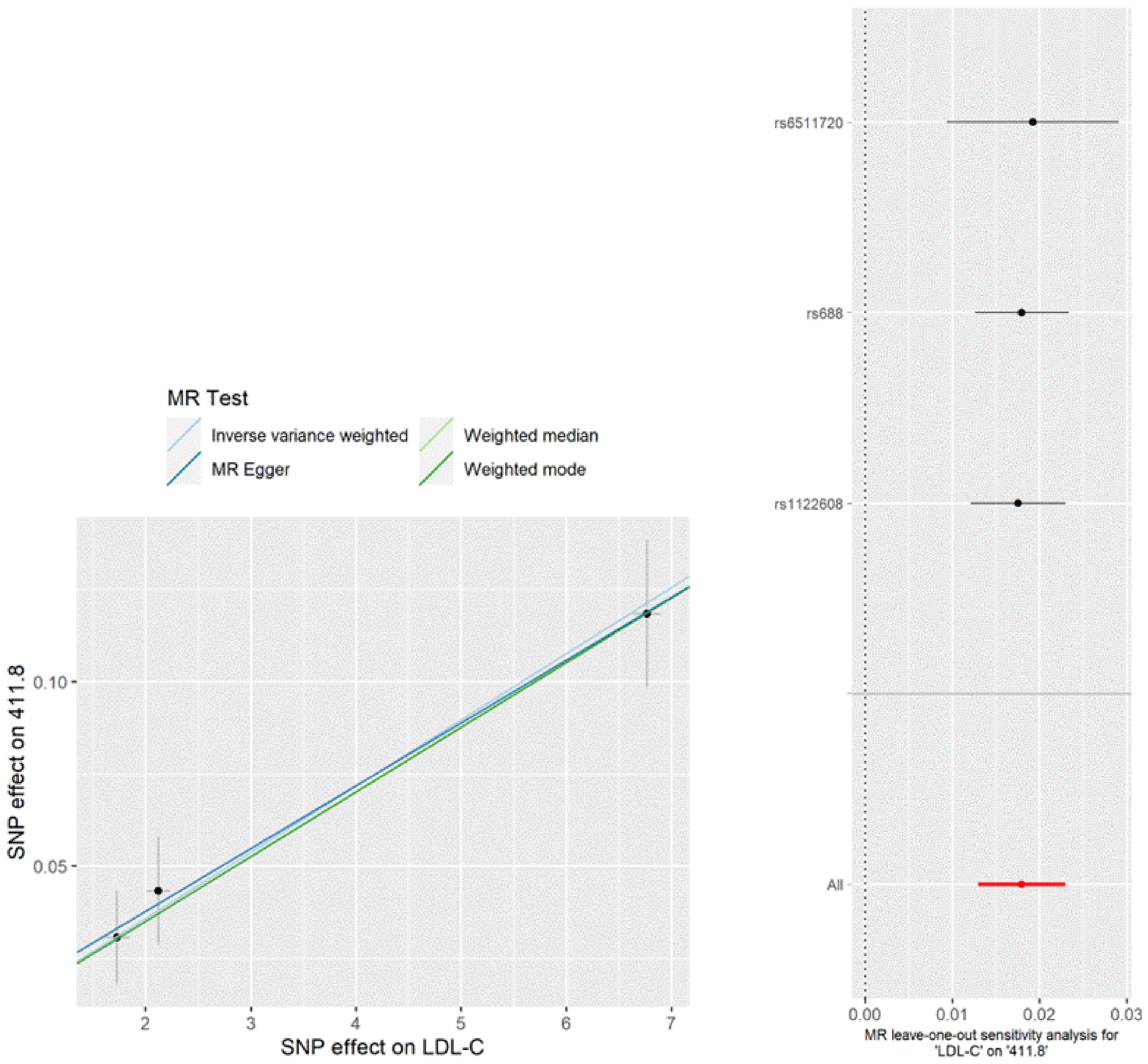

Plot A **Plot B**

Plot A shows the scatter plot of the variant-outcome association estimates for each genetic variant, against the overall estimates for the variant-exposure (i.e. LDL-C lowering) association using different MR methods.

Plot B displays the leave-one-out analysis, which compares the effect estimates when excluding each of the genetic variants in the GRS, one at a time, to the overall estimate including all variants.

Hypertensive heart and/or renal disease (401.2)

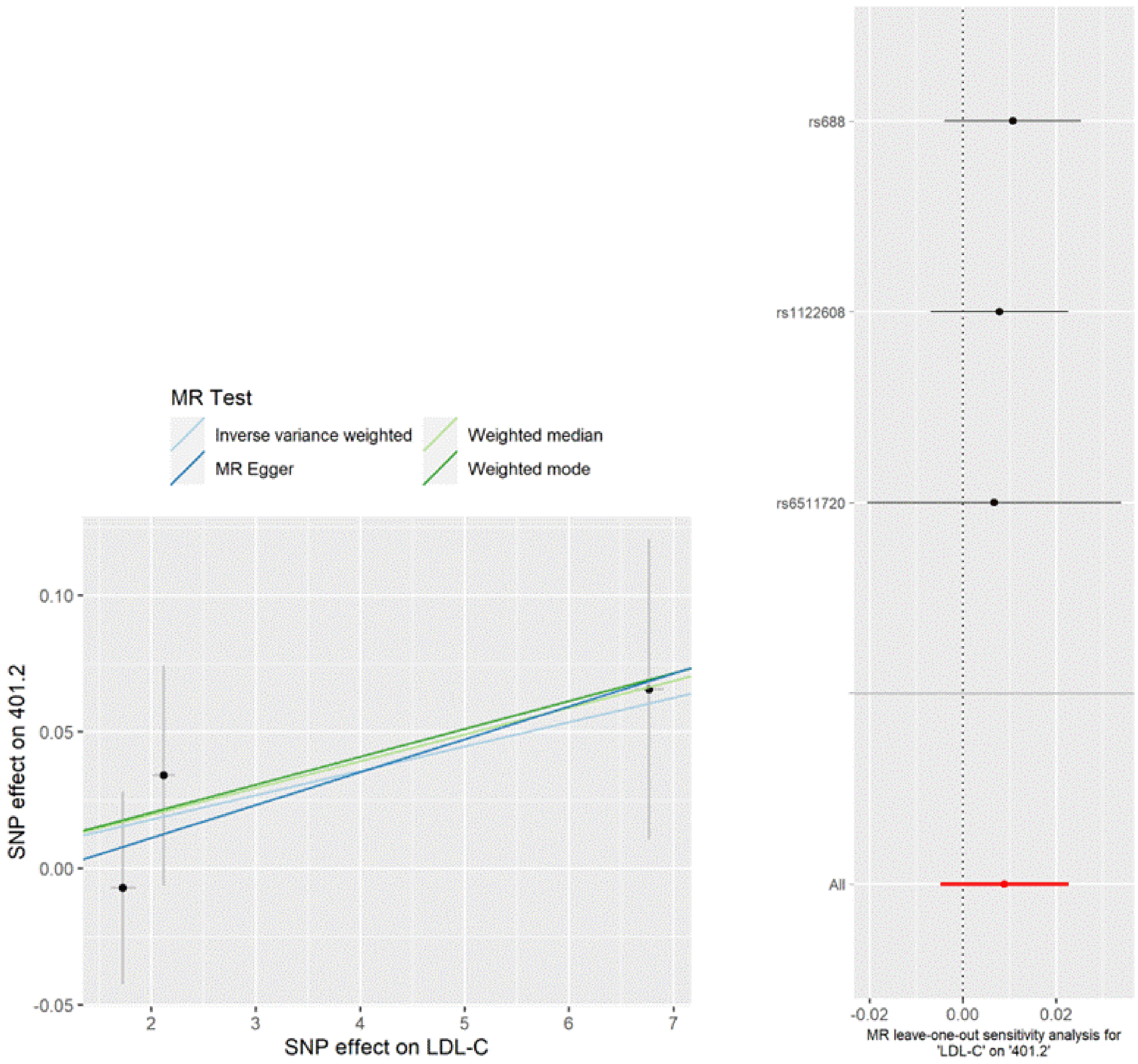

Plot A **Plot B**

Plot A shows the scatter plot of the variant-outcome association estimates for each genetic variant, against the overall estimates for the variant-exposure (i.e. LDL-C lowering) association using different MR methods.

Plot B displays the leave-one-out analysis, which compares the effect estimates when excluding each of the genetic variants in the GRS, one at a time, to the overall estimate including all variants.

Abdominal aortic aneurysm (442.11)

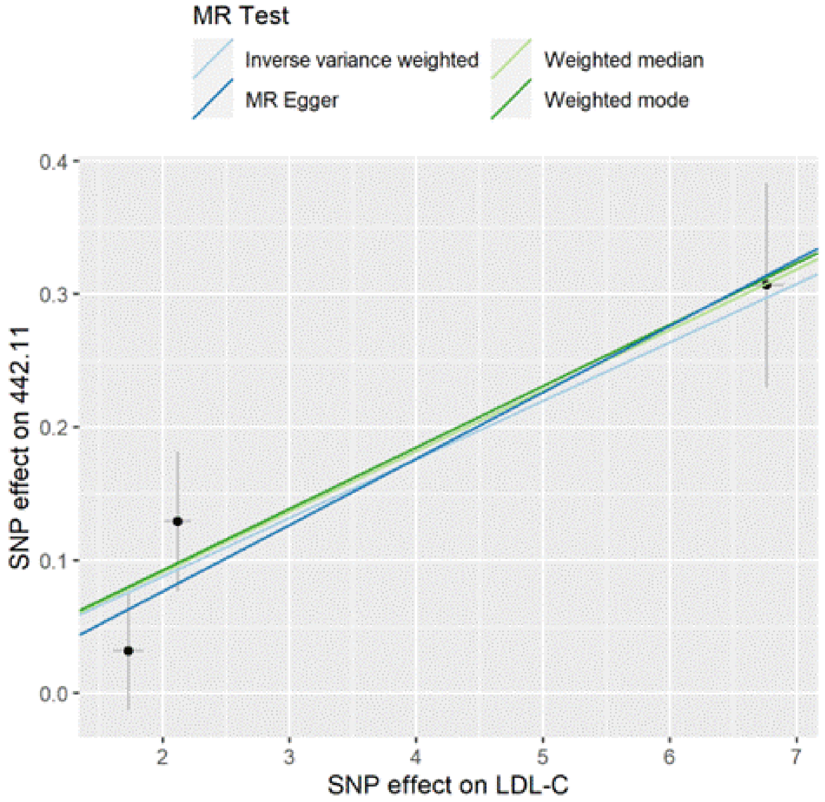

Plot A

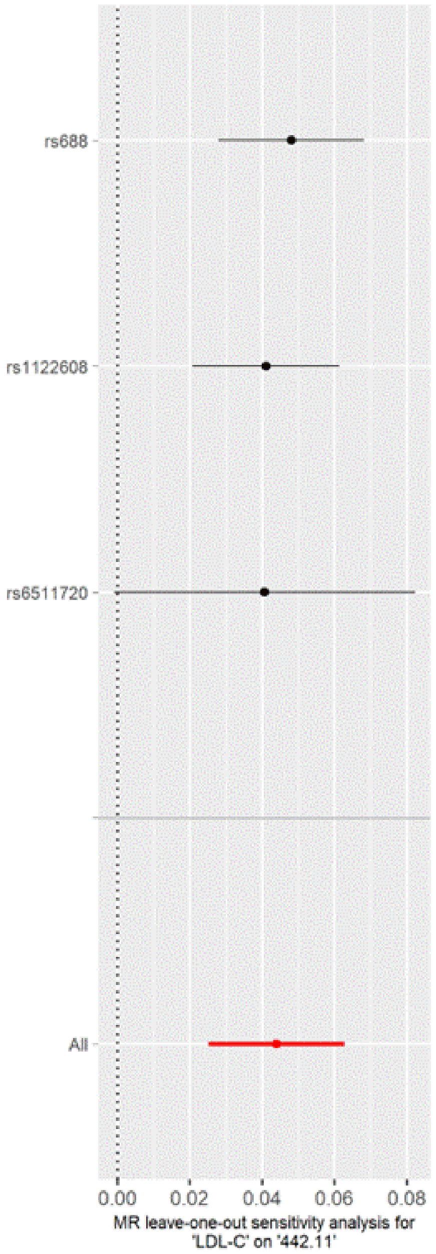

Plot B

Plot A shows the scatter plot of the variant-outcome association estimates for each genetic variant, against the overall estimates for the variant-exposure (i.e. LDL-C lowering) association using different MR methods.

Plot B displays the leave-one-out analysis, which compares the effect estimates when excluding each of the genetic variants in the GRS, one at a time, to the overall estimate including all variants.

Peripheral vascular disease (443)

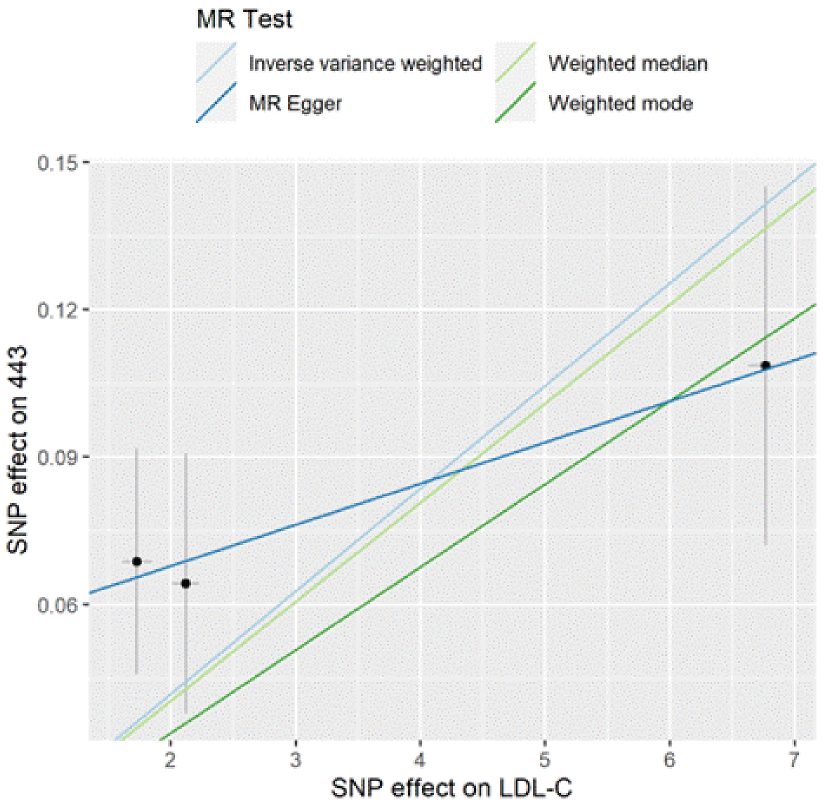

Plot A

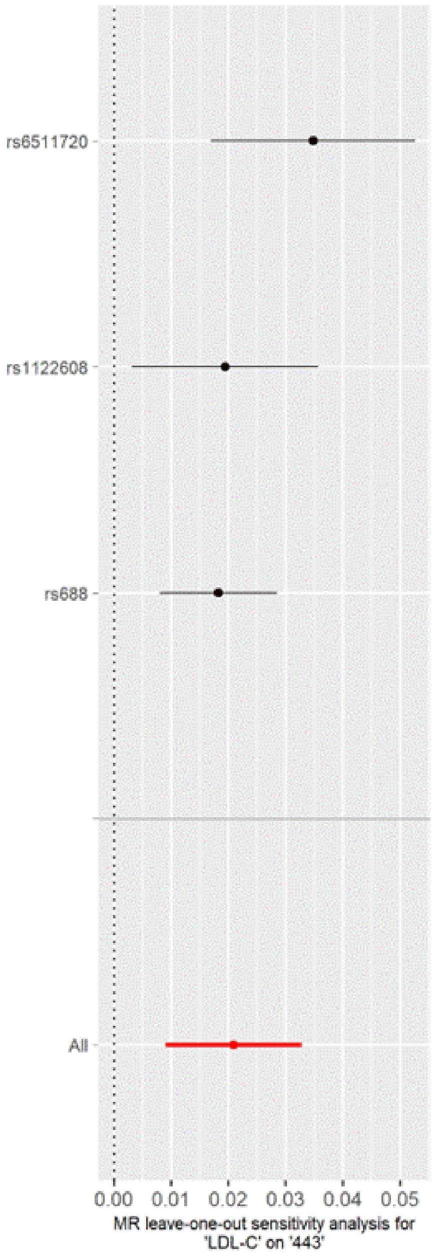

Plot B

Plot A shows the scatter plot of the variant-outcome association estimates for each genetic variant, against the overall estimates for the variant-exposure (i.e. LDL-C lowering) association using different MR methods.

Plot B displays the leave-one-out analysis, which compares the effect estimates when excluding each of the genetic variants in the GRS, one at a time, to the overall estimate including all variants.

Hypercholesterolemia (272.11)

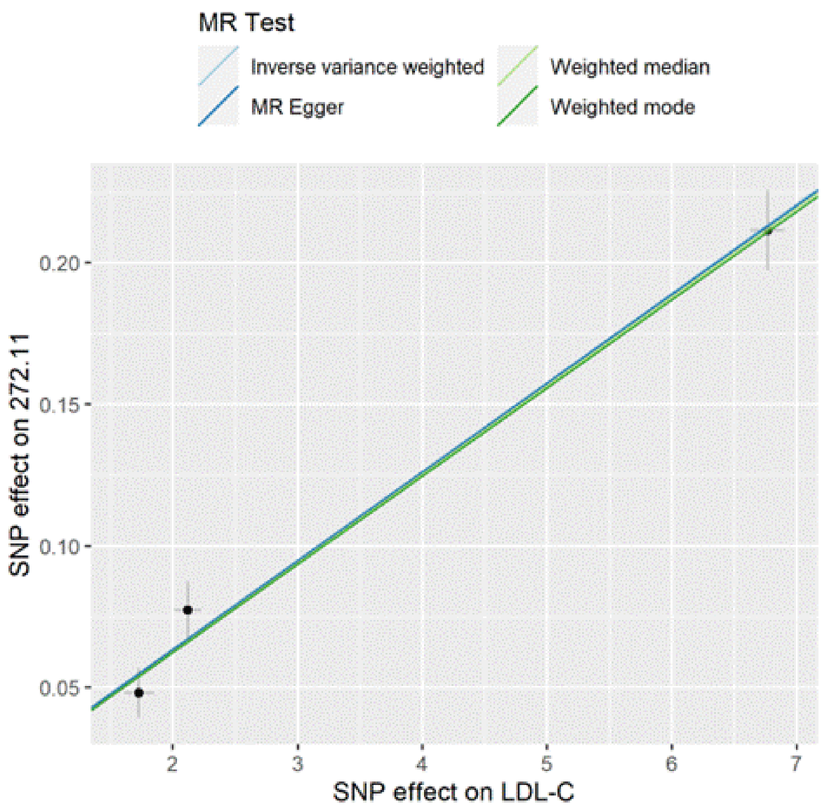

Plot A

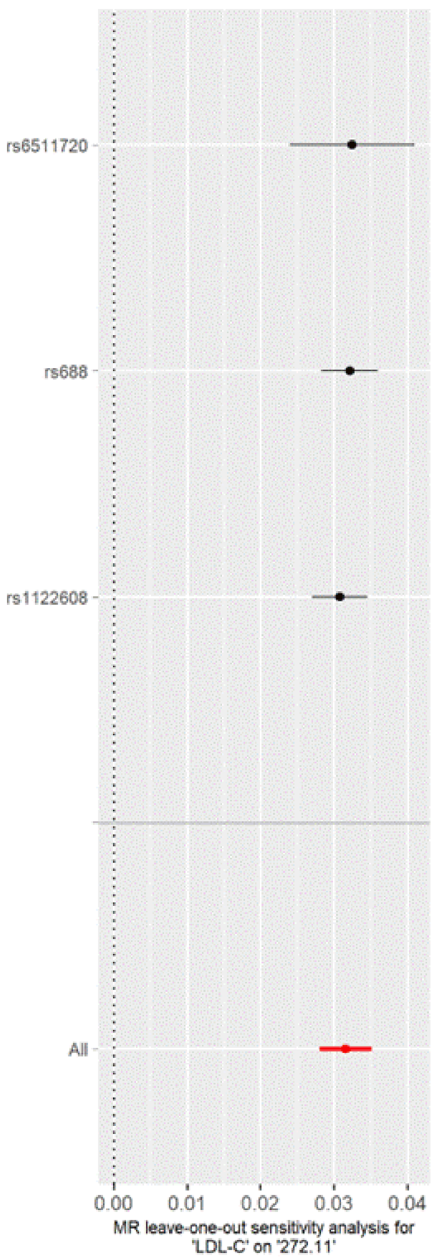

Plot B

Plot A shows the scatter plot of the variant-outcome association estimates for each genetic variant, against the overall estimates for the variant-exposure (i.e. LDL-C lowering) association using different MR methods.

Plot B displays the leave-one-out analysis, which compares the effect estimates when excluding each of the genetic variants in the GRS, one at a time, to the overall estimate including all variants.

Diarrhea (561.1)

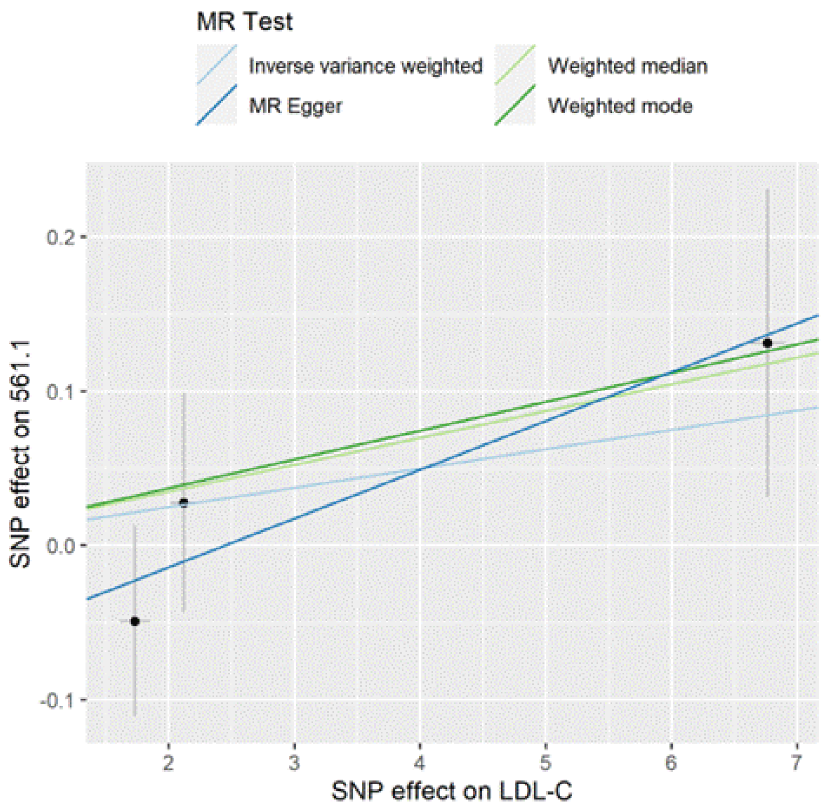

Plot A

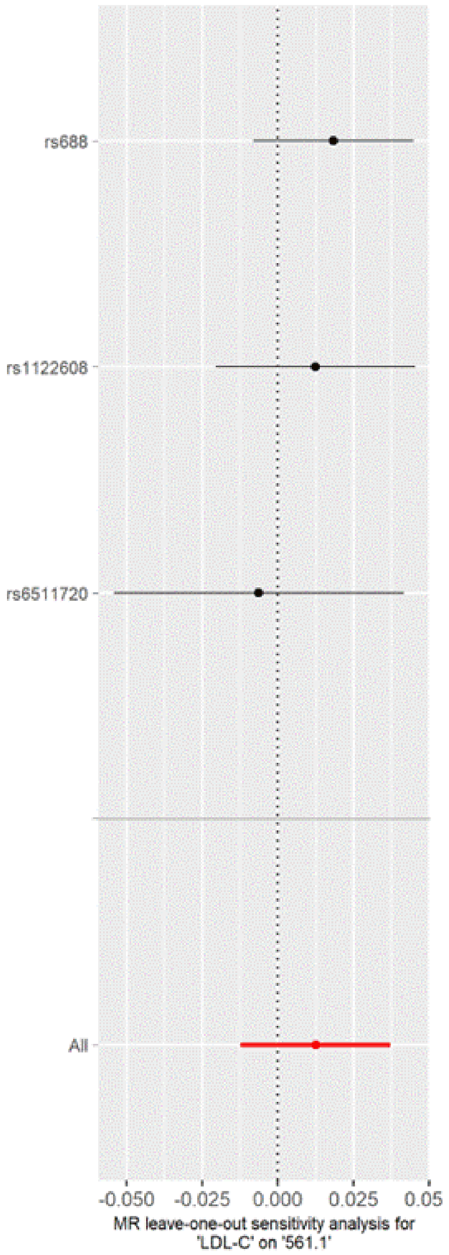

Plot B

Plot A shows the scatter plot of the variant-outcome association estimates for each genetic variant, against the overall estimates for the variant-exposure (i.e. LDL-C lowering) association using different MR methods.

Plot B displays the leave-one-out analysis, which compares the effect estimates when excluding each of the genetic variants in the GRS, one at a time, to the overall estimate including all variants.

Supplementary Figure 5: Plots for the 10 distinct LDL-C-disease associations significant under FDR correction, for the LDLR genetic risk score.

Supplementary Figure 6: Forest plots for the mendelian randomisation analyses on 5 LDL-C lowering hormone biomarker associations, using the LDL-C lowering genetic instruments and stratified by sex. Analyses using inverse-variance weighted mendelian randomisation (IVWMR) are shown. Estimates are odds ratios (OR 95% CI) per 1mg/dL lower LDL-cholesterol.

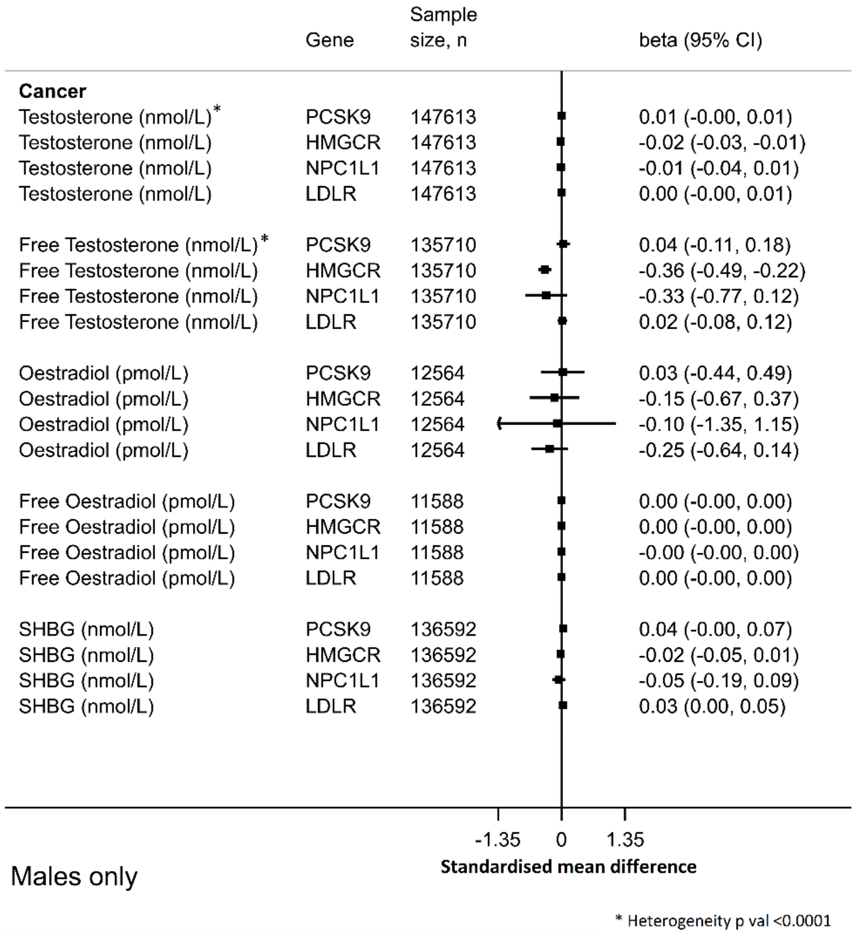

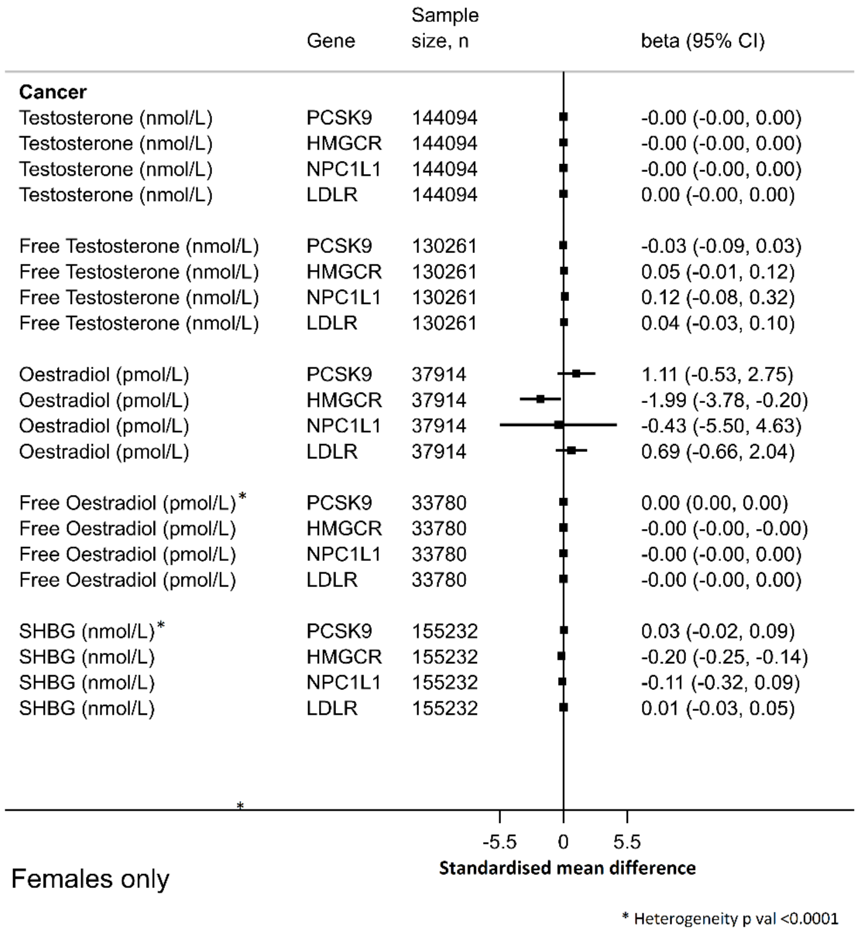

Supplement: Supplementary file 1 — Table S1. Association with LDL‐cholesterol for the 21 LDL‐C lowering variants (one variant excluded from GRS) used to construct the genetic risk score in the UK Biobank and the GLGC consortium (for PCKS9, HMGCR, NPC1L1 and LDLR). Table S2. Construction of LDL‐C lowering genetic risk scores. Table S3. Disease categories and the number of cases with a phecode. Table S4. List of 1135 phecodes tested in the PheWAS analyses. Table S5. Power to detect associations with disease outcomes based on available numbers of cases, alpha 5%, instrument strength determined from the UK Biobank (PCSK9 r2 = 0.12%; HMGCR r2 = 0.15%; NPC1L1 r2 = 0.026%; LDLR r2 = 0.64%) and four different assumed effect sizes per 1 mg/dL decrease in LDL‐C (beta = 0.2; beta = 0.5; beta = 1.0; beta = 1.5). Phenotypes are listed by phecode number, as specified in Table S4. Table S6. Associations of each genetic instrument (PCSK9, HMGCR, NPC1L1 and LDLR) with cardiovascular related biomarkers (from the IVWMR analyses). Table S7. Associations between each LDL‐C lowering genetic risk score and known confounders. Table S8. MR analyses using MR IVW, weighted medium, weighted mode, MR‐Egger and MR‐PRESSO approaches, for 13 disease outcomes identified in the PheWAS of LDL‐C lowering variants under FDR corrected thresholds. Table S9. Replication of MR analyses for disease phenotypes available in the MR‐Base repository. Table S10. List of 52 serum, cardiac imaging, brain imaging and clinical biomarkers. Table S11. MR analyses using MR IVW, weighted medium, weighted mode, MR‐Egger and MR‐PRESSO approaches, for all 52 serum, urine, cardiac imaging, brain imaging and clinical biomarkers, using the PCSK9 genetic instrument. Table S12. MR analyses using MR IVW, weighted medium, weighted mode, MR‐Egger and MR‐PRESSO approaches, for all 52 serum, urine, cardiac imaging, brain imaging and clinical biomarkers, using the HMGCR genetic instrument. Table S13. MR analyses using MR IVW, weighted medium, weighted mode, MR‐Egger an [file BCP-89-2992-s001.pdf]
